# Prenatal and postnatal methamphetamine exposure alters prefrontal cortical gene expression and behavior in mice

**DOI:** 10.3389/fnbeh.2024.1286872

**Published:** 2024-03-05

**Authors:** Philip A. Adeniyi, Tolulope T. Adeyelu, Amita Shrestha, Chin-Chi Liu, Charles C. Lee

**Affiliations:** ^1^Department of Comparative Biomedical Sciences, Louisiana State University School of Veterinary Medicine, Baton Rouge, LA, United States; ^2^Department of Pediatrics, Oregon Health & Science University, Portland, OR, United States; ^3^Department of Veterinary Clinical Sciences, Louisiana State University School of Veterinary Medicine, Baton Rouge, LA, United States

**Keywords:** methamphetamine, dopaminergic signaling, synaptic plasticity, ADCY1, pAKT3, Pi3k-akt

## Abstract

Methamphetamine is a highly abused psychostimulant that substantially impacts public health. Prenatal and postnatal methamphetamine exposure alters gene expression, brain development, and behavior in the offspring, although the underlying mechanisms are not fully defined. To assess these adverse outcomes in the offspring, we employed a mouse model of prenatal and postnatal methamphetamine exposure. Juvenile offspring were behaviorally assessed on the open field, novel object recognition, Y-maze, and forced swim tests. In addition, RNA sequencing was used to explore potential alterations in prefrontal cortical gene expression. We found that methamphetamine-exposed mice exhibited decreased locomotor activity and impaired cognitive performance. In addition, differential expression of genes involved in neurotransmission, synaptic plasticity, and neuroinflammation were found with notable changes in dopaminergic signaling pathways. These data suggest potential neural and molecular mechanisms underlying methamphetamine-exposed behavioral changes. The altered expression of genes involved in dopaminergic signaling and synaptic plasticity highlights potential targets for therapeutic interventions for substance abuse disorders and related psychiatric complications.

## 1 Introduction

Methamphetamine, a highly potent and profoundly addictive substance, has garnered a pervasive presence on the global stage, particularly resonating with adolescents and young adults (Chomchai and Chomchai, 2015; Farrell et al., 2019). Its influence extends across geographical and cultural boundaries, since it operates as a potent central nervous system (CNS) stimulant, inducing profound sensations of pleasure through the disruption of dopamine reuptake from synaptic clefts, prolonging the presence of a neurotransmitter that is integral to the brain’s reward and motivation pathways (Cho and Melega, 2008; Hayward et al., 2010). An ongoing public health concern is methamphetamine abuse during pregnancy, with the United States struggling with the continued impact on maternal and fetal health (Hayward et al., 2010; NSDUH, 2021). However, its developmental influence during the delicate gestational period remains an area imbued with incomplete insights (Behnke et al., 2013; Warton et al., 2018; NSDUH, 2021).

Methamphetamine exerts its primary impact on the brain by influencing monoaminergic neurotransmitter systems. This substance enhances the release of dopamine, norepinephrine, and serotonin while inhibiting their reuptake, leading to heightened neural activity. Moreover, methamphetamine can also facilitate the non-vesicular release of dopamine via dopamine transporters (Bennett et al., 1998; Goodwin et al., 2009; Xie and Miller, 2009). The rewarding effects of the drug are particularly evident through the excessive release of dopamine in crucial brain regions like the nucleus accumbens. Furthermore, documented neurotoxic effects of methamphetamine involve damage to dopamine and serotonin terminals, with oxidative stress, inflammation, and excitotoxicity playing key roles in this process (Cadet et al., 2001; Sulzer et al., 2005; Adeniyi et al., 2016b).

The continuum of methamphetamine usage during pregnancy significantly affects maternal physical and psychological well-being, ultimately impacting the developing fetus through prenatal drug exposure (Smith et al., 2015a,b; Eze et al., 2016). This results in a range of deleterious changes in the offspring, including cardiac anomalies, cleft lip, biliary atresia, and stillbirth (Smith et al., 2015a,b). Neurological consequences manifest as autism spectrum traits, speech impediments, an inherent wariness of unfamiliar individuals, and a noticeable scholastic lag, particularly in mathematics and language (Billing et al., 1988; Eze et al., 2016). Methamphetamine can affect lactation, nurturing behaviors, and epigenetic influences (Smith et al., 2015a,b). These behavioral changes provide a more comprehensive context for the maternal–infant relationship in relation to methamphetamine exposure. This highlights the urgent need for comprehensive research and awareness regarding the profound implications of maternal methamphetamine exposure on both maternal and infant outcomes.

Methamphetamine exposure affects pivotal brain regions involved in higher-order cognition, such as the prefrontal cortex (PFC), hippocampus, striatum, and ventral tegmental area (VTA) (Ogundele and Lee, 2018; Li et al., 2021; Sanjari Moghaddam et al., 2021). Of these, the prefrontal cortex is of particular interest due to its multifaceted role in cognitive behaviors, decision-making processes, social dynamics, and the consolidation of memory (Sultana et al., 2021; Armenta-Resendiz et al., 2022; Gandhi et al., 2023). Therefore, this study explored the role of prenatal and postnatal methamphetamine exposure and its subsequent impact on behavior and gene expression related to the PFC in the offspring.

## 2 Materials and methods

### 2.1 Animals

Young adult C57BL/6 J mice (3–6 months old of both sexes) were obtained from our colony housed in the vivarium at the Louisiana State University School of Veterinary Medicine. All the procedures were approved by the Institutional Animal Care and Use Committee (IACUC) of LSU. Animals were housed under a 12-h light and dark cycle (light on 7 a.m.–7 p.m.) in a group of two females per male and were allowed food and water *ad libitum*. The temperature was maintained at 23 ± 1°C.

#### 2.1.1 Drug administration

**Figure 1:**
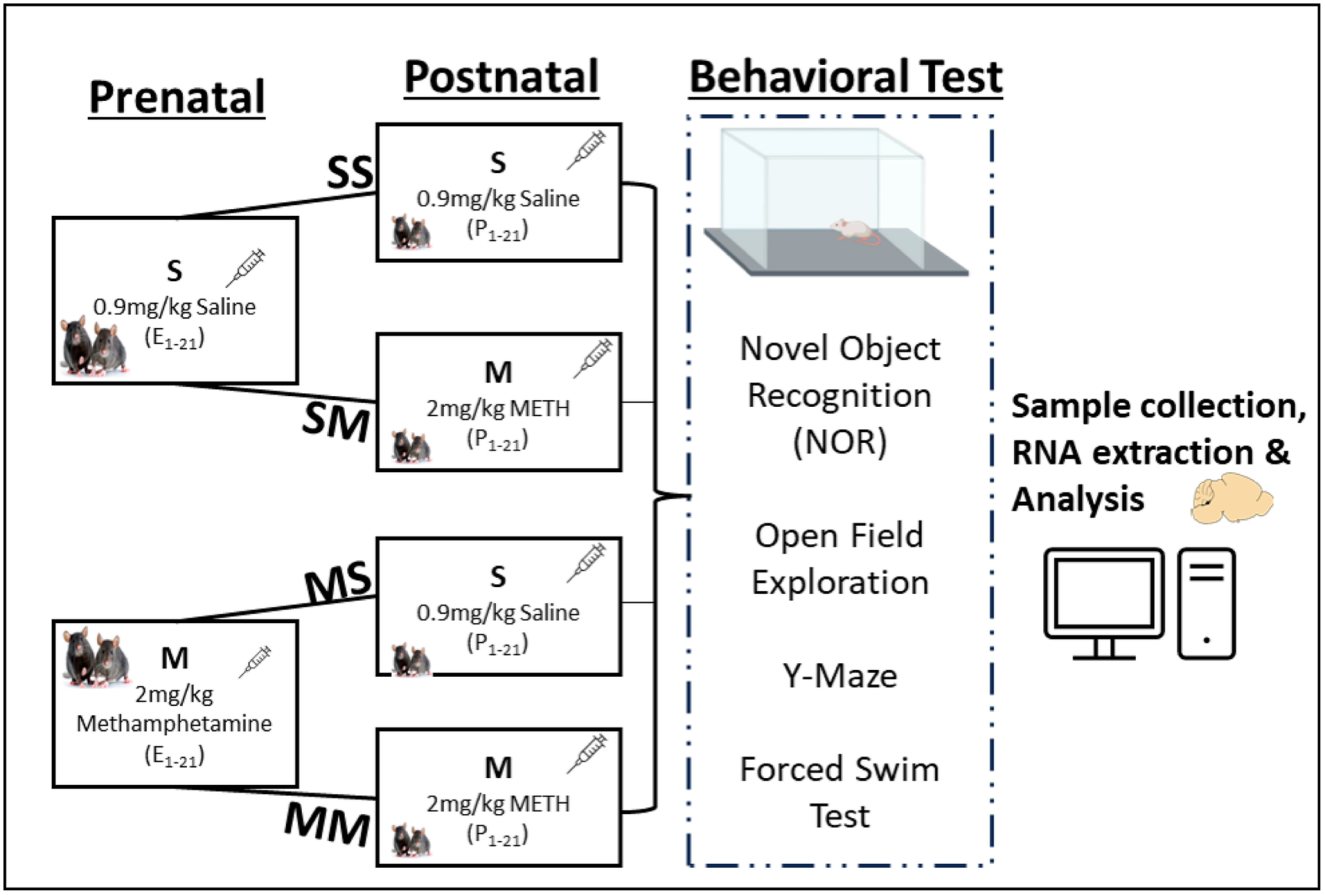
Experimental framework and behavioral analysis. Experimental groups and timeline for prenatal or postnatal methamphetamine (M) or saline (S) exposure.

Methamphetamine hydrochloride purchased from Sigma (St. Louis, MO) was dissolved in 0.9% saline, and sterilized to prepare the injectable solution. Methamphetamine solutions (2.0 mg/kg) were administered subsequently at 10 mL/kg body weight during the experimental epochs (Figure 1), which was the optimum average dose identified from prior studies (Itzhak et al., 2015; Dong et al., 2022).

*Prenatal exposure to methamphetamine*: Pregnant dams were segregated randomly into saline (S) or methamphetamine (M) prenatal injection groups. Diestrus female mice were housed with a mature healthy male (1 male: 2 females) for 24 h; the females were introduced to the cage of the male. Males were removed from the cage after 24 h and the date marked as prenatal day 1 (PND 1). Methamphetamine (2.0 mg/kg) was administered subcutaneously every other day and body weights were assessed to screen pregnant mice during the first two weeks, and to determine weight changes throughout the three trimesters (see Figure 1).

*Postnatal exposure to methamphetamine*: After pups were delivered, the dams in the S and M prenatal groups were further segregated for subsequent postnatal injections. The dams in the prenatal S group were further divided into saline-saline (SS) and saline-meth (SM) postnatal injection groups. Similarly, the prenatal M group was further divided into meth-saline (MS) and meth-meth (MM) groups of about 4 mothers each (Figure 1). The dams in SS and MS groups were given saline (s.c.) only from postnatal day one (P1) until P28 while those in SM and MM groups were given methamphetamine (2.0 mg/kg, s.c.) from P1 till P28 when the pups were weaned. In summary, the SS group was exposed to saline during the pre-and -postnatal period, the SM group was exposed to saline and 2.0 mg/kg of meth during the pre- and post-natal period respectively, MS group was exposed to 2.0 mg/kg of meth and saline during the pre- and post-natal period respectively, while MM group was exposed to 2.0 mg/kg of meth during the pre- and post-natal period (Figure 1). The litters were housed in group of five mice in a box, with majority coming from the same mother. The litters were selected randomly taking into consideration equal distribution in each group. The use of a crossover design in the study aims to minimize variability between participants and enhance the efficiency of comparing treatments within the same group. This design allows each participant to receive both treatments, serving as their own control, thereby reducing potential confounding factors.

### 2.2 Behavioral evaluation

Cognitive and affective behaviors were evaluated postnatally after P28 using the open field, novel object recognition (NOR), Y–maze, and forced swim tests, as we have previously described (Adeniyi et al., 2016a,c; Sultana et al., 2019; Sultana and Lee, 2020). The mice were divided into four groups (SS, SM, MS, and MM) with n = 6–8 mice per group. There was no discrimination with regard to the sex of the animals - male and female mice were treated equally. The mazes underwent thorough cleaning with 70% ethanol before the tests to ensure uniformity in cleaning procedures across different equipment, especially considering that the apparatus were utilized for multiple animals in the experiments. The use of ethanol serves the purpose of eliminating potential olfactory cues and contaminants, thereby establishing a neutral testing environment conducive to behavioral studies. Additionally, the choice of a 70% ethanol solution ensured rapid evaporation and minimized any lingering effects on the experimental conditions.

*Open field test.* An open field arena (75 cm × 75 cm × 45 cm) enclosed with an opaque wall was used for this behavioral test. During this task, the mouse was placed in the center of the arena and allowed to move freely for 5 min while being recorded with an overhead camera. The footage was analyzed for distance covered, speed, mobility/immobility, and zones explored by the mice, using ANY-maze software (ANY-maze, Wood Dale, IL).

*Novel object recognition.* The novel object recognition test (NOR) evaluated the preference of test mice for a novel object compared to an old object and is implicated in assessing prefrontal cortical function, among other regions (Adeniyi et al., 2016a,c; Lueptow, 2017). The task involves three phases which include habituation, familiarization, and memorization. The testing chamber dimensions were 75 cm × 75 cm × 45 cm, and objects utilized were of similar sizes (~16.0 cm^3^), but different shapes and colors. Animals were habituated to the testing environment prior to the experiment. The habituation phase lasted 1–2 h, followed by a training phase with two identical objects for 10 min. The interval between the training and memory test phase was 1 h. During the memory test, one familiar object is replaced with a novel one, and animals were observed for 5 min. These parameters align with our prior established NOR protocols (Adeniyi et al., 2016a,c). During the familiarization phase, the mice were exposed to two similar objects at opposite ends of the experimental chamber. During the memory test phase, the mice were introduced to a new object and one of the old objects. All behavioral recordings were then analyzed using ANY-maze software to calculate the discrimination index (DI) of the novel object: DI = Time (new) − Time (old) /Time (Total).

*Spontaneous Alternations in the Y-Maze.* The Y-Maze is a classic behavioral test for evaluating spatial working memory (Garcia and Esquivel, 2018; Sultana et al., 2019). The Y-maze was custom made and had three arms of equal length (35 cm), width (5 cm) and height (10 cm), which were connected at the center at an angle of 120°. Animals were habituated (a day before the texting and on the testing day) to the testing environment prior to the experiment. Prior to the start of the experiment, the chamber was wiped with 70% EtOH followed by distilled H_2_O. The animal was placed in the center of the Y-maze; all the arms were opened, and the animal was allowed to roam for 7 min to test their exploratory and alternation behavior. The alternations were scored manually, where a sequence of arm visits (e.g., A to B to C) without repetition was counted as one complete alternation. The total time spent in each arm, the number of arm entries, the number of entries made into each arm, and the sequential list of arms entered were then used to assess alternation. Spontaneous alternation occurred when a mouse entered a different arm of the maze in each of 3 consecutive arm entries and was calculated using Anymaze software: Spontaneous alternation (%) = # spontaneous alterations × 100 / (Total number of arm entries – 2).

*Forced Swim Test.* As a metric of despair-related behaviors, we utilized a modified Porsolt forced swim test. In this test, animals were placed in a 3 L beaker that contained 2.5 L of distilled water and were video recorded for 6 min. Despair-related behaviors in the Forced Swim Test (FST) are typically defined as immobility or minimal movement, reflecting a state of behavioral despair. This behavior is considered a response to the perceived inescapability of the situation (Porsolt et al., 1977). ANY-maze was used to score the percent mobility time from a total 4-min testing period, following an initial 4-min acclimation period. Utilizing Any-maze for the FST ensured an accurate and unbiased assessment of both mobility and immobility, contributing to a thorough evaluation of the animal’s behavior in the study. As part of our analysis, we also meticulously inspected the results for validity.

### 2.3 RNA extraction, sequencing, and transcriptomic analysis

After all behavioral assays were completed, mice were euthanized, and the prefrontal cortex was dissected, kept in TRIzol, and stored at −80°C. RNA was extracted from 5 samples per group using the Purelink RNA mini kit (Invitrogen, CA, United States) following the manufacturer’s instructions. The quality and quantity of the RNA were measured using a Nanodrop Spectrophotometer ND-1000 UV/Vis (Thermo Scientific) and using the nucleotide fragment analysis through the Advanced Analytical Fragment Analyzer (Agilent, Santa Clara, CA). The quality of RNA in each sample was ≥8.0. At least 3 samples per group with optimum RNA quality were selected for processing by Novogene Co., Ltd. (Durham, NC) for RNA sequencing using the Illumina platform. Novogene performed the RNA quality evaluation (yield, purity, and integrity), construction of the cDNA library, and Illumina sequencing as detailed in previous reports (Lam et al., 2021; Naldurtiker et al., 2023). The differential expression analysis was conducted by the DESeq2 R package (Love et al., 2014) with a criterion of log2 fold-change < −2 or > 2 and q-value (adjusted *p*-value) < 0.05.

#### 2.3.1 Functional enrichment analysis and protein interaction network

Pathway analyses were conducted for the differentially expressed genes (DEGs) using gene ontology biological processes (GO-BP) and the KEGGs pathways analyses. Significant enrichments were only assigned to processes with adjusted *p* < 0.05. We also constructed a Methamphetamine Derived Protein Interaction Network (MDPIN) based on the protein interaction data from the MM groups, and analyzed the network, calculating the topological properties, e.g., degrees, between the centrality of each node in the network using Cytoscape software (Kohl et al., 2011). The network has 648 nodes and 2,655 edges. From this network, we identified hubs and bottlenecks as the top 20% of proteins in this network ranked by their degrees and betweenness centrality (Yu et al., 2007).

### 2.4 Statistical analysis

In this study, a two-way ANOVA with *post hoc* (LSD) was performed to compare the behavioral studies among the 4 groups. Normality of residuals from the models were confirmed by quartile plots. Student’s *t*-test was performed to compare the body and birth weight of saline (S) and meth (M) groups. Boxplots were used to show the distribution of the behavioral data (*n* = 6–8 animals per group). Analyses were performed using JMP Pro 17.0.0 (JMP Statistical Discovery LLC, Cary, NC) and graph plotting was performed using OriginPro 2021b software (OriginLab, Northampton, MA). Differentially expressed genes were obtained by comparing the gene expression of other groups to SS, and genes with significance *p* < 0.05 were considered differentially expressed. Pathways of differentially expressed genes was analyzed using R-program, ClusterProfiler, as previously described (Yu et al., 2012; Wu et al., 2021).

## 3 Results

### 3.1 Body weight changes and number of pups

The body weight of pregnant mice grew consistently in both the prenatally treated methamphetamine and control groups from gestation till parturition, with no significant difference between the two groups (*p* > 0.05), although the meth-treated group had a slightly higher body weight from day 5 to 20 (Supplementary Figure S1A). The average number of pups per mother in both methamphetamine (5.50) and control (7.25) groups exhibited no significant difference (*p =* 0.2139) (Supplementary Figure S1B). Also, there is no significant difference in the birth weight between meth-treated and control (*p* = 0.7011) (Supplementary Figure S1A).

### 3.2 Behavioral analysis of offspring

**Figure 2:**
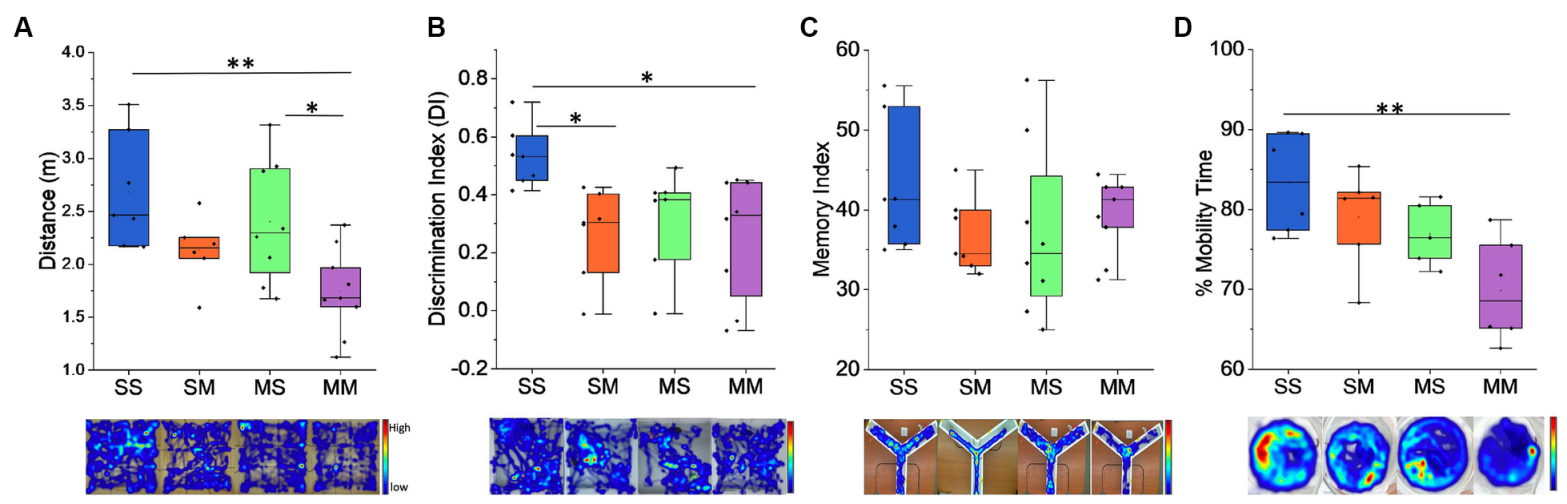
Behavioral results. **(A)** Distance traveled by the mice during open field exploration. **(B)** Discrimination index for novel object exploration during NOR test. **(C)** Memory index analysis during the Y-Maze test. Administration of methamphetamine reduces the exploration time in mice and the ability to detect novel objects, although we observe no changes in the memory index during Y-Maze. **(D)** Percent mobility time during Forced Swim Test. Representative heatmaps are shown under each graph for the behavioral task performed (*p < 0.05, **p < 0.01).

The impact of methamphetamine exposure was assessed on a battery of behavioral tests. In the open field test, we observed that pre-natal exposure to methamphetamine caused a significant decline in distance covered (F_1,26_ = 11.88, *p* = 0.0019) (Figure 2A) with an average of 2.54 ± 1.23 m covered by those exposed to saline compared to 1.94 ± 1.25 m in the methamphetamine exposed group. Similarly, we observed the MM cohort covered an average distance of 1.74 m which was significantly lower to SS with an average distance of 2.68 m (*p* = 0.0031) and MS – 2.40 m (*p* = 0.003), but not SM – 2.13 m (*p* = 0.428) (Figure 2A). In the novel object recognition task, we observed that prenatal exposure to methamphetamine caused a significant decline in the discrimination index: (F_1,25_ = 6.92, *p* = 0.0143) (Methamphetamine: 0.26 ± 0.04 vs. Saline: 0.43 ± 0.01) (Figure 2B). Similarly, comparison across the groups showed that the discrimination index was significantly lower in the MM with an average score of 0.25 (*p =* 0.019) and SM groups – 0.27 (*p =* 0.034), relative to the SS group – 0.53 (Figure 2B), while no difference was observed with the MS group – 0.32. We also observed no significant difference (*p* > 0.05) in spatial working memory as observed in the Y–maze test between the control and methamphetamine-exposed groups—(F_1,27_ = 2.811, *p* = 0.105) (Figure 2C). Finally, results from the forced swimming test (FST), revealed that prenatal exposure to methamphetamine does not cause significant decline in the mobility time for the mice (F_1,28_ = 0.062, *p* = 0.806) (Figure 2D). Comparatively, we observed that mice in the MM group had a lower percentage of mobility time compared to the SS group (69.84% vs. 83.29%, *p =* 0.0042), unlike SM (79.05%, *p* = 0.603) and MS (76.9%, *p* = 0.305) groups (Figure 2D).

### 3.3 RNA sequencing of prefrontal cortical gene expression

**Figure 3:**
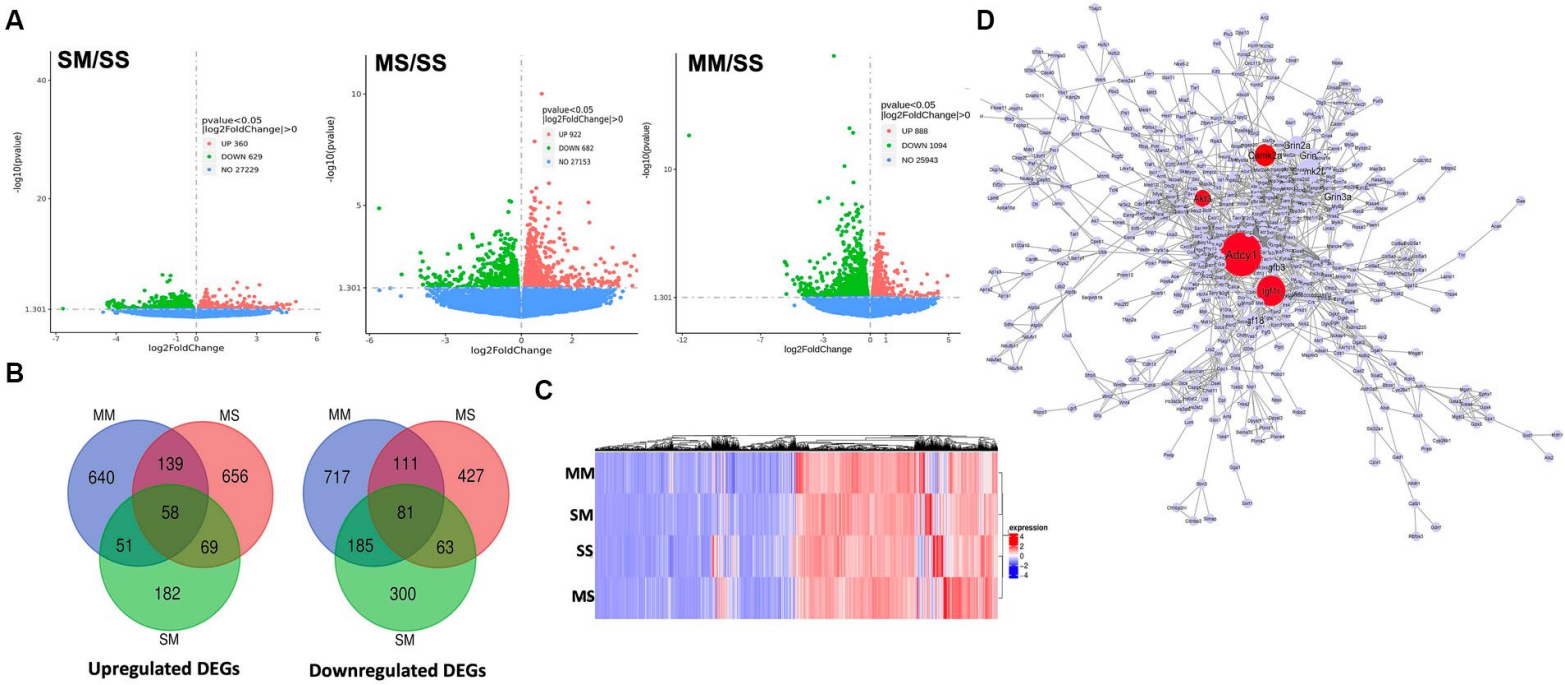
RNA sequencing of differentially expressed genes in the prefrontal cortex. **(A)** Volcano plot showing the numbers of upregulated and downregulated differentially expressed genes in SM, MS, and MM groups in comparison to control (SS). **(B)** Overlap between differentially upregulated and downregulated genes in each group. **(C)** Heatmaps showing clusters of differentially expressed genes in different groups. **(D)** Protein interaction network showing ADCY1, Akt3, and CAMk2a (in red) are hubs and highly differentially expressed during methamphetamine treatment.

The RNA sequencing data revealed alterations to gene transcription; the SM group shows 360 upregulated genes, 629 downregulated genes, and 27,229 genes with no significant difference (Figure 3). In MS, there were 922 upregulated genes, 682 downregulated genes, and 27,153 genes with no significant difference expression while the MM group has 888 upregulated genes, and 25,943 genes with no significant expression (Figures 3A–C). We observed 197 upregulated genes that were co-expressed by MS and MM groups, 109 upregulated genes that were co-expressed by SM and MM groups, and 127 upregulated genes that were also co-expressed by SM and MS groups, while the MM, MS, and SM groups co-expressed 58 genes (Figure 3B). There were 192 downregulated genes co-expressed by MS and MM groups, 266 downregulated genes co-expressed by SM and MM groups, and 144 upregulated genes were also co-expressed by SM and MS groups, while the MM, MS, and SM groups co-expressed 81 genes (Figure 3B). A list of differentially expressed genes is provided in Supplementary Table S1.

### 3.4 Pathway analysis of differentially expressed genes (DEGs)

**Figure 4:**
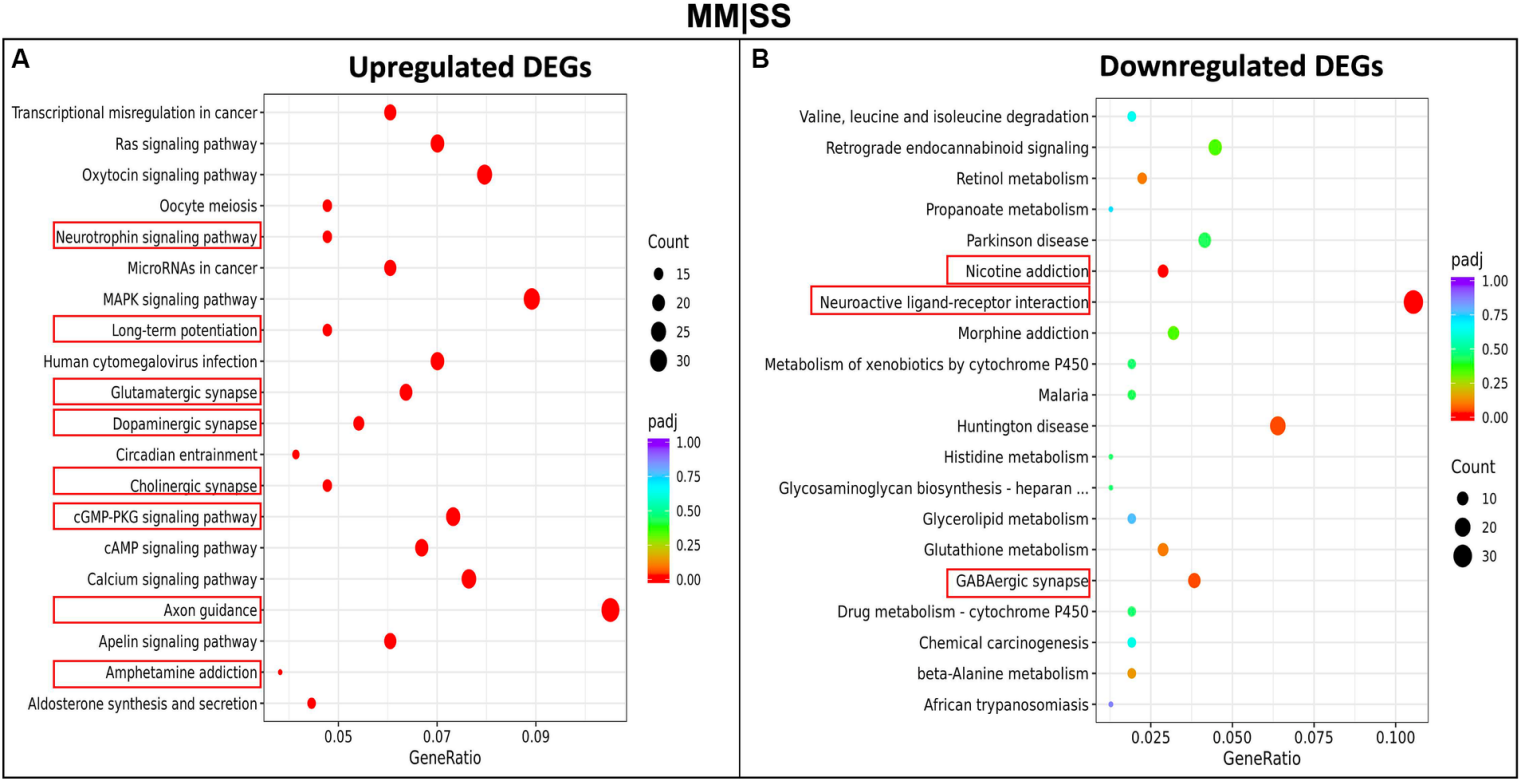
KEGG-pathway analysis of differentially expressed genes (DEGs). **(A)** Enriched pathways in upregulated genes between MM in comparison to control (SS). **(B)** Enriched pathways in the downregulated MM genes in comparison to control (SS). The red rectangular boxes are the significantly enriched neurological pathways or processes.

We investigated the pathways and biological processes of differentially expressed genes (DEGs) in the PFC, using the KEGG (Kyoto Encylopedia of Genes and Genomes) pathway (Figure 4). The KEGG pathway analysis, using an adjusted *p*-value < 0.05, shows upregulated differentially expressed genes from the MM are involved in several neurological-related processes such as amphetamine addiction, cholinergic synapses, axon guidance, long-term potentiation, glutamatergic synapse, dopaminergic synapse, cAMP signaling pathways (Figure 4A). Interestingly, no significant difference was observed between the processes for the upregulated genes in the SM group and control SS group (Supplementary Figure S2). However, we observe that DEGs in the SM groups are significantly involved in processes such as long-term potentiation, glutamatergic synapses, cholinergic synapses, and other interesting pathways such as PI3K and MAPK signaling (Supplementary Figure S2A). The downregulated DEGs in the MM group are involved in processes such as nicotine addiction, morphine addiction, neuroactive ligand-receptor interactions, and GABAergic synapse function (Figure 4B). Other KEGGs pathways downregulated DEGs in other groups (SM and MS) are highlighted in Supplementary Figure S3.

The Gene Ontology biological process (GO-bp) reflects the experimental functions carried out by a set of genes (Supplementary Figures S4, S5). We observed that DEGs within the MM groups were associated with neurological developmental processes such as axon-genesis, synapse organization, and postsynaptic density (Supplementary Figure S4). GO-bp of DEGs within the MS groups were also associated with synapse organization, dendritic morphogenesis, GTPase activity regulation, and ion channels (Supplementary Figure S4). The GO-bp of DEGs within the SM groups showed fewer neurologically associated processes which include cerebral cortex development, and forebrain development (Supplementary Figure S4). Other processes observed in the DEGs were associated with the immune response to the effect of methamphetamine, which, though interesting, is beyond the scope of this current study and therefore excluded from our discussion. The majority of the GO-bp in the downregulated DEGs (Supplementary Figure S5) for all the groups are involved in structural elements of the cells such as microtubule formation, ciliary plasm, and extracellular matrix structural constituents.

### 3.5 Methamphetamine derived protein interaction network

Several differentially expressed genes were upregulated and significantly involved in neurological processes associated with methamphetamine exposure. To streamline the protein of interest, we adopted the approach of finding crucial genes based on whether they were hubs/bottlenecks within a protein interaction network. We constructed a methamphetamine-derived protein interaction network (MDPIN) from the upregulated DEGs using the STRING-database PPI with a strong confidence level (>800 combined scores) and obtained a network with 1932 interactions and 521 nodes (proteins). We calculated hubs and bottlenecks based on the number of degrees (interactors) and betweenness. This allowed us to filter the genes to select only those that are within the top 4, which were the following genes: ADCY1, Akt3, CAMK2a, and IGF1R (Figure 3D).

## 4 Discussion

Methamphetamine usage during and after pregnancy can significantly affect the development of the nervous system and behavior in the offspring (Smith et al., 2015a,b; Eze et al., 2016). In the present study, we utilized a mouse model to study behavioral changes and differentially expressed genes following pre- and post-natal methamphetamine methamphetamine exposure. We also examined body weight among pregnant mice exposed to methamphetamine, which were found to be comparable to those in the control group and did not affect the number of offspring per mouse when compared to the control group. These findings suggest that prenatal development and litter size of the pups remain largely unaffected by methamphetamine exposure, suggesting a limited impact on these parameters; though, there was a significant variance in the birth weight within the meth—treated compared to the control group.

In this study, we found that early-life methamphetamine exposure affected many behavioral domains in the mice offspring. On the open field test, the MM group showed significantly less distance traveled, similar to prior studies in mice that were exposed to other dopaminergic stimulants like cocaine and amphetamine (Schlussman et al., 1998; Chen et al., 2007). In the novel object recognition task, the MM and SM exhibited memory impairment, which was not observed in the Y-maze (spatial working memory) task between the control and meth-exposed groups. A caveat of the NOR in this study is that we opted for the minimum acceptable time (5 min), capitalizing on the high activity index of mice compared to rats in the open field, as utilized in our prior studies (Adeniyi et al., 2016a,c; Sultana et al., 2019, 2021). Although an extended monitoring period may reveal additional alterations, the numerous animals and multiple tests in our study provides a compensatory effect for the abbreviated monitoring time. The NOR results suggest methamphetamine exposure during both prenatal and postnatal periods may negatively and selectively impact only certain aspects of memory behavior in mice. The decline in NOR behavior, especially in the MM group, suggests an impact of methamphetamine on the mesocorticolimbic pathway, with methamphetamine exposure leading to impairment of recognition memory as a result of normal maturation of these regions, especially during adolescence (Joca et al., 2014; Itzhak et al., 2015; Buck et al., 2017; Groman et al., 2018; Yan et al., 2019; Dong et al., 2022). The complexity of the methamphetamine biochemical interaction on the behavioral deficits may be an interplay of several factors including pharmacological and genetically related impacts on the dopaminergic system (Bubenikova-Valesova et al., 2009; Groman et al., 2012; Buck and Siegel, 2015).

Our study also implicated altered gene expression through our RNA sequencing studies, which showed several differentially expressed genes between the treatment groups. Our KEGG pathway analysis revealed several pathways that were significantly expressed in the different treatment groups (Figure 4). The SM group showed upregulation in pathways associated with long-term depression (LTD), dopaminergic synapse, cholinergic synapse, and amphetamine addiction, though this was not significant. The MS group showed significant elevation in the PI3K-Akt signaling pathway, MARK signaling pathway, long-term potentiation (LTP), glutamatergic synapse, and cholinergic synapse pathway. The MM group showed an elevated level of genes associated with the neurotrophic signaling pathway, LTP, glutamatergic synapse, cholinergic synapse, CAMP signaling pathway, axon guidance, and amphetamine addiction-related pathway. These results suggest that methamphetamine exposure may alter several important signaling pathways in the brain, potentially leading to negative behavioral outcomes.

Methamphetamine exposure during pre- and post-natal periods can impact the development of the brain, potentially through altered gene expression (Itzhak et al., 2015; Tomášková et al., 2020). In our study, we utilized gene ontology biological processes that implicated several synaptic-related processes that are potentially altered in the MM group compared with the SS group (Figure 4). The PFC is involved in motivation and social and emotional related activity that exhibit learning-dependent plasticity (Li et al., 2019). Hence, synaptic loss and abnormality appear strongly correlated with the behavioral impairments observed in the MM group. Furthermore, several key genes were identified as being significantly upregulated in the methamphetamine-exposed animals, including ADCY1, CAMK2a, and AKT3 as identified through our constructed methamphetamine-derived protein interaction network (Figure 3D). These proteins putatively work together as part of a cAMP signaling network (Supplementary Figure S6). Among these proteins, ADCY1 in methamphetamine exposure has contrasting findings in several studies. For instance, dopamine stimulation of ADCY1 in the striatum might be due to drug withdrawal from methamphetamine (Tong et al., 2003), but other studies have reported the possible role of ADCY1 in memory/learning and long-term potentiation (Mons et al., 2003, 2004).

Although this study focused on the impact of prenatal and postnatal exposure to methamphetamine on prefrontal cortical genes, we note that other regions are likely also affected and interface with the PFC through the meso-corticolimbic pathway, such as the hippocampus, and ventral tegmental area (VTA), as well as others (Theyel et al., 2010; Itzhak et al., 2015; Lacy et al., 2016; Dong et al., 2018, 2022; Sanjari Moghaddam et al., 2021). Future work will focus on the interplay with these other regions to better understand the drivers of cognitive activities due to methamphetamine exposure.

Overall, the results of this study suggest that prenatal and postnatal methamphetamine exposure can lead to significant changes in gene expression patterns in mice and result in a significant behavioral change. These changes may be associated with alterations in important signaling pathways in the brain, potentially leading to negative outcomes. These findings have important implications for the potential risks associated with methamphetamine use during pregnancy and the potential impact on offspring behavior and gene expression. Further research could explore other downstream transcriptional factors such as the cAMP response element binding proteins as suggested from our proposed altered pathways in methamphetamine toxicity (Supplementary Figure S3).

## Data availability statement

The original contributions presented in the study are publicly available. This data can be found here: https://zenodo.org/records/10728651.

## Ethics statement

The animal study was approved by Institutional Animal Care and Use Committee (IACUC) of the Louisiana State University School of Veterinary Medicine. The study was conducted in accordance with the local legislation and institutional requirements.

## Author contributions

PA: Conceptualization, Data curation, Formal analysis, Investigation, Methodology, Project administration, Validation, Visualization, Writing – original draft, Writing – review & editing. TA: Data curation, Formal analysis, Investigation, Methodology, Project administration, Resources, Validation, Visualization, Writing – original draft, Writing – review & editing. AS: Investigation, Methodology, Writing – review & editing. C-CL: Formal Analysis, Writing - review & editing. CL: Conceptualization, Funding acquisition, Project administration, Supervision, Writing – original draft, Writing – review & editing.
