# Supplementary material for: Prenatal and postnatal methamphetamine exposure alters prefrontal cortical gene expression and behavior in mice
Source: Front Behav Neurosci. 2024 Mar 5;18:1286872. doi: 10.3389/fnbeh.2024.1286872 (PMC10949922; doi:10.3389/fnbeh.2024.1286872)

Supplementary Figure 1

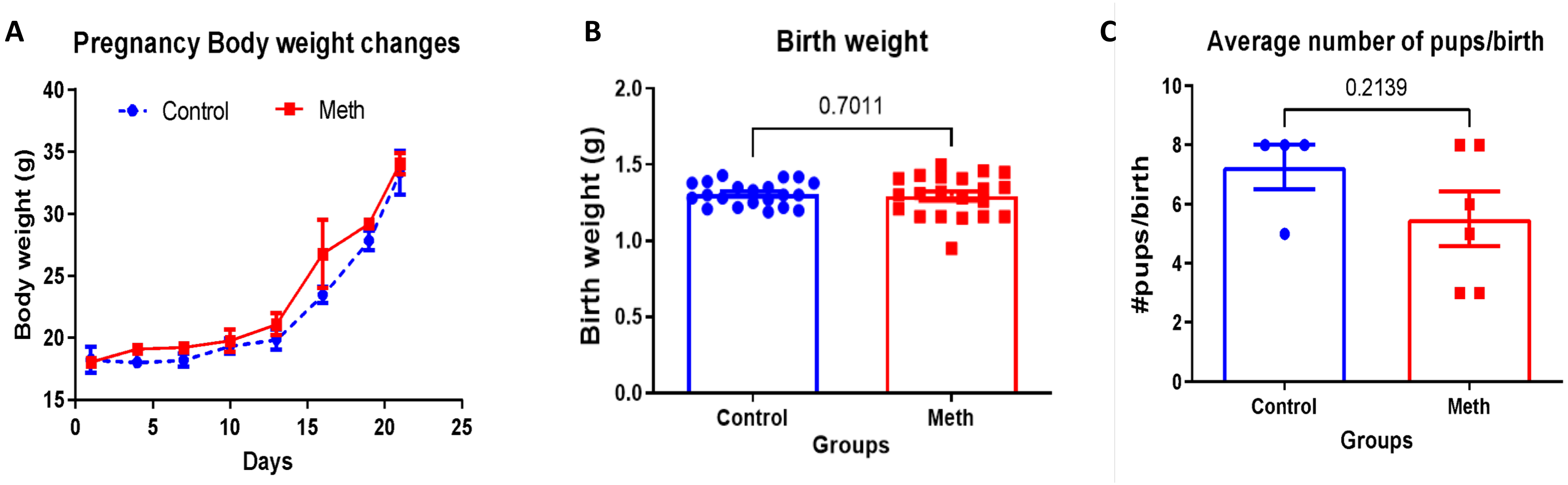

Figure 1: (A). Average pregnancy body weight from day 0 to parturition in Control and Meth exposure groups. (B). Birth weight of pubs (litters). (C). Average number of pubs (litters) per birth in Control and Meth exposure groups.

Supplementary Figure 2

KEGGs Pathways in Upregulated Differentially Expressed Genes

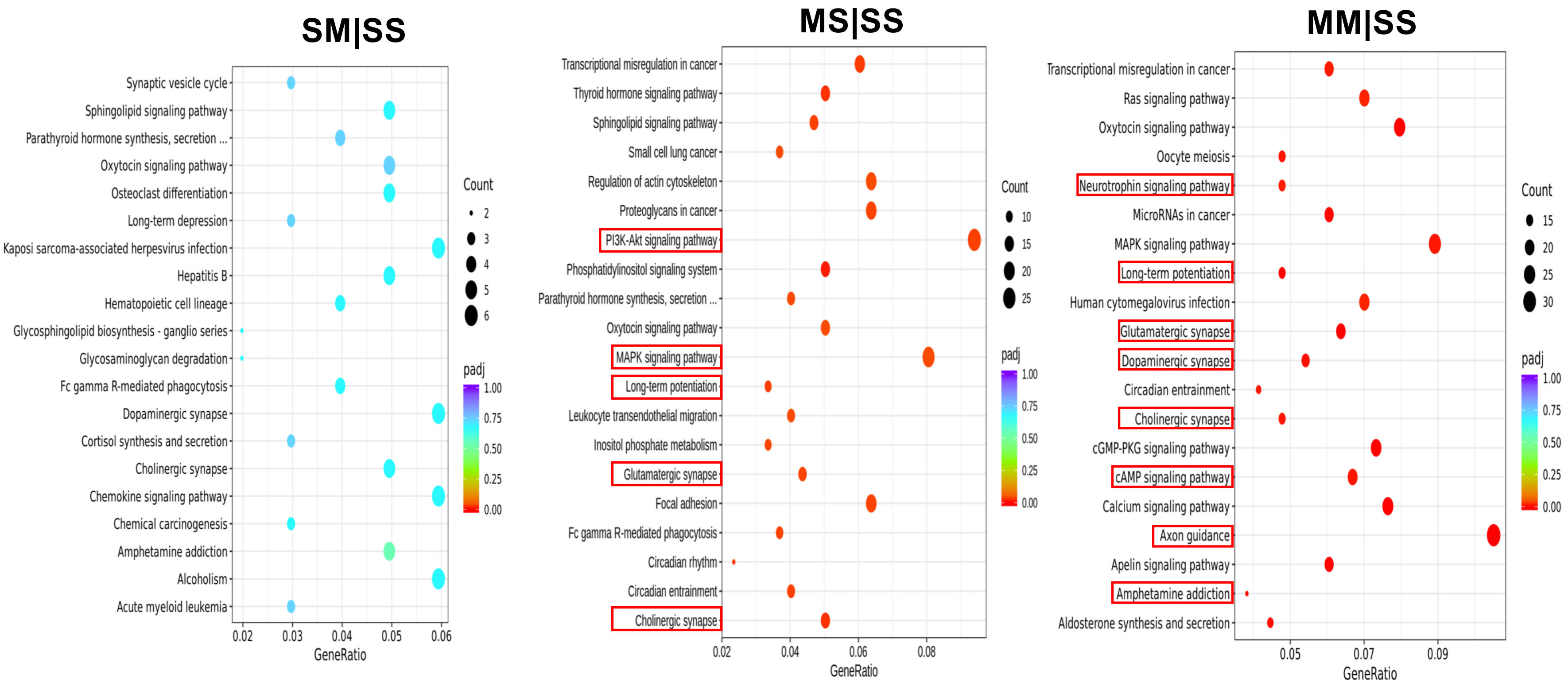

Supplementary Figure 3

KEGGs Pathways in Downregulated Differentially Expressed Genes

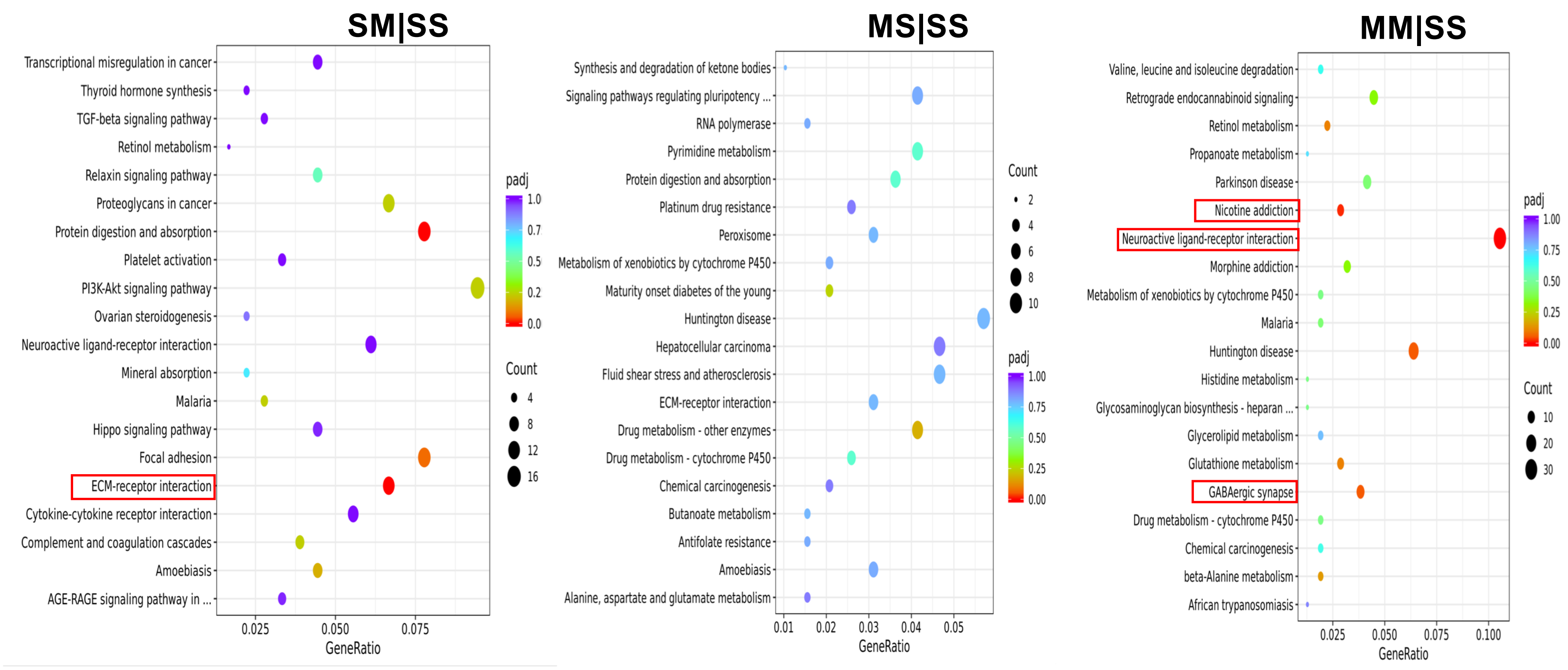

Supplementary Figure 4

Gene Ontology Biological Process in Upregulated Differentially Expressed Genes

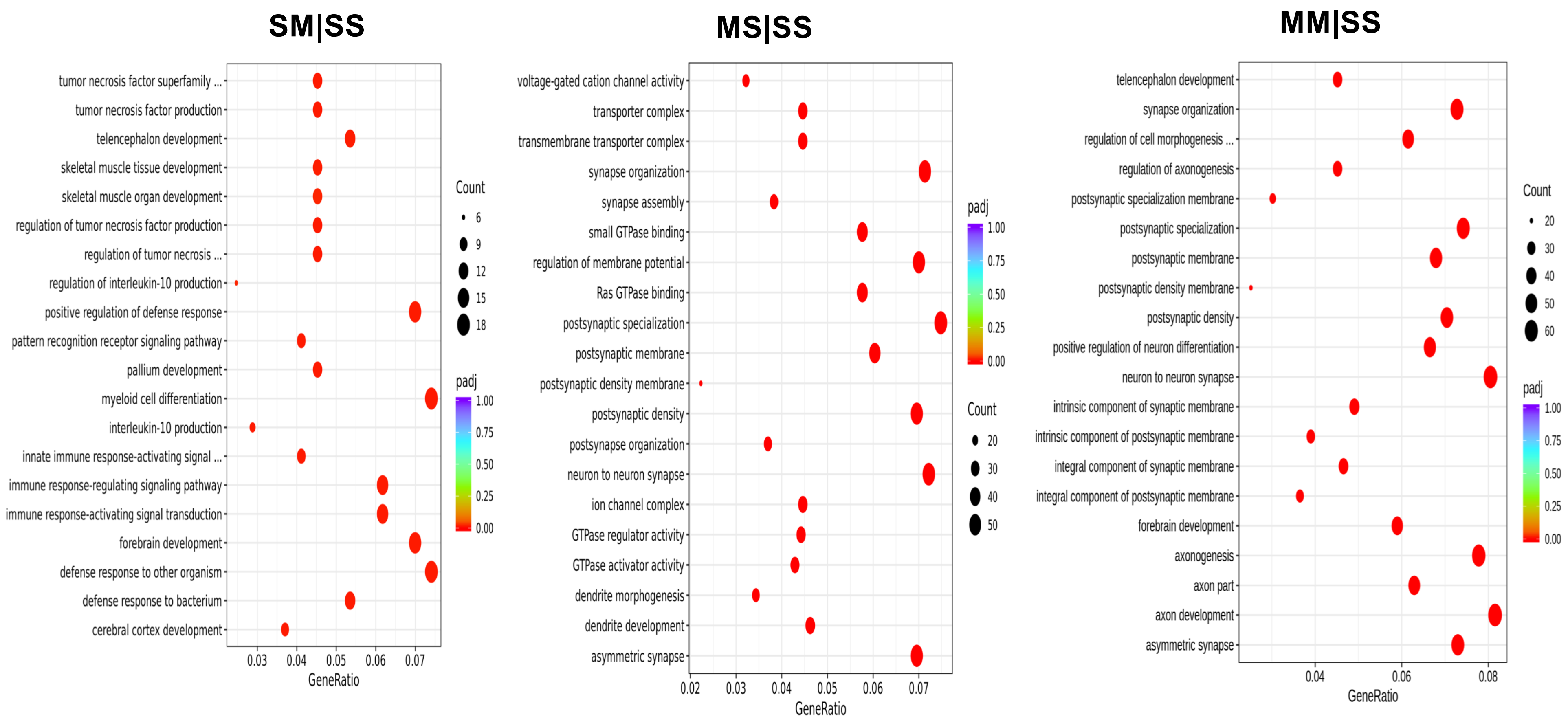

Supplementary Figure 5

Gene Ontology Biological Process in Downregulated Differentially Expressed Genes

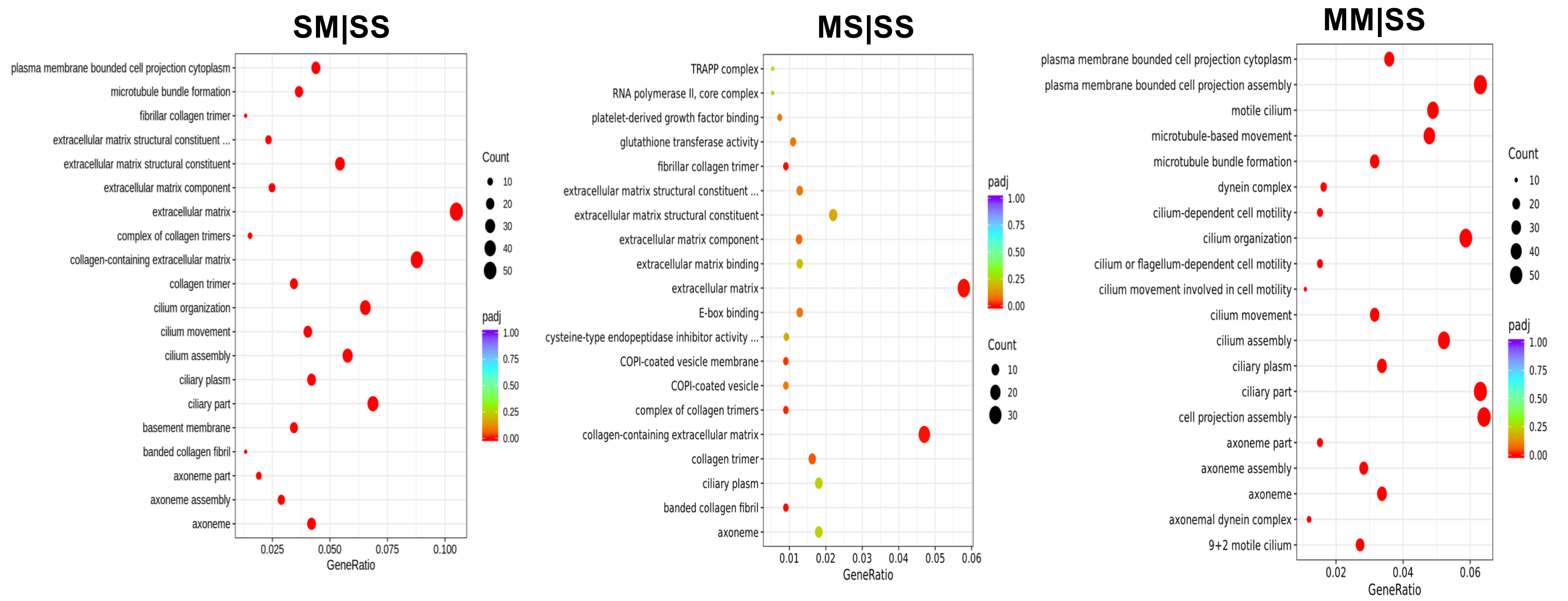

Supplementary Figure 6

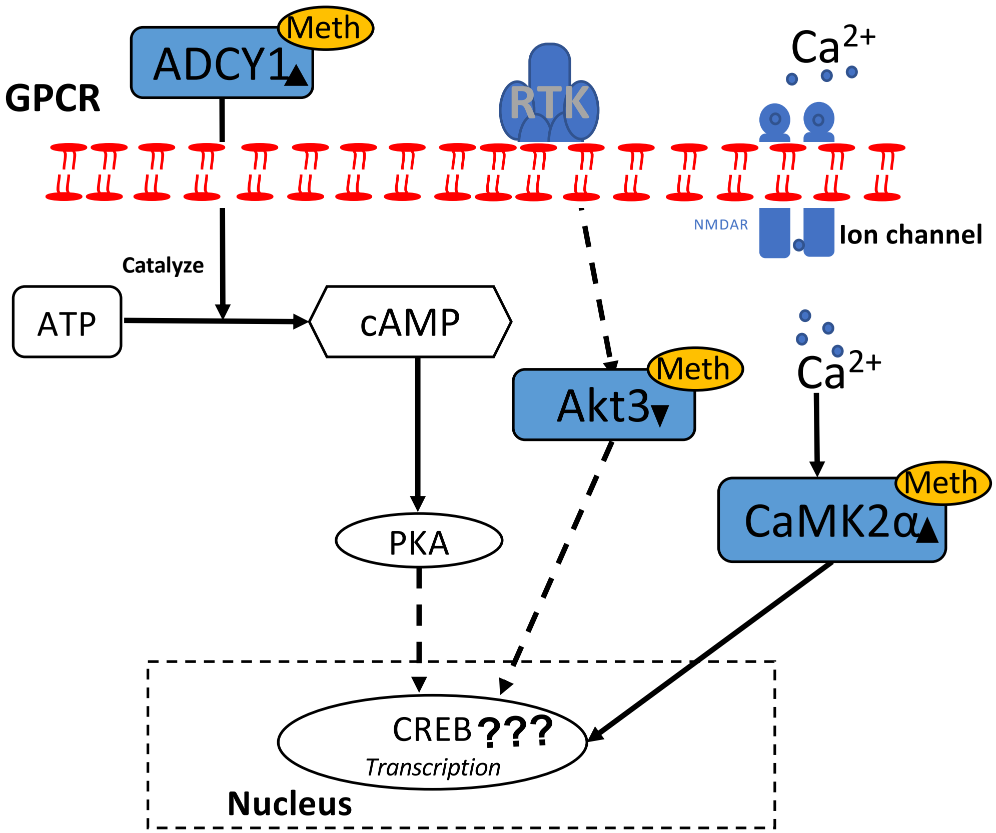

Supplement: Supplementary file 1 [file Data_Sheet_1.PDF]
