# Supplementary material for: Prenatal and postnatal methamphetamine exposure alters prefrontal cortical gene expression and behavior in mice
Source: Front Behav Neurosci. 2024 Mar 5;18:1286872. doi: 10.3389/fnbeh.2024.1286872 (PMC10949922; doi:10.3389/fnbeh.2024.1286872)
Supplement: Supplementary file 2 [file Data_Sheet_2.PDF]

**Supplementary Table S1 Differentially expressed genes in Prefrontal Cortex (PFC) of mice following METH exposure**

| Gene Symbol | Gene Description | MM group (RPKM) | SS group (RPKM) | log2FoldChange | pvalue | padj |
|---|---|---|---|---|---|---|
| Ccr6 | chemokine (C-C motif) receptor 6 | 0.109922801 | 0 | 4.929882925 | 0.001668 | 1 |
| Gm20716 | predicted gene 20716 | 0.032210878 | 0 | 4.335474417 | 0.047691 | 1 |
| Gm44633 | predicted gene 44633 | 0.385499415 | 0 | 4.327980834 | 0.025161 | 1 |
| Gm7237 | predicted gene 7237 | 0.118668593 | 0 | 4.309176193 | 0.019116 | 1 |
| Gm37124 | predicted gene, 37124 | 0.082858493 | 0 | 4.302355586 | 0.023355 | 1 |
| Uba1y | ubiquitin-activating enzyme, Chr Y | 0.059961092 | 0.002523629 | 4.275448202 | 0.00484 | 1 |
| Gm10604 | predicted gene 10604 | 0.126716337 | 0.005983633 | 4.103153913 | 0.026676 | 1 |
| Gm37106 | predicted gene, 37106 | 0.089367239 | 0 | 4.026426438 | 0.042718 | 1 |
| Skint3 | selection and upkeep of intraepithelial T cells | 0.048113315 | 0.002424288 | 3.8773911 | 0.026472 | 1 |
| Gm19963 | predicted gene, 19963 | 0.13164117 | 0.006902837 | 3.725653443 | 0.025723 | 1 |
| Gm44228 | predicted gene, 44228 | 0.218884511 | 0.017605675 | 3.624435576 | 0.001327 | 1 |
| E030026E1 | RIKEN cDNA E030026E10 gene | 0.068844242 | 0.00390756 | 3.604140889 | 0.04373 | 1 |
| Gm43099 | predicted gene 43099 | 0.028523677 | 0.001947735 | 3.583757438 | 0.036434 | 1 |
| Btn1a1 | butyrophilin, subfamily 1, member A1 | 0.035773411 | 0.002261845 | 3.569725721 | 0.049286 | 1 |
| Gm9111 | predicted gene 9111 | 0.27138483 | 0.024437406 | 3.375312452 | 0.037637 | 1 |
| Gm37529 | predicted gene, 37529 | 0.082050051 | 0.00870367 | 3.271771514 | 0.005888 | 1 |
| 4933425D2 | RIKEN cDNA 4933425D22 gene | 0.103246921 | 0.010813722 | 3.260791124 | 0.037749 | 1 |
| Gm45698 | predicted gene 45698 | 0.219392036 | 0.024029494 | 3.196043796 | 0.034661 | 1 |
| Klhl38 | kelch-like 38 | 0.103890183 | 0.012625206 | 3.046591818 | 0.034355 | 1 |
| Gm43322 | predicted gene 43322 | 0.263661692 | 0.033377843 | 2.96921621 | 0.022668 | 1 |
| Gm38340 | predicted gene, 38340 | 0.067262786 | 0.008808813 | 2.898577436 | 0.029983 | 1 |
| Gm48538 | predicted gene, 48538 | 0.279923975 | 0.040776359 | 2.745551271 | 0.04992 | 1 |
| Gm37452 | predicted gene, 37452 | 0.40619718 | 0.066762923 | 2.63786198 | 0.021624 | 1 |
| 1810019N2 | RIKEN cDNA 1810019N24 gene | 0.142574974 | 0.022536977 | 2.629409821 | 0.045368 | 1 |
| Gm48342 | predicted gene, 48342 | 0.11677511 | 0.018798567 | 2.618006487 | 0.046274 | 1 |
| Gm46210 | predicted gene, 46210 | 0.536984492 | 0.096159131 | 2.487758819 | 0.039336 | 1 |
| Tmem181c | transmembrane protein 181C, pseudogene | 0.399977657 | 0.07713662 | 2.352408578 | 0.002971 | 1 |
| Gm37735 | predicted gene, 37735 | 0.258813442 | 0.051693636 | 2.308892981 | 0.036538 | 1 |
| Gm15894 | predicted gene 15894 | 0.14464692 | 0.029580818 | 2.257223634 | 0.04231 | 1 |
| Gm14776 | predicted gene 14776 | 1.192709407 | 0.254902009 | 2.21936611 | 0.000833 | 0.065202 |
| Gm44241 | predicted gene, 44241 | 0.320742535 | 0.069990273 | 2.176311718 | 0.014925 | 1 |
| Gm14662 | predicted gene 14662 | 0.763446496 | 0.16851728 | 2.16704158 | 0.017091 | 0.315784 |
| Gm37285 | predicted gene, 37285 | 0.437805441 | 0.10105796 | 2.08294449 | 0.023868 | 1 |
| Gm42772 | predicted gene 42772 | 0.464475999 | 0.111785607 | 2.044741014 | 0.000239 | 0.02983 |
| Gm3364 | predicted gene 3364 | 0.332343173 | 0.080100309 | 2.030803029 | 0.024424 | 1 |

| | | | | | | |
|---|---|---|---|---|---|---|
| Gm16551 | predicted gene 16551 | 0.087968544 | 0.021511111 | 2.01600225 | 0.043088 | 1 |
| Pcp2 | Purkinje cell protein 2 (L7) | 1.164085127 | 0.290696345 | 1.977208585 | 0.007404 | 0.218248 |
| Fhl5 | four and a half LIM domains 5 | 0.393908759 | 0.100840304 | 1.957964228 | 0.022704 | 1 |
| Gm47623 | predicted gene, 47623 | 1.57373121 | 0.413945957 | 1.885825631 | 0.046449 | 1 |
| Gm12532 | predicted gene 12532 | 0.132540555 | 0.036214744 | 1.853318387 | 0.045998 | 1 |
| Gm42798 | predicted gene 42798 | 0.097108049 | 0.027009428 | 1.835453204 | 0.041447 | 1 |
| Gm43355 | predicted gene 43355 | 1.036608508 | 0.292131813 | 1.8327581 | 0.035952 | 1 |
| Gm26736 | predicted gene, 26736 | 0.557512848 | 0.16482435 | 1.764844047 | 0.008094 | 0.225748 |
| Gm8738 | predicted gene 8738 | 2.819149608 | 0.827066191 | 1.763882155 | 0.001075 | 0.0767 |
| Gm42970 | predicted gene 42970 | 1.319517251 | 0.386149882 | 1.757711198 | 0.001139 | 0.078396 |
| Gm44799 | predicted gene 44799 | 1.758706778 | 0.534948271 | 1.713095316 | 0.000703 | 0.059309 |
| Tex13c2 | TEX13 family member C2 | 0.855598467 | 0.285149989 | 1.564984887 | 0.000851 | 0.066047 |
| Gm11839 | predicted gene 11839 | 0.417020615 | 0.143526418 | 1.519150113 | 0.037331 | 0.429807 |
| Mpl | myeloproliferative leukemia virus oncogene | 0.208231266 | 0.072602308 | 1.5170499 | 0.029168 | 0.389312 |
| G530011O | RIKEN cDNA G530011O06 gene | 0.658213776 | 0.234534784 | 1.494438363 | 0.005057 | 0.178989 |
| Gm42600 | predicted gene 42600 | 0.251443898 | 0.088783407 | 1.474872649 | 0.004315 | 0.165914 |
| Gm5881 | predicted gene 5881 | 2.265870167 | 0.814796318 | 1.452747806 | 0.010808 | 0.259903 |
| Gm26801 | predicted gene, 26801 | 0.39177435 | 0.143559182 | 1.429692326 | 0.01907 | 0.328038 |
| Cd163l1 | CD163 molecule-like 1 | 0.154801235 | 0.058938454 | 1.390463151 | 0.049636 | 0.479319 |
| 9430081H | RIKEN cDNA 9430081H08 gene | 0.997909965 | 0.381874926 | 1.361580684 | 0.013681 | 0.288529 |
| Gm14033 | predicted gene 14033 | 0.377278652 | 0.145768217 | 1.354764653 | 0.04843 | 0.475326 |
| Gm19445 | predicted gene, 19445 | 0.292086929 | 0.117781502 | 1.315724283 | 0.038877 | 0.436769 |
| Gm45643 | predicted gene 45643 | 0.760174184 | 0.315214722 | 1.269620523 | 0.030953 | 0.40001 |
| Ncapg | non-SMC condensin I complex, subunit G | 0.174315109 | 0.073282353 | 1.239145587 | 0.005969 | 0.19607 |
| Arhgef33 | Rho guanine nucleotide exchange factor (GEF | 0.623072759 | 0.26080674 | 1.230330962 | 0.011256 | 0.264259 |
| AC154486. | C2H2 zinc finger protein pseudogene | 0.667415893 | 0.30211093 | 1.128500109 | 0.033097 | 0.411041 |
| CT010433. | C2H2 zinc finger protein pseudogene | 0.842963497 | 0.387962855 | 1.11627279 | 0.014 | 0.289976 |
| Tcf24 | transcription factor 24 | 0.205073553 | 0.094293841 | 1.111629423 | 0.036578 | 0.426227 |
| Gm9761 | predicted gene 9761 | 1.485691263 | 0.687108528 | 1.111295585 | 0.039166 | 0.438331 |
| Nsl1 | NSL1, MIS12 kinetochore complex compone | 0.411733579 | 0.190329699 | 1.106732701 | 0.004176 | 0.163555 |
| 3300002I0 | RIKEN cDNA 3300002I08 gene | 0.997104023 | 0.460422536 | 1.094233406 | 0.002523 | 0.12422 |
| Iyd | iodotyrosine deiodinase | 6.755405113 | 3.151902407 | 1.085005751 | 5.58E-05 | 0.012729 |
| Cysltr2 | cysteinyl leukotriene receptor 2 | 0.299495451 | 0.138871109 | 1.083283845 | 0.037999 | 0.432908 |
| Gm37824 | predicted gene, 37824 | 0.580414666 | 0.271856776 | 1.074906812 | 0.016618 | 0.31404 |
| Gng10 | guanine nucleotide binding protein (G protei | 0.994716246 | 0.471909726 | 1.06730177 | 0.030372 | 0.396303 |
| Gm36823 | predicted gene, 36823 | 0.355585446 | 0.169462861 | 1.058438204 | 0.043561 | 0.453419 |
| Gm5083 | predicted gene 5083 | 1.076022012 | 0.519148871 | 1.041330149 | 0.028842 | 0.387794 |
| 2900022M | RIKEN cDNA 2900022M07 gene | 1.131488712 | 0.554939417 | 1.022358483 | 0.031153 | 0.401125 |
| Dsp | desmoplakin | 5.777675732 | 2.837723446 | 1.013180279 | 0.010444 | 0.256495 |

| | | | | | | |
|---|---|---|---|---|---|---|
| Il20rb | interleukin 20 receptor beta | 1.039051577 | 0.519637144 | 0.986171313 | 0.000376 | 0.040122 |
| Gm35037 | predicted gene, 35037 | 2.498890812 | 1.246887763 | 0.985411168 | 0.027613 | 0.38026 |
| Kif18a | kinesin family member 18A | 0.279801325 | 0.140270626 | 0.982888695 | 0.002256 | 0.119646 |
| Pappa2 | pappalysin 2 | 0.389725627 | 0.195814533 | 0.979306975 | 0.001106 | 0.078012 |
| Mid1 | midline 1 | 0.591283608 | 0.298382385 | 0.973819323 | 0.001041 | 0.075232 |
| St6gal2 | beta galactoside alpha 2,6 sialyltransferase 2 | 4.688741097 | 2.379921384 | 0.967254488 | 0.021844 | 0.349401 |
| Ltbp2 | latent transforming growth factor beta bindi | 0.201554459 | 0.102944005 | 0.958698849 | 0.014115 | 0.290324 |
| Ptx3 | pentraxin related gene | 1.477676689 | 0.755735903 | 0.951317973 | 0.001485 | 0.092854 |
| Lct | lactase | 8.168361828 | 4.225836637 | 0.93890448 | 0.005009 | 0.17802 |
| Ghsr | growth hormone secretagogue receptor | 0.561897765 | 0.290318678 | 0.93037055 | 0.015774 | 0.307705 |
| C630031E1 | RIKEN cDNA C630031E19 gene | 0.736724507 | 0.382344881 | 0.930025312 | 0.045851 | 0.465295 |
| Cep55 | centrosomal protein 55 | 0.4182656 | 0.219304574 | 0.91025833 | 0.044632 | 0.459003 |
| Slco5a1 | solute carrier organic anion transporter fami | 0.313788259 | 0.165190822 | 0.907601121 | 0.017068 | 0.315784 |
| Gm42853 | predicted gene 42853 | 1.216537681 | 0.645137356 | 0.897852045 | 0.022934 | 0.357433 |
| AC165271. | novel transcript, antisense to Kcnj6 | 2.95857825 | 1.568201592 | 0.895690601 | 0.005737 | 0.19214 |
| Ackr2 | atypical chemokine receptor 2 | 0.526175826 | 0.281212138 | 0.889506677 | 0.016901 | 0.315592 |
| Snora57 | small nucleolar RNA, H/ACA box 57 | 8.464852028 | 4.542903378 | 0.886373248 | 0.04176 | 0.449122 |
| Npas4 | neuronal PAS domain protein 4 | 2.556659655 | 1.370972174 | 0.88583496 | 5.19E-05 | 0.01236 |
| Hpdl | 4-hydroxyphenylpyruvate dioxygenase-like | 3.753639801 | 2.025651156 | 0.874514052 | 0.000749 | 0.062264 |
| Dmrta1 | doublesex and mab-3 related transcription fa | 0.602458812 | 0.328823076 | 0.851596874 | 0.009136 | 0.237861 |
| Dbh | dopamine beta hydroxylase | 0.483766211 | 0.266815855 | 0.838287968 | 0.04737 | 0.47273 |
| Mex3a | mex3 RNA binding family member A | 2.427142003 | 1.345485842 | 0.836114507 | 2.36E-06 | 0.001191 |
| Gm42636 | predicted gene 42636 | 0.587100345 | 0.328342745 | 0.825577924 | 0.038333 | 0.434618 |
| Ube2cbp | ubiquitin-conjugating enzyme E2C binding p | 1.058023211 | 0.591198328 | 0.822378715 | 0.035963 | 0.424092 |
| Mylk3 | myosin light chain kinase 3 | 0.622121392 | 0.347565459 | 0.82075263 | 0.02222 | 0.352216 |
| Gm42492 | predicted gene 42492 | 0.472392119 | 0.265842037 | 0.810793427 | 0.033625 | 0.411285 |
| Pclaf | PCNA clamp associated factor | 0.756864507 | 0.43347027 | 0.790959906 | 0.00377 | 0.154715 |
| Nhlh1 | nescient helix loop helix 1 | 1.07606622 | 0.619833767 | 0.786505397 | 0.038217 | 0.434338 |
| Fosb | FBJ osteosarcoma oncogene B | 0.971760454 | 0.557853505 | 0.781127704 | 0.018258 | 0.323776 |
| Rtl3 | retrotransposon Gag like 3 | 1.180706354 | 0.685340459 | 0.774669987 | 0.002629 | 0.127172 |
| Prox1 | prospero homeobox 1 | 22.01665058 | 12.77096994 | 0.772312455 | 2.00E-05 | 0.006406 |
| Gal | galanin | 2.826749838 | 1.637794128 | 0.764962149 | 0.046421 | 0.469189 |
| Pcdha5 | protocadherin alpha 5 | 0.643846155 | 0.373362793 | 0.764497215 | 0.019316 | 0.330205 |
| Cyp26b1 | cytochrome P450, family 26, subfamily b, pc | 4.121446189 | 2.406676711 | 0.761533291 | 0.000202 | 0.026964 |
| Pcdhgb1 | protocadherin gamma subfamily B, 1 | 1.090000804 | 0.643733111 | 0.744124792 | 0.000703 | 0.059309 |
| Igsf6 | immunoglobulin superfamily, member 6 | 0.737882115 | 0.439945262 | 0.739015331 | 0.042061 | 0.449674 |
| Gm17322 | predicted gene, 17322 | 4.744635911 | 2.823187541 | 0.731627648 | 5.04E-05 | 0.012331 |
| Fstl4 | follistatin-like 4 | 2.166323916 | 1.302298643 | 0.714840232 | 0.000435 | 0.043554 |
| Draxin | dorsal inhibitory axon guidance protein | 1.437590126 | 0.867294895 | 0.714583846 | 0.012773 | 0.281232 |

| | | | | | | |
|---|---|---|---|---|---|---|
| Nhlh2 | nescient helix loop helix 2 | 1.091350444 | 0.659595113 | 0.71239826 | 0.006607 | 0.206058 |
| Syt10 | synaptotagmin X | 2.002944466 | 1.210241512 | 0.710755383 | 0.00522 | 0.181853 |
| Bhlhe22 | basic helix-loop-helix family, member e22 | 63.23580337 | 38.38801734 | 0.706981047 | 7.41E-05 | 0.015003 |
| Sema3c | sema domain, immunoglobulin domain (Ig), | 10.17960453 | 6.187089715 | 0.704513319 | 9.09E-05 | 0.016866 |
| Chrna5 | cholinergic receptor, nicotinic, alpha polype | 1.644145765 | 1.002799735 | 0.698680633 | 0.007387 | 0.218248 |
| Sema5a | sema domain, seven thrombospondin repeat | 10.41378506 | 6.39862102 | 0.688170603 | 1.88E-05 | 0.006267 |
| Dgki | diacylglycerol kinase, iota | 2.794400892 | 1.72010105 | 0.682305947 | 0.000413 | 0.042141 |
| Epha7 | Eph receptor A7 | 19.14200708 | 11.86993817 | 0.677237028 | 0.000299 | 0.035408 |
| Rimbp3 | RIMS binding protein 3 | 0.484243032 | 0.299991079 | 0.671788497 | 0.033972 | 0.411285 |
| Thumpd2 | THUMP domain containing 2 | 2.872687738 | 1.790187259 | 0.669298912 | 0.002297 | 0.11979 |
| Fam111a | family with sequence similarity 111, member | 0.843487682 | 0.524894361 | 0.668705221 | 0.00878 | 0.234897 |
| Neurod1 | neurogenic differentiation 1 | 17.54416634 | 10.94661529 | 0.667567355 | 0.001332 | 0.087213 |
| Ccbe1 | collagen and calcium binding EGF domains 1 | 2.82509598 | 1.77185776 | 0.656784795 | 3.98E-05 | 0.010568 |
| Dclk3 | doublecortin-like kinase 3 | 5.567203605 | 3.503063364 | 0.654320773 | 0.000358 | 0.03939 |
| Jph1 | junctophilin 1 | 9.353820084 | 5.896122788 | 0.652790047 | 5.96E-05 | 0.012806 |
| Ap1s3 | adaptor-related protein complex AP-1, sigma | 0.764106546 | 0.482326971 | 0.650919619 | 0.029516 | 0.390897 |
| Klk8 | kallikrein related-peptidase 8 | 10.43597588 | 6.602018993 | 0.649823481 | 0.001596 | 0.097347 |
| 3110001I2 | RIKEN cDNA 3110001I22 gene | 1.158936409 | 0.733189171 | 0.646759875 | 0.033268 | 0.411225 |
| AI115009 | expressed sequence AI115009 | 2.287790652 | 1.449173073 | 0.643027702 | 0.01253 | 0.277167 |
| Fam163b | family with sequence similarity 163, member | 75.64974556 | 48.06075042 | 0.641955442 | 3.93E-05 | 0.010568 |
| Ckap2l | cytoskeleton associated protein 2-like | 0.812477249 | 0.519068073 | 0.634944071 | 0.042316 | 0.449801 |
| Dcx | doublecortin | 4.746302216 | 3.028943868 | 0.63333962 | 9.00E-05 | 0.016866 |
| Thbs4 | thrombospondin 4 | 5.817576924 | 3.727080484 | 0.625670939 | 0.000235 | 0.029635 |
| Sox11 | SRY (sex determining region Y)-box 11 | 7.266037639 | 4.678018734 | 0.621303055 | 0.000423 | 0.042821 |
| Mkx | mohawk homeobox | 2.59450308 | 1.669903991 | 0.621075657 | 0.000118 | 0.019405 |
| Sypl2 | synaptophysin-like 2 | 1.718729703 | 1.104492846 | 0.620536554 | 0.002169 | 0.118362 |
| Pcdhb4 | protocadherin beta 4 | 0.820342501 | 0.529129781 | 0.620227843 | 0.034735 | 0.414579 |
| AC151284. | microtubule-associated protein 7 (Map7) pse | 7.452438123 | 4.823063421 | 0.615515566 | 0.012236 | 0.275097 |
| Kcnj6 | potassium inwardly-rectifying channel, subfa | 2.470285605 | 1.593236221 | 0.615150646 | 9.94E-05 | 0.017496 |
| Dgkh | diacylglycerol kinase, eta | 8.624412841 | 5.578673195 | 0.614685831 | 0.000587 | 0.052503 |
| Tbr1 | T-box brain gene 1 | 10.7098374 | 6.921466218 | 0.614049179 | 2.34E-06 | 0.001191 |
| Clspn | claspin | 0.454315343 | 0.295779639 | 0.607262584 | 0.034598 | 0.414579 |
| Igfbpl1 | insulin-like growth factor binding protein-lik | 11.51906724 | 7.501393989 | 0.605821091 | 0.002295 | 0.11979 |
| Trim30a | tripartite motif-containing 30A | 1.060117643 | 0.689326075 | 0.603706305 | 0.03671 | 0.426602 |
| Epha4 | Eph receptor A4 | 42.66363566 | 27.81825797 | 0.603171001 | 6.22E-05 | 0.01305 |
| AC171111. | novel transcript | 1.560595189 | 1.016632671 | 0.601943287 | 0.011283 | 0.264259 |
| Zfp960 | zinc finger protein 960 | 0.897293607 | 0.589050979 | 0.59606976 | 0.034655 | 0.414579 |
| Dtl | denticleless E3 ubiquitin protein ligase | 0.243323435 | 0.160431797 | 0.588361256 | 0.015791 | 0.307705 |
| Ly75 | lymphocyte antigen 75 | 0.933820596 | 0.614341318 | 0.587437192 | 0.011374 | 0.264852 |

| | | | | | | |
|---|---|---|---|---|---|---|
| Slc26a10 | solute carrier family 26, member 10 | 3.393453488 | 2.243188926 | 0.586742003 | 0.029156 | 0.389312 |
| Egr1 | early growth response 1 | 29.25598368 | 19.29349862 | 0.585321795 | 0.000184 | 0.025482 |
| Kcnj2 | potassium inwardly-rectifying channel, subfa | 2.241229326 | 1.48507468 | 0.580589404 | 0.009015 | 0.236533 |
| Gm4202 | predicted gene 4202 | 7.926684616 | 5.260791325 | 0.580159699 | 0.033602 | 0.411285 |
| Ntf3 | neurotrophin 3 | 6.192873513 | 4.114590287 | 0.579136373 | 0.020023 | 0.334904 |
| Gm15624 | predicted gene 15624 | 0.703140088 | 0.468348539 | 0.575655192 | 0.029855 | 0.3931 |
| Card6 | caspase recruitment domain family, member | 0.753447574 | 0.500318033 | 0.574808989 | 0.00635 | 0.202147 |
| Aldh1l2 | aldehyde dehydrogenase 1 family, member L | 0.870987104 | 0.579384586 | 0.573102907 | 0.009475 | 0.243219 |
| B3gnt5 | UDP-GlcNAc:betaGal beta-1,3-N-acetylgluco | 0.62103954 | 0.412749683 | 0.57043569 | 0.023432 | 0.360229 |
| Inhba | inhibin beta-A | 1.143696989 | 0.763441696 | 0.567212659 | 0.006198 | 0.200106 |
| Olfml2b | olfactomedin-like 2B | 13.5024902 | 9.030594359 | 0.565884759 | 0.004784 | 0.173499 |
| Abcc9 | ATP-binding cassette, sub-family C (CFTR/MR | 1.7647554 | 1.177126895 | 0.565861554 | 0.004396 | 0.165947 |
| Uhrf1 | ubiquitin-like, containing PHD and RING fing | 1.042515028 | 0.699796108 | 0.557903277 | 0.025945 | 0.372836 |
| Sertad4 | SERTA domain containing 4 | 8.900246077 | 5.99226357 | 0.557722307 | 0.000822 | 0.064659 |
| Ano3 | anoctamin 3 | 12.70893511 | 8.549930214 | 0.557608404 | 0.000176 | 0.024665 |
| Plxna4 | plexin A4 | 13.39323045 | 9.008845243 | 0.557219612 | 0.000161 | 0.024035 |
| Pcdhga2 | protocadherin gamma subfamily A, 2 | 0.981059551 | 0.657828853 | 0.556390124 | 0.010654 | 0.25868 |
| Fgf5 | fibroblast growth factor 5 | 1.809561317 | 1.21614617 | 0.555637428 | 0.024383 | 0.363714 |
| Ifi203 | interferon activated gene 203 | 0.898226122 | 0.605706867 | 0.555203797 | 0.004043 | 0.16123 |
| Lrrtm4 | leucine rich repeat transmembrane neuronal | 5.129700958 | 3.46536741 | 0.552859042 | 5.13E-05 | 0.01236 |
| Maml2 | mastermind like transcriptional coactivator | 4.196099252 | 2.830880251 | 0.552322686 | 1.78E-05 | 0.00604 |
| C1ql3 | C1q-like 3 | 21.72150294 | 14.72325055 | 0.547730288 | 0.000196 | 0.026393 |
| Fat4 | FAT atypical cadherin 4 | 1.835611498 | 1.244076528 | 0.54555084 | 0.000773 | 0.063082 |
| Wnt2 | wingless-type MMTV integration site family, | 2.291696024 | 1.558704013 | 0.54513556 | 0.031766 | 0.404338 |
| Trhde | TRH-degrading enzyme | 5.413141401 | 3.675583778 | 0.543163012 | 0.000349 | 0.03892 |
| Trpc5 | transient receptor potential cation channel, | 2.375333232 | 1.612133872 | 0.539264791 | 0.036882 | 0.427431 |
| Tmem114 | transmembrane protein 114 | 6.688310584 | 4.565033584 | 0.53695118 | 0.018706 | 0.327107 |
| Dmrta2 | doublesex and mab-3 related transcription fa | 3.164778878 | 2.164141011 | 0.534053925 | 0.016767 | 0.315592 |
| Cadm2 | cell adhesion molecule 2 | 32.79448798 | 22.42987834 | 0.533993096 | 1.10E-05 | 0.004358 |
| Epha6 | Eph receptor A6 | 5.988288328 | 4.096963987 | 0.532420987 | 0.00048 | 0.046785 |
| Mndal | myeloid nuclear differentiation antigen like | 2.068602178 | 1.419842864 | 0.531490906 | 0.009751 | 0.245904 |
| Cd46 | CD46 antigen, complement regulatory prote | 0.498984993 | 0.342250415 | 0.531211946 | 0.045321 | 0.462098 |
| Lyst | lysosomal trafficking regulator | 4.502343315 | 3.083452654 | 0.530893987 | 0.000176 | 0.024665 |
| Plekha2 | pleckstrin homology domain-containing, fam | 6.513205385 | 4.469749815 | 0.527973898 | 0.000362 | 0.03939 |
| Sphkap | SPHK1 interactor, AKAP domain containing | 29.31868682 | 20.14873625 | 0.527302349 | 0.000218 | 0.028228 |
| Pcdhb18 | protocadherin beta 18 | 1.721824751 | 1.183347978 | 0.525487145 | 0.012708 | 0.28035 |
| Spata13 | spermatogenesis associated 13 | 4.364303375 | 2.9994738 | 0.524838954 | 0.001121 | 0.078396 |
| Zbtb20 | zinc finger and BTB domain containing 20 | 7.836387082 | 5.397814322 | 0.522544267 | 0.000469 | 0.046411 |
| Cecr2 | CECR2, histone acetyl-lysine reader | 0.698653408 | 0.481572476 | 0.521749273 | 0.011283 | 0.264259 |

| | | | | | | |
|---|---|---|---|---|---|---|
| Acot3 | acyl-CoA thioesterase 3 | 1.13419074 | 0.780699004 | 0.520886071 | 0.028898 | 0.388256 |
| Zbtb18 | zinc finger and BTB domain containing 18 | 101.7124037 | 70.25330855 | 0.520855362 | 0.000365 | 0.039456 |
| Rfx3 | regulatory factor X, 3 (influences HLA class II ( | 10.7010637 | 7.396307012 | 0.519178047 | 0.000763 | 0.062908 |
| Asxl3 | additional sex combs like 3, transcriptional r | 0.749455938 | 0.517373376 | 0.518965174 | 0.011957 | 0.271962 |
| Zfp882 | zinc finger protein 882 | 2.324180662 | 1.603707854 | 0.518380274 | 0.00404 | 0.16123 |
| Bcl11b | B cell leukemia/lymphoma 11B | 19.55585697 | 13.55410138 | 0.515755018 | 0.001584 | 0.097312 |
| Gm42864 | predicted gene 42864 | 2.721521263 | 1.888197841 | 0.514341983 | 0.029427 | 0.390595 |
| Tpx2 | TPX2, microtubule-associated | 0.964559761 | 0.671400262 | 0.512867814 | 0.02462 | 0.365666 |
| Kctd4 | potassium channel tetramerisation domain c | 23.19734313 | 16.11118004 | 0.512778111 | 0.0013 | 0.086483 |
| Stc2 | stanniocalcin 2 | 1.546795879 | 1.073498016 | 0.510617821 | 0.0286 | 0.38601 |
| Gpr63 | G protein-coupled receptor 63 | 2.296246457 | 1.590719155 | 0.509703304 | 0.028035 | 0.382242 |
| Il16 | interleukin 16 | 4.116340722 | 2.860068593 | 0.509349018 | 0.001755 | 0.103969 |
| Pcdhgb6 | protocadherin gamma subfamily B, 6 | 2.238391623 | 1.550888298 | 0.509329983 | 0.04989 | 0.479765 |
| Arhgap4 | Rho GTPase activating protein 4 | 0.490533369 | 0.341602875 | 0.508976583 | 0.02034 | 0.33697 |
| Ttc28 | tetratricopeptide repeat domain 28 | 3.267703858 | 2.272031431 | 0.507293981 | 0.005714 | 0.191845 |
| Adcy1 | adenylate cyclase 1 | 98.93536698 | 68.83189946 | 0.506977902 | 0.000145 | 0.022492 |
| Mkl2 | MKL/myocardin-like 2 | 20.06087412 | 13.99288798 | 0.505613114 | 0.000368 | 0.039532 |
| Fam84b | family with sequence similarity 84, member I | 4.313389076 | 3.01099573 | 0.505074839 | 0.038018 | 0.432908 |
| Acan | aggrecan | 0.89518332 | 0.624657445 | 0.503368886 | 0.007391 | 0.218248 |
| Grm2 | glutamate receptor, metabotropic 2 | 17.24020381 | 12.07973877 | 0.498980331 | 0.000188 | 0.025857 |
| Ikzf1 | IKAROS family zinc finger 1 | 1.189752839 | 0.833932438 | 0.496321263 | 0.015816 | 0.307705 |
| Ephb2 | Eph receptor B2 | 3.288550358 | 2.313650406 | 0.492249528 | 0.009736 | 0.245858 |
| Islr2 | immunoglobulin superfamily containing leu | 39.36538508 | 27.72898874 | 0.491406307 | 0.004193 | 0.163555 |
| Skil | SKI-like | 10.01098787 | 7.058937566 | 0.489309868 | 6.10E-05 | 0.012955 |
| Stxbp6 | syntaxin binding protein 6 (amisyn) | 22.98944822 | 16.24369371 | 0.486802304 | 7.00E-06 | 0.003008 |
| Stxbp5l | syntaxin binding protein 5-like | 2.356146382 | 1.661098969 | 0.485662102 | 0.046301 | 0.468784 |
| Enc1 | ectodermal-neural cortex 1 | 171.162234 | 121.1840594 | 0.484807039 | 0.002482 | 0.123592 |
| A330008L1 | RIKEN cDNA A330008L17 gene | 1.272482936 | 0.901934058 | 0.484757781 | 0.013678 | 0.288529 |
| Lrrc55 | leucine rich repeat containing 55 | 4.078401662 | 2.885559273 | 0.484263455 | 0.008031 | 0.225748 |
| Iqgap2 | IQ motif containing GTPase activating protei | 11.6711141 | 8.271655321 | 0.483641119 | 0.013616 | 0.288529 |
| Slc8a2 | solute carrier family 8 (sodium/calcium exch | 77.33990137 | 54.87337507 | 0.481194984 | 0.001561 | 0.097199 |
| Prc1 | protein regulator of cytokinesis 1 | 0.4972873 | 0.354044968 | 0.480078645 | 0.045893 | 0.465449 |
| Ednra | endothelin receptor type A | 2.011119162 | 1.428087155 | 0.479276167 | 0.009594 | 0.244306 |
| Csmd1 | CUB and Sushi multiple domains 1 | 2.316107872 | 1.643780962 | 0.479207841 | 0.000323 | 0.037361 |
| Dock8 | dedicator of cytokinesis 8 | 1.029675657 | 0.730341156 | 0.478513212 | 0.005104 | 0.179213 |
| Nwd2 | NACHT and WD repeat domain containing 2 | 2.873911426 | 2.041154962 | 0.478256749 | 0.015756 | 0.307705 |
| Lonrf3 | LON peptidase N-terminal domain and ring fi | 2.029569494 | 1.440253818 | 0.477482773 | 0.035508 | 0.421479 |
| Adamts17 | a disintegrin-like and metallopeptidase (repr | 1.305651678 | 0.928480584 | 0.477237162 | 0.010365 | 0.255897 |
| Prox1os | prospero homeobox 1, opposite strand | 9.671646786 | 6.898228559 | 0.476029039 | 0.022902 | 0.357433 |

| | | | | | | |
|---|---|---|---|---|---|---|
| Chrdl1 | chordin-like 1 | 1.047183069 | 0.745917813 | 0.473601755 | 0.044957 | 0.461002 |
| Cblb | Casitas B-lineage lymphoma b | 3.404753837 | 2.428545088 | 0.472850959 | 0.002502 | 0.123962 |
| Prdm8 | PR domain containing 8 | 11.06340401 | 7.904405659 | 0.472824852 | 0.027429 | 0.379505 |
| Pdlim1 | PDZ and LIM domain 1 (elfin) | 1.033332843 | 0.73517431 | 0.472256435 | 0.039908 | 0.442148 |
| Susd5 | sushi domain containing 5 | 2.848787898 | 2.033002239 | 0.470965207 | 0.006078 | 0.197766 |
| Setbp1 | SET binding protein 1 | 3.728248903 | 2.665266633 | 0.468262037 | 0.000932 | 0.069618 |
| Lingo3 | leucine rich repeat and Ig domain containing | 13.20320215 | 9.463389383 | 0.4666765 | 0.008636 | 0.23324 |
| Ripk2 | receptor (TNFRSF)-interacting serine-threonin | 2.487716246 | 1.782326076 | 0.466051285 | 0.01819 | 0.3229 |
| Nexmif | neurite extension and migration factor | 4.46246253 | 3.197896192 | 0.465003662 | 0.000545 | 0.050274 |
| Cebpd | CCAAT/enhancer binding protein (C/EBP), del | 6.28219084 | 4.528170058 | 0.459644691 | 0.024729 | 0.365666 |
| Rbfox1 | RNA binding protein, fox-1 homolog (C. elega | 65.66195278 | 47.31226911 | 0.458940975 | 0.000886 | 0.067868 |
| Hsd11b1 | hydroxysteroid 11-beta dehydrogenase 1 | 15.28178379 | 11.01979307 | 0.45779604 | 0.000139 | 0.022109 |
| Dhx33 | DEAH (Asp-Glu-Ala-His) box polypeptide 33 | 6.158583603 | 4.44416142 | 0.456766506 | 0.000864 | 0.066502 |
| Cdh8 | cadherin 8 | 8.421138903 | 6.098299067 | 0.451181011 | 0.001864 | 0.107364 |
| P2ry6 | pyrimidinergic receptor P2Y, G-protein coup | 1.832103273 | 1.325957224 | 0.450036153 | 0.027955 | 0.382242 |
| Rrm2 | ribonucleotide reductase M2 | 2.153998209 | 1.566311809 | 0.447851316 | 0.020014 | 0.334904 |
| Orai2 | ORAI calcium release-activated calcium modu | 43.10503494 | 31.32502016 | 0.446886945 | 0.000119 | 0.019405 |
| Kcnt2 | potassium channel, subfamily T, member 2 | 3.514838115 | 2.554822543 | 0.445639706 | 0.014499 | 0.295459 |
| Wdhd1 | WD repeat and HMG-box DNA binding protei | 0.992623717 | 0.721988295 | 0.445487817 | 0.045155 | 0.461421 |
| Zfpm2 | zinc finger protein, multitype 2 | 2.966801311 | 2.156080791 | 0.444755429 | 0.016546 | 0.31404 |
| Atad2b | ATPase family, AAA domain containing 2B | 1.837350657 | 1.335449209 | 0.444753149 | 0.000815 | 0.064539 |
| Rlf | rearranged L-myc fusion sequence | 5.071139297 | 3.687872941 | 0.444499287 | 0.002071 | 0.115461 |
| Rab40b | Rab40B, member RAS oncogene family | 26.59795449 | 19.39268115 | 0.443675934 | 0.01064 | 0.25868 |
| Mcm6 | minichromosome maintenance complex con | 4.470970868 | 3.25579308 | 0.442972894 | 0.000272 | 0.033351 |
| Megf10 | multiple EGF-like-domains 10 | 3.878967399 | 2.823109671 | 0.44141617 | 0.00077 | 0.063082 |
| Pik3c2a | phosphatidylinositol-4-phosphate 3-kinase c | 2.65737725 | 1.932896354 | 0.441382074 | 0.024319 | 0.363714 |
| Slc16a7 | solute carrier family 16 (monocarboxylic acid | 3.937071918 | 2.872153383 | 0.439643697 | 0.001023 | 0.074646 |
| Smim3 | small integral membrane protein 3 | 14.24161863 | 10.39757322 | 0.438518668 | 0.001581 | 0.097312 |
| Prkce | protein kinase C, epsilon | 104.8791488 | 76.67863434 | 0.437737062 | 0.000452 | 0.045039 |
| Pter | phosphotriesterase related | 25.81117829 | 18.89926163 | 0.437352269 | 0.011254 | 0.264259 |
| Npnt | nephronectin | 10.27165133 | 7.513260916 | 0.436451345 | 0.002504 | 0.123962 |
| Wipf3 | WAS/WASL interacting protein family, memb | 73.25111035 | 53.66722108 | 0.436361163 | 0.012139 | 0.274334 |
| Srl | sarcalumenin | 4.538244021 | 3.325189267 | 0.435074254 | 0.011496 | 0.267239 |
| Zfp72 | zinc finger protein 72 | 1.56865878 | 1.148660682 | 0.434573393 | 0.016562 | 0.31404 |
| Onecut2 | one cut domain, family member 2 | 0.511951712 | 0.374882977 | 0.434078648 | 0.045467 | 0.462192 |
| Sall3 | spalt like transcription factor 3 | 1.655302757 | 1.21104509 | 0.433602838 | 0.014001 | 0.289976 |
| Grin2a | glutamate receptor, ionotropic, NMDA2A (ep | 13.443562 | 9.866015135 | 0.433101761 | 0.001409 | 0.090323 |
| Cdc40 | cell division cycle 40 | 7.636677511 | 5.613792421 | 0.430864895 | 0.0085 | 0.232553 |
| Ptpro | protein tyrosine phosphatase, receptor type, | 11.71818366 | 8.625737109 | 0.427882826 | 0.001848 | 0.107364 |

| | | | | | | |
|---|---|---|---|---|---|---|
| Klf9 | Kruppel-like factor 9 | 62.30674133 | 45.84007547 | 0.427257437 | 0.002518 | 0.12422 |
| Dagla | diacylglycerol lipase, alpha | 30.71527396 | 22.6294996 | 0.426114889 | 0.001136 | 0.078396 |
| Igsf3 | immunoglobulin superfamily, member 3 | 4.338215756 | 3.196366703 | 0.425698259 | 0.010566 | 0.258274 |
| Trib1 | tribbles pseudokinase 1 | 3.025386616 | 2.226333726 | 0.425312569 | 0.003061 | 0.139048 |
| Tanc1 | tetratricopeptide repeat, ankyrin repeat and | 6.716667237 | 4.954343485 | 0.424250195 | 0.007592 | 0.220817 |
| Epha3 | Eph receptor A3 | 2.025742521 | 1.494197664 | 0.424132941 | 0.008575 | 0.23271 |
| Pdyn | prodynorphin | 5.373843143 | 3.967855863 | 0.423619526 | 0.00776 | 0.222945 |
| Neurod2 | neurogenic differentiation 2 | 64.04738084 | 47.3683042 | 0.422412585 | 0.011518 | 0.267239 |
| Ccdc71l | coiled-coil domain containing 71 like | 10.58468771 | 7.823508689 | 0.421946085 | 0.000568 | 0.05165 |
| Cd33 | CD33 antigen | 1.466578432 | 1.086619266 | 0.421225162 | 0.012516 | 0.277167 |
| Slc35f3 | solute carrier family 35, member F3 | 16.13595659 | 11.94083513 | 0.420079895 | 0.001081 | 0.076812 |
| Dusp6 | dual specificity phosphatase 6 | 20.99830283 | 15.55789252 | 0.418535097 | 0.006837 | 0.208871 |
| Mfsd4a | major facilitator superfamily domain contair | 6.254340799 | 4.631758062 | 0.418378346 | 7.12E-05 | 0.014589 |
| Kdm7a | lysine (K)-specific demethylase 7A | 6.893881134 | 5.103143188 | 0.417997143 | 0.004391 | 0.165947 |
| Nfib | nuclear factor I/B | 16.75048069 | 12.42309528 | 0.417593425 | 0.004665 | 0.171304 |
| Sstr2 | somatostatin receptor 2 | 11.20907614 | 8.317372618 | 0.417134579 | 0.009018 | 0.236533 |
| Mycn | v-myc avian myelocytomatosis viral related c | 5.03911461 | 3.738426064 | 0.415795313 | 0.010069 | 0.251078 |
| Smchd1 | SMC hinge domain containing 1 | 2.041230129 | 1.515362863 | 0.414496317 | 0.002356 | 0.120747 |
| Hpgds | hematopoietic prostaglandin D synthase | 1.715825454 | 1.278504042 | 0.413141173 | 0.026666 | 0.376034 |
| Sema5b | sema domain, seven thrombospondin repeat | 3.366544689 | 2.505272095 | 0.411595394 | 0.00478 | 0.173499 |
| Ppp3ca | protein phosphatase 3, catalytic subunit, alp | 221.5933058 | 165.149216 | 0.41153488 | 0.01088 | 0.260931 |
| Plxna2 | plexin A2 | 6.462162563 | 4.806641935 | 0.411300247 | 0.001817 | 0.106678 |
| Il10ra | interleukin 10 receptor, alpha | 2.209873535 | 1.645681886 | 0.410831021 | 0.041731 | 0.449122 |
| Mef2c | myocyte enhancer factor 2C | 10.17104409 | 7.570192821 | 0.410814286 | 0.000166 | 0.024184 |
| Btbd3 | BTB (POZ) domain containing 3 | 78.00397247 | 58.11748791 | 0.410038739 | 2.67E-05 | 0.007839 |
| Bdnf | brain derived neurotrophic factor | 9.868143913 | 7.360028598 | 0.409411394 | 0.007358 | 0.218248 |
| Fat3 | FAT atypical cadherin 3 | 1.999012483 | 1.493660858 | 0.405097164 | 0.006533 | 0.204904 |
| Sh3bp2 | SH3-domain binding protein 2 | 1.816575491 | 1.358206592 | 0.404925999 | 0.029161 | 0.389312 |
| Shisa6 | shisa family member 6 | 16.89057912 | 12.6388174 | 0.40421089 | 0.006752 | 0.208257 |
| Cemip | cell migration inducing protein, hyaluronan | 3.413928172 | 2.555588064 | 0.401887695 | 0.004099 | 0.161621 |
| Trps1 | transcriptional repressor GATA binding 1 | 3.154048017 | 2.359543942 | 0.401562873 | 0.00404 | 0.16123 |
| Frrs1l | ferric-chelate reductase 1 like | 52.32448272 | 39.25401097 | 0.400861227 | 0.001032 | 0.074878 |
| Rasgrp1 | RAS guanyl releasing protein 1 | 88.45519365 | 66.44035852 | 0.399580102 | 0.008924 | 0.236504 |
| Epha5 | Eph receptor A5 | 11.30395167 | 8.490692759 | 0.399488708 | 0.005966 | 0.19607 |
| Mmp16 | matrix metallopeptidase 16 | 1.992874245 | 1.495256557 | 0.398426655 | 0.036272 | 0.424833 |
| Ptgs2 | prostaglandin-endoperoxide synthase 2 | 5.396893271 | 4.054202802 | 0.397665485 | 0.026962 | 0.376587 |
| Egr3 | early growth response 3 | 24.28010226 | 18.25690882 | 0.397266326 | 0.005116 | 0.179231 |
| Kcns2 | K+ voltage-gated channel, subfamily S, 2 | 6.967284877 | 5.246580113 | 0.395655756 | 0.009631 | 0.244306 |
| Slc7a14 | solute carrier family 7 (cationic amino acid tr | 16.19323204 | 12.18674941 | 0.395550632 | 0.003375 | 0.144949 |

| | | | | | | |
|---|---|---|---|---|---|---|
| Zmym1 | zinc finger, MYM domain containing 1 | 2.207236944 | 1.661541046 | 0.395197818 | 0.013138 | 0.2831 |
| Pcdh20 | protocadherin 20 | 24.5764053 | 18.54276704 | 0.393243438 | 0.021988 | 0.350747 |
| Capn3 | calpain 3 | 2.962899275 | 2.234843875 | 0.392785784 | 0.016621 | 0.31404 |
| Tnr | tenascin R | 19.24025088 | 14.492222 | 0.392777921 | 0.01234 | 0.276598 |
| Nck2 | non-catalytic region of tyrosine kinase adapt | 13.7681498 | 10.38292245 | 0.392733238 | 0.006758 | 0.208257 |
| Ipcef1 | interaction protein for cytohesin exchange fa | 3.161485849 | 2.379976509 | 0.392304315 | 0.010309 | 0.254867 |
| Kcnk1 | potassium channel, subfamily K, member 1 | 56.36508565 | 42.54095552 | 0.390968526 | 1.46E-05 | 0.005312 |
| Atp2b1 | ATPase, Ca++ transporting, plasma membran | 48.4792452 | 36.62288808 | 0.390939054 | 0.002294 | 0.11979 |
| Syndig1 | synapse differentiation inducing 1 | 16.78138666 | 12.68324245 | 0.39021168 | 0.009452 | 0.243219 |
| Arhgap15 | Rho GTPase activating protein 15 | 1.702036005 | 1.286722549 | 0.390117462 | 0.024288 | 0.363714 |
| Robo2 | roundabout guidance receptor 2 | 5.664572904 | 4.279518074 | 0.390037869 | 0.01007 | 0.251078 |
| Ptprj | protein tyrosine phosphatase, receptor type, | 16.44443544 | 12.431023 | 0.389428207 | 0.002333 | 0.120423 |
| Btg2 | B cell translocation gene 2, anti-proliferative | 9.301220757 | 7.032669415 | 0.388744979 | 0.0082 | 0.227225 |
| Skida1 | SKI/DACH domain containing 1 | 4.087243383 | 3.094135926 | 0.387595046 | 0.00544 | 0.185818 |
| Kif26b | kinesin family member 26B | 2.574184212 | 1.948651875 | 0.385193347 | 0.024024 | 0.363714 |
| Camk2b | calcium/calmodulin-dependent protein kina | 139.2114683 | 105.6330892 | 0.385126396 | 0.006526 | 0.204904 |
| Zfp81 | zinc finger protein 81 | 4.364578743 | 3.310283396 | 0.385074126 | 0.000667 | 0.056816 |
| Fnip2 | folliculin interacting protein 2 | 3.45116971 | 2.612763523 | 0.384767482 | 0.004483 | 0.16849 |
| Vcan | versican | 2.382529555 | 1.806350478 | 0.383411216 | 0.005887 | 0.195705 |
| Scn3a | sodium channel, voltage-gated, type III, alph | 3.365000552 | 2.554336979 | 0.383023206 | 0.016848 | 0.315592 |
| Plcl1 | phospholipase C-like 1 | 8.266176862 | 6.274065712 | 0.382906228 | 0.00409 | 0.161621 |
| 4921524J1 | RIKEN cDNA 4921524J17 gene | 12.03126489 | 9.134300063 | 0.382657363 | 0.002917 | 0.13602 |
| Sipa1l3 | signal-induced proliferation-associated 1 like | 9.495964716 | 7.212081416 | 0.381802596 | 0.004341 | 0.165947 |
| Kbtbd11 | kelch repeat and BTB (POZ) domain containir | 37.29625357 | 28.35713395 | 0.380917168 | 0.000362 | 0.03939 |
| Npy1r | neuropeptide Y receptor Y1 | 8.751285976 | 6.666489003 | 0.379538434 | 0.009658 | 0.244306 |
| Tmem144 | transmembrane protein 144 | 4.655026564 | 3.544621742 | 0.379250557 | 0.003284 | 0.142987 |
| Mctp1 | multiple C2 domains, transmembrane 1 | 8.033759707 | 6.116634639 | 0.378610564 | 0.017038 | 0.315784 |
| Nr3c2 | nuclear receptor subfamily 3, group C, memb | 27.80425519 | 21.1951828 | 0.37815532 | 0.005985 | 0.196099 |
| Slc39a6 | solute carrier family 39 (metal ion transporte | 21.2124606 | 16.1667476 | 0.377901759 | 0.000288 | 0.034695 |
| Cacna1h | calcium channel, voltage-dependent, T type, | 10.35245253 | 7.888570315 | 0.377679311 | 0.002183 | 0.118768 |
| Inf2 | inverted formin, FH2 and WH2 domain conta | 31.33416124 | 23.91237481 | 0.375884769 | 0.008041 | 0.225748 |
| Disp3 | dispatched RND transporter family member 3 | 7.720287337 | 5.886424859 | 0.375369087 | 0.008667 | 0.23324 |
| Scn3b | sodium channel, voltage-gated, type III, beta | 52.17264001 | 39.86337572 | 0.375288863 | 0.008076 | 0.225748 |
| Nfia | nuclear factor I/A | 9.65752951 | 7.373805921 | 0.374901104 | 2.62E-05 | 0.007829 |
| Cilp2 | cartilage intermediate layer protein 2 | 1.322583545 | 1.008940162 | 0.374783155 | 0.036099 | 0.424092 |
| Bend4 | BEN domain containing 4 | 1.117482446 | 0.851686663 | 0.374680777 | 0.017984 | 0.321183 |
| Cdh9 | cadherin 9 | 9.560276523 | 7.305277409 | 0.373824703 | 0.014674 | 0.297056 |
| Rasa1 | RAS p21 protein activator 1 | 10.03635256 | 7.669908188 | 0.37353521 | 0.000808 | 0.064539 |
| Qser1 | glutamine and serine rich 1 | 2.59068066 | 1.978455664 | 0.373031426 | 0.01529 | 0.304034 |

| | | | | | | |
|---|---|---|---|---|---|---|
| Nedd4l | neural precursor cell expressed, developmen | 10.37291534 | 7.938363951 | 0.37205484 | 0.004996 | 0.177891 |
| Tiam2 | T cell lymphoma invasion and metastasis 2 | 2.702549951 | 2.067534093 | 0.371139469 | 0.003292 | 0.142987 |
| Lrrc10b | leucine rich repeat containing 10B | 54.36872947 | 41.66978485 | 0.370555129 | 0.007467 | 0.219002 |
| Frmd4b | FERM domain containing 4B | 3.147983431 | 2.409345945 | 0.370355521 | 0.000509 | 0.048725 |
| Fam19a2 | family with sequence similarity 19, member / | 18.21574963 | 13.9689643 | 0.36939948 | 0.003631 | 0.150965 |
| Pxylp1 | 2-phosphoxylose phosphatase 1 | 2.823308694 | 2.169552368 | 0.368450338 | 0.030912 | 0.399905 |
| Ryr2 | ryanodine receptor 2, cardiac | 5.461906245 | 4.190948984 | 0.366941977 | 0.018683 | 0.327041 |
| Garem1 | GRB2 associated regulator of MAPK1 subtype | 7.151858426 | 5.483619882 | 0.366619168 | 0.01067 | 0.25868 |
| Kmt2c | lysine (K)-specific methyltransferase 2C | 3.75564644 | 2.88083993 | 0.366440206 | 0.013114 | 0.2831 |
| Tcf4 | transcription factor 4 | 19.9320912 | 15.31419306 | 0.366000079 | 0.003016 | 0.137747 |
| Arl15 | ADP-ribosylation factor-like 15 | 15.51391086 | 11.93415249 | 0.365351856 | 0.014841 | 0.298633 |
| Pkia | protein kinase inhibitor, alpha | 69.0982778 | 53.12031939 | 0.364842846 | 2.42E-05 | 0.007603 |
| Arhgap20 | Rho GTPase activating protein 20 | 5.431774643 | 4.178014397 | 0.364116852 | 0.000122 | 0.019675 |
| Neurod6 | neurogenic differentiation 6 | 96.99738721 | 74.73901356 | 0.363749768 | 0.04296 | 0.450475 |
| Qtrt2 | queuine tRNA-ribosyltransferase accessory su | 3.680721537 | 2.835580157 | 0.363120757 | 0.018371 | 0.324251 |
| Samd12 | sterile alpha motif domain containing 12 | 3.919173059 | 3.014825558 | 0.361939245 | 0.015554 | 0.307021 |
| Nav1 | neuron navigator 1 | 9.093305733 | 7.003064604 | 0.361902562 | 0.007567 | 0.220817 |
| 2010300C( | RIKEN cDNA 2010300C02 gene | 72.79806818 | 56.16318111 | 0.361068704 | 0.011755 | 0.270195 |
| Spsb1 | splA/ryanodine receptor domain and SOCS b | 7.181614645 | 5.533670029 | 0.360481777 | 0.006123 | 0.198769 |
| Emx2 | empty spiracles homeobox 2 | 6.275421045 | 4.843017234 | 0.359531987 | 0.017567 | 0.318417 |
| Ablim3 | actin binding LIM protein family, member 3 | 11.02751102 | 8.516314663 | 0.358945379 | 0.012532 | 0.277167 |
| Chd1 | chromodomain helicase DNA binding protein | 2.808074846 | 2.166169081 | 0.358217734 | 0.00314 | 0.139773 |
| Mdc1 | mediator of DNA damage checkpoint 1 | 2.214718625 | 1.709603711 | 0.357953574 | 0.010224 | 0.253124 |
| Sh3kbp1 | SH3-domain kinase binding protein 1 | 4.230816344 | 3.268419073 | 0.357918685 | 0.000389 | 0.04091 |
| Atrx | ATRX, chromatin remodeler | 7.173235204 | 5.539111589 | 0.357875467 | 0.001286 | 0.086189 |
| Lmo7 | LIM domain only 7 | 7.118425087 | 5.506819843 | 0.356462872 | 0.011522 | 0.267239 |
| Prickle2 | prickle planar cell polarity protein 2 | 34.97717143 | 27.04504097 | 0.356227685 | 0.000414 | 0.042141 |
| Napepld | N-acyl phosphatidylethanolamine phospholi | 8.230811918 | 6.365427603 | 0.355165751 | 0.005744 | 0.19214 |
| Lamc1 | laminin, gamma 1 | 9.785143841 | 7.567766178 | 0.354285712 | 0.019337 | 0.330205 |
| Chgb | chromogranin B | 228.3928925 | 177.4701849 | 0.35037143 | 0.035753 | 0.42352 |
| Zfp729a | zinc finger protein 729a | 1.789073222 | 1.389276669 | 0.349858571 | 0.015864 | 0.308301 |
| Bag4 | BCL2-associated athanogene 4 | 10.57477728 | 8.219722077 | 0.349586135 | 0.002694 | 0.129245 |
| Tle1 | transducin-like enhancer of split 1 | 8.532067065 | 6.636474368 | 0.348370743 | 0.00899 | 0.236504 |
| Ralgapa2 | Ral GTPase activating protein, alpha subunit | 1.278880601 | 0.994598303 | 0.347715099 | 0.008077 | 0.225748 |
| P2ry13 | purinergic receptor P2Y, G-protein coupled 1 | 7.740550846 | 6.029845836 | 0.347531775 | 0.017083 | 0.315784 |
| Inka2 | inka box actin regulator 2 | 23.04431679 | 17.93533419 | 0.347183993 | 0.001137 | 0.078396 |
| Kalrn | kalirin, RhoGEF kinase | 23.88385535 | 18.6019846 | 0.346048621 | 0.005433 | 0.185818 |
| Stim2 | stromal interaction molecule 2 | 22.61939035 | 17.62690708 | 0.345943152 | 0.00509 | 0.179091 |
| Zcchc14 | zinc finger, CCHC domain containing 14 | 16.37692044 | 12.7507314 | 0.345799436 | 0.000356 | 0.03939 |

| | | | | | | |
|---|---|---|---|---|---|---|
| Nol4 | nucleolar protein 4 | 12.90514379 | 10.05239742 | 0.34566026 | 0.000816 | 0.064539 |
| Zfp101 | zinc finger protein 101 | 1.526226377 | 1.189089476 | 0.345527882 | 0.019991 | 0.334904 |
| Il1rap | interleukin 1 receptor accessory protein | 6.527857672 | 5.085988247 | 0.345160613 | 0.000602 | 0.053064 |
| Auts2 | autism susceptibility candidate 2 | 4.188016077 | 3.265735822 | 0.344407015 | 0.003846 | 0.156222 |
| Sorl1 | sortilin-related receptor, LDLR class A repeats | 23.54970763 | 18.36716949 | 0.343871931 | 0.006462 | 0.204144 |
| Rasgrf2 | RAS protein-specific guanine nucleotide-relea | 8.753841843 | 6.827891274 | 0.343513328 | 0.004987 | 0.177891 |
| 2700049A0 | RIKEN cDNA 2700049A03 gene | 1.42610267 | 1.11323777 | 0.343295631 | 0.024657 | 0.365666 |
| 2810025M | RIKEN cDNA 2810025M15 gene | 15.50615891 | 12.11620159 | 0.342594547 | 0.004185 | 0.163555 |
| Bcl6 | B cell leukemia/lymphoma 6 | 6.589659596 | 5.149511255 | 0.342179802 | 0.040428 | 0.444433 |
| Golm1 | golgi membrane protein 1 | 16.45158177 | 12.84997465 | 0.341705694 | 0.013451 | 0.287051 |
| Prkx | protein kinase, X-linked | 3.611921465 | 2.819727522 | 0.341567256 | 0.002474 | 0.123592 |
| Slc1a2 | solute carrier family 1 (glial high affinity gluta | 361.0986736 | 281.7725509 | 0.34125411 | 0.005267 | 0.182498 |
| Exoc6 | exocyst complex component 6 | 15.5286065 | 12.13795407 | 0.341020845 | 0.002331 | 0.120423 |
| Nrp1 | neuropilin 1 | 8.318253769 | 6.497410596 | 0.340787707 | 0.008525 | 0.232607 |
| Ptprz1 | protein tyrosine phosphatase, receptor type | 24.45822038 | 19.101284 | 0.340493334 | 0.001193 | 0.081211 |
| Tiam1 | T cell lymphoma invasion and metastasis 1 | 11.96108541 | 9.347370533 | 0.340380107 | 0.005378 | 0.184785 |
| E2f3 | E2F transcription factor 3 | 7.606908024 | 5.951737983 | 0.339322632 | 0.001477 | 0.092627 |
| Spry1 | sprouty RTK signaling antagonist 1 | 5.865840541 | 4.589732849 | 0.338771548 | 0.007689 | 0.221946 |
| Prr14l | proline rich 14-like | 6.128473567 | 4.797355023 | 0.337922641 | 0.007167 | 0.214723 |
| Atf6 | activating transcription factor 6 | 9.742634644 | 7.632771043 | 0.33672454 | 0.002288 | 0.11979 |
| Fry | FRY microtubule binding protein | 4.163187362 | 3.263798738 | 0.336067317 | 0.011046 | 0.263468 |
| Zeb2 | zinc finger E-box binding homeobox 2 | 12.34012918 | 9.680687991 | 0.335795356 | 0.002703 | 0.129245 |
| Tspan18 | tetraspanin 18 | 5.391432525 | 4.231338566 | 0.335662259 | 0.036199 | 0.424258 |
| Zfp26 | zinc finger protein 26 | 1.249315797 | 0.979260577 | 0.335120176 | 0.020653 | 0.339596 |
| Lrp1b | low density lipoprotein-related protein 1B (c | 1.432564271 | 1.123910304 | 0.334651688 | 0.010542 | 0.258095 |
| Itpr2 | inositol 1,4,5-triphosphate receptor 2 | 3.543383077 | 2.777848324 | 0.334386998 | 0.01075 | 0.259227 |
| Irak2 | interleukin-1 receptor-associated kinase 2 | 3.462229215 | 2.719365328 | 0.334120578 | 0.016209 | 0.31191 |
| Erc2 | ELKS/RAB6-interacting/CAST family member | 22.50711201 | 17.6754828 | 0.333961716 | 0.018574 | 0.326431 |
| Mpped2 | metallophosphoesterase domain containing | 12.12310532 | 9.528416684 | 0.333840718 | 0.015989 | 0.309549 |
| Mpeg1 | macrophage expressed gene 1 | 10.34898771 | 8.125564395 | 0.333481934 | 0.001978 | 0.111379 |
| Als2 | alsin Rho guanine nucleotide exchange factor | 7.223429642 | 5.672293486 | 0.333271072 | 0.005067 | 0.179001 |
| Lhfpl2 | lipoma HMGIC fusion partner-like 2 | 7.410844369 | 5.824833848 | 0.332476137 | 0.004366 | 0.165947 |
| Cnksr2 | connector enhancer of kinase suppressor of R | 21.7582738 | 17.11580096 | 0.332088877 | 0.018668 | 0.327041 |
| Tmem196 | transmembrane protein 196 | 2.118279946 | 1.663535566 | 0.331843408 | 0.047623 | 0.472936 |
| Chrm1 | cholinergic receptor, muscarinic 1, CNS | 33.80344771 | 26.6168693 | 0.330953448 | 0.032954 | 0.411041 |
| Pdgfra | platelet derived growth factor receptor, alph | 10.95202515 | 8.612982873 | 0.330548382 | 0.006663 | 0.206058 |
| Ctdspl2 | CTD (carboxy-terminal domain, RNA polymer | 2.823438026 | 2.222626777 | 0.329088985 | 0.018417 | 0.324311 |
| Cttnbp2nl | CTTNBP2 N-terminal like | 4.84023744 | 3.809318417 | 0.328917357 | 0.007106 | 0.213581 |
| Hecw1 | HECT, C2 and WW domain containing E3 ubi | 3.806119029 | 2.996557744 | 0.32887628 | 0.039837 | 0.441913 |

| | | | | | | |
|---|---|---|---|---|---|---|
| C1ql2 | complement component 1, q subcomponent | 45.56252873 | 35.98512209 | 0.327761584 | 0.024339 | 0.363714 |
| Plcb1 | phospholipase C, beta 1 | 16.44343421 | 12.97264622 | 0.327479106 | 0.020029 | 0.334904 |
| Rbm12 | RNA binding motif protein 12 | 2.600863365 | 2.05035359 | 0.327122562 | 0.038814 | 0.436339 |
| Lrp4 | low density lipoprotein receptor-related pro | 4.874505527 | 3.840559788 | 0.326753188 | 0.022667 | 0.355156 |
| Ascl1 | achaete-scute family bHLH transcription fact | 4.021682091 | 3.177510031 | 0.32671502 | 0.035113 | 0.417415 |
| Pfkfb3 | 6-phosphofructo-2-kinase/fructose-2,6-biph | 5.750149995 | 4.533932244 | 0.326330388 | 0.021163 | 0.343188 |
| Mycl | v-myc avian myelocytomatosis viral oncogen | 16.8275372 | 13.28659096 | 0.326116393 | 0.001449 | 0.091226 |
| Scara3 | scavenger receptor class A, member 3 | 14.30147099 | 11.29424707 | 0.326024699 | 0.021086 | 0.343188 |
| Ago1 | argonaute RISC catalytic subunit 1 | 15.78593317 | 12.46028898 | 0.325881922 | 0.000721 | 0.060481 |
| Carnmt1 | carnosine N-methyltransferase 1 | 5.193399672 | 4.10189728 | 0.324224065 | 0.025922 | 0.372836 |
| Prkd1 | protein kinase D1 | 5.969133385 | 4.723918017 | 0.324003063 | 0.017762 | 0.319156 |
| Homer3 | homer scaffolding protein 3 | 13.41285397 | 10.6104252 | 0.32361046 | 0.018112 | 0.322482 |
| Cebpa | CCAAT/enhancer binding protein (C/EBP), alp | 11.4294757 | 9.036834995 | 0.323433572 | 0.024944 | 0.367325 |
| Gabrb3 | gamma-aminobutyric acid (GABA) A receptor | 53.99019292 | 42.74606684 | 0.323389304 | 0.007395 | 0.218248 |
| Dab1 | disabled 1 | 16.99457418 | 13.44623633 | 0.322969424 | 0.003002 | 0.137611 |
| Gpr68 | G protein-coupled receptor 68 | 3.72792255 | 2.948573024 | 0.32238327 | 0.026882 | 0.376367 |
| Cyp7b1 | cytochrome P450, family 7, subfamily b, poly | 13.03797258 | 10.33942987 | 0.321256123 | 0.012027 | 0.27285 |
| Fgd4 | FYVE, RhoGEF and PH domain containing 4 | 2.636509805 | 2.086842562 | 0.321214303 | 0.012361 | 0.276598 |
| Add2 | adducin 2 (beta) | 37.65643211 | 29.83506716 | 0.32098176 | 0.002988 | 0.1375 |
| Tet3 | tet methylcytosine dioxygenase 3 | 5.178955648 | 4.100278734 | 0.320834446 | 0.017032 | 0.315784 |
| Pml | promyelocytic leukemia | 1.878665799 | 1.487028239 | 0.320705822 | 0.044253 | 0.456743 |
| Cxadr | coxsackie virus and adenovirus receptor | 4.709984509 | 3.732032804 | 0.320647503 | 0.01313 | 0.2831 |
| Clstn2 | calsyntenin 2 | 22.11893349 | 17.53924075 | 0.320448498 | 0.018377 | 0.324251 |
| Egfem1 | EGF-like and EMI domain containing 1 | 3.12455243 | 2.480443833 | 0.320441176 | 0.020887 | 0.341521 |
| Zfp518b | zinc finger protein 518B | 2.668016996 | 2.111529593 | 0.320001214 | 0.027835 | 0.381825 |
| Paqr9 | progestin and adipoQ receptor family membe | 13.73879317 | 10.89599364 | 0.319966681 | 0.016618 | 0.31404 |
| Rasal2 | RAS protein activator like 2 | 6.919648826 | 5.485233787 | 0.319954254 | 0.001677 | 0.101923 |
| Galnt17 | polypeptide N-acetylgalactosaminyltransfera | 18.37769454 | 14.5767798 | 0.319927893 | 0.007497 | 0.21953 |
| Slc7a2 | solute carrier family 7 (cationic amino acid tr | 5.93367439 | 4.702985912 | 0.31940254 | 0.017708 | 0.318827 |
| Slc30a3 | solute carrier family 30 (zinc transporter), me | 24.01311468 | 19.07435345 | 0.318968153 | 0.041089 | 0.447916 |
| Pitpnm2 | phosphatidylinositol transfer protein, memb | 20.86499946 | 16.55715148 | 0.318950568 | 0.00118 | 0.080589 |
| Emid1 | EMI domain containing 1 | 5.801468089 | 4.608741259 | 0.31883749 | 0.036937 | 0.427789 |
| Ccdc85a | coiled-coil domain containing 85A | 18.65660137 | 14.80970379 | 0.318525597 | 0.004735 | 0.172749 |
| Zfp831 | zinc finger protein 831 | 2.389148025 | 1.896552588 | 0.318150119 | 0.032334 | 0.407104 |
| Sh3bp5 | SH3-domain binding protein 5 (BTK-associate | 40.54039357 | 32.20794948 | 0.31792964 | 0.028541 | 0.385806 |
| Rfx7 | regulatory factor X, 7 | 1.954816347 | 1.551549313 | 0.317545201 | 0.009537 | 0.244306 |
| Fam19a1 | family with sequence similarity 19, member A | 13.06634524 | 10.39561587 | 0.317071309 | 0.0382 | 0.434338 |
| Kcnh1 | potassium voltage-gated channel, subfamily I | 6.860192607 | 5.44578505 | 0.317027489 | 0.033576 | 0.411285 |
| Slc30a6 | solute carrier family 30 (zinc transporter), me | 4.416945668 | 3.510452184 | 0.316965994 | 0.0299 | 0.3931 |

| | | | | | | |
|---|---|---|---|---|---|---|
| Ccdc88a | coiled coil domain containing 88A | 7.42968439 | 5.900004846 | 0.316897949 | 0.013819 | 0.289375 |
| Col4a1 | collagen, type IV, alpha 1 | 16.04508887 | 12.74085018 | 0.316814741 | 0.037929 | 0.432603 |
| C130071C( | RIKEN cDNA C130071C03 gene | 10.21792376 | 8.134205329 | 0.316444622 | 0.041714 | 0.449122 |
| Zfp867 | zinc finger protein 867 | 4.083425357 | 3.25355315 | 0.315702081 | 0.026716 | 0.376183 |
| Mex3b | mex3 RNA binding family member B | 8.982188387 | 7.149686539 | 0.315605235 | 0.017226 | 0.316008 |
| Kcna4 | potassium voltage-gated channel, shaker-rela | 5.005914283 | 3.985330809 | 0.315534876 | 0.021434 | 0.345671 |
| Bmp2k | BMP2 inducible kinase | 2.830174887 | 2.249900681 | 0.31539678 | 0.014468 | 0.295459 |
| Eva1a | eva-1 homolog A (C. elegans) | 9.967689377 | 7.923153451 | 0.315323054 | 0.006222 | 0.200208 |
| Kcnh7 | potassium voltage-gated channel, subfamily I | 2.218121913 | 1.76491245 | 0.315243603 | 0.022158 | 0.351612 |
| Rcor1 | REST corepressor 1 | 6.085523279 | 4.839342193 | 0.314745179 | 0.029879 | 0.3931 |
| Ncan | neurocan | 48.74522538 | 38.76102752 | 0.3147437 | 0.002936 | 0.136556 |
| Dgkz | diacylglycerol kinase zeta | 107.4851837 | 85.59084752 | 0.314131952 | 0.002168 | 0.118362 |
| Gabra5 | gamma-aminobutyric acid (GABA) A receptor | 75.87596758 | 60.5154905 | 0.313192743 | 0.036309 | 0.424984 |
| Prkca | protein kinase C, alpha | 26.39437047 | 21.03651601 | 0.312993694 | 0.029338 | 0.389892 |
| Serpina3n | serine (or cysteine) peptidase inhibitor, clade | 22.29532368 | 17.78738662 | 0.312500946 | 0.048897 | 0.475925 |
| Nrde2 | nrde-2 necessary for RNA interference, domai | 4.653420054 | 3.713964566 | 0.312461687 | 0.019 | 0.327873 |
| Tmem74 | transmembrane protein 74 | 5.903985889 | 4.707112316 | 0.312386903 | 0.029994 | 0.393614 |
| Slc4a7 | solute carrier family 4, sodium bicarbonate c | 3.051682674 | 2.434399045 | 0.312357927 | 0.021132 | 0.343188 |
| Zfx | zinc finger protein X-linked | 3.657979822 | 2.918378096 | 0.310209674 | 0.031038 | 0.400448 |
| Eya1 | EYA transcriptional coactivator and phospha | 2.671748346 | 2.134753881 | 0.309888529 | 0.025373 | 0.369715 |
| Camkk1 | calcium/calmodulin-dependent protein kina | 47.2325634 | 37.74967971 | 0.30982309 | 0.023702 | 0.362124 |
| Lysmd3 | LysM, putative peptidoglycan-binding, doma | 2.883832613 | 2.306037143 | 0.309818766 | 0.033953 | 0.411285 |
| Foxk1 | forkhead box K1 | 8.46571172 | 6.755180533 | 0.309523391 | 0.030747 | 0.3988 |
| Abi2 | abl-interactor 2 | 27.43299293 | 21.93645975 | 0.308620741 | 0.005083 | 0.179091 |
| Spry2 | sprouty RTK signaling antagonist 2 | 22.73274444 | 18.1742493 | 0.308568323 | 0.004614 | 0.170505 |
| Tgfa | transforming growth factor alpha | 12.22946504 | 9.77263763 | 0.308490376 | 0.002345 | 0.12053 |
| Cacna1e | calcium channel, voltage-dependent, R type, | 9.518713435 | 7.610816757 | 0.308477445 | 0.015145 | 0.302784 |
| Naa25 | N(alpha)-acetyltransferase 25, NatB auxiliary | 4.049489175 | 3.23421878 | 0.307592834 | 0.038667 | 0.435798 |
| Ptbp3 | polypyrimidine tract binding protein 3 | 5.12135053 | 4.095502092 | 0.307423307 | 0.003486 | 0.146404 |
| Ankrd45 | ankyrin repeat domain 45 | 10.28659674 | 8.235184767 | 0.30677849 | 0.006968 | 0.211417 |
| Trim2 | tripartite motif-containing 2 | 20.41585201 | 16.34479998 | 0.306521052 | 0.004546 | 0.169288 |
| Stxbp5 | syntaxin binding protein 5 (tomosyn) | 6.821645039 | 5.462939271 | 0.305989679 | 0.022634 | 0.35495 |
| Chn1 | chimerin 1 | 142.3094991 | 114.0825114 | 0.305801611 | 0.023827 | 0.363225 |
| Robo1 | roundabout guidance receptor 1 | 3.709469089 | 2.969577297 | 0.305775933 | 0.025766 | 0.372221 |
| Arl5b | ADP-ribosylation factor-like 5B | 3.965819362 | 3.176163644 | 0.305396932 | 0.023357 | 0.360229 |
| Ackr3 | atypical chemokine receptor 3 | 8.06150553 | 6.448073616 | 0.305067739 | 0.023707 | 0.362124 |
| Plppr5 | phospholipid phosphatase related 5 | 16.38582041 | 13.13831398 | 0.304754941 | 0.006372 | 0.20238 |
| Pgbd5 | piggyBac transposable element derived 5 | 54.2312035 | 43.51356931 | 0.304382994 | 0.025783 | 0.372221 |
| Mfap3 | microfibrillar-associated protein 3 | 6.558230662 | 5.256932442 | 0.304313638 | 0.013373 | 0.286425 |

| | | | | | | |
|---|---|---|---|---|---|---|
| Tgfbr1 | transforming growth factor, beta receptor I | 8.417074499 | 6.742292385 | 0.304057946 | 0.002258 | 0.119646 |
| Tle4 | transducin-like enhancer of split 4 | 12.95596742 | 10.37945899 | 0.303409013 | 0.018303 | 0.323926 |
| Rock1 | Rho-associated coiled-coil containing protei | 6.322767709 | 5.06512597 | 0.303173184 | 0.014636 | 0.297056 |
| Btbd8 | BTB (POZ) domain containing 8 | 8.222825721 | 6.593243679 | 0.302926997 | 0.023353 | 0.360229 |
| Creg2 | cellular repressor of E1A-stimulated genes 2 | 22.07965044 | 17.73069149 | 0.302815579 | 0.005946 | 0.19607 |
| Lmnb1 | lamin B1 | 5.975059455 | 4.79816876 | 0.302384166 | 0.012958 | 0.28183 |
| Ccng2 | cyclin G2 | 10.81193092 | 8.686911364 | 0.30181756 | 0.023206 | 0.360229 |
| Map3k5 | mitogen-activated protein kinase kinase kina | 4.347051513 | 3.488516102 | 0.301303434 | 0.017649 | 0.318417 |
| Gpc1 | glypican 1 | 26.76731197 | 21.50182421 | 0.300967893 | 0.012443 | 0.277167 |
| Foxo1 | forkhead box O1 | 5.348301543 | 4.299362366 | 0.300432498 | 0.006789 | 0.208472 |
| Grin2c | glutamate receptor, ionotropic, NMDA2C (ep | 16.28982935 | 13.0847778 | 0.300355798 | 0.020294 | 0.336853 |
| Zfp738 | zinc finger protein 738 | 2.07629608 | 1.668482066 | 0.300081251 | 0.021535 | 0.346348 |
| Slc24a2 | solute carrier family 24 (sodium/potassium/c | 36.50716149 | 29.38435262 | 0.298770897 | 0.035975 | 0.424092 |
| Unc13a | unc-13 homolog A | 25.60149122 | 20.60477004 | 0.298769306 | 0.012967 | 0.28183 |
| Ddx21 | DEAD (Asp-Glu-Ala-Asp) box polypeptide 21 | 7.383574886 | 5.935485301 | 0.298630489 | 0.005808 | 0.193892 |
| Pak7 | p21 (RAC1) activated kinase 7 | 5.549191171 | 4.462548686 | 0.298618039 | 0.003056 | 0.139048 |
| Myt1l | myelin transcription factor 1-like | 14.24111956 | 11.47884269 | 0.296047994 | 0.011217 | 0.264259 |
| Rbm24 | RNA binding motif protein 24 | 5.214755284 | 4.209532113 | 0.295920537 | 0.044144 | 0.456222 |
| Epc2 | enhancer of polycomb homolog 2 | 10.52534798 | 8.485571079 | 0.295118086 | 0.00527 | 0.182498 |
| Grm3 | glutamate receptor, metabotropic 3 | 17.15857355 | 13.82920121 | 0.294806061 | 0.033878 | 0.411285 |
| Cspg4 | chondroitin sulfate proteoglycan 4 | 2.761253582 | 2.226722566 | 0.294604168 | 0.049049 | 0.47636 |
| Homer1 | homer scaffolding protein 1 | 10.79983799 | 8.71148836 | 0.29412205 | 0.04256 | 0.449801 |
| AI593442 | expressed sequence AI593442 | 27.85155189 | 22.50580621 | 0.29410961 | 0.013433 | 0.287016 |
| Enox2 | ecto-NOX disulfide-thiol exchanger 2 | 5.254999305 | 4.240965204 | 0.293903139 | 0.033702 | 0.411285 |
| Rps6ka3 | ribosomal protein S6 kinase polypeptide 3 | 4.890661885 | 3.945020949 | 0.293879628 | 0.014072 | 0.290117 |
| Rnf2 | ring finger protein 2 | 10.90243512 | 8.815335829 | 0.29295558 | 0.024367 | 0.363714 |
| Tnfrsf19 | tumor necrosis factor receptor superfamily, r | 23.0245193 | 18.59766454 | 0.292888768 | 0.003304 | 0.142987 |
| Med13 | mediator complex subunit 13 | 7.179650006 | 5.797998923 | 0.292482079 | 0.025229 | 0.36904 |
| Carmil1 | capping protein regulator and myosin 1 linke | 5.003363675 | 4.041015456 | 0.292059682 | 0.010696 | 0.25868 |
| Itpr1 | inositol 1,4,5-trisphosphate receptor 1 | 13.84207886 | 11.17515843 | 0.291679593 | 0.02527 | 0.369043 |
| Ncald | neurocalcin delta | 76.51638646 | 61.90897686 | 0.291149593 | 0.004831 | 0.174505 |
| Cog5 | component of oligomeric golgi complex 5 | 6.399870593 | 5.177851969 | 0.290530375 | 0.042892 | 0.450034 |
| Prex1 | phosphatidylinositol-3,4,5-trisphosphate-de | 19.71574289 | 15.93638461 | 0.290098044 | 0.029248 | 0.389312 |
| Nptx1 | neuronal pentraxin 1 | 90.82644312 | 73.55957444 | 0.290018255 | 0.037649 | 0.431765 |
| Sirt1 | sirtuin 1 | 3.027928188 | 2.450889873 | 0.289991829 | 0.035949 | 0.424092 |
| Synpr | synaptoporin | 19.32875658 | 15.67259431 | 0.289439312 | 0.034251 | 0.412986 |
| Rell1 | RELT-like 1 | 5.850367428 | 4.738725759 | 0.289152599 | 0.03424 | 0.412986 |
| Mertk | c-mer proto-oncogene tyrosine kinase | 10.69378247 | 8.661552304 | 0.288981534 | 0.023573 | 0.360702 |
| Arsb | arylsulfatase B | 10.1006313 | 8.173155104 | 0.288954818 | 0.04272 | 0.449801 |

| | | | | | | |
|---|---|---|---|---|---|---|
| Mef2a | myocyte enhancer factor 2A | 12.60228849 | 10.21610308 | 0.288909214 | 0.001859 | 0.107364 |
| Klf13 | Kruppel-like factor 13 | 33.90249777 | 27.46938702 | 0.288288435 | 0.001876 | 0.107364 |
| Rbpj | recombination signal binding protein for imr | 2.943108859 | 2.384733461 | 0.287902609 | 0.012366 | 0.276598 |
| Lin7c | lin-7 homolog C (C. elegans) | 32.034445 | 25.98111492 | 0.287888709 | 0.007058 | 0.212654 |
| Sp4 | trans-acting transcription factor 4 | 3.593160741 | 2.909803945 | 0.287858328 | 0.032016 | 0.405418 |
| Map3k3 | mitogen-activated protein kinase kinase kina | 6.784992552 | 5.500520192 | 0.28767733 | 0.008224 | 0.227225 |
| Gnaq | guanine nucleotide binding protein, alpha q | 43.18623597 | 35.01825218 | 0.287617526 | 0.015484 | 0.30598 |
| Tmtc3 | transmembrane and tetratricopeptide repeat | 1.755440747 | 1.422991168 | 0.28629052 | 0.047954 | 0.474116 |
| Ankhd1 | ankyrin repeat and KH domain containing 1 | 3.987242843 | 3.233402989 | 0.286148935 | 0.027371 | 0.379505 |
| Lrrc4 | leucine rich repeat containing 4 | 26.89592417 | 21.85539063 | 0.28574867 | 0.012912 | 0.281812 |
| Zfp324 | zinc finger protein 324 | 2.655061195 | 2.152569488 | 0.285631057 | 0.038534 | 0.435404 |
| Tanc2 | tetratricopeptide repeat, ankyrin repeat and | 7.496976251 | 6.084433752 | 0.285551164 | 0.021088 | 0.343188 |
| Arfgef3 | ARFGEF family member 3 | 8.314266662 | 6.755938567 | 0.284757116 | 0.030034 | 0.393614 |
| Tgs1 | trimethylguanosine synthase 1 | 6.404278148 | 5.205808206 | 0.284448531 | 0.004558 | 0.169288 |
| Tmem178 | transmembrane protein 178 | 38.04455661 | 30.92290183 | 0.2842377 | 0.027169 | 0.37798 |
| Prdm2 | PR domain containing 2, with ZNF domain | 10.99333884 | 8.934359525 | 0.28387483 | 0.033199 | 0.411061 |
| Pde8b | phosphodiesterase 8B | 9.922769824 | 8.0644562 | 0.283451747 | 0.005345 | 0.184391 |
| Dip2c | disco interacting protein 2 homolog C | 11.07570858 | 9.005731785 | 0.283084737 | 0.009421 | 0.243136 |
| Zfp251 | zinc finger protein 251 | 18.94373302 | 15.41295807 | 0.282795999 | 0.00919 | 0.238232 |
| Limd2 | LIM domain containing 2 | 30.18133519 | 24.58795136 | 0.282722224 | 0.026562 | 0.375461 |
| Cpeb4 | cytoplasmic polyadenylation element bindir | 19.65550474 | 16.00095795 | 0.282434062 | 0.009085 | 0.237092 |
| Mast4 | microtubule associated serine/threonine kin | 1.421138795 | 1.155126346 | 0.282232703 | 0.037851 | 0.432513 |
| Top1 | topoisomerase (DNA) I | 10.09599687 | 8.214750418 | 0.281973914 | 0.018936 | 0.327826 |
| Nbea | neurobeachin | 8.134625794 | 6.622524136 | 0.281708978 | 0.025563 | 0.37117 |
| Ddx19a | DEAD (Asp-Glu-Ala-Asp) box polypeptide 19a | 10.79883636 | 8.789848181 | 0.281477149 | 0.003268 | 0.142987 |
| Prdm10 | PR domain containing 10 | 2.054812674 | 1.674861066 | 0.280916955 | 0.034517 | 0.414161 |
| Fsd1l | fibronectin type III and SPRY domain contain | 3.293090317 | 2.682712682 | 0.279919321 | 0.01172 | 0.270195 |
| Fbxo34 | F-box protein 34 | 17.0828745 | 13.93424089 | 0.279690572 | 0.028168 | 0.382668 |
| Htra1 | HtrA serine peptidase 1 | 90.43529129 | 73.78164874 | 0.279236748 | 0.010833 | 0.260161 |
| Slitrk3 | SLIT and NTRK-like family, member 3 | 13.90911378 | 11.35006164 | 0.279218194 | 0.016205 | 0.31191 |
| Rgs7bp | regulator of G-protein signalling 7 binding pr | 46.91475516 | 38.28392095 | 0.279074829 | 0.003987 | 0.161189 |
| Hcfc2 | host cell factor C2 | 7.288041082 | 5.944299959 | 0.279022715 | 0.032166 | 0.406386 |
| Mn1 | meningioma 1 | 3.738910578 | 3.05144726 | 0.278346317 | 0.018551 | 0.326346 |
| Xpr1 | xenotropic and polytropic retrovirus recepto | 8.498652019 | 6.93366737 | 0.278046361 | 0.01061 | 0.258322 |
| 2700081O | RIKEN cDNA 2700081O15 gene | 14.91151618 | 12.17571953 | 0.277455839 | 0.012918 | 0.281812 |
| Lnpep | leucyl/cystinyl aminopeptidase | 7.883555162 | 6.435009009 | 0.277196959 | 0.020978 | 0.342388 |
| Nckap1 | NCK-associated protein 1 | 152.417709 | 124.5756556 | 0.277077896 | 0.015329 | 0.304034 |
| Abhd17c | abhydrolase domain containing 17C | 13.95605633 | 11.4145467 | 0.276291013 | 0.026006 | 0.372998 |
| Dtna | dystrobrevin alpha | 10.57412337 | 8.642645493 | 0.276055005 | 0.000109 | 0.018431 |

| | | | | | | |
|---|---|---|---|---|---|---|
| Kat6b | K(lysine) acetyltransferase 6B | 2.73723655 | 2.236380638 | 0.276031217 | 0.025508 | 0.370675 |
| Kdm2b | lysine (K)-specific demethylase 2B | 2.677103616 | 2.186494257 | 0.27545571 | 0.041576 | 0.449122 |
| Gpr158 | G protein-coupled receptor 158 | 13.68161427 | 11.19416656 | 0.275014901 | 0.033935 | 0.411285 |
| Pcsk2 | proprotein convertase subtilisin/kexin type 2 | 29.78417458 | 24.38140144 | 0.274872701 | 0.023413 | 0.360229 |
| 2610021A0 | RIKEN cDNA 2610021A01 gene | 3.51604251 | 2.878270742 | 0.274542895 | 0.037409 | 0.430148 |
| Isg20l2 | interferon stimulated exonuclease gene 20-li | 6.350900903 | 5.199611872 | 0.274324703 | 0.041716 | 0.449122 |
| Zfp266 | zinc finger protein 266 | 7.22600615 | 5.914134644 | 0.273907332 | 0.024175 | 0.363714 |
| Cdkn1b | cyclin-dependent kinase inhibitor 1B | 15.01433844 | 12.28793161 | 0.273868657 | 0.000899 | 0.06839 |
| Kdm5b | lysine (K)-specific demethylase 5B | 4.313435998 | 3.530577 | 0.273725426 | 0.032849 | 0.410953 |
| Zkscan8 | zinc finger with KRAB and SCAN domains 8 | 2.382999742 | 1.949261761 | 0.273656776 | 0.044155 | 0.456222 |
| Pou2f2 | POU domain, class 2, transcription factor 2 | 1.424558392 | 1.168247042 | 0.273349483 | 0.043742 | 0.454367 |
| Cnot6 | CCR4-NOT transcription complex, subunit 6 | 12.50410507 | 10.23875291 | 0.273053025 | 0.00435 | 0.165947 |
| Tmem204 | transmembrane protein 204 | 7.312372931 | 5.993092681 | 0.272892364 | 0.030604 | 0.397591 |
| Zfpm1 | zinc finger protein, multitype 1 | 14.10121737 | 11.55981917 | 0.272397968 | 0.012796 | 0.281232 |
| Trib2 | tribbles pseudokinase 2 | 16.07518527 | 13.16690322 | 0.272355029 | 0.019743 | 0.333335 |
| Slc2a13 | solute carrier family 2 (facilitated glucose tra | 12.24208154 | 10.04168761 | 0.271794382 | 0.009216 | 0.238545 |
| Arnt | aryl hydrocarbon receptor nuclear translocat | 3.612661694 | 2.960755933 | 0.271763432 | 0.025409 | 0.36985 |
| 9330159F1 | RIKEN cDNA 9330159F19 gene | 13.15142342 | 10.78763443 | 0.271674195 | 0.038724 | 0.43587 |
| Nrn1 | neuritin 1 | 57.47970459 | 47.16759932 | 0.271290157 | 0.0154 | 0.304655 |
| Kbtbd7 | kelch repeat and BTB (POZ) domain containir | 12.5318217 | 10.27941574 | 0.271068488 | 0.004898 | 0.175838 |
| Fbxo7 | F-box protein 7 | 8.3985603 | 6.892051456 | 0.270657816 | 0.026215 | 0.373263 |
| Cacna2d1 | calcium channel, voltage-dependent, alpha2 | 13.92740166 | 11.43343785 | 0.270569832 | 0.011277 | 0.264259 |
| Ppp3r1 | protein phosphatase 3, regulatory subunit B, | 318.1742437 | 261.4101347 | 0.270365096 | 0.031566 | 0.403493 |
| Sipa1l2 | signal-induced proliferation-associated 1 like | 9.134250454 | 7.494081315 | 0.270207859 | 0.026633 | 0.375863 |
| Rnf19a | ring finger protein 19A | 12.25280328 | 10.05613761 | 0.270040996 | 0.011903 | 0.271429 |
| Gpr155 | G protein-coupled receptor 155 | 10.9866352 | 9.019244552 | 0.269924653 | 0.005216 | 0.181853 |
| Tlr3 | toll-like receptor 3 | 3.608468015 | 2.964256743 | 0.269555785 | 0.034539 | 0.414161 |
| Foxo3 | forkhead box O3 | 11.09928382 | 9.111106986 | 0.268911877 | 0.008782 | 0.234897 |
| Cdk19 | cyclin-dependent kinase 19 | 16.68156304 | 13.69410825 | 0.268896025 | 0.012452 | 0.277167 |
| Sfpq | splicing factor proline/glutamine rich (polyp | 36.02870704 | 29.60625081 | 0.268486232 | 0.013936 | 0.289976 |
| Zfp148 | zinc finger protein 148 | 9.96213816 | 8.184876824 | 0.268469919 | 0.00874 | 0.234495 |
| Igf1r | insulin-like growth factor I receptor | 3.911440121 | 3.211164893 | 0.268402238 | 0.010713 | 0.25868 |
| Kcnd2 | potassium voltage-gated channel, Shal-relate | 24.44684825 | 20.11749664 | 0.268137395 | 0.02566 | 0.371663 |
| Ncoa1 | nuclear receptor coactivator 1 | 14.94935874 | 12.286521 | 0.268120271 | 0.039031 | 0.437099 |
| Cpeb1 | cytoplasmic polyadenylation element bindir | 12.43281779 | 10.22606275 | 0.268113748 | 0.030025 | 0.393614 |
| Bmpr2 | bone morphogenetic protein receptor, type I | 10.9471982 | 8.996273815 | 0.267802211 | 0.004296 | 0.165686 |
| Hivep2 | human immunodeficiency virus type I enhan | 18.54203385 | 15.2322187 | 0.267723666 | 0.019384 | 0.330205 |
| N4bp2l2 | NEDD4 binding protein 2-like 2 | 2.197728999 | 1.80575421 | 0.26763346 | 0.014047 | 0.289976 |
| Dnajb5 | DnaJ heat shock protein family (Hsp40) mem | 27.91917077 | 22.97944361 | 0.267178484 | 0.041621 | 0.449122 |

| | | | | | | |
|---|---|---|---|---|---|---|
| Ier5 | immediate early response 5 | 18.63699998 | 15.32719325 | 0.267067275 | 0.021294 | 0.344035 |
| Ssh2 | slingshot protein phosphatase 2 | 2.518360783 | 2.069843115 | 0.2666886 | 0.016526 | 0.31404 |
| Cdc42ep4 | CDC42 effector protein (Rho GTPase binding) | 23.56318005 | 19.37008541 | 0.266685885 | 0.019112 | 0.328038 |
| Camk1d | calcium/calmodulin-dependent protein kina | 27.36325749 | 22.51887382 | 0.266460481 | 0.015805 | 0.307705 |
| Acvr1 | activin A receptor, type 1 | 8.307154007 | 6.838844519 | 0.266239075 | 0.045678 | 0.463803 |
| Washc4 | WASH complex subunit 4 | 7.903606261 | 6.499492201 | 0.266074039 | 0.021884 | 0.34972 |
| Olfm1 | olfactomedin 1 | 340.9296895 | 280.934864 | 0.26567064 | 0.02757 | 0.379964 |
| Pum1 | pumilio RNA-binding family member 1 | 16.94630309 | 13.95247614 | 0.265365615 | 0.03145 | 0.402589 |
| Itsn2 | intersectin 2 | 4.862540697 | 4.004315792 | 0.265135345 | 0.003126 | 0.139645 |
| Usp38 | ubiquitin specific peptidase 38 | 5.837595867 | 4.809310512 | 0.265123438 | 0.031889 | 0.404373 |
| Lrrc57 | leucine rich repeat containing 57 | 18.36508551 | 15.13419927 | 0.264986353 | 0.02711 | 0.377755 |
| Fkbp5 | FK506 binding protein 5 | 7.260556201 | 5.982109118 | 0.264905735 | 0.033659 | 0.411285 |
| Fam84a | family with sequence similarity 84, member / | 24.49162137 | 20.19407434 | 0.26470749 | 0.030604 | 0.397591 |
| Nrip1 | nuclear receptor interacting protein 1 | 3.704399376 | 3.053001619 | 0.264642997 | 0.022933 | 0.357433 |
| Dopey2 | dopey family member 2 | 8.290258488 | 6.825076793 | 0.264314938 | 0.011228 | 0.264259 |
| Arntl | aryl hydrocarbon receptor nuclear transloca | 2.915848615 | 2.405144736 | 0.264003659 | 0.048403 | 0.475326 |
| Atp8a1 | ATPase, aminophospholipid transporter (APL | 14.85546303 | 12.24642588 | 0.263943736 | 0.028018 | 0.382242 |
| Cttnbp2 | cortactin binding protein 2 | 13.80186592 | 11.38711363 | 0.263796013 | 0.047413 | 0.47273 |
| Pgm2l1 | phosphoglucomutase 2-like 1 | 42.90695288 | 35.37062975 | 0.263661595 | 0.02757 | 0.379964 |
| Hunk | hormonally upregulated Neu-associated kina | 3.962107863 | 3.271231132 | 0.262981914 | 0.031675 | 0.404297 |
| Ppm1l | protein phosphatase 1 (formerly 2C)-like | 11.93128774 | 9.839849236 | 0.26256399 | 0.031385 | 0.402589 |
| Dhx29 | DEAH (Asp-Glu-Ala-His) box polypeptide 29 | 5.231931931 | 4.311999832 | 0.262562584 | 0.040434 | 0.444433 |
| Arhgap12 | Rho GTPase activating protein 12 | 8.714162873 | 7.193062561 | 0.262524495 | 0.025974 | 0.372836 |
| Rsf1 | remodeling and spacing factor 1 | 3.131370471 | 2.582263347 | 0.262400774 | 0.008951 | 0.236504 |
| Rapgefl1 | Rap guanine nucleotide exchange factor (GEF | 38.65312494 | 31.91877859 | 0.261999659 | 0.020306 | 0.336853 |
| Vps37b | vacuolar protein sorting 37B | 9.352160722 | 7.714325012 | 0.261840451 | 0.039893 | 0.442148 |
| Lrrc8b | leucine rich repeat containing 8 family, mem | 18.4376451 | 15.2156082 | 0.261796253 | 0.002386 | 0.12122 |
| Rras2 | related RAS viral (r-ras) oncogene 2 | 16.67358071 | 13.76206721 | 0.261749894 | 0.013823 | 0.289375 |
| D16Ertd47 | DNA segment, Chr 16, ERATO Doi 472, expres | 3.107265383 | 2.56182795 | 0.26160985 | 0.043454 | 0.452964 |
| Nfya | nuclear transcription factor-Y alpha | 4.810118069 | 3.971773858 | 0.260800613 | 0.029875 | 0.3931 |
| Btbd9 | BTB (POZ) domain containing 9 | 19.28939049 | 15.9395032 | 0.260364614 | 0.005973 | 0.19607 |
| Topbp1 | topoisomerase (DNA) II binding protein 1 | 2.433195296 | 2.009688789 | 0.259730726 | 0.036193 | 0.424258 |
| Panx1 | pannexin 1 | 5.289373373 | 4.376471913 | 0.259524214 | 0.025008 | 0.36754 |
| Pde4dip | phosphodiesterase 4D interacting protein (m | 11.90697583 | 9.841863494 | 0.259366398 | 0.043575 | 0.453419 |
| Scfd2 | Sec1 family domain containing 2 | 2.372364592 | 1.960145324 | 0.259118142 | 0.028287 | 0.383852 |
| Dlg3 | discs large MAGUK scaffold protein 3 | 37.79070995 | 31.28111961 | 0.25890848 | 0.032506 | 0.408399 |
| Spred2 | sprouty-related, EVH1 domain containing 2 | 22.50560077 | 18.61172481 | 0.258692114 | 0.017602 | 0.318417 |
| Dpysl5 | dihydropyrimidinase-like 5 | 8.517949235 | 7.045842547 | 0.258550677 | 0.04151 | 0.449122 |
| Siah1a | siah E3 ubiquitin protein ligase 1A | 15.06490094 | 12.46969777 | 0.258439354 | 0.026932 | 0.376476 |

| | | | | | | |
|---|---|---|---|---|---|---|
| Acap2 | ArfGAP with coiled-coil, ankyrin repeat and P | 20.08937138 | 16.62116024 | 0.258355437 | 0.015355 | 0.304124 |
| Ighm | immunoglobulin heavy constant mu | 8.062234011 | 6.675552247 | 0.257968447 | 0.042037 | 0.449674 |
| Ermp1 | endoplasmic reticulum metallopeptidase 1 | 10.86056697 | 8.991220924 | 0.257741437 | 0.011737 | 0.270195 |
| Dgat2 | diacylglycerol O-acyltransferase 2 | 38.5877718 | 31.96767668 | 0.257720221 | 0.021713 | 0.348581 |
| Ubash3b | ubiquitin associated and SH3 domain contai | 5.278552525 | 4.369141829 | 0.257577741 | 0.005919 | 0.19607 |
| Wdr5 | WD repeat domain 5 | 10.24826483 | 8.489681854 | 0.257002996 | 0.005365 | 0.184709 |
| Zfp84 | zinc finger protein 84 | 4.70794086 | 3.897362466 | 0.256958796 | 0.049014 | 0.476275 |
| Jmjd1c | jumonji domain containing 1C | 4.15377193 | 3.440749536 | 0.256478586 | 0.039773 | 0.441913 |
| Slitrk5 | SLIT and NTRK-like family, member 5 | 14.18421204 | 11.76076293 | 0.256160067 | 0.027087 | 0.377734 |
| Med13l | mediator complex subunit 13-like | 5.267200134 | 4.361739461 | 0.255922109 | 0.018642 | 0.326981 |
| Nebl | nebulette | 7.88912838 | 6.537443209 | 0.255316075 | 0.029992 | 0.393614 |
| Alg10b | asparagine-linked glycosylation 10B (alpha-1 | 9.126073547 | 7.568142768 | 0.254927302 | 0.008194 | 0.227225 |
| Foxn2 | forkhead box N2 | 4.360927018 | 3.618444692 | 0.254861217 | 0.022503 | 0.354158 |
| Nudt10 | nudix (nucleoside diphosphate linked moiety | 17.02929802 | 14.13312701 | 0.254497833 | 0.037564 | 0.431368 |
| Smad4 | SMAD family member 4 | 11.17520944 | 9.262613413 | 0.254379088 | 0.030153 | 0.394834 |
| Zbtb1 | zinc finger and BTB domain containing 1 | 2.33175616 | 1.933419655 | 0.253903421 | 0.022244 | 0.352282 |
| Lrrc8c | leucine rich repeat containing 8 family, mem | 4.778435372 | 3.961165023 | 0.253791962 | 0.033679 | 0.411285 |
| Acvr2a | activin receptor IIA | 9.755525694 | 8.101403433 | 0.25364037 | 0.014805 | 0.298243 |
| Gatad2b | GATA zinc finger domain containing 2B | 7.836033134 | 6.507983617 | 0.253492341 | 0.012187 | 0.274713 |
| Gpsm2 | G-protein signalling modulator 2 (AGS3-like, | 6.32170761 | 5.249921162 | 0.252332212 | 0.034916 | 0.415856 |
| Fbxl18 | F-box and leucine-rich repeat protein 18 | 5.496930821 | 4.562608453 | 0.252044445 | 0.034829 | 0.415377 |
| Tdg | thymine DNA glycosylase | 11.09886777 | 9.231588255 | 0.251287708 | 0.025308 | 0.36929 |
| Zbtb41 | zinc finger and BTB domain containing 41 | 4.45504392 | 3.70131303 | 0.251062065 | 0.023516 | 0.36066 |
| Ncoa6 | nuclear receptor coactivator 6 | 6.251073985 | 5.199378168 | 0.250554551 | 0.007038 | 0.212654 |
| Smurf2 | SMAD specific E3 ubiquitin protein ligase 2 | 9.022536436 | 7.502858872 | 0.250352607 | 0.012482 | 0.277167 |
| Rbfox3 | RNA binding protein, fox-1 homolog (C. elega | 43.17082736 | 35.95785316 | 0.249884837 | 0.042333 | 0.449801 |
| Calb1 | calbindin 1 | 28.58403403 | 23.80662405 | 0.249869421 | 0.023412 | 0.360229 |
| Sema6d | sema domain, transmembrane domain (TM), | 8.932697125 | 7.433065158 | 0.249569172 | 0.026297 | 0.373273 |
| Otub2 | OTU domain, ubiquitin aldehyde binding 2 | 17.32187758 | 14.43603845 | 0.248688237 | 0.035043 | 0.417091 |
| Ss18l1 | SS18, nBAF chromatin remodeling complex s | 12.78130329 | 10.64258259 | 0.248683235 | 0.018278 | 0.323798 |
| Smpd3 | sphingomyelin phosphodiesterase 3, neutral | 22.31261328 | 18.58464293 | 0.248659242 | 0.040619 | 0.445273 |
| Lats2 | large tumor suppressor 2 | 5.737593458 | 4.78117825 | 0.248320295 | 0.048738 | 0.475516 |
| Brinp1 | bone morphogenic protein/retinoic acid ind | 33.19085017 | 27.67534415 | 0.248120446 | 0.047267 | 0.47273 |
| Magi2 | membrane associated guanylate kinase, WW | 8.484478472 | 7.066101158 | 0.24801534 | 0.042818 | 0.449801 |
| Grm7 | glutamate receptor, metabotropic 7 | 7.441310741 | 6.198158597 | 0.247941376 | 0.046208 | 0.468112 |
| Mbtps2 | membrane-bound transcription factor pepti | 3.219143733 | 2.684974594 | 0.246842481 | 0.04809 | 0.474629 |
| Fam160b1 | family with sequence similarity 160, membe | 6.516103408 | 5.432819416 | 0.246841947 | 0.041029 | 0.447817 |
| Akt3 | thymoma viral proto-oncogene 3 | 41.08163773 | 34.25737926 | 0.246544957 | 0.004517 | 0.169034 |
| Mycbp2 | MYC binding protein 2, E3 ubiquitin protein | 12.7671054 | 10.65394573 | 0.24570607 | 0.033844 | 0.411285 |

| | | | | | | |
|---|---|---|---|---|---|---|
| Abcb7 | ATP-binding cassette, sub-family B (MDR/TAP | 8.829935028 | 7.367022247 | 0.245699077 | 0.022929 | 0.357433 |
| Ncor1 | nuclear receptor co-repressor 1 | 16.77674691 | 13.9953041 | 0.245641393 | 0.019395 | 0.330205 |
| Zik1 | zinc finger protein interacting with K protein | 3.459467134 | 2.89084464 | 0.244826588 | 0.047555 | 0.472936 |
| Zfp212 | Zinc finger protein 212 | 6.04214977 | 5.04584689 | 0.244407628 | 0.034738 | 0.414579 |
| Desi2 | desumoylating isopeptidase 2 | 5.118391327 | 4.276512262 | 0.244240911 | 0.021753 | 0.348898 |
| Abcb10 | ATP-binding cassette, sub-family B (MDR/TAP | 10.36727074 | 8.668227382 | 0.243605566 | 0.039717 | 0.441694 |
| Slf2 | SMC5-SMC6 complex localization factor 2 | 6.47224181 | 5.415140127 | 0.243373764 | 0.03191 | 0.404373 |
| Iqsec2 | IQ motif and Sec7 domain 2 | 36.64864386 | 30.64030272 | 0.243175149 | 0.020108 | 0.335484 |
| Tnks2 | tankyrase, TRF1-interacting ankyrin-related A | 20.11758465 | 16.82445572 | 0.242916591 | 0.013697 | 0.288529 |
| Pwwp2a | PWWP domain containing 2A | 6.150201677 | 5.144372385 | 0.242896533 | 0.024721 | 0.365666 |
| Abi1 | abl-interactor 1 | 47.438858 | 39.70853575 | 0.242845888 | 0.029631 | 0.391827 |
| Pou3f3 | POU domain, class 3, transcription factor 3 | 27.82734913 | 23.27720428 | 0.242818488 | 0.007865 | 0.22354 |
| Ino80d | INO80 complex subunit D | 1.840731926 | 1.538104237 | 0.242787372 | 0.033371 | 0.411285 |
| Zyg11b | zyg-ll family member B, cell cycle regulator | 24.34701376 | 20.36489713 | 0.242493891 | 0.038391 | 0.434618 |
| Sort1 | sortilin 1 | 70.31235174 | 58.85118853 | 0.242332024 | 0.019893 | 0.334598 |
| Ppp4r2 | protein phosphatase 4, regulatory subunit 2 | 13.05705526 | 10.92478253 | 0.242041597 | 0.008038 | 0.225748 |
| Abcd2 | ATP-binding cassette, sub-family D (ALD), mei | 9.59353175 | 8.028003479 | 0.241985363 | 0.025379 | 0.369715 |
| Nectin3 | nectin cell adhesion molecule 3 | 3.557521765 | 2.977158622 | 0.241898168 | 0.010203 | 0.252958 |
| Syncrip | synaptotagmin binding, cytoplasmic RNA int | 8.737730916 | 7.313326967 | 0.241693296 | 0.004715 | 0.172429 |
| Kdm4c | lysine (K)-specific demethylase 4C | 5.185946007 | 4.340539963 | 0.241447952 | 0.044935 | 0.461002 |
| Map9 | microtubule-associated protein 9 | 15.86130872 | 13.29418999 | 0.240180748 | 0.047648 | 0.472936 |
| Nfix | nuclear factor I/X | 44.2523484 | 37.04512252 | 0.240043221 | 0.043752 | 0.454367 |
| Myo9a | myosin IXa | 6.110413072 | 5.118241097 | 0.239987276 | 0.02344 | 0.360229 |
| Abhd13 | abhydrolase domain containing 13 | 11.00271525 | 9.224075936 | 0.23994384 | 0.008431 | 0.231481 |
| Tspan5 | tetraspanin 5 | 24.93474976 | 20.91498752 | 0.239915523 | 0.026187 | 0.373263 |
| Nrxn1 | neurexin I | 20.53615612 | 17.21173743 | 0.239818909 | 0.004363 | 0.165947 |
| Mllt3 | myeloid/lymphoid or mixed-lineage leukemi | 2.741212392 | 2.299839791 | 0.238738341 | 0.04718 | 0.47273 |
| Dcp1a | decapping mRNA 1A | 3.047712194 | 2.55772427 | 0.238543857 | 0.031022 | 0.400448 |
| Gng2 | guanine nucleotide binding protein (G protei | 44.01646773 | 36.94626119 | 0.238370015 | 0.023302 | 0.360229 |
| Msl2 | MSL complex subunit 2 | 10.77050704 | 9.041119095 | 0.238261092 | 0.011258 | 0.264259 |
| Lrrc58 | leucine rich repeat containing 58 | 16.40426155 | 13.75826621 | 0.238160511 | 0.005895 | 0.195705 |
| Frmd4a | FERM domain containing 4A | 4.197861881 | 3.520979374 | 0.237828556 | 0.046942 | 0.471749 |
| Mgat4a | mannoside acetylglucosaminyltransferase 4, | 3.08081562 | 2.585440642 | 0.237605104 | 0.036738 | 0.426602 |
| Klf3 | Kruppel-like factor 3 (basic) | 4.451176022 | 3.737557469 | 0.23759824 | 0.005626 | 0.189963 |
| Nrep | neuronal regeneration related protein | 76.22224968 | 64.01385692 | 0.237205161 | 0.017729 | 0.318884 |
| Slmap | sarcolemma associated protein | 13.45930708 | 11.29398054 | 0.237140421 | 0.017512 | 0.318417 |
| Fam222b | family with sequence similarity 222, membe | 10.11053721 | 8.491741509 | 0.236617738 | 0.04264 | 0.449801 |
| Stat3 | signal transducer and activator of transcripti | 6.243109108 | 5.242308191 | 0.236574826 | 0.027085 | 0.377734 |
| Fam49a | family with sequence similarity 49, member A | 17.79648803 | 14.9617577 | 0.236519424 | 0.034084 | 0.412355 |

| | | | | | | |
|---|---|---|---|---|---|---|
| Tmem170b | transmembrane protein 170B | 12.4003659 | 10.42498911 | 0.2353329 | 0.002638 | 0.127172 |
| Bmi1 | Bmi1 polycomb ring finger oncogene | 15.83177649 | 13.32647343 | 0.234854737 | 0.009173 | 0.238131 |
| Mib1 | mindbomb E3 ubiquitin protein ligase 1 | 20.60523326 | 17.32621593 | 0.23465901 | 0.016291 | 0.312811 |
| Frmpd4 | FERM and PDZ domain containing 4 | 7.61214254 | 6.400988586 | 0.234065802 | 0.044389 | 0.457039 |
| Epn2 | epsin 2 | 36.95784795 | 31.14891155 | 0.232329814 | 0.008889 | 0.236504 |
| Fbxw11 | F-box and WD-40 domain protein 11 | 43.84451956 | 36.95628626 | 0.231789741 | 0.006165 | 0.199751 |
| Fam171b | family with sequence similarity 171, member | 66.17483986 | 55.79299515 | 0.231383608 | 0.008035 | 0.225748 |
| Zfp281 | zinc finger protein 281 | 10.50625223 | 8.868562376 | 0.229998904 | 0.042609 | 0.449801 |
| Tnks | tankyrase, TRF1-interacting ankyrin-related A | 8.754675294 | 7.387889974 | 0.229441707 | 0.044293 | 0.456743 |
| Syt7 | synaptotagmin VII | 49.32235233 | 41.64049906 | 0.229303838 | 0.009607 | 0.244306 |
| Tsr1 | TSR1 20S rRNA accumulation | 10.48056634 | 8.851623087 | 0.228867734 | 0.029352 | 0.389892 |
| Hnrnpa3 | heterogeneous nuclear ribonucleoprotein A3 | 54.08989642 | 45.70022875 | 0.228392847 | 0.000289 | 0.034695 |
| Zfp704 | zinc finger protein 704 | 5.45097694 | 4.602697628 | 0.228030957 | 0.041521 | 0.449122 |
| Dgkd | diacylglycerol kinase, delta | 21.58093383 | 18.24503334 | 0.22762329 | 0.009779 | 0.24593 |
| Kcnq3 | potassium voltage-gated channel, subfamily ( | 11.6579422 | 9.853916269 | 0.227508347 | 0.039619 | 0.441354 |
| 1700020I1 | RIKEN cDNA 1700020I14 gene | 70.14978243 | 59.25064001 | 0.22746534 | 0.026294 | 0.373273 |
| Ddx46 | DEAD (Asp-Glu-Ala-Asp) box polypeptide 46 | 9.901068182 | 8.371294654 | 0.227210174 | 0.019813 | 0.333567 |
| Glce | glucuronyl C5-epimerase | 4.02686064 | 3.40805975 | 0.226823434 | 0.048669 | 0.475516 |
| Jmy | junction-mediating and regulatory protein | 9.40874966 | 7.955436011 | 0.226329255 | 0.042387 | 0.449801 |
| Zmym2 | zinc finger, MYM-type 2 | 10.22214878 | 8.653714501 | 0.22574315 | 0.027459 | 0.379626 |
| B3galt1 | UDP-Gal:betaGlcNAc beta 1,3-galactosyltran | 9.611877267 | 8.133792097 | 0.225446782 | 0.027192 | 0.378003 |
| Rab11fip2 | RAB11 family interacting protein 2 (class I) | 10.36409233 | 8.774828253 | 0.225280758 | 0.022161 | 0.351612 |
| Vcpip1 | valosin containing protein (p97)/p47 comple | 7.414510752 | 6.277511923 | 0.225167556 | 0.016138 | 0.311239 |
| Camk2n1 | calcium/calmodulin-dependent protein kina | 283.1151201 | 239.7039067 | 0.224524215 | 0.04339 | 0.452562 |
| Atf7ip | activating transcription factor 7 interacting p | 3.770231575 | 3.193604106 | 0.224290701 | 0.026072 | 0.373081 |
| Dyrk1a | dual-specificity tyrosine-(Y)-phosphorylation | 10.27214832 | 8.708804555 | 0.223355253 | 0.008275 | 0.228265 |
| Lrrn3 | leucine rich repeat protein 3, neuronal | 17.48768404 | 14.83475102 | 0.223230665 | 0.026546 | 0.375461 |
| Dlgap1 | DLG associated protein 1 | 34.90397637 | 29.62610348 | 0.22189511 | 0.04546 | 0.462192 |
| Sema7a | sema domain, immunoglobulin domain (Ig), | 48.19218862 | 40.89287342 | 0.221824166 | 0.042612 | 0.449801 |
| Usp1 | ubiquitin specific peptidase 1 | 9.522411534 | 8.083127041 | 0.221708853 | 0.048437 | 0.475326 |
| Ddx6 | DEAD (Asp-Glu-Ala-Asp) box polypeptide 6 | 23.15732082 | 19.66237849 | 0.220821198 | 0.025249 | 0.36904 |
| Celf2 | CUGBP, Elav-like family member 2 | 15.35104182 | 13.04356622 | 0.220439246 | 0.033735 | 0.411285 |
| Ppp3cb | protein phosphatase 3, catalytic subunit, bet | 50.83040223 | 43.21128518 | 0.22039092 | 0.030631 | 0.397591 |
| Dsel | dermatan sulfate epimerase-like | 3.496162912 | 2.970003905 | 0.220336103 | 0.046749 | 0.470345 |
| Fam117b | family with sequence similarity 117, member | 9.646795474 | 8.195512976 | 0.21973399 | 0.03646 | 0.425902 |
| Ythdf3 | YTH domain family 3 | 15.47882228 | 13.16368001 | 0.218914195 | 0.007539 | 0.220393 |
| Mosmo | modulator of smoothened | 8.212717978 | 6.989713471 | 0.218573235 | 0.033061 | 0.411041 |
| Fmr1 | fragile X mental retardation 1 | 8.793829829 | 7.479571102 | 0.218391097 | 0.004184 | 0.163555 |
| Dpysl3 | dihydropyrimidinase-like 3 | 17.7496652 | 15.10220983 | 0.217893707 | 0.026518 | 0.375448 |

| | | | | | | |
|---|---|---|---|---|---|---|
| B230219D2 | RIKEN cDNA B230219D22 gene | 34.61047987 | 29.45851299 | 0.217436153 | 0.035863 | 0.424092 |
| Chm | choroidermia (RAB escort protein 1) | 7.536689235 | 6.418423771 | 0.216970746 | 0.041024 | 0.447817 |
| Elk1 | ELK1, member of ETS oncogene family | 15.19028623 | 12.94714124 | 0.216660071 | 0.046658 | 0.469916 |
| Fbrsl1 | fibrosin-like 1 | 8.962243784 | 7.635875466 | 0.216573742 | 0.031811 | 0.404338 |
| Rragd | Ras-related GTP binding D | 19.83160241 | 16.88950688 | 0.216407245 | 0.047958 | 0.474116 |
| Tmem132b | transmembrane protein 132B | 19.87747937 | 16.92879898 | 0.216262929 | 0.014764 | 0.297981 |
| Gpr85 | G protein-coupled receptor 85 | 9.876271305 | 8.41621962 | 0.216070975 | 0.037227 | 0.429183 |
| Rock2 | Rho-associated coiled-coil containing protei | 13.52961098 | 11.53330196 | 0.215842201 | 0.040443 | 0.444433 |
| Ppp1r12a | protein phosphatase 1, regulatory subunit 1 | 12.11933254 | 10.34055183 | 0.213672391 | 0.046973 | 0.471794 |
| Socs4 | suppressor of cytokine signaling 4 | 3.551897931 | 3.030486492 | 0.213580597 | 0.039636 | 0.441354 |
| Camsap2 | calmodulin regulated spectrin-associated pr | 19.50207041 | 16.6397964 | 0.213502825 | 0.041509 | 0.449122 |
| Amer2 | APC membrane recruitment 2 | 9.068157552 | 7.73489643 | 0.213502589 | 0.031449 | 0.402589 |
| Mef2d | myocyte enhancer factor 2D | 32.70445936 | 27.9087551 | 0.213287112 | 0.025021 | 0.36754 |
| Camkk2 | calcium/calmodulin-dependent protein kina | 17.39052219 | 14.84802541 | 0.213089262 | 0.015008 | 0.300956 |
| Map4k5 | mitogen-activated protein kinase kinase kina | 6.70976016 | 5.732820189 | 0.212941218 | 0.017269 | 0.316103 |
| Vps54 | VPS54 GARP complex subunit | 4.634716844 | 3.959940583 | 0.212693971 | 0.043833 | 0.454496 |
| Wapl | WAPL cohesin release factor | 11.12127896 | 9.498249971 | 0.212618757 | 0.021815 | 0.349401 |
| Agfg1 | ArfGAP with FG repeats 1 | 14.11456395 | 12.05389476 | 0.212427673 | 0.013132 | 0.2831 |
| Sh3gl1 | SH3-domain GRB2-like 1 | 15.11001129 | 12.91349579 | 0.212274377 | 0.040338 | 0.444433 |
| Scarb1 | scavenger receptor class B, member 1 | 4.370070867 | 3.737491091 | 0.212176202 | 0.031715 | 0.404338 |
| Psen1 | presenilin 1 | 14.24247751 | 12.16637086 | 0.212041426 | 0.014161 | 0.290588 |
| Ptpn12 | protein tyrosine phosphatase, non-receptor | 11.32578529 | 9.677067773 | 0.211842331 | 0.049946 | 0.479779 |
| Ust | uronyl-2-sulfotransferase | 19.59232921 | 16.75363642 | 0.21145837 | 0.048433 | 0.475326 |
| Rc3h2 | ring finger and CCCH-type zinc finger domain | 3.703438134 | 3.169044661 | 0.210246574 | 0.022404 | 0.353547 |
| Hexb | hexosaminidase B | 51.82932481 | 44.33322727 | 0.210212105 | 0.033033 | 0.411041 |
| Esyt2 | extended synaptotagmin-like protein 2 | 6.470616332 | 5.534127672 | 0.21017818 | 0.01402 | 0.289976 |
| Ski | ski sarcoma viral oncogene homolog (avian) | 37.25099632 | 31.87359297 | 0.209664994 | 0.007048 | 0.212654 |
| Mapk1ip1l | mitogen-activated protein kinase 1 interacti | 4.664415201 | 3.99197313 | 0.209607403 | 0.043362 | 0.452562 |
| Ppp1cc | protein phosphatase 1 catalytic subunit gam | 35.17080314 | 30.12888588 | 0.209282518 | 0.029516 | 0.390897 |
| Klhl2 | kelch-like 2, Mayven | 37.33265878 | 31.97816618 | 0.20901551 | 0.026141 | 0.373103 |
| Pkig | protein kinase inhibitor, gamma | 17.46693504 | 14.97274514 | 0.208784555 | 0.042 | 0.449674 |
| Megf9 | multiple EGF-like-domains 9 | 21.73755283 | 18.62133466 | 0.208675144 | 0.036445 | 0.425902 |
| Mgll | monoglyceride lipase | 74.9651422 | 64.17589012 | 0.208579634 | 0.027139 | 0.377862 |
| Plxnd1 | plexin D1 | 6.045472234 | 5.175408248 | 0.20854751 | 0.039012 | 0.437099 |
| Asphd2 | aspartate beta-hydroxylase domain containi | 34.00320235 | 29.14106146 | 0.208387884 | 0.034339 | 0.413733 |
| Tril | TLR4 interactor with leucine-rich repeats | 14.96258486 | 12.81618301 | 0.208119532 | 0.03872 | 0.43587 |
| Hcfc1 | host cell factor C1 | 12.3741714 | 10.6057697 | 0.206875788 | 0.036558 | 0.426227 |
| Socs7 | suppressor of cytokine signaling 7 | 20.35929375 | 17.44422986 | 0.206873247 | 0.03318 | 0.411061 |
| Vps26b | VPS26 retromer complex component B | 33.0067823 | 28.32974595 | 0.206051272 | 0.016486 | 0.31404 |

| | | | | | | |
|---|---|---|---|---|---|---|
| Ogfrl1 | opioid growth factor receptor-like 1 | 30.97334651 | 26.58507652 | 0.206009523 | 0.032677 | 0.409368 |
| Nmt1 | N-myristoyltransferase 1 | 43.46606794 | 37.31930346 | 0.205858441 | 0.042374 | 0.449801 |
| 2510009E0 | RIKEN cDNA 2510009E07 gene | 11.86464952 | 10.18594984 | 0.204850947 | 0.025692 | 0.371823 |
| Abr | active BCR-related gene | 67.1079149 | 57.64434221 | 0.204810057 | 0.021319 | 0.344127 |
| Ppp1cb | protein phosphatase 1 catalytic subunit beta | 38.56985682 | 33.14274395 | 0.204420289 | 0.008946 | 0.236504 |
| Kcnj11 | potassium inwardly rectifying channel, subfa | 10.76488662 | 9.254312532 | 0.203867809 | 0.047319 | 0.47273 |
| Sgtb | small glutamine-rich tetratricopeptide repea | 28.601353 | 24.57388272 | 0.203668704 | 0.041358 | 0.449122 |
| B4galt6 | UDP-Gal:betaGlcNAc beta 1,4-galactosyltran | 35.17144521 | 30.24376572 | 0.202978692 | 0.032385 | 0.407454 |
| Trim9 | tripartite motif-containing 9 | 20.95194126 | 18.02585404 | 0.202653751 | 0.041714 | 0.449122 |
| Hipk4 | homeodomain interacting protein kinase 4 | 18.47584723 | 15.88965653 | 0.202648868 | 0.01093 | 0.261775 |
| Uhrf2 | ubiquitin-like, containing PHD and RING fing | 5.772468429 | 4.965395724 | 0.202217304 | 0.036198 | 0.424258 |
| Pik3r1 | phosphoinositide-3-kinase regulatory subuni | 14.35383684 | 12.34299256 | 0.201779833 | 0.048504 | 0.475516 |
| Sf3b1 | splicing factor 3b, subunit 1 | 17.92608781 | 15.43496888 | 0.201591613 | 0.043063 | 0.450751 |
| Prickle1 | prickle planar cell polarity protein 1 | 20.96537928 | 18.05951272 | 0.200492439 | 0.037646 | 0.431765 |
| Usp12 | ubiquitin specific peptidase 12 | 17.31504622 | 14.91464319 | 0.20033767 | 0.019705 | 0.333017 |
| Chst11 | carbohydrate sulfotransferase 11 | 17.3208032 | 14.92914173 | 0.198980572 | 0.026932 | 0.376476 |
| Ulk2 | unc-51 like kinase 2 | 26.4139335 | 22.77109967 | 0.198253222 | 0.036049 | 0.424092 |
| Tsc22d2 | TSC22 domain family, member 2 | 3.860819287 | 3.329884481 | 0.197933677 | 0.046895 | 0.471541 |
| Celf1 | CUGBP, Elav-like family member 1 | 23.07360961 | 19.91302021 | 0.197893152 | 0.041891 | 0.449674 |
| Ankle2 | ankyrin repeat and LEM domain containing 2 | 7.129214306 | 6.149962825 | 0.19765099 | 0.042161 | 0.449801 |
| Rab10 | RAB10, member RAS oncogene family | 69.3600833 | 59.87881725 | 0.197390478 | 0.010701 | 0.25868 |
| Gabra4 | gamma-aminobutyric acid (GABA) A receptor | 18.71392208 | 16.16719261 | 0.197034283 | 0.047152 | 0.47273 |
| Cggbp1 | CGG triplet repeat binding protein 1 | 18.72454978 | 16.17960431 | 0.195217559 | 0.022551 | 0.354592 |
| Qrich1 | glutamine-rich 1 | 9.269147242 | 8.015835648 | 0.195049897 | 0.033722 | 0.411285 |
| Ppp6r3 | protein phosphatase 6, regulatory subunit 3 | 9.636941824 | 8.339639479 | 0.193484658 | 0.032559 | 0.408778 |
| Rmnd5a | required for meiotic nuclear division 5 homo | 26.54474288 | 22.97845527 | 0.19277648 | 0.035628 | 0.422336 |
| Fam3c | family with sequence similarity 3, member C | 21.89026639 | 18.97261421 | 0.191866587 | 0.040615 | 0.445273 |
| Strn3 | striatin, calmodulin binding protein 3 | 11.84070673 | 10.25914685 | 0.191352224 | 0.047703 | 0.472936 |
| Igsf11 | immunoglobulin superfamily, member 11 | 9.38425829 | 8.143926203 | 0.190424717 | 0.039824 | 0.441913 |
| Insr | insulin receptor | 3.934612142 | 3.41139583 | 0.190392957 | 0.043951 | 0.455453 |
| Dclk1 | doublecortin-like kinase 1 | 43.5985308 | 37.82297494 | 0.190002561 | 0.021006 | 0.342524 |
| Kidins220 | kinase D-interacting substrate 220 | 36.80168294 | 31.94430681 | 0.188888738 | 0.040074 | 0.442871 |
| Diras2 | DIRAS family, GTP-binding RAS-like 2 | 66.36556319 | 57.66070639 | 0.187843285 | 0.019157 | 0.328485 |
| Arpp19 | cAMP-regulated phosphoprotein 19 | 24.91709224 | 21.66520075 | 0.186945996 | 0.011765 | 0.270195 |
| Tbc1d20 | TBC1 domain family, member 20 | 20.56876605 | 17.89717144 | 0.185780377 | 0.017081 | 0.315784 |
| Krr1 | KRR1, small subunit (SSU) processome comp | 6.44534426 | 5.608061794 | 0.185700567 | 0.044126 | 0.456222 |
| Usp22 | ubiquitin specific peptidase 22 | 59.81249254 | 52.07939168 | 0.185468903 | 0.049922 | 0.479779 |
| Rnf38 | ring finger protein 38 | 5.959423299 | 5.190950215 | 0.18458488 | 0.040363 | 0.444433 |
| Srsf10 | serine/arginine-rich splicing factor 10 | 12.17101099 | 10.60690918 | 0.183569552 | 0.018894 | 0.327826 |

| | | | | | | |
|---|---|---|---|---|---|---|
| Slc7a5 | solute carrier family 7 (cationic amino acid tr | 38.99969445 | 33.98831423 | 0.183162085 | 0.049198 | 0.477274 |
| Fbxo11 | F-box protein 11 | 35.67182488 | 31.11716256 | 0.182453692 | 0.030901 | 0.399905 |
| Arhgap21 | Rho GTPase activating protein 21 | 20.00623132 | 17.44977472 | 0.182205975 | 0.049696 | 0.47935 |
| Slc35a5 | solute carrier family 35, member A5 | 7.005990515 | 6.111915941 | 0.181934373 | 0.033696 | 0.411285 |
| Tbpl1 | TATA box binding protein-like 1 | 17.09816282 | 14.92458069 | 0.181922115 | 0.039309 | 0.439102 |
| 3-Sep | septin 3 | 91.66093591 | 80.05539334 | 0.180677138 | 0.017463 | 0.318417 |
| Tmcc1 | transmembrane and coiled coil domains 1 | 5.049809842 | 4.416126285 | 0.178781559 | 0.049454 | 0.478698 |
| Rasa3 | RAS p21 protein activator 3 | 16.87457841 | 14.75772086 | 0.177851359 | 0.04808 | 0.474629 |
| Ap3m1 | adaptor-related protein complex 3, mu 1 sub | 10.35802177 | 9.065118057 | 0.177601076 | 0.025807 | 0.372254 |
| Ralgps1 | Ral GEF with PH domain and SH3 binding mo | 9.367693036 | 8.202387177 | 0.176561156 | 0.040487 | 0.444433 |
| Ppm1h | protein phosphatase 1H (PP2C domain conta | 9.066591908 | 7.941230351 | 0.176213397 | 0.020687 | 0.339596 |
| Atp2c1 | ATPase, Ca++-sequestering | 18.3928001 | 16.11664293 | 0.176001818 | 0.038485 | 0.43513 |
| Med1 | mediator complex subunit 1 | 4.716060976 | 4.131702635 | 0.175954884 | 0.040492 | 0.444433 |
| Arih1 | ariadne RBR E3 ubiquitin protein ligase 1 | 14.05115652 | 12.33200872 | 0.173416053 | 0.026814 | 0.376367 |
| Mta2 | metastasis-associated gene family, member 2 | 13.66135686 | 11.998056 | 0.172563776 | 0.037114 | 0.428712 |
| Ybx1 | Y box protein 1 | 110.9749301 | 97.48158576 | 0.172264395 | 0.038387 | 0.434618 |
| Dcun1d1 | DCN1, defective in cullin neddylation 1, dom | 6.831647949 | 6.000713906 | 0.172042752 | 0.03929 | 0.439102 |
| Clcn3 | chloride channel, voltage-sensitive 3 | 11.52210626 | 10.12003275 | 0.172008345 | 0.037942 | 0.432603 |
| Marcks | myristoylated alanine rich protein kinase C su | 88.17896513 | 77.49146835 | 0.171473668 | 0.046653 | 0.469916 |
| Tcea1 | transcription elongation factor A (SII) 1 | 22.29237212 | 19.59278577 | 0.171109981 | 0.041981 | 0.449674 |
| Ss18 | SS18, nBAF chromatin remodeling complex s | 12.92198516 | 11.37147714 | 0.169342047 | 0.042549 | 0.449801 |
| Extl2 | exostoses (multiple)-like 2 | 25.5891397 | 22.56213492 | 0.167397676 | 0.048598 | 0.475516 |
| Clcn4 | chloride channel, voltage-sensitive 4 | 30.53267609 | 26.92875923 | 0.166703165 | 0.027399 | 0.379505 |
| Spin1 | spindlin 1 | 47.5604516 | 41.95546194 | 0.165835024 | 0.03245 | 0.40799 |
| Wdr26 | WD repeat domain 26 | 22.85344212 | 20.18251991 | 0.164488088 | 0.045425 | 0.462192 |
| Mcl1 | myeloid cell leukemia sequence 1 | 33.2935748 | 29.40194842 | 0.164380325 | 0.037228 | 0.429183 |
| Csnk2a1 | casein kinase 2, alpha 1 polypeptide | 17.88748502 | 15.81389717 | 0.163535697 | 0.046552 | 0.469916 |
| Crk | v-crk avian sarcoma virus CT10 oncogene hor | 39.31786505 | 34.74993368 | 0.162940415 | 0.043052 | 0.450751 |
| Fut8 | fucosyltransferase 8 | 11.11415711 | 9.826706844 | 0.162660996 | 0.048445 | 0.475326 |
| Arf4 | ADP-ribosylation factor 4 | 38.51301326 | 34.10547675 | 0.16062107 | 0.037773 | 0.432069 |
| Ppp2r5c | protein phosphatase 2, regulatory subunit B' | 29.56545042 | 26.18468747 | 0.160329536 | 0.016082 | 0.310485 |
| Tapt1 | transmembrane anterior posterior transform | 13.88535215 | 12.31542947 | 0.158661002 | 0.040759 | 0.446526 |
| Tlk1 | tousled-like kinase 1 | 12.92908434 | 11.46538567 | 0.15858571 | 0.04263 | 0.449801 |
| Api5 | apoptosis inhibitor 5 | 24.84629395 | 22.14789386 | 0.150524505 | 0.044547 | 0.458398 |
| Atp5b | ATP synthase, H+ transporting mitochondrial | 248.1275316 | 275.5931928 | -0.166489918 | 0.033065 | 0.411041 |
| Limk2 | LIM motif-containing protein kinase 2 | 14.55345822 | 16.20852554 | -0.170327396 | 0.046669 | 0.469916 |
| Miga2 | mitoguardin 2 | 12.18851665 | 13.6082813 | -0.17402251 | 0.048747 | 0.475516 |
| Zmat3 | zinc finger matrin type 3 | 8.144635873 | 9.096899827 | -0.174539142 | 0.01692 | 0.31561 |
| Hdhd2 | haloacid dehalogenase-like hydrolase domaiı | 18.01784332 | 20.15144918 | -0.176695771 | 0.049322 | 0.477954 |

| | | | | | | |
|---|---|---|---|---|---|---|
| Oaz2 | ornithine decarboxylase antizyme 2 | 61.79743066 | 69.16195485 | -0.177405026 | 0.034195 | 0.412986 |
| Efnb3 | ephrin B3 | 54.38474176 | 60.92496932 | -0.177527074 | 0.036113 | 0.424092 |
| Chid1 | chitinase domain containing 1 | 4.623998165 | 5.181040554 | -0.178449887 | 0.038748 | 0.43587 |
| Ndufv1 | NADH:ubiquinone oxidoreductase core subu | 54.66999419 | 61.38278303 | -0.1815519 | 0.028982 | 0.38834 |
| Mdh1 | malate dehydrogenase 1, NAD (soluble) | 201.5340874 | 227.1044275 | -0.186688462 | 0.028597 | 0.38601 |
| Slc22a17 | solute carrier family 22 (organic cation trans | 115.9131074 | 130.6289858 | -0.187122714 | 0.022969 | 0.357433 |
| Mpnd | MPN domain containing | 35.01187195 | 39.50649284 | -0.1881902 | 0.039304 | 0.439102 |
| Hexa | hexosaminidase A | 24.98480243 | 28.19908682 | -0.188635631 | 0.048393 | 0.475326 |
| Sub1 | SUB1 homolog (S. cerevisiae) | 50.39622203 | 56.88354378 | -0.188801397 | 0.040336 | 0.444433 |
| Tmem240 | transmembrane protein 240 | 6.123339963 | 6.916687985 | -0.189814066 | 0.031272 | 0.40182 |
| Ap3s2 | adaptor-related protein complex 3, sigma 2 s | 14.4586665 | 16.32277074 | -0.190234757 | 0.024699 | 0.365666 |
| Map1lc3a | microtubule-associated protein 1 light chain | 129.4465706 | 146.3470498 | -0.191631212 | 0.039974 | 0.44253 |
| Tspyl2 | TSPY-like 2 | 25.60048804 | 28.9488859 | -0.192021497 | 0.029489 | 0.390897 |
| Tecr | trans-2,3-enoyl-CoA reductase | 107.3312363 | 121.5982534 | -0.194293496 | 0.035536 | 0.421534 |
| Aldh3a2 | aldehyde dehydrogenase family 3, subfamily | 8.827543444 | 10.00021585 | -0.195738985 | 0.045656 | 0.463803 |
| Tsc22d4 | TSC22 domain family, member 4 | 20.1198627 | 22.80854384 | -0.196473457 | 0.045025 | 0.461155 |
| Cadm1 | cell adhesion molecule 1 | 16.32892193 | 18.53187012 | -0.197781768 | 0.049653 | 0.479319 |
| Sdhc | succinate dehydrogenase complex, subunit C | 28.71622106 | 32.64387354 | -0.199175681 | 0.030374 | 0.396303 |
| Ctsf | cathepsin F | 30.99272486 | 35.25590899 | -0.200657119 | 0.025015 | 0.36754 |
| Cby1 | chibby family member 1, beta catenin antag | 31.61381427 | 36.06220059 | -0.203473897 | 0.034517 | 0.414161 |
| Rhbdl3 | rhomboid like 3 | 5.857737746 | 6.685511593 | -0.206229739 | 0.045326 | 0.462098 |
| Ubb | ubiquitin B | 384.2712588 | 439.293794 | -0.206700142 | 0.038986 | 0.437099 |
| Gm1673 | predicted gene 1673 | 39.0208435 | 44.64780796 | -0.207828035 | 0.049711 | 0.47935 |
| Esrra | estrogen related receptor, alpha | 20.20140012 | 23.11325615 | -0.209358 | 0.017478 | 0.318417 |
| Blcap | bladder cancer associated protein | 26.09175657 | 29.89542949 | -0.210173677 | 0.02297 | 0.357433 |
| Mthfsd | methenyltetrahydrofolate synthetase domai | 2.674944622 | 3.072122498 | -0.21348899 | 0.048174 | 0.474657 |
| Bscl2 | Berardinelli-Seip congenital lipodystrophy 2 | 10.24838946 | 11.77355582 | -0.214357271 | 0.013952 | 0.289976 |
| Tmco3 | transmembrane and coiled-coil domains 3 | 5.580315977 | 6.404351908 | -0.215031341 | 0.045149 | 0.461421 |
| Ergic3 | ERGIC and golgi 3 | 43.53512115 | 50.05600348 | -0.215938323 | 0.013973 | 0.289976 |
| Ndufa9 | NADH:ubiquinone oxidoreductase subunit A | 41.11163804 | 47.27075415 | -0.216330052 | 0.023294 | 0.360229 |
| Crebl2 | cAMP responsive element binding protein-lik | 5.388119263 | 6.195973967 | -0.216591894 | 0.037022 | 0.428229 |
| Cisd1 | CDGSH iron sulfur domain 1 | 66.39958542 | 76.49315883 | -0.217535791 | 0.038907 | 0.436827 |
| Atp5d | ATP synthase, H+ transporting, mitochondria | 104.5263049 | 120.333836 | -0.217736067 | 0.018766 | 0.327186 |
| Mrps24 | mitochondrial ribosomal protein S24 | 16.79069051 | 19.35941248 | -0.21957825 | 0.030614 | 0.397591 |
| Aspscr1 | alveolar soft part sarcoma chromosome regic | 5.913095191 | 6.816338649 | -0.220177195 | 0.033113 | 0.411041 |
| Ypel3 | yippee like 3 | 77.98002293 | 90.01605416 | -0.221145765 | 0.032187 | 0.406386 |
| 1500009C( | RIKEN cDNA 1500009C09 gene | 49.62756877 | 57.28101898 | -0.221697111 | 0.028746 | 0.387682 |
| Stmn3 | stathmin-like 3 | 382.7831433 | 442.3403441 | -0.222554068 | 0.026089 | 0.373081 |
| Gm14325 | predicted gene 14325 | 9.103786049 | 10.5335906 | -0.223994979 | 0.049597 | 0.479302 |

| | | | | | | |
|---|---|---|---|---|---|---|
| Naxe | NAD(P)HX epimerase | 42.05860666 | 48.66554462 | -0.224227627 | 0.031444 | 0.402589 |
| Gipc1 | GIPC PDZ domain containing family, member | 42.68526169 | 49.33569321 | -0.224311757 | 0.017705 | 0.318827 |
| Tceal9 | transcription elongation factor A like 9 | 54.15507783 | 62.69020947 | -0.22478764 | 0.024385 | 0.363714 |
| Clpp | caseinolytic mitochondrial matrix peptidase | 32.17211514 | 37.22508725 | -0.224863273 | 0.022622 | 0.35495 |
| Lsm6 | LSM6 homolog, U6 small nuclear RNA and ml | 8.973737185 | 10.38901326 | -0.224977122 | 0.020255 | 0.336853 |
| Acbd4 | acyl-Coenzyme A binding domain containing | 8.480212505 | 9.815328175 | -0.226234276 | 0.023994 | 0.363714 |
| Cend1 | cell cycle exit and neuronal differentiation 1 | 111.5539843 | 129.3065165 | -0.22749561 | 0.01134 | 0.264414 |
| Elob | elongin B | 91.04621176 | 105.6707227 | -0.228644759 | 0.026118 | 0.373081 |
| Pnpo | pyridoxine 5'-phosphate oxidase | 9.893766521 | 11.48515578 | -0.228858833 | 0.02834 | 0.384263 |
| Cbarp | calcium channel, voltage-dependent, beta su | 59.30810287 | 68.81494469 | -0.229382811 | 0.019087 | 0.328038 |
| Ap1s1 | adaptor protein complex AP-1, sigma 1 | 83.43671132 | 96.88056579 | -0.230103957 | 0.042876 | 0.450034 |
| Sord | sorbitol dehydrogenase | 7.291233942 | 8.46761557 | -0.230178095 | 0.048984 | 0.476253 |
| Abhd16a | abhydrolase domain containing 16A | 18.4734063 | 21.46530563 | -0.230272821 | 0.008567 | 0.23271 |
| Narfl | nuclear prelamin A recognition factor-like | 5.937022182 | 6.901414773 | -0.231394967 | 0.029233 | 0.389312 |
| Fbxo44 | F-box protein 44 | 23.96792269 | 27.84742759 | -0.231841456 | 0.033507 | 0.411285 |
| Ift22 | intraflagellar transport 22 | 13.73696961 | 15.97611176 | -0.23239756 | 0.014019 | 0.289976 |
| Plppr3 | phospholipid phosphatase related 3 | 7.676985721 | 8.931494894 | -0.232527298 | 0.043281 | 0.452233 |
| Tmem161a | transmembrane protein 161A | 5.478266993 | 6.377965895 | -0.232808219 | 0.039425 | 0.439843 |
| Fkbp2 | FK506 binding protein 2 | 76.91412019 | 89.54065336 | -0.233722329 | 0.023475 | 0.360442 |
| 9-Mar | membrane-associated ring finger (C3HC4) 9 | 10.86300253 | 12.65829751 | -0.23394647 | 0.033055 | 0.411041 |
| Diras1 | DIRAS family, GTP-binding RAS-like 1 | 33.93020969 | 39.46158861 | -0.234048654 | 0.037861 | 0.432513 |
| Gpx1 | glutathione peroxidase 1 | 45.31975053 | 52.7934393 | -0.234702658 | 0.040825 | 0.446692 |
| Mrpl13 | mitochondrial ribosomal protein L13 | 3.915487765 | 4.564592248 | -0.234810091 | 0.042819 | 0.449801 |
| Isyna1 | myo-inositol 1-phosphate synthase A1 | 13.94622288 | 16.23824769 | -0.235111891 | 0.046393 | 0.469169 |
| Mob2 | MOB kinase activator 2 | 4.541148176 | 5.295097342 | -0.235164335 | 0.036011 | 0.424092 |
| Fam210b | family with sequence similarity 210, member | 11.22888415 | 13.08824688 | -0.235250168 | 0.036086 | 0.424092 |
| Bbs2 | Bardet-Biedl syndrome 2 (human) | 4.701274644 | 5.478016804 | -0.235255576 | 0.030475 | 0.396869 |
| Ckmt1 | creatine kinase, mitochondrial 1, ubiquitous | 45.80573766 | 53.39443263 | -0.235438352 | 0.031832 | 0.404338 |
| Hspbp1 | HSPA (heat shock 70kDa) binding protein, cyt | 36.37068004 | 42.40385231 | -0.235652994 | 0.017076 | 0.315784 |
| Pgrmc1 | progesterone receptor membrane componer | 119.0921889 | 139.015236 | -0.23725263 | 0.01888 | 0.327826 |
| Ddrgk1 | DDRGK domain containing 1 | 27.35220531 | 31.92558504 | -0.238228322 | 0.010807 | 0.259903 |
| Nrbp2 | nuclear receptor binding protein 2 | 24.01999755 | 28.06174331 | -0.238963324 | 0.020906 | 0.341521 |
| Ctbp2 | C-terminal binding protein 2 | 4.08989045 | 4.788123158 | -0.242171192 | 0.010117 | 0.251529 |
| Xpc | xeroderma pigmentosum, complementation | 2.680485004 | 3.137704993 | -0.242877297 | 0.033324 | 0.411285 |
| Dnajc27 | DnaJ heat shock protein family (Hsp40) mem | 29.47969653 | 34.50282732 | -0.242962548 | 0.014157 | 0.290588 |
| Mrpl58 | mitochondrial ribosomal protein L58 | 8.96500956 | 10.51599335 | -0.244011266 | 0.020539 | 0.338826 |
| Ndufv3 | NADH:ubiquinone oxidoreductase core subu | 22.54658631 | 26.4503609 | -0.244570732 | 0.020661 | 0.339596 |
| Tmem134 | transmembrane protein 134 | 6.971753166 | 8.178204282 | -0.244614496 | 0.044371 | 0.457039 |
| Kcnj9 | potassium inwardly-rectifying channel, subfa | 18.60806317 | 21.86739271 | -0.247399798 | 0.030922 | 0.399905 |

| | | | | | | |
|---|---|---|---|---|---|---|
| Tbcb | tubulin folding cofactor B | 45.34343252 | 53.29948194 | -0.247588277 | 0.014864 | 0.298753 |
| Osbpl7 | oxysterol binding protein-like 7 | 2.419702858 | 2.843938372 | -0.247602727 | 0.033701 | 0.411285 |
| Zcchc18 | zinc finger, CCHC domain containing 18 | 39.364291 | 46.28622357 | -0.247784347 | 0.008662 | 0.23324 |
| Fam96b | family with sequence similarity 96, member I | 15.49223856 | 18.22748339 | -0.249162295 | 0.028963 | 0.38834 |
| Mrpl40 | mitochondrial ribosomal protein L40 | 11.06556796 | 13.02688191 | -0.249244125 | 0.039707 | 0.441694 |
| Ilvbl | ilvB (bacterial acetolactate synthase)-like | 3.962718548 | 4.659766986 | -0.249249168 | 0.038058 | 0.433086 |
| Sf3b5 | splicing factor 3b, subunit 5 | 8.705831118 | 10.2548162 | -0.249484776 | 0.036799 | 0.426753 |
| Fn3k | fructosamine 3 kinase | 3.825012689 | 4.500508676 | -0.249683357 | 0.047824 | 0.473596 |
| Spr | sepiapterin reductase | 8.623846735 | 10.15389553 | -0.249919245 | 0.049088 | 0.476472 |
| Lyrm9 | LYR motif containing 9 | 3.307238652 | 3.892352972 | -0.250737371 | 0.033906 | 0.411285 |
| Zdhhc1 | zinc finger, DHHC domain containing 1 | 6.364969187 | 7.502432668 | -0.250829878 | 0.016423 | 0.31404 |
| Scg5 | secretogranin V | 81.90469379 | 96.53480752 | -0.25126817 | 0.004534 | 0.169288 |
| Ubxn6 | UBX domain protein 6 | 2.680982396 | 3.15966592 | -0.252453033 | 0.028835 | 0.387794 |
| Brd3os | bromodomain containing 3, opposite strand | 10.87229707 | 12.83253816 | -0.252459972 | 0.030423 | 0.396647 |
| Zfr2 | zinc finger RNA binding protein 2 | 5.611189432 | 6.620699779 | -0.253525555 | 0.010174 | 0.252588 |
| Sod1 | superoxide dismutase 1, soluble | 36.87142609 | 43.56793437 | -0.255230936 | 0.024312 | 0.363714 |
| Parm1 | prostate androgen-regulated mucin-like prot | 9.638302156 | 11.39736432 | -0.257003702 | 0.025222 | 0.36904 |
| Iscu | iron-sulfur cluster assembly enzyme | 29.73667016 | 35.19928844 | -0.257837332 | 0.008544 | 0.23271 |
| Rbfa | ribosome binding factor A | 19.11493029 | 22.6692563 | -0.260911621 | 0.025454 | 0.370192 |
| Ndufb11 | NADH:ubiquinone oxidoreductase subunit B | 104.8831856 | 124.4858767 | -0.261301088 | 0.044875 | 0.460965 |
| Gga1 | golgi associated, gamma adaptin ear containi | 14.2125131 | 16.87469078 | -0.261791935 | 0.002255 | 0.119646 |
| Ldhb | lactate dehydrogenase B | 216.1304689 | 256.7347028 | -0.262730575 | 0.004025 | 0.16123 |
| Inafm1 | InaF motif containing 1 | 17.46961387 | 20.76511935 | -0.263380894 | 0.022441 | 0.35382 |
| Snx32 | sorting nexin 32 | 34.98622855 | 41.61086858 | -0.263617369 | 0.00553 | 0.188022 |
| Oaz1 | ornithine decarboxylase antizyme 1 | 99.08987393 | 117.7202579 | -0.264009738 | 0.008322 | 0.228857 |
| Fhl1 | four and a half LIM domains 1 | 19.91132121 | 23.69001628 | -0.264717511 | 0.001374 | 0.088894 |
| Pnck | pregnancy upregulated non-ubiquitously exp | 34.41853079 | 40.95830146 | -0.264911966 | 0.026879 | 0.376367 |
| Rnf25 | ring finger protein 25 | 11.4392766 | 13.62817623 | -0.265186939 | 0.049538 | 0.478994 |
| Ptgr2 | prostaglandin reductase 2 | 4.281683066 | 5.08910569 | -0.265204581 | 0.019112 | 0.328038 |
| Pnkd | paroxysmal nonkinesiogenic dyskinesia | 18.16769934 | 21.61751874 | -0.265604795 | 0.000153 | 0.023397 |
| Podxl2 | podocalyxin-like 2 | 14.15199837 | 16.83711094 | -0.265836824 | 0.003099 | 0.13957 |
| Gabrg1 | gamma-aminobutyric acid (GABA) A receptor | 3.437330419 | 4.095565754 | -0.268961099 | 0.011304 | 0.264259 |
| Ascc1 | activating signal cointegrator 1 complex sub | 8.348606361 | 9.953843834 | -0.269257263 | 0.025218 | 0.36904 |
| Mea1 | male enhanced antigen 1 | 24.23205702 | 28.92484509 | -0.269565495 | 0.00889 | 0.236504 |
| Spg7 | SPG7, paraplegin matrix AAA peptidase subu | 11.8904403 | 14.18485348 | -0.269800148 | 0.018942 | 0.327826 |
| Rab10os | RAB10, member RAS oncogene family, oppos | 3.526137141 | 4.207628844 | -0.270766374 | 0.016957 | 0.315784 |
| Gm17018 | predicted gene 17018 | 17.33917954 | 20.7230495 | -0.27205295 | 0.030485 | 0.396869 |
| Pink1 | PTEN induced putative kinase 1 | 131.018051 | 156.5647585 | -0.272371025 | 0.028516 | 0.385769 |
| Mlycd | malonyl-CoA decarboxylase | 8.337119873 | 9.9680175 | -0.272967889 | 0.032207 | 0.406386 |

| | | | | | | |
|---|---|---|---|---|---|---|
| Gad1 | glutamate decarboxylase 1 | 27.1279637 | 32.46974433 | -0.273551103 | 0.007434 | 0.21876 |
| Grk3 | G protein-coupled receptor kinase 3 | 8.981407512 | 10.73255993 | -0.273649501 | 0.026836 | 0.376367 |
| BC029722 | cDNA sequence BC029722 | 17.59774237 | 21.06456526 | -0.273970127 | 0.012339 | 0.276598 |
| Mief2 | mitochondrial elongation factor 2 | 5.274852817 | 6.324022126 | -0.276575278 | 0.039995 | 0.44253 |
| Chchd6 | coiled-coil-helix-coiled-coil-helix domain cor | 17.08121778 | 20.49943145 | -0.276836209 | 0.027791 | 0.381817 |
| Prune2 | prune homolog 2 | 2.477714664 | 2.971736898 | -0.276913462 | 0.010398 | 0.256085 |
| Mcrip1 | MAPK regulated corepressor interacting prot | 47.6840245 | 57.20521644 | -0.277758843 | 0.005024 | 0.178181 |
| Bex1 | brain expressed X-linked 1 | 22.78721986 | 27.36811569 | -0.277998615 | 0.034719 | 0.414579 |
| Hint1 | histidine triad nucleotide binding protein 1 | 126.9128158 | 152.4298385 | -0.278464773 | 0.017246 | 0.316008 |
| Trmt112 | tRNA methyltransferase 11-2 | 14.22593197 | 17.102255 | -0.278858912 | 0.019772 | 0.333512 |
| Abcf3 | ATP-binding cassette, sub-family F (GCN20), r | 12.37108288 | 14.8606749 | -0.28000675 | 0.005398 | 0.18512 |
| Maged2 | melanoma antigen, family D, 2 | 12.38548866 | 14.89109067 | -0.280954867 | 0.01189 | 0.271429 |
| Gpx4 | glutathione peroxidase 4 | 171.6271356 | 206.3988781 | -0.28127917 | 0.043044 | 0.450751 |
| Plch2 | phospholipase C, eta 2 | 12.80508309 | 15.39534773 | -0.281914074 | 0.017917 | 0.320299 |
| Mtfp1 | mitochondrial fission process 1 | 39.22199769 | 47.24584495 | -0.282883506 | 0.020024 | 0.334904 |
| Tspan4 | tetraspanin 4 | 8.062846126 | 9.713995754 | -0.283616255 | 0.036765 | 0.426634 |
| Phf1 | PHD finger protein 1 | 9.365761585 | 11.31202666 | -0.285196925 | 0.023336 | 0.360229 |
| Mgst3 | microsomal glutathione S-transferase 3 | 48.36058722 | 58.38847822 | -0.285389248 | 0.01402 | 0.289976 |
| Lzts2 | leucine zipper, putative tumor suppressor 2 | 16.06583018 | 19.36819518 | -0.285880828 | 0.037697 | 0.431765 |
| Pnma3 | paraneoplastic antigen MA3 | 6.041409784 | 7.297384083 | -0.286119802 | 0.015231 | 0.303835 |
| Mroh1 | maestro heat-like repeat family member 1 | 2.551918475 | 3.078124883 | -0.286295189 | 0.023379 | 0.360229 |
| Pde9a | phosphodiesterase 9A | 4.390605178 | 5.305965673 | -0.286516408 | 0.028788 | 0.387794 |
| Gabrg3 | gamma-aminobutyric acid (GABA) A receptor | 1.26942499 | 1.532108517 | -0.286521108 | 0.027752 | 0.381577 |
| Tomm7 | translocase of outer mitochondrial membrar | 28.49655439 | 34.42704064 | -0.286562833 | 0.042512 | 0.449801 |
| Gsta4 | glutathione S-transferase, alpha 4 | 30.64741414 | 37.02059118 | -0.287185068 | 0.011925 | 0.27157 |
| Ptpn5 | protein tyrosine phosphatase, non-receptor t | 14.45466297 | 17.4760839 | -0.289315063 | 0.00248 | 0.123592 |
| Rab3c | RAB3C, member RAS oncogene family | 31.29513227 | 37.86618026 | -0.290552551 | 0.001856 | 0.107364 |
| Ifitm10 | interferon induced transmembrane protein 1 | 3.126918279 | 3.789756062 | -0.290596444 | 0.031862 | 0.404338 |
| Trim62 | tripartite motif-containing 62 | 3.399237815 | 4.117477347 | -0.290952723 | 0.01577 | 0.307705 |
| Sox1 | SRY (sex determining region Y)-box 1 | 4.103714139 | 4.97093944 | -0.292573206 | 0.025774 | 0.372221 |
| Tram1l1 | translocation associated membrane protein 1 | 7.132368908 | 8.662403909 | -0.293281049 | 0.029728 | 0.392694 |
| Eml2 | echinoderm microtubule associated protein | 50.66633355 | 61.43335087 | -0.293343717 | 0.003387 | 0.144949 |
| Smim12 | small integral membrane protein 12 | 25.40332625 | 30.86678296 | -0.293427504 | 0.019374 | 0.330205 |
| Dzip1 | DAZ interacting protein 1 | 4.226815635 | 5.120110004 | -0.293435803 | 0.047704 | 0.472936 |
| Smarcd3 | SWI/SNF related, matrix associated, actin dep | 6.823417024 | 8.28397254 | -0.29575758 | 0.013717 | 0.288529 |
| Hspb8 | heat shock protein 8 | 11.96473464 | 14.55372405 | -0.296518964 | 0.04201 | 0.449674 |
| Ap1s2 | adaptor-related protein complex 1, sigma 2 s | 9.159501806 | 11.14139495 | -0.29770918 | 0.017576 | 0.318417 |
| Exosc5 | exosome component 5 | 2.412168242 | 2.93874551 | -0.298252562 | 0.009867 | 0.247402 |
| Timm17b | translocase of inner mitochondrial membrar | 5.576520081 | 6.799749117 | -0.29914547 | 0.027509 | 0.379964 |

| | | | | | | |
|---|---|---|---|---|---|---|
| Cplx1 | complexin 1 | 217.6556267 | 265.0444503 | -0.29946484 | 0.003388 | 0.144949 |
| Acaa2 | acetyl-Coenzyme A acyltransferase 2 (mitoch | 16.44718201 | 20.0220664 | -0.299637476 | 0.040433 | 0.444433 |
| Tspan9 | tetraspanin 9 | 13.58119419 | 16.55667254 | -0.302023079 | 0.02439 | 0.363714 |
| Nxph1 | neurexophilin 1 | 5.061397937 | 6.184289276 | -0.302801192 | 0.009617 | 0.244306 |
| Gale | galactose-4-epimerase, UDP | 4.574169319 | 5.591128126 | -0.302964809 | 0.028137 | 0.382668 |
| Cds1 | CDP-diacylglycerol synthase 1 | 15.46492691 | 18.88493365 | -0.30310629 | 0.00432 | 0.165914 |
| Sdhaf3 | succinate dehydrogenase complex assembly | 1.721536485 | 2.106967741 | -0.304829128 | 0.049354 | 0.478 |
| Gpsm1 | G-protein signalling modulator 1 (AGS3-like, | 6.407190566 | 7.836838109 | -0.305500956 | 0.005536 | 0.188022 |
| Mrpl14 | mitochondrial ribosomal protein L14 | 12.66403357 | 15.49453479 | -0.305641202 | 0.024932 | 0.367325 |
| Sncb | synuclein, beta | 109.3185439 | 133.8294124 | -0.306375617 | 0.007617 | 0.220817 |
| Smim17 | small integral membrane protein 17 | 4.604769444 | 5.647524084 | -0.308279097 | 0.041758 | 0.449122 |
| Frs3 | fibroblast growth factor receptor substrate 3 | 4.623214276 | 5.678516031 | -0.31003145 | 0.013485 | 0.287077 |
| Nenf | neuron derived neurotrophic factor | 47.46366759 | 58.30790114 | -0.310804734 | 0.00224 | 0.119646 |
| Ptpru | protein tyrosine phosphatase, receptor type, | 4.755921162 | 5.843128037 | -0.311124105 | 0.00847 | 0.232193 |
| Atp5h | ATP synthase, H+ transporting, mitochondria | 99.80731808 | 122.6797662 | -0.312771327 | 0.001734 | 0.103969 |
| Camk1 | calcium/calmodulin-dependent protein kina | 6.97206196 | 8.573263738 | -0.3132449 | 0.002641 | 0.127172 |
| Dohh | deoxyhypusine hydroxylase/monooxygenase | 8.765433078 | 10.78147457 | -0.313626847 | 0.033019 | 0.411041 |
| Dzank1 | double zinc ribbon and ankyrin repeat domai | 17.97854022 | 22.10463335 | -0.314178621 | 0.023305 | 0.360229 |
| Aldh2 | aldehyde dehydrogenase 2, mitochondrial | 14.47546849 | 17.80470095 | -0.314488726 | 0.006815 | 0.208871 |
| Anxa2 | annexin A2 | 4.401818008 | 5.423748908 | -0.31517737 | 0.036095 | 0.424092 |
| Dgat1 | diacylglycerol O-acyltransferase 1 | 4.346757287 | 5.3681848 | -0.316864886 | 0.02184 | 0.349401 |
| 3110040N | RIKEN cDNA 3110040N11 gene | 6.53930217 | 8.082594029 | -0.317622953 | 0.044111 | 0.456222 |
| Fuca1 | fucosidase, alpha-L- 1, tissue | 10.33445337 | 12.74982456 | -0.318310394 | 0.005336 | 0.184391 |
| Abat | 4-aminobutyrate aminotransferase | 37.03184177 | 45.67343387 | -0.318432144 | 0.004252 | 0.164736 |
| Efnb2 | ephrin B2 | 4.012105744 | 4.952756785 | -0.318641721 | 0.020548 | 0.338826 |
| Tbc1d9 | TBC1 domain family, member 9 | 14.34449678 | 17.70636717 | -0.320513005 | 0.024748 | 0.365666 |
| Zwint | ZW10 interactor | 109.9326307 | 135.914292 | -0.320736278 | 0.000248 | 0.03084 |
| Rwdd2a | RWD domain containing 2A | 11.00446088 | 13.63523414 | -0.322038135 | 0.042255 | 0.449801 |
| Tmtc4 | transmembrane and tetratricopeptide repea | 1.078878484 | 1.335075742 | -0.322651608 | 0.035128 | 0.417415 |
| B9d1 | B9 protein domain 1 | 5.871023448 | 7.270546885 | -0.322701632 | 0.048317 | 0.475326 |
| Pcsk1 | proprotein convertase subtilisin/kexin type 1 | 2.765211888 | 3.418351564 | -0.323287166 | 0.039523 | 0.440649 |
| Uimc1 | ubiquitin interaction motif containing 1 | 2.132717829 | 2.645928071 | -0.324092605 | 0.021269 | 0.344035 |
| Commd9 | COMM domain containing 9 | 15.30449674 | 18.97601405 | -0.324095433 | 0.004242 | 0.164711 |
| Ndst3 | N-deacetylase/N-sulfotransferase (heparan gl | 1.43895487 | 1.788697596 | -0.325505214 | 0.042464 | 0.449801 |
| Gria4 | glutamate receptor, ionotropic, AMPA4 (alp | 7.534496388 | 9.336063244 | -0.325545207 | 0.030172 | 0.394834 |
| 2010204K | RIKEN cDNA 2010204K13 gene | 7.983361775 | 9.922780847 | -0.326009088 | 0.047824 | 0.473596 |
| Fam161b | family with sequence similarity 161, membe | 2.255495943 | 2.800644418 | -0.326176999 | 0.017873 | 0.319851 |
| Carmil3 | capping protein regulator and myosin 1 links | 5.066385563 | 6.290552674 | -0.326371003 | 0.004879 | 0.175521 |
| Nbdy | negative regulator of P-body association | 5.638091443 | 7.011487477 | -0.327214455 | 0.014496 | 0.295459 |

| | | | | | | |
|---|---|---|---|---|---|---|
| Ccdc28a | coiled-coil domain containing 28A | 4.984713313 | 6.198383377 | -0.328511725 | 0.011895 | 0.271429 |
| Psmg1 | proteasome (prosome, macropain) assembly | 6.46712472 | 8.044564079 | -0.328724562 | 0.022162 | 0.351612 |
| Car10 | carbonic anhydrase 10 | 5.016602109 | 6.228834362 | -0.328842917 | 0.033262 | 0.411225 |
| Gng4 | guanine nucleotide binding protein (G protei | 10.12031221 | 12.55966417 | -0.329100766 | 0.033444 | 0.411285 |
| Fbxw9 | F-box and WD-40 domain protein 9 | 3.543858303 | 4.418171439 | -0.330617523 | 0.014775 | 0.297981 |
| Cdkl4 | cyclin-dependent kinase-like 4 | 5.336709082 | 6.640894057 | -0.331131905 | 0.034499 | 0.414161 |
| Chga | chromogranin A | 33.20488956 | 41.37977348 | -0.332492345 | 0.009815 | 0.246441 |
| Trmt11 | tRNA methyltransferase 11 | 0.988568109 | 1.234364302 | -0.332994647 | 0.048586 | 0.475516 |
| Aven | apoptosis, caspase activation inhibitor | 1.540855695 | 1.920778744 | -0.333817835 | 0.041606 | 0.449122 |
| Dlx1 | distal-less homeobox 1 | 5.008794121 | 6.25300079 | -0.333866026 | 0.021504 | 0.346168 |
| Clba1 | clathrin binding box of aftiphilin containing | 3.965395629 | 4.941820552 | -0.33465059 | 0.040018 | 0.44253 |
| Thap3 | THAP domain containing, apoptosis associat | 6.662430057 | 8.320010672 | -0.334669608 | 0.020691 | 0.339596 |
| Slc6a7 | solute carrier family 6 (neurotransmitter tra | 13.98023343 | 17.45311086 | -0.335582266 | 0.010448 | 0.256495 |
| Ephx1 | epoxide hydrolase 1, microsomal | 14.15293923 | 17.67061384 | -0.335728309 | 0.012845 | 0.281613 |
| Pvalb | parvalbumin | 32.08902868 | 40.09207774 | -0.33768107 | 0.036342 | 0.425088 |
| Rxra | retinoid X receptor alpha | 5.567431 | 6.956960366 | -0.337750775 | 0.006352 | 0.202147 |
| Vstm2b | V-set and transmembrane domain containin | 10.10111786 | 12.65511062 | -0.337816289 | 0.011002 | 0.262772 |
| Cbx7 | chromobox 7 | 4.31774892 | 5.399868082 | -0.338560142 | 0.001868 | 0.107364 |
| Tgfb3 | transforming growth factor, beta 3 | 2.884222092 | 3.610633129 | -0.339076896 | 0.047415 | 0.47273 |
| Metrnl | meteorin, glial cell differentiation regulator-l | 4.889321537 | 6.126534904 | -0.341733518 | 0.028393 | 0.384695 |
| Acsf3 | acyl-CoA synthetase family member 3 | 3.327443137 | 4.180831231 | -0.342468981 | 0.021116 | 0.343188 |
| Wls | wntless WNT ligand secretion mediator | 4.522756485 | 5.673239741 | -0.343061243 | 0.036738 | 0.426602 |
| Megf11 | multiple EGF-like-domains 11 | 2.225150687 | 2.79436375 | -0.343463971 | 0.005998 | 0.196144 |
| Tmem176a | transmembrane protein 176A | 3.670671237 | 4.618716957 | -0.345966328 | 0.008964 | 0.236504 |
| Rcan2 | regulator of calcineurin 2 | 19.10740861 | 24.03635193 | -0.346280393 | 0.048533 | 0.475516 |
| Prdm16 | PR domain containing 16 | 1.409167619 | 1.772023829 | -0.347206694 | 0.041012 | 0.447817 |
| Lnx1 | ligand of numb-protein X 1 | 3.074033017 | 3.866694804 | -0.34783829 | 0.007918 | 0.224398 |
| Spsb4 | splA/ryanodine receptor domain and SOCS b | 1.129734383 | 1.42451914 | -0.348363724 | 0.04242 | 0.449801 |
| Snrnp25 | small nuclear ribonucleoprotein 25 (U11/U1 | 11.84163474 | 14.94985412 | -0.348663107 | 0.013798 | 0.289375 |
| Grin3a | glutamate receptor ionotropic, NMDA3A | 2.965416376 | 3.737533596 | -0.350090802 | 0.014745 | 0.297981 |
| Ikbip | IKBKB interacting protein | 2.22671704 | 2.81229434 | -0.350541985 | 0.010402 | 0.256085 |
| Anapc13 | anaphase promoting complex subunit 13 | 8.645409798 | 10.93070918 | -0.351314595 | 0.007984 | 0.225748 |
| Elof1 | ELF1 homolog, elongation factor 1 | 21.90160645 | 27.67374009 | -0.351809799 | 0.000601 | 0.053064 |
| Cntnap4 | contactin associated protein-like 4 | 1.597601313 | 2.014878836 | -0.352003429 | 0.031408 | 0.402589 |
| Tenm4 | teneurin transmembrane protein 4 | 2.728214534 | 3.442882293 | -0.352515151 | 0.017295 | 0.316243 |
| Sdr39u1 | short chain dehydrogenase/reductase family | 5.293806927 | 6.691258141 | -0.352828777 | 0.009635 | 0.244306 |
| Myl6b | myosin, light polypeptide 6B | 5.477543292 | 6.941641383 | -0.354484595 | 0.025888 | 0.37282 |
| Nat8f1 | N-acetyltransferase 8 (GCN5-related) family n | 7.659043624 | 9.709028397 | -0.355168689 | 0.036523 | 0.426227 |
| Gm9866 | predicted gene 9866 | 2.468995741 | 3.122887881 | -0.355271348 | 0.004641 | 0.171146 |

| | | | | | | |
|---|---|---|---|---|---|---|
| Mcrip2 | MAPK regulated corepressor interacting prot | 13.32273833 | 16.88619654 | -0.355729478 | 0.017225 | 0.316008 |
| Lrsam1 | leucine rich repeat and sterile alpha motif co | 4.692634324 | 5.940151174 | -0.355883972 | 0.000982 | 0.072698 |
| Crtac1 | cartilage acidic protein 1 | 30.90006998 | 39.18069448 | -0.356900696 | 0.015616 | 0.307206 |
| Mmab | methylmalonic aciduria (cobalamin deficienc | 6.922478615 | 8.782383922 | -0.357277588 | 0.000781 | 0.06342 |
| Coprs | coordinator of PRMT5, differentiation stimul | 5.609496429 | 7.121871831 | -0.357505367 | 0.005828 | 0.194211 |
| Cdh22 | cadherin 22 | 3.918114544 | 4.973729288 | -0.358409177 | 0.009244 | 0.238921 |
| Crocc | ciliary rootlet coiled-coil, rootletin | 4.642924615 | 5.908266679 | -0.36153554 | 0.00053 | 0.049955 |
| Arl2 | ADP-ribosylation factor-like 2 | 14.77532066 | 18.82644599 | -0.363674811 | 0.001691 | 0.102076 |
| Cib2 | calcium and integrin binding family member | 12.51403719 | 15.92479404 | -0.363825275 | 0.021936 | 0.350233 |
| Atp2b4 | ATPase, Ca++ transporting, plasma membrane | 5.045359104 | 6.414131888 | -0.364220854 | 0.030296 | 0.395865 |
| Mycbp | MYC binding protein | 1.786406483 | 2.281021653 | -0.365214994 | 0.037261 | 0.429285 |
| Tmem192 | transmembrane protein 192 | 5.628670855 | 7.177085452 | -0.365237588 | 0.006284 | 0.201399 |
| Impact | impact, RWD domain protein | 41.25444901 | 52.61048757 | -0.365677297 | 0.000477 | 0.046785 |
| Cmss1 | cms small ribosomal subunit 1 | 3.788633277 | 4.842843378 | -0.366785005 | 0.038567 | 0.435499 |
| Tppp3 | tubulin polymerization-promoting protein fa | 22.43198179 | 28.65739812 | -0.367998999 | 0.029039 | 0.38867 |
| Meis2 | Meis homeobox 2 | 3.115709882 | 3.972873331 | -0.368110158 | 0.023873 | 0.363225 |
| S100a10 | S100 calcium binding protein A10 (calpactin | 11.94500376 | 15.27823989 | -0.368289253 | 0.030206 | 0.39498 |
| Cryz | crystallin, zeta | 2.551059561 | 3.262926887 | -0.369842204 | 0.024233 | 0.363714 |
| Zfp385a | zinc finger protein 385A | 16.51696864 | 21.12883648 | -0.370683973 | 0.01284 | 0.281613 |
| Cpq | carboxypeptidase Q | 3.271361061 | 4.179615717 | -0.370784104 | 0.036737 | 0.426602 |
| B630019K0 | RIKEN cDNA B630019K06 gene | 12.37568057 | 15.85026217 | -0.371833377 | 0.003551 | 0.148691 |
| Ucp2 | uncoupling protein 2 (mitochondrial, proton | 5.205332486 | 6.661854563 | -0.372189562 | 0.019109 | 0.328038 |
| Klc4 | kinesin light chain 4 | 8.332771863 | 10.68269408 | -0.372568496 | 0.000533 | 0.049955 |
| Khk | ketohexokinase | 4.537153678 | 5.832786291 | -0.375244323 | 0.028143 | 0.382668 |
| Nkx6-2 | NK6 homeobox 2 | 11.85881594 | 15.21703473 | -0.375321775 | 0.008716 | 0.234212 |
| Prdx4 | peroxiredoxin 4 | 5.371294722 | 6.902539357 | -0.376086828 | 0.018111 | 0.322482 |
| Mgst1 | microsomal glutathione S-transferase 1 | 4.261252623 | 5.4756714 | -0.376338998 | 0.026616 | 0.375863 |
| Rab26 | RAB26, member RAS oncogene family | 9.992455656 | 12.85598673 | -0.376691614 | 0.043388 | 0.452562 |
| Nol3 | nucleolar protein 3 (apoptosis repressor with | 3.495464371 | 4.487898141 | -0.377038356 | 0.003783 | 0.154715 |
| Cyb5rl | cytochrome b5 reductase-like | 0.572809703 | 0.735796247 | -0.377914114 | 0.042683 | 0.449801 |
| Snx8 | sorting nexin 8 | 4.305533187 | 5.543345172 | -0.378275662 | 0.017362 | 0.317131 |
| Dmac2 | distal membrane arm assembly complex 2 | 9.232272538 | 11.88985249 | -0.378937826 | 0.000503 | 0.048481 |
| Klhdc8b | kelch domain containing 8B | 2.489510702 | 3.205878854 | -0.379232649 | 0.003172 | 0.140489 |
| Zfp575 | zinc finger protein 575 | 2.049978432 | 2.643189382 | -0.379678283 | 0.031844 | 0.404338 |
| Aifm3 | apoptosis-inducing factor, mitochondrion-as | 10.527075 | 13.56054694 | -0.380164985 | 0.009093 | 0.237092 |
| Trpm2 | transient receptor potential cation channel, | 1.524276987 | 1.964736221 | -0.381687495 | 0.005125 | 0.179231 |
| Zfp768 | zinc finger protein 768 | 3.030911777 | 3.911598699 | -0.381972085 | 0.018065 | 0.322299 |
| Spire2 | spire type actin nucleation factor 2 | 5.915721206 | 7.633244511 | -0.382398843 | 0.006018 | 0.196435 |
| Ggact | gamma-glutamylamine cyclotransferase | 2.598447514 | 3.35851777 | -0.384183123 | 0.005501 | 0.187548 |

| | | | | | | |
|---|---|---|---|---|---|---|
| Ppp1r1b | protein phosphatase 1, regulatory inhibitor s | 12.14881026 | 15.68734861 | -0.384228378 | 0.01457 | 0.296217 |
| Samd14 | sterile alpha motif domain containing 14 | 21.20775544 | 27.39257968 | -0.384638432 | 0.00041 | 0.042141 |
| Dcdc2a | doublecortin domain containing 2a | 0.906716206 | 1.17213376 | -0.385951441 | 0.031264 | 0.40182 |
| Polm | polymerase (DNA directed), mu | 1.563505213 | 2.028143586 | -0.386195119 | 0.048816 | 0.475925 |
| Fgf11 | fibroblast growth factor 11 | 8.138758787 | 10.53351637 | -0.386412522 | 0.008121 | 0.226134 |
| Morn2 | MORN repeat containing 2 | 12.10985957 | 15.67973555 | -0.386969696 | 0.022489 | 0.354158 |
| Copz2 | coatomer protein complex, subunit zeta 2 | 2.924811663 | 3.790469388 | -0.388004399 | 0.041844 | 0.449674 |
| Tnfaip8 | tumor necrosis factor, alpha-induced protein | 0.996962804 | 1.291252491 | -0.388176426 | 0.025963 | 0.372836 |
| Zswim3 | zinc finger SWIM-type containing 3 | 2.224267895 | 2.880322616 | -0.388924152 | 0.022312 | 0.353044 |
| Cdh4 | cadherin 4 | 1.751594312 | 2.27251322 | -0.390832941 | 0.020507 | 0.338782 |
| Tmem106c | transmembrane protein 106C | 8.769289325 | 11.38957583 | -0.391045348 | 0.002823 | 0.133079 |
| Pygm | muscle glycogen phosphorylase | 11.09581816 | 14.40963456 | -0.391909026 | 0.002831 | 0.133079 |
| Tctn2 | tectonic family member 2 | 1.879238873 | 2.440119495 | -0.392035618 | 0.003445 | 0.14633 |
| P3h1 | prolyl 3-hydroxylase 1 | 2.369372412 | 3.082613087 | -0.393134285 | 0.005621 | 0.189963 |
| Col5a1 | collagen, type V, alpha 1 | 1.054923379 | 1.371665462 | -0.394234888 | 0.036732 | 0.426602 |
| Adssl1 | adenylosuccinate synthetase like 1 | 8.045493265 | 10.47714165 | -0.396565586 | 0.048036 | 0.474628 |
| C77080 | expressed sequence C77080 | 2.564825866 | 3.333130556 | -0.396607222 | 0.033376 | 0.411285 |
| Gm10076 | predicted gene 10076 | 121.1671011 | 157.8317761 | -0.397053953 | 0.013552 | 0.287813 |
| Hes1 | hes family bHLH transcription factor 1 | 4.728621338 | 6.166065627 | -0.397295241 | 0.02426 | 0.363714 |
| Rnf227 | ring finger protein 227 | 37.46748481 | 48.84803053 | -0.397743097 | 0.00016 | 0.024035 |
| 1700001L1 | RIKEN cDNA 1700001L19 gene | 3.404150225 | 4.449169765 | -0.400170418 | 0.0203 | 0.336853 |
| Myo16 | myosin XVI | 2.308811838 | 3.011518391 | -0.400187785 | 0.001407 | 0.090323 |
| Slc22a4 | solute carrier family 22 (organic cation trans | 2.372334499 | 3.102650572 | -0.40051032 | 0.038415 | 0.434618 |
| Fras1 | Fraser extracellular matrix complex subunit 1 | 0.54253885 | 0.70950526 | -0.400609736 | 0.007655 | 0.221556 |
| Krt1 | keratin 1 | 1.491482628 | 1.950385654 | -0.400803635 | 0.033844 | 0.411285 |
| Gm14597 | predicted gene 14597 | 16.67841955 | 21.82614893 | -0.403100239 | 0.011997 | 0.272506 |
| Slc5a6 | solute carrier family 5 (sodium-dependent vi | 3.464254281 | 4.538190923 | -0.404243978 | 0.029186 | 0.389312 |
| Mrps6 | mitochondrial ribosomal protein S6 | 7.860056718 | 10.28334553 | -0.404250507 | 0.006832 | 0.208871 |
| Csrp2 | cysteine and glycine-rich protein 2 | 5.313795065 | 6.972391716 | -0.406221573 | 0.01567 | 0.307705 |
| AI854703 | expressed sequence AI854703 | 5.192562044 | 6.824440112 | -0.408941594 | 0.012794 | 0.281232 |
| Ppp1r14a | protein phosphatase 1, regulatory inhibitor s | 19.59824761 | 25.75570485 | -0.409191832 | 0.034521 | 0.414161 |
| Rtl8c | retrotransposon Gag like 8C | 108.7810555 | 143.1727938 | -0.411674226 | 0.000349 | 0.03892 |
| Rab3b | RAB3B, member RAS oncogene family | 8.184394493 | 10.79225838 | -0.412907407 | 0.011868 | 0.271429 |
| Cacna1g | calcium channel, voltage-dependent, T type, | 4.437293497 | 5.843257438 | -0.413333343 | 0.045066 | 0.461229 |
| Galk1 | galactokinase 1 | 6.812749231 | 8.988987646 | -0.413847671 | 0.021145 | 0.343188 |
| Gaa | glucosidase, alpha, acid | 41.32208057 | 54.49031761 | -0.414172611 | 4.93E-05 | 0.012243 |
| Vstm5 | V-set and transmembrane domain containing | 2.771353429 | 3.654336652 | -0.415956962 | 0.011297 | 0.264259 |
| Rpl41 | ribosomal protein L41 | 200.2543384 | 264.5445667 | -0.416620269 | 0.004061 | 0.161238 |
| Unc119 | unc-119 lipid binding chaperone | 8.425369579 | 11.1238023 | -0.416831747 | 0.003318 | 0.142987 |

| | | | | | | |
|---|---|---|---|---|---|---|
| Rbms3 | RNA binding motif, single stranded interactir | 1.627788199 | 2.152325698 | -0.418074887 | 0.039361 | 0.439408 |
| Gas6 | growth arrest specific 6 | 37.2669953 | 49.26673109 | -0.418631224 | 0.000387 | 0.04091 |
| Grik1 | glutamate receptor, ionotropic, kainate 1 | 1.599308156 | 2.119084057 | -0.419772509 | 0.01628 | 0.312811 |
| Dmpk | dystrophia myotonica-protein kinase | 2.822495625 | 3.738356532 | -0.420211092 | 0.008984 | 0.236504 |
| Camk2n2 | calcium/calmodulin-dependent protein kina | 58.7944231 | 77.82077055 | -0.420811513 | 0.01901 | 0.327873 |
| Ankrd34b | ankyrin repeat domain 34B | 2.52914074 | 3.34918064 | -0.421801163 | 0.001978 | 0.111379 |
| Cyb561 | cytochrome b-561 | 10.50893909 | 13.93254537 | -0.422703353 | 0.0013 | 0.086483 |
| Hdac11 | histone deacetylase 11 | 27.92487492 | 37.08680023 | -0.424558703 | 0.000143 | 0.022479 |
| 6330403K( | RIKEN cDNA 6330403K07 gene | 73.73516646 | 98.07762484 | -0.425467772 | 0.00039 | 0.04091 |
| Ddt | D-dopachrome tautomerase | 7.732593115 | 10.29016863 | -0.425883277 | 0.007761 | 0.222945 |
| Lypd6b | LY6/PLAUR domain containing 6B | 2.734797958 | 3.641988859 | -0.428419363 | 0.026198 | 0.373263 |
| Col25a1 | collagen, type XXV, alpha 1 | 3.037078561 | 4.048491579 | -0.429808201 | 0.003833 | 0.156047 |
| Dpm1 | dolichol-phosphate (beta-D) mannosyltransf | 0.893010884 | 1.193704861 | -0.42987321 | 0.034444 | 0.414161 |
| Rspo3 | R-spondin 3 | 2.475312557 | 3.308996242 | -0.430942572 | 0.03079 | 0.399067 |
| Thnsl2 | threonine synthase-like 2 (bacterial) | 1.684424385 | 2.252016316 | -0.432216078 | 0.028179 | 0.382668 |
| Kcne1l | potassium voltage-gated channel, Isk-related | 4.374792505 | 5.85256312 | -0.434161316 | 0.046076 | 0.467042 |
| Plekhb2 | pleckstrin homology domain containing, fam | 21.29571556 | 28.4380885 | -0.434169966 | 0.003104 | 0.13957 |
| Fam149a | family with sequence similarity 149, member | 3.013314814 | 4.025160972 | -0.434866314 | 0.022717 | 0.355623 |
| Sh3bgrl2 | SH3 domain binding glutamic acid-rich prote | 2.979904919 | 3.988083683 | -0.435315264 | 0.007864 | 0.22354 |
| Ache | acetylcholinesterase | 9.446885319 | 12.65012549 | -0.436296914 | 0.000909 | 0.068802 |
| Edn3 | endothelin 3 | 2.297715642 | 3.078124352 | -0.437197011 | 0.001882 | 0.107364 |
| Polr2m | polymerase (RNA) II (DNA directed) polypepti | 48.17162665 | 64.52746241 | -0.437937538 | 0.008907 | 0.236504 |
| Zcwpw1 | zinc finger, CW type with PWWP domain 1 | 0.700844109 | 0.939281323 | -0.438220892 | 0.016848 | 0.315592 |
| Tmem179 | transmembrane protein 179 | 36.03097003 | 48.30117946 | -0.438234205 | 5.44E-05 | 0.012624 |
| Ppcdc | phosphopantothenoylcysteine decarboxylas | 1.587108356 | 2.132644662 | -0.438572796 | 0.002396 | 0.121348 |
| Pcgf2 | polycomb group ring finger 2 | 3.259229454 | 4.378864242 | -0.438983989 | 0.003481 | 0.146404 |
| Unc5d | unc-5 netrin receptor D | 1.526447607 | 2.048467075 | -0.44063089 | 0.000836 | 0.065202 |
| BC034090 | cDNA sequence BC034090 | 0.928772064 | 1.246125119 | -0.442157633 | 0.026815 | 0.376367 |
| Meg3 | maternally expressed 3 | 41.27296121 | 55.6690133 | -0.444107168 | 0.016622 | 0.31404 |
| Plekha7 | pleckstrin homology domain containing, fam | 1.117398252 | 1.503512598 | -0.444307904 | 0.010958 | 0.26209 |
| Fbxl12 | F-box and leucine-rich repeat protein 12 | 1.104163352 | 1.490316803 | -0.446748182 | 0.014363 | 0.294051 |
| Zbtb11os1 | zinc finger and BTB domain containing 11, op | 2.70381758 | 3.656417788 | -0.446801006 | 0.041336 | 0.449122 |
| Vwc2l | von Willebrand factor C domain-containing p | 0.643230057 | 0.866654372 | -0.446882085 | 0.018149 | 0.322491 |
| Mrgpre | MAS-related GPR, member E | 1.62883277 | 2.195209798 | -0.449294937 | 0.018395 | 0.324251 |
| Dnah1 | dynein, axonemal, heavy chain 1 | 0.407280836 | 0.552403588 | -0.452276101 | 0.013703 | 0.288529 |
| Nme6 | NME/NM23 nucleoside diphosphate kinase 6 | 1.766165887 | 2.401103152 | -0.454159686 | 0.025614 | 0.371302 |
| Kcnk9 | potassium channel, subfamily K, member 9 | 2.779781373 | 3.767794714 | -0.454582436 | 0.000633 | 0.055207 |
| Il33 | interleukin 33 | 5.812169957 | 7.873748286 | -0.455313941 | 0.023883 | 0.363225 |
| Itih3 | inter-alpha trypsin inhibitor, heavy chain 3 | 6.920121383 | 9.380981912 | -0.455740592 | 0.042759 | 0.449801 |

| | | | | | | |
|---|---|---|---|---|---|---|
| Lum | lumican | 2.165151291 | 2.939169161 | -0.457599171 | 0.049286 | 0.477865 |
| Oprl1 | opioid receptor-like 1 | 7.557237423 | 10.29753389 | -0.46103295 | 0.000536 | 0.049969 |
| Mirg | miRNA containing gene | 1.06610709 | 1.461770117 | -0.467506467 | 0.01173 | 0.270195 |
| Necab2 | N-terminal EF-hand calcium binding protein | 35.33784699 | 48.40097317 | -0.468830362 | 5.81E-05 | 0.012806 |
| Abca8a | ATP-binding cassette, sub-family A (ABC1), m | 0.822453625 | 1.130008883 | -0.469484218 | 0.042241 | 0.449801 |
| Slc27a2 | solute carrier family 27 (fatty acid transporte | 0.724991668 | 0.994286644 | -0.470678034 | 0.041399 | 0.449122 |
| Enpp1 | ectonucleotide pyrophosphatase/phosphodi | 0.616826719 | 0.846180543 | -0.473307984 | 0.048716 | 0.475516 |
| Rit2 | Ras-like without CAAX 2 | 10.99398649 | 15.10495496 | -0.473321448 | 0.00063 | 0.055207 |
| Cdc42ep3 | CDC42 effector protein (Rho GTPase binding) | 2.153374696 | 2.963539265 | -0.473810051 | 0.012381 | 0.276598 |
| Col6a2 | collagen, type VI, alpha 2 | 1.288423518 | 1.770121024 | -0.474165066 | 0.031481 | 0.402699 |
| Cxxc4 | CXXC finger 4 | 1.306632337 | 1.801124071 | -0.478726733 | 0.007875 | 0.22354 |
| Ccdc151 | coiled-coil domain containing 151 | 1.292902602 | 1.784684607 | -0.4797956 | 0.017193 | 0.316008 |
| Esrrg | estrogen-related receptor gamma | 2.017426721 | 2.7819722 | -0.479945903 | 0.01762 | 0.318417 |
| Zdbf2 | zinc finger, DBF-type containing 2 | 1.389886107 | 1.915736724 | -0.480316769 | 0.000525 | 0.049955 |
| Sox1ot | Sox1 overlapping transcript | 2.165051131 | 2.985761803 | -0.480756185 | 0.00209 | 0.115461 |
| Rasgrp2 | RAS, guanyl releasing protein 2 | 0.930767795 | 1.286493791 | -0.481062785 | 0.002466 | 0.123592 |
| Trmt9b | tRNA methyltransferase 9B | 1.09733828 | 1.520267915 | -0.482179537 | 0.002826 | 0.133079 |
| Zfp688 | zinc finger protein 688 | 2.441571652 | 3.379621383 | -0.482783827 | 0.004194 | 0.163555 |
| Nkd2 | naked cuticle 2 | 1.902363539 | 2.628913359 | -0.483036533 | 0.00226 | 0.119646 |
| Dusp26 | dual specificity phosphatase 26 (putative) | 20.83625955 | 28.82211645 | -0.484469517 | 0.007319 | 0.217916 |
| Ankrd24 | ankyrin repeat domain 24 | 2.430957782 | 3.371580815 | -0.485041247 | 0.000335 | 0.038099 |
| Gad2 | glutamic acid decarboxylase 2 | 16.84140356 | 23.34687718 | -0.485517559 | 0.003296 | 0.142987 |
| Tsku | tsukushi, small leucine rich proteoglycan | 0.854569222 | 1.183440758 | -0.48616944 | 0.034708 | 0.414579 |
| Eif2s3x | eukaryotic translation initiation factor 2, sub | 13.84090816 | 19.17273165 | -0.486218969 | 0.001583 | 0.097312 |
| Cdh13 | cadherin 13 | 3.03217413 | 4.202301779 | -0.486399871 | 0.000232 | 0.029615 |
| Spats2l | spermatogenesis associated, serine-rich 2-lik | 1.685280431 | 2.339443686 | -0.486592165 | 0.000396 | 0.041268 |
| Htr3a | 5-hydroxytryptamine (serotonin) receptor 3A | 2.889294861 | 4.014815241 | -0.486855862 | 0.031616 | 0.403838 |
| Dhrs11 | dehydrogenase/reductase (SDR family) memb | 2.696272488 | 3.744253956 | -0.488352606 | 0.01003 | 0.250767 |
| Cryzl2 | crystallin zeta like 2 | 3.510385181 | 4.879250116 | -0.489041187 | 0.008509 | 0.232553 |
| Ly6h | lymphocyte antigen 6 complex, locus H | 43.63473654 | 60.64099299 | -0.489187285 | 0.00086 | 0.066489 |
| 1700037H( | RIKEN cDNA 1700037H04 gene | 12.30634912 | 17.1313415 | -0.490655562 | 0.000665 | 0.056816 |
| Sparc | secreted acidic cysteine rich glycoprotein | 198.5887423 | 276.2073342 | -0.492397224 | 0.004594 | 0.170132 |
| 9030407P2 | RIKEN cDNA 9030407P20 gene | 1.175866602 | 1.643290558 | -0.49307376 | 0.033109 | 0.411041 |
| Xylb | xylulokinase homolog (H. influenzae) | 0.864910047 | 1.20517455 | -0.493727102 | 0.016782 | 0.315592 |
| Gm4419 | predicted gene 4419 | 1.016524675 | 1.418264409 | -0.494532918 | 0.035776 | 0.42352 |
| Klf5 | Kruppel-like factor 5 | 0.878242726 | 1.224788792 | -0.494905263 | 0.022364 | 0.353445 |
| Gpank1 | G patch domain and ankyrin repeats 1 | 3.253002955 | 4.543747016 | -0.495750714 | 0.006926 | 0.210497 |
| Gpc3 | glypican 3 | 1.540320425 | 2.148120626 | -0.495897096 | 0.033817 | 0.411285 |
| Rhov | ras homolog family member V | 2.670323334 | 3.727587884 | -0.496018817 | 0.01897 | 0.327826 |

| | | | | | | |
|---|---|---|---|---|---|---|
| Tmem132e | transmembrane protein 132E | 1.778141541 | 2.480231452 | -0.496159936 | 0.01378 | 0.289375 |
| Impa2 | inositol (myo)-1(or 4)-monophosphatase 2 | 1.721629267 | 2.406199534 | -0.498715373 | 0.043769 | 0.454367 |
| Oxld1 | oxidoreductase like domain containing 1 | 5.4738525 | 7.679556984 | -0.49893218 | 0.012946 | 0.28183 |
| Wdr6 | WD repeat domain 6 | 23.72905284 | 33.18232961 | -0.499952879 | 6.65E-05 | 0.013786 |
| Zbtb8b | zinc finger and BTB domain containing 8b | 0.538184244 | 0.754274107 | -0.500460085 | 0.043642 | 0.453848 |
| Dleu7 | deleted in lymphocytic leukemia, 7 | 2.463152809 | 3.473972355 | -0.506889624 | 0.011081 | 0.26378 |
| A530058N | RIKEN cDNA A530058N18 gene | 0.429789503 | 0.605078734 | -0.507417726 | 0.049858 | 0.479724 |
| Ldb2 | LIM domain binding 2 | 6.703408548 | 9.448214885 | -0.509662685 | 0.000585 | 0.052503 |
| Ccdc65 | coiled-coil domain containing 65 | 1.950294587 | 2.743390946 | -0.509941697 | 0.006434 | 0.203601 |
| Katnal2 | katanin p60 subunit A-like 2 | 0.955706926 | 1.349921636 | -0.513804822 | 0.024368 | 0.363714 |
| Slc5a5 | solute carrier family 5 (sodium iodide sympo | 4.586157643 | 6.49636061 | -0.515162721 | 0.0009 | 0.06839 |
| Kcnt1 | potassium channel, subfamily T, member 1 | 2.799980099 | 3.960870782 | -0.515201481 | 0.003778 | 0.154715 |
| Crhbp | corticotropin releasing hormone binding pro | 7.294422583 | 10.33601749 | -0.516178941 | 0.003476 | 0.146404 |
| 2010320M | RIKEN cDNA 2010320M18 gene | 5.467851447 | 7.775893145 | -0.519135059 | 0.01924 | 0.329593 |
| Ier3 | immediate early response 3 | 3.399816459 | 4.82993956 | -0.521064124 | 0.040473 | 0.444433 |
| Vstm2l | V-set and transmembrane domain containing | 39.42484308 | 56.04056096 | -0.521551298 | 1.49E-06 | 0.000909 |
| Kctd9 | potassium channel tetramerisation domain c | 1.820187916 | 2.586708116 | -0.522390103 | 0.003093 | 0.13957 |
| Slc26a11 | solute carrier family 26, member 11 | 1.60186501 | 2.279852258 | -0.522779498 | 0.004397 | 0.165947 |
| Gm37090 | predicted gene, 37090 | 0.686873123 | 0.981492413 | -0.5233924 | 0.049793 | 0.479614 |
| Bst2 | bone marrow stromal cell antigen 2 | 6.918847339 | 9.846561626 | -0.52430206 | 0.045292 | 0.462098 |
| Cfap69 | cilia and flagella associated protein 69 | 0.896871518 | 1.278914677 | -0.526566841 | 0.015805 | 0.307705 |
| Gprasp2 | G protein-coupled receptor associated sortin | 12.88080675 | 18.40157275 | -0.529019029 | 3.50E-08 | 5.15E-05 |
| Nmnat1 | nicotinamide nucleotide adenylyltransferase | 1.366832395 | 1.949289523 | -0.529063537 | 0.003892 | 0.157718 |
| Cradd | CASP2 and RIPK1 domain containing adaptor | 0.788748028 | 1.130160796 | -0.531204684 | 0.029593 | 0.391618 |
| Ttc12 | tetratricopeptide repeat domain 12 | 0.677593634 | 0.97111223 | -0.531490636 | 0.017591 | 0.318417 |
| Tox2 | TOX high mobility group box family member | 4.794326059 | 6.862062444 | -0.53322817 | 0.001069 | 0.076601 |
| Hfe | hemochromatosis | 1.930161392 | 2.761922573 | -0.534380045 | 0.022048 | 0.351135 |
| Tex9 | testis expressed gene 9 | 0.210463509 | 0.302296511 | -0.53543827 | 0.041115 | 0.447916 |
| 3-Mar | membrane-associated ring finger (C3HC4) 3 | 0.409081508 | 0.588730536 | -0.537848692 | 0.024617 | 0.365666 |
| Tspan33 | tetraspanin 33 | 3.843146899 | 5.52138198 | -0.538924472 | 0.000217 | 0.028228 |
| Renbp | renin binding protein | 1.244839566 | 1.788720018 | -0.539471926 | 0.02686 | 0.376367 |
| Gm4285 | predicted gene 4285 | 2.346966041 | 3.380226716 | -0.540169758 | 0.039827 | 0.441913 |
| Met | met proto-oncogene | 0.443171904 | 0.635207045 | -0.540189568 | 0.044259 | 0.456743 |
| Fam161a | family with sequence similarity 161, member | 0.854869887 | 1.230517531 | -0.543749699 | 0.029741 | 0.392694 |
| Aebp1 | AE binding protein 1 | 4.212801662 | 6.074501626 | -0.544186904 | 0.026301 | 0.373273 |
| A730063M | RIKEN cDNA A730063M14 gene | 0.947458414 | 1.366906255 | -0.544450249 | 0.037065 | 0.428419 |
| Mir124-2h | Mir124-2 host gene (non-protein coding) | 2.486524874 | 3.590799062 | -0.544622627 | 0.017245 | 0.316008 |
| Car14 | carbonic anhydrase 14 | 4.014326217 | 5.789459425 | -0.544870858 | 0.042751 | 0.449801 |
| Ifi27 | interferon, alpha-inducible protein 27 | 17.54828041 | 25.34708769 | -0.545556005 | 0.008179 | 0.227225 |

| | | | | | | |
|---|---|---|---|---|---|---|
| Dync2li1 | dynein cytoplasmic 2 light intermediate chai | 9.357559179 | 13.53282646 | -0.546606193 | 4.55E-05 | 0.011625 |
| Tfr2 | transferrin receptor 2 | 1.071762783 | 1.553362318 | -0.54915039 | 0.003672 | 0.152278 |
| Chst8 | carbohydrate (N-acetylgalactosamine 4-0) su | 1.935193388 | 2.806171578 | -0.550935213 | 0.000145 | 0.022492 |
| Spata6 | spermatogenesis associated 6 | 0.751531836 | 1.095182889 | -0.555739969 | 0.014462 | 0.295459 |
| Coa4 | cytochrome c oxidase assembly factor 4 | 1.062651162 | 1.551140133 | -0.557612757 | 0.014107 | 0.290324 |
| Adgra1 | adhesion G protein-coupled receptor A1 | 7.598833312 | 11.06670618 | -0.558213156 | 9.68E-05 | 0.017426 |
| Gap43 | growth associated protein 43 | 81.38834168 | 118.6227977 | -0.559311264 | 1.66E-06 | 0.000917 |
| Bmp6 | bone morphogenetic protein 6 | 3.362545656 | 4.904794678 | -0.55983115 | 0.007464 | 0.219002 |
| Abhd14b | abhydrolase domain containing 14b | 1.578480647 | 2.308533883 | -0.561281968 | 0.002612 | 0.127172 |
| Gjd2 | gap junction protein, delta 2 | 1.852677454 | 2.709467746 | -0.563899845 | 0.001317 | 0.086969 |
| Rsph9 | radial spoke head 9 homolog (Chlamydomon | 5.989347975 | 8.77650518 | -0.5642719 | 0.000479 | 0.046785 |
| Flnc | filamin C, gamma | 0.371493918 | 0.54560426 | -0.568896135 | 0.020465 | 0.338405 |
| 4930539E( | RIKEN cDNA 4930539E08 gene | 0.702650316 | 1.033965385 | -0.569008667 | 0.025586 | 0.371193 |
| Spef2 | sperm flagellar 2 | 0.280566368 | 0.410485186 | -0.570109008 | 0.047875 | 0.473834 |
| Mpp7 | membrane protein, palmitoylated 7 (MAGUK | 0.741793531 | 1.091671913 | -0.574504048 | 0.043823 | 0.454496 |
| Rom1 | rod outer segment membrane protein 1 | 2.701168373 | 3.98816392 | -0.575754777 | 0.020374 | 0.337222 |
| Prelp | proline arginine-rich end leucine-rich repeat | 5.56976163 | 8.217358258 | -0.576960002 | 0.000309 | 0.036069 |
| Grb10 | growth factor receptor bound protein 10 | 2.268263501 | 3.351663627 | -0.578300371 | 0.002007 | 0.112681 |
| Trim66 | tripartite motif-containing 66 | 0.856926826 | 1.2656073 | -0.579323646 | 0.02806 | 0.382242 |
| Tac1 | tachykinin 1 | 2.173922351 | 3.222551873 | -0.580525666 | 0.000918 | 0.069185 |
| Ccdc114 | coiled-coil domain containing 114 | 0.548459212 | 0.812193843 | -0.582252939 | 0.02895 | 0.38834 |
| Wnt4 | wingless-type MMTV integration site family, r | 4.542639296 | 6.728512447 | -0.582278156 | 2.76E-05 | 0.007969 |
| Aox1 | aldehyde oxidase 1 | 0.419117733 | 0.62089901 | -0.583266152 | 0.01111 | 0.26378 |
| Nog | noggin | 3.188041777 | 4.729562254 | -0.585873088 | 0.002288 | 0.11979 |
| Rftn1 | raftlin lipid raft linker 1 | 2.142545315 | 3.186573272 | -0.586069661 | 1.50E-05 | 0.005312 |
| Ccdc160 | coiled-coil domain containing 160 | 1.182758933 | 1.759573113 | -0.58612594 | 0.042614 | 0.449801 |
| Hsf4 | heat shock transcription factor 4 | 1.704759759 | 2.533927675 | -0.586152168 | 0.013111 | 0.2831 |
| Mum1l1 | melanoma associated antigen (mutated) 1-lik | 0.558013182 | 0.828608203 | -0.58725374 | 0.045018 | 0.461155 |
| 1700037C1 | RIKEN cDNA 1700037C18 gene | 0.671371478 | 0.997748999 | -0.587509222 | 0.024308 | 0.363714 |
| Dpp10 | dipeptidylpeptidase 10 | 2.172726661 | 3.228408419 | -0.588162002 | 0.00283 | 0.133079 |
| Cdkn1c | cyclin-dependent kinase inhibitor 1C (P57) | 4.177929648 | 6.22537764 | -0.591618432 | 0.006224 | 0.200208 |
| Peg10 | paternally expressed 10 | 2.1205487 | 3.165895029 | -0.593346016 | 2.88E-06 | 0.001396 |
| Meis1 | Meis homeobox 1 | 0.227850667 | 0.340548838 | -0.594885517 | 0.025882 | 0.37282 |
| Kcnh2 | potassium voltage-gated channel, subfamily I | 3.987021506 | 5.961582945 | -0.595761434 | 0.001311 | 0.086853 |
| Cdr2 | cerebellar degeneration-related 2 | 1.698769231 | 2.535385968 | -0.596118322 | 0.020234 | 0.336853 |
| Timp2 | tissue inhibitor of metalloproteinase 2 | 53.60946192 | 80.18733496 | -0.597549325 | 2.58E-05 | 0.007829 |
| Stk32b | serine/threonine kinase 32B | 1.2099069 | 1.818188276 | -0.600576247 | 0.000964 | 0.071692 |
| Smyd1 | SET and MYND domain containing 1 | 0.786724823 | 1.178656914 | -0.601035327 | 0.033208 | 0.411061 |
| Krt2 | keratin 2 | 1.281329439 | 1.930992483 | -0.605210233 | 0.01951 | 0.331313 |

| | | | | | | |
|---|---|---|---|---|---|---|
| Nnat | neuronatin | 156.8007222 | 236.3142769 | -0.606291353 | 0.000406 | 0.042135 |
| Ano2 | anoctamin 2 | 0.379452951 | 0.569461614 | -0.606527328 | 0.037677 | 0.431765 |
| Nudt14 | nudix (nucleoside diphosphate linked moiety | 3.175259323 | 4.80348789 | -0.609935629 | 0.00466 | 0.171304 |
| Fam131c | family with sequence similarity 131, member | 1.804650553 | 2.72918075 | -0.611162424 | 0.006506 | 0.204794 |
| Wdr78 | WD repeat domain 78 | 0.733807248 | 1.111904375 | -0.61270499 | 0.013367 | 0.286425 |
| Myof | myoferlin | 0.430548345 | 0.650456978 | -0.614114021 | 0.014678 | 0.297056 |
| Gabra3 | gamma-aminobutyric acid (GABA) A receptor | 9.325574714 | 14.10859489 | -0.614275466 | 5.91E-05 | 0.012806 |
| Kif6 | kinesin family member 6 | 0.302309723 | 0.459899518 | -0.615250463 | 0.018643 | 0.326981 |
| Zfp423 | zinc finger protein 423 | 2.851896609 | 4.325594042 | -0.616501098 | 0.00187 | 0.107364 |
| Epb41l4a | erythrocyte membrane protein band 4.1 like | 1.310535531 | 1.990690916 | -0.619000219 | 0.000298 | 0.035408 |
| Cacna2d2 | calcium channel, voltage-dependent, alpha 2 | 2.623027645 | 3.979206998 | -0.619185399 | 0.007113 | 0.213581 |
| Rxfp3 | relaxin family peptide receptor 3 | 0.897537925 | 1.36172214 | -0.620045824 | 0.035142 | 0.417415 |
| Sytl4 | synaptotagmin-like 4 | 0.379923734 | 0.580099048 | -0.621429082 | 0.045219 | 0.461802 |
| Cfap43 | cilia and flagella associated protein 43 | 0.405662658 | 0.617611618 | -0.621813574 | 0.016072 | 0.310485 |
| C8g | complement component 8, gamma polypept | 0.882941158 | 1.34926162 | -0.622337277 | 0.042134 | 0.449801 |
| Dlx5 | distal-less homeobox 5 | 0.909675887 | 1.384804283 | -0.623959352 | 0.018795 | 0.327337 |
| Rpp25 | ribonuclease P/MRP 25 subunit | 5.428732464 | 8.273803407 | -0.624129706 | 0.000176 | 0.024665 |
| Gprin2 | G protein regulated inducer of neurite outgr | 0.256295391 | 0.391086195 | -0.62489826 | 0.020117 | 0.335484 |
| Mns1 | meiosis-specific nuclear structural protein 1 | 0.531276896 | 0.810785758 | -0.628075948 | 0.015939 | 0.309428 |
| Akip1 | A kinase (PRKA) interacting protein 1 | 1.95484457 | 2.998545427 | -0.63138898 | 0.026245 | 0.373273 |
| Lrguk | leucine-rich repeats and guanylate kinase dor | 0.19276625 | 0.29515259 | -0.632459058 | 0.007778 | 0.222945 |
| Cntnap3 | contactin associated protein-like 3 | 0.436001902 | 0.666897022 | -0.633455114 | 0.026112 | 0.373081 |
| Drc3 | dynein regulatory complex subunit 3 | 1.34382462 | 2.073159983 | -0.639084823 | 0.001754 | 0.103969 |
| Camk2d | calcium/calmodulin-dependent protein kina | 1.570691723 | 2.425599381 | -0.642190044 | 0.001925 | 0.10908 |
| 4732491K2 | RIKEN cDNA 4732491K20 gene | 0.537069936 | 0.834261977 | -0.642283375 | 0.041405 | 0.449122 |
| Hs3st2 | heparan sulfate (glucosamine) 3-O-sulfotrans | 2.243149159 | 3.459771946 | -0.645763683 | 0.013106 | 0.2831 |
| Dnah10 | dynein, axonemal, heavy chain 10 | 0.144152203 | 0.223453698 | -0.649092109 | 0.037751 | 0.432069 |
| Spata24 | spermatogenesis associated 24 | 3.282932531 | 5.114492554 | -0.652673041 | 0.006081 | 0.197766 |
| Npr3 | natriuretic peptide receptor 3 | 1.358337658 | 2.112961012 | -0.653860363 | 0.00053 | 0.049955 |
| Gm19744 | predicted gene, 19744 | 0.234780841 | 0.365559772 | -0.654410455 | 0.043573 | 0.453419 |
| Hs6st2 | heparan sulfate 6-O-sulfotransferase 2 | 2.194727169 | 3.416115946 | -0.654778323 | 5.04E-06 | 0.002279 |
| Emb | embigin | 2.35550378 | 3.670904198 | -0.656079689 | 0.001137 | 0.078396 |
| Gm19531 | predicted gene, 19531 | 1.81155821 | 2.838803525 | -0.656870546 | 0.027821 | 0.381825 |
| Mme | membrane metallo endopeptidase | 0.337765713 | 0.52770926 | -0.65839126 | 0.000593 | 0.052797 |
| Gm973 | predicted gene 973 | 1.140712401 | 1.781585212 | -0.659556434 | 0.004238 | 0.164711 |
| E430024P1 | RIKEN cDNA E430024P14 gene | 0.281522367 | 0.441550797 | -0.659733023 | 0.040806 | 0.446692 |
| 5730414N1 | RIKEN cDNA 5730414N17 gene | 0.88138136 | 1.382906168 | -0.662134086 | 0.046493 | 0.469649 |
| Zc2hc1c | zinc finger, C2HC-type containing 1C | 0.413646463 | 0.648184905 | -0.664209734 | 0.037359 | 0.42985 |
| Cpne2 | copine II | 9.183744922 | 14.44377396 | -0.668329264 | 8.30E-05 | 0.01642 |

| | | | | | | |
|---|---|---|---|---|---|---|
| Tmem130 | transmembrane protein 130 | 31.70711041 | 49.91976983 | -0.669677726 | 3.63E-07 | 0.000278 |
| Tcerg1l | transcription elongation regulator 1-like | 1.876941379 | 2.955126992 | -0.669943902 | 5.33E-05 | 0.012524 |
| Itga10 | integrin, alpha 10 | 0.333561986 | 0.524705297 | -0.675026235 | 0.024371 | 0.363714 |
| BC064078 | cDNA sequence BC064078 | 0.938077406 | 1.482427741 | -0.675782503 | 0.010086 | 0.251107 |
| Wdr66 | WD repeat domain 66 | 0.30947054 | 0.489229705 | -0.676860429 | 0.009083 | 0.237092 |
| Pbx3 | pre B cell leukemia homeobox 3 | 1.000348646 | 1.584418324 | -0.678140068 | 0.016866 | 0.315592 |
| Cd55 | CD55 molecule, decay accelerating factor for | 0.992357871 | 1.574268962 | -0.679758036 | 0.000928 | 0.069592 |
| Podn | podocan | 0.453845267 | 0.720440547 | -0.680447202 | 0.017643 | 0.318417 |
| Aifm2 | apoptosis-inducing factor, mitochondrion-as | 1.079764205 | 1.711796383 | -0.680652238 | 0.012156 | 0.274364 |
| AF529169 | cDNA sequence AF529169 | 0.262612942 | 0.417508054 | -0.683026591 | 0.037192 | 0.429183 |
| Pcsk4 | proprotein convertase subtilisin/kexin type 4 | 0.609882366 | 0.972185917 | -0.683684499 | 0.033872 | 0.411285 |
| Nov | nephroblastoma overexpressed gene | 23.51558021 | 37.41803089 | -0.684087758 | 0.027417 | 0.379505 |
| Tacr1 | tachykinin receptor 1 | 0.244368661 | 0.390328143 | -0.690965836 | 0.033648 | 0.411285 |
| Nap1l5 | nucleosome assembly protein 1-like 5 | 105.700018 | 169.2528015 | -0.693969244 | 1.11E-05 | 0.004358 |
| mt-Ts2 | mitochondrially encoded tRNA serine 2 | 63.42233483 | 101.6053907 | -0.695293864 | 0.011889 | 0.271429 |
| Nppc | natriuretic peptide type C | 1.826136039 | 2.925215081 | -0.695411832 | 0.007599 | 0.220817 |
| Dlx2 | distal-less homeobox 2 | 0.911255273 | 1.461646472 | -0.697290293 | 0.019614 | 0.332118 |
| Htr7 | 5-hydroxytryptamine (serotonin) receptor 7 | 2.564234728 | 4.107078039 | -0.69731806 | 0.001025 | 0.074646 |
| Col5a3 | collagen, type V, alpha 3 | 0.315888398 | 0.50926728 | -0.698869343 | 0.017787 | 0.319281 |
| Ccdc162 | coiled-coil domain containing 162 | 0.208430443 | 0.335191647 | -0.699026609 | 0.041087 | 0.447916 |
| Gm9794 | predicted pseudogene 9794 | 2.656938042 | 4.278762467 | -0.699428061 | 0.02786 | 0.381871 |
| Nhs | NHS actin remodeling regulator | 0.346270665 | 0.556905519 | -0.704233727 | 0.020314 | 0.336853 |
| Pcp4l1 | Purkinje cell protein 4-like 1 | 34.84133257 | 56.26653787 | -0.706951421 | 0.047375 | 0.47273 |
| Cab39l | calcium binding protein 39-like | 3.940562685 | 6.359985771 | -0.707522495 | 0.036585 | 0.426227 |
| Plagl1 | pleiomorphic adenoma gene-like 1 | 1.229816438 | 1.988936952 | -0.710768171 | 0.001377 | 0.088894 |
| Enkur | enkurin, TRPC channel interacting protein | 1.568029343 | 2.540080828 | -0.711037437 | 0.015584 | 0.307181 |
| Arhgap36 | Rho GTPase activating protein 36 | 0.511285936 | 0.828011143 | -0.712820845 | 0.026719 | 0.376183 |
| Radil | Ras association and DIL domains | 1.302182596 | 2.111382925 | -0.713970007 | 1.42E-05 | 0.005312 |
| Mst1r | macrophage stimulating 1 receptor (c-met-re | 0.327256514 | 0.531796869 | -0.717107996 | 0.033915 | 0.411285 |
| Vwc2 | von Willebrand factor C domain containing 2 | 0.903022919 | 1.470140779 | -0.718112259 | 0.015597 | 0.307181 |
| Kdelr3 | KDEL (Lys-Asp-Glu-Leu) endoplasmic reticulu | 0.84155576 | 1.37402426 | -0.720709981 | 0.042729 | 0.449801 |
| Cfap65 | cilia and flagella associated protein 65 | 0.349066959 | 0.569338128 | -0.721774288 | 0.024792 | 0.36601 |
| Efna5 | ephrin A5 | 0.997907354 | 1.63510885 | -0.726712896 | 0.001892 | 0.107597 |
| Ubxn11 | UBX domain protein 11 | 1.289698046 | 2.12486443 | -0.73356296 | 0.000805 | 0.064539 |
| Rnf207 | ring finger protein 207 | 0.202980553 | 0.334043946 | -0.733874662 | 0.01532 | 0.304034 |
| Scml4 | Scm polycomb group protein like 4 | 0.366671297 | 0.602686525 | -0.734682167 | 0.003157 | 0.140191 |
| Gm15417 | predicted gene 15417 | 1.442253838 | 2.384801553 | -0.734826559 | 0.015961 | 0.309501 |
| Kif9 | kinesin family member 9 | 0.876025465 | 1.446340132 | -0.738974081 | 0.007175 | 0.214723 |
| Bace2 | beta-site APP-cleaving enzyme 2 | 0.736932739 | 1.219653032 | -0.739260047 | 0.000257 | 0.031631 |

| | | | | | | |
|---|---|---|---|---|---|---|
| Myoc | myocilin | 11.12925373 | 18.39643146 | -0.741358287 | 0.001121 | 0.078396 |
| D830030K2 | RIKEN cDNA D830030K20 gene | 0.623788961 | 1.030695343 | -0.743591948 | 0.015334 | 0.304034 |
| Cdh7 | cadherin 7, type 2 | 0.382847036 | 0.634467938 | -0.744617375 | 0.016491 | 0.31404 |
| Rtl1 | retrotransposon Gaglike 1 | 0.372508346 | 0.623598425 | -0.751405769 | 0.02355 | 0.36066 |
| Slc32a1 | solute carrier family 32 (GABA vesicular trans | 15.60571857 | 25.99991998 | -0.752009707 | 9.95E-08 | 0.00011 |
| Rdh5 | retinol dehydrogenase 5 | 1.14844027 | 1.915396216 | -0.755456044 | 0.038361 | 0.434618 |
| Col9a1 | collagen, type IX, alpha 1 | 0.233608741 | 0.390562711 | -0.756228786 | 0.020861 | 0.341521 |
| Grp | gastrin releasing peptide | 12.65621791 | 21.199398 | -0.757768949 | 0.000651 | 0.055933 |
| Mak | male germ cell-associated kinase | 0.215201877 | 0.360999968 | -0.75850434 | 0.027265 | 0.378721 |
| Ucma | upper zone of growth plate and cartilage mat | 2.073075091 | 3.488956189 | -0.759738619 | 0.007368 | 0.218248 |
| Slc7a3 | solute carrier family 7 (cationic amino acid tr | 0.831200293 | 1.395210089 | -0.763066251 | 0.001266 | 0.085514 |
| Gm14539 | predicted gene 14539 | 9.077953125 | 15.32519222 | -0.763351324 | 0.015738 | 0.307705 |
| Gas2l2 | growth arrest-specific 2 like 2 | 0.587654785 | 0.98497238 | -0.764673669 | 0.024315 | 0.363714 |
| Kif27 | kinesin family member 27 | 0.406578305 | 0.683596018 | -0.7650933 | 0.019567 | 0.33164 |
| 4933431K1 | RIKEN cDNA 4933431K14 gene | 0.797288569 | 1.341217437 | -0.766761223 | 0.01568 | 0.307705 |
| Pcbd1 | pterin 4 alpha carbinolamine dehydratase/di | 4.294309722 | 7.249335982 | -0.767399637 | 0.001351 | 0.087896 |
| Ifit3b | interferon-induced protein with tetratricope | 1.478206523 | 2.497063785 | -0.768300438 | 0.002333 | 0.120423 |
| Pyroxd2 | pyridine nucleotide-disulphide oxidoreducta | 0.531658442 | 0.899795014 | -0.768427322 | 0.024318 | 0.363714 |
| Cthrc1 | collagen triple helix repeat containing 1 | 3.270181889 | 5.522299318 | -0.769679414 | 0.000117 | 0.019405 |
| Btbd11 | BTB (POZ) domain containing 11 | 1.256749275 | 2.119394829 | -0.771189787 | 0.000645 | 0.055691 |
| Scn7a | sodium channel, voltage-gated, type VII, alph | 0.201249124 | 0.33905373 | -0.772714199 | 0.013647 | 0.288529 |
| Rarres2 | retinoic acid receptor responder (tazarotene | 2.141099697 | 3.630374501 | -0.774578861 | 0.001336 | 0.087213 |
| Hrh3 | histamine receptor H3 | 8.811432502 | 14.94773454 | -0.777416014 | 5.28E-08 | 7.16E-05 |
| Chdh | choline dehydrogenase | 0.318063792 | 0.539973731 | -0.782161533 | 0.044852 | 0.460965 |
| Cpne7 | copine VII | 24.62643816 | 41.93830596 | -0.782399865 | 8.69E-05 | 0.016659 |
| Neil2 | nei like 2 (E. coli) | 0.196548674 | 0.33639724 | -0.782754379 | 0.031055 | 0.400448 |
| Mdfic | MyoD family inhibitor domain containing | 0.760863213 | 1.295814503 | -0.782959823 | 0.007206 | 0.215301 |
| Glp2r | glucagon-like peptide 2 receptor | 0.445492721 | 0.757436179 | -0.785583783 | 0.013178 | 0.28328 |
| Foxj1 | forkhead box J1 | 4.796673858 | 8.192364288 | -0.787033082 | 9.74E-05 | 0.017426 |
| mt-Tg | mitochondrially encoded tRNA glycine | 47.53338632 | 81.33694861 | -0.787545432 | 0.026806 | 0.376367 |
| Tmem200c | transmembrane protein 200C | 1.712209294 | 2.920697614 | -0.787742317 | 4.87E-05 | 0.012243 |
| Gpat3 | glycerol-3-phosphate acyltransferase 3 | 0.338089373 | 0.576943119 | -0.788544706 | 0.045446 | 0.462192 |
| 4933407L2 | RIKEN cDNA 4933407L21 gene | 0.489979963 | 0.840080484 | -0.79350928 | 0.013485 | 0.287077 |
| Fndc9 | fibronectin type III domain containing 9 | 0.558838877 | 0.962443607 | -0.799347548 | 0.031725 | 0.404338 |
| Dnaic2 | dynein, axonemal, intermediate chain 2 | 0.502848265 | 0.8671621 | -0.800414529 | 0.001432 | 0.091154 |
| Sst | somatostatin | 170.4991128 | 294.6314213 | -0.802112238 | 0.006477 | 0.204234 |
| Dmkn | dermokine | 0.585230051 | 1.013787915 | -0.802283612 | 0.009031 | 0.236533 |
| Casc1 | cancer susceptibility candidate 1 | 0.092128163 | 0.15992442 | -0.802479953 | 0.047538 | 0.472936 |
| Npbwr1 | neuropeptides B/W receptor 1 | 0.250837552 | 0.434837968 | -0.806297799 | 0.022377 | 0.353445 |

| | | | | | | |
|---|---|---|---|---|---|---|
| Catip | ciliogenesis associated TTC17 interacting pro | 0.885340169 | 1.539775159 | -0.807198171 | 0.013172 | 0.28328 |
| Bfsp1 | beaded filament structural protein 1, in lens- | 0.308377778 | 0.532814764 | -0.808611387 | 0.04309 | 0.450771 |
| Fgf18 | fibroblast growth factor 18 | 1.243751473 | 2.168141235 | -0.810113446 | 0.008049 | 0.225748 |
| Akr1c18 | aldo-keto reductase family 1, member C18 | 1.008924978 | 1.748728612 | -0.810127641 | 0.046384 | 0.469169 |
| Nipal2 | NIPA-like domain containing 2 | 0.363541348 | 0.630497632 | -0.811056654 | 0.001417 | 0.090523 |
| Tjp3 | tight junction protein 3 | 0.323978447 | 0.562509208 | -0.816196214 | 0.01645 | 0.31404 |
| Zfp941 | zinc finger protein 941 | 1.54634876 | 2.698223938 | -0.816944115 | 2.24E-06 | 0.001191 |
| Dlx6 | distal-less homeobox 6 | 0.905869119 | 1.574689815 | -0.817806396 | 0.002133 | 0.117104 |
| Ccdc121 | coiled-coil domain containing 121 | 0.417808187 | 0.73147831 | -0.818852185 | 0.034253 | 0.412986 |
| 6430571L1 | RIKEN cDNA 6430571L13 gene | 0.660939294 | 1.156932576 | -0.819889805 | 0.013324 | 0.28606 |
| Iqcg | IQ motif containing G | 0.457892344 | 0.800432477 | -0.821171427 | 0.002948 | 0.136672 |
| Zfp618 | zinc finger protein 618 | 0.277088431 | 0.484598853 | -0.821219986 | 0.016506 | 0.31404 |
| Clrn1 | clarin 1 | 0.183709692 | 0.321755655 | -0.824125917 | 0.047073 | 0.472522 |
| Esyt3 | extended synaptotagmin-like protein 3 | 0.125234541 | 0.220528177 | -0.8244936 | 0.021583 | 0.346799 |
| Sp9 | trans-acting transcription factor 9 | 0.83355424 | 1.471721259 | -0.836871538 | 1.11E-05 | 0.004358 |
| Tal1 | T cell acute lymphocytic leukemia 1 | 0.713223229 | 1.260391495 | -0.837283503 | 0.025076 | 0.367737 |
| Phf2os1 | PHD finger protein 2, opposite strand 1 | 1.522205981 | 2.709040245 | -0.838007299 | 0.04668 | 0.469916 |
| H2-M3 | histocompatibility 2, M region locus 3 | 0.876871146 | 1.559200625 | -0.838459597 | 0.02441 | 0.363714 |
| Gm2694 | predicted gene 2694 | 0.260485759 | 0.463284638 | -0.841529702 | 0.00781 | 0.22311 |
| Spint2 | serine protease inhibitor, Kunitz type 2 | 2.92812691 | 5.18952782 | -0.841842279 | 0.027531 | 0.379964 |
| Myh7 | myosin, heavy polypeptide 7, cardiac muscle | 0.451475292 | 0.799530244 | -0.843465156 | 8.38E-05 | 0.01642 |
| Krt77 | keratin 77 | 1.608583717 | 2.857575335 | -0.845249056 | 0.004386 | 0.165947 |
| Lhfpl5 | lipoma HMGIC fusion partner-like 5 | 0.721677906 | 1.283947951 | -0.846784039 | 0.003087 | 0.13957 |
| Efcab1 | EF-hand calcium binding domain 1 | 1.450774275 | 2.583242719 | -0.848374442 | 9.52E-05 | 0.017426 |
| Gm14204 | predicted gene 14204 | 0.496170689 | 0.884856746 | -0.850583346 | 0.000812 | 0.064539 |
| Syne4 | spectrin repeat containing, nuclear envelope | 0.410822608 | 0.739304319 | -0.852712735 | 0.037024 | 0.428229 |
| Calca | calcitonin/calcitonin-related polypeptide, al | 0.876933392 | 1.574809702 | -0.857954728 | 0.028993 | 0.38834 |
| Col6a1 | collagen, type VI, alpha 1 | 3.542221321 | 6.366698096 | -0.860866371 | 1.63E-05 | 0.005642 |
| Bc1 | brain cytoplasmic RNA 1 | 15.56394238 | 28.01580403 | -0.861654126 | 0.019407 | 0.330205 |
| Gm33869 | predicted gene, 33869 | 0.214628257 | 0.38586748 | -0.862006146 | 0.035907 | 0.424092 |
| Eno4 | enolase 4 | 0.826668419 | 1.485030127 | -0.863275541 | 0.00328 | 0.142987 |
| 1700023F0 | RIKEN cDNA 1700023F06 gene | 0.697577356 | 1.251325481 | -0.863978903 | 0.011321 | 0.264305 |
| B130034C1 | RIKEN cDNA B130034C11 gene | 0.42596743 | 0.771476757 | -0.864378542 | 0.008654 | 0.23324 |
| Col23a1 | collagen, type XXIII, alpha 1 | 1.513281557 | 2.728230769 | -0.865916148 | 1.56E-07 | 0.000145 |
| Hap1 | huntingtin-associated protein 1 | 19.69382906 | 35.57539981 | -0.867415908 | 2.69E-23 | 4.75E-19 |
| Agt | angiotensinogen (serpin peptidase inhibitor, | 17.14672174 | 30.94177388 | -0.867970058 | 0.02065 | 0.339596 |
| Nr2f2 | nuclear receptor subfamily 2, group F, memb | 2.387302854 | 4.335217395 | -0.874897576 | 7.48E-08 | 9.41E-05 |
| Klc3 | kinesin light chain 3 | 0.340358246 | 0.619447358 | -0.879889787 | 0.02985 | 0.3931 |
| Drd2 | dopamine receptor D2 | 0.887822008 | 1.620631099 | -0.880829027 | 0.006633 | 0.206058 |

| | | | | | | |
|---|---|---|---|---|---|---|
| Postn | periostin, osteoblast specific factor | 0.275141649 | 0.50126093 | -0.883636715 | 0.002963 | 0.136714 |
| Ccdc113 | coiled-coil domain containing 113 | 1.145979735 | 2.097033523 | -0.886751676 | 0.014567 | 0.296217 |
| Rasa4 | RAS p21 protein activator 4 | 0.384592591 | 0.70586265 | -0.890779527 | 0.000575 | 0.051969 |
| Myo5c | myosin VC | 0.092972102 | 0.169999063 | -0.89089804 | 0.034858 | 0.415443 |
| Gpx3 | glutathione peroxidase 3 | 2.63417286 | 4.854187763 | -0.892304193 | 6.97E-06 | 0.003008 |
| Nme9 | NME/NM23 family member 9 | 0.435910057 | 0.805505029 | -0.893345137 | 0.04948 | 0.478698 |
| Armc4 | armadillo repeat containing 4 | 0.278766392 | 0.514025087 | -0.897479764 | 0.013426 | 0.287016 |
| Mmp9 | matrix metallopeptidase 9 | 0.281569065 | 0.523017424 | -0.897651117 | 0.018344 | 0.324251 |
| Iqub | IQ motif and ubiquitin domain containing | 0.409739991 | 0.760450635 | -0.901503995 | 0.015998 | 0.309549 |
| Vwa5b1 | von Willebrand factor A domain containing 5 | 0.269566507 | 0.498738142 | -0.904197993 | 0.008594 | 0.23271 |
| Npffr1 | neuropeptide FF receptor 1 | 0.724107894 | 1.342682745 | -0.905381272 | 0.000171 | 0.024665 |
| Sult5a1 | sulfotransferase family 5A, member 1 | 0.138472161 | 0.256640861 | -0.905916683 | 0.049755 | 0.479514 |
| Dlec1 | deleted in lung and esophageal cancer 1 | 0.183338431 | 0.34068929 | -0.911243817 | 0.00822 | 0.227225 |
| 4932438H2 | RIKEN cDNA 4932438H23 gene | 0.300223628 | 0.562878246 | -0.915710273 | 0.039026 | 0.437099 |
| Frmpd2 | FERM and PDZ domain containing 2 | 0.249168907 | 0.467479258 | -0.918822377 | 0.003297 | 0.142987 |
| Susd2 | sushi domain containing 2 | 0.728641613 | 1.366188485 | -0.920257002 | 0.004962 | 0.177404 |
| Capsl | calcyphosine-like | 2.766133207 | 5.204267786 | -0.922925572 | 0.001822 | 0.106678 |
| Zcchc12 | zinc finger, CCHC domain containing 12 | 10.99831234 | 20.7277989 | -0.928446503 | 2.34E-08 | 3.78E-05 |
| Cdhr3 | cadherin-related family member 3 | 0.406667367 | 0.770271578 | -0.928648651 | 0.01515 | 0.302784 |
| Mroh5 | maestro heat-like repeat family member 5 | 0.119058059 | 0.22440874 | -0.931153813 | 0.023413 | 0.360229 |
| Atg9b | autophagy related 9B | 0.484864121 | 0.917002243 | -0.931391487 | 0.000155 | 0.023623 |
| 1810044D0 | RIKEN cDNA 1810044D09 gene | 1.28636326 | 2.4459113 | -0.934908696 | 0.02209 | 0.351424 |
| Dgkk | diacylglycerol kinase kappa | 0.243723995 | 0.461608003 | -0.937931745 | 0.003621 | 0.150892 |
| Gm9905 | predicted gene 9905 | 0.250435197 | 0.478496711 | -0.942675731 | 0.042785 | 0.449801 |
| Odf3b | outer dense fiber of sperm tails 3B | 0.627376982 | 1.197825387 | -0.944395096 | 0.02355 | 0.36066 |
| Ccdc81 | coiled-coil domain containing 81 | 0.369100264 | 0.704336289 | -0.945239522 | 0.004864 | 0.175325 |
| Rxrg | retinoid X receptor gamma | 0.929953552 | 1.770744215 | -0.949386293 | 0.002115 | 0.116494 |
| Togaram2 | TOG array regulator of axonemal microtubul | 0.248030516 | 0.476497254 | -0.95481327 | 0.006555 | 0.20522 |
| Cfap44 | cilia and flagella associated protein 44 | 0.255341329 | 0.490427097 | -0.957788179 | 0.00761 | 0.220817 |
| Scn9a | sodium channel, voltage-gated, type IX, alpha | 0.131323258 | 0.252670021 | -0.959294125 | 0.00257 | 0.126208 |
| Cfap45 | cilia and flagella associated protein 45 | 0.388988597 | 0.751541156 | -0.960652145 | 0.000166 | 0.024184 |
| Gm26902 | predicted gene, 26902 | 0.153133216 | 0.296925984 | -0.962387285 | 0.028444 | 0.385093 |
| Gpr149 | G protein-coupled receptor 149 | 0.205720139 | 0.398172799 | -0.96298932 | 0.012892 | 0.281812 |
| Ppp1r3b | protein phosphatase 1, regulatory subunit 3B | 0.304185958 | 0.586950783 | -0.964168322 | 0.024268 | 0.363714 |
| BC067074 | cDNA sequence BC067074 | 0.269476023 | 0.518684484 | -0.964974143 | 0.00777 | 0.222945 |
| Oxtr | oxytocin receptor | 1.189223103 | 2.295706904 | -0.965395977 | 3.50E-05 | 0.009634 |
| Tmem91 | transmembrane protein 91 | 1.06696241 | 2.066414251 | -0.965908746 | 8.95E-05 | 0.016866 |
| Casp1 | caspase 1 | 0.543155249 | 1.05039368 | -0.972483386 | 0.012122 | 0.274296 |
| Gpr101 | G protein-coupled receptor 101 | 0.984827097 | 1.925439227 | -0.977247863 | 0.000193 | 0.026207 |

| | | | | | | |
|---|---|---|---|---|---|---|
| Gm17383 | predicted gene, 17383 | 0.609385616 | 1.186991479 | -0.977699637 | 0.033808 | 0.411285 |
| mt-Th | mitochondrially encoded tRNA histidine | 48.87974214 | 95.26164639 | -0.978264594 | 4.76E-06 | 0.00221 |
| Gm19935 | predicted gene, 19935 | 4.448033814 | 8.736084529 | -0.985495396 | 0.001448 | 0.091226 |
| 5930420M | RIKEN cDNA 5930420M18 gene | 0.346666006 | 0.680163434 | -0.987663515 | 0.007792 | 0.222975 |
| Gm16702 | predicted gene, 16702 | 0.514881957 | 1.014826618 | -0.991745911 | 0.00689 | 0.209771 |
| Wdr93 | WD repeat domain 93 | 0.18898003 | 0.37691537 | -0.998955603 | 0.047274 | 0.47273 |
| Ccdc146 | coiled-coil domain containing 146 | 0.287406161 | 0.569920013 | -1.001661054 | 0.002196 | 0.119117 |
| Gm43759 | predicted gene 43759 | 0.538295889 | 1.074509536 | -1.001854515 | 0.027696 | 0.381104 |
| Cfap126 | cilia and flagella associated protein 126 | 1.250369787 | 2.48139447 | -1.002544102 | 0.004501 | 0.168794 |
| Sbsn | suprabasin | 0.241153795 | 0.47928105 | -1.002791244 | 0.014049 | 0.289976 |
| Dnaaf3 | dynein, axonemal assembly factor 3 | 0.315968027 | 0.625451245 | -1.003823099 | 0.028822 | 0.387794 |
| Lrp2 | low density lipoprotein receptor-related pro | 0.047337861 | 0.094119208 | -1.005542242 | 0.011134 | 0.26378 |
| Efcab12 | EF-hand calcium binding domain 12 | 0.665954006 | 1.326657379 | -1.007693014 | 0.000638 | 0.055376 |
| Gpr55 | G protein-coupled receptor 55 | 0.154753506 | 0.306062968 | -1.010995665 | 0.032603 | 0.40904 |
| Ccdc187 | coiled-coil domain containing 187 | 0.207112521 | 0.414424122 | -1.014932231 | 0.010501 | 0.257446 |
| Hdc | histidine decarboxylase | 0.310552346 | 0.623581776 | -1.018204926 | 0.012083 | 0.273752 |
| Gm11992 | predicted gene 11992 | 0.623227197 | 1.253102397 | -1.018449142 | 0.002369 | 0.121033 |
| Fam181a | family with sequence similarity 181, membe | 0.578647419 | 1.169189472 | -1.02039207 | 0.018955 | 0.327826 |
| Etnppl | ethanolamine phosphate phospholyase | 1.519347969 | 3.064161475 | -1.027470399 | 0.004001 | 0.16123 |
| Cfap54 | cilia and flagella associated protein 54 | 0.187540709 | 0.378067621 | -1.03028823 | 8.64E-05 | 0.016659 |
| Crhr2 | corticotropin releasing hormone receptor 2 | 0.213066284 | 0.431541429 | -1.036235106 | 0.011134 | 0.26378 |
| Crb1 | crumbs family member 1, photoreceptor mo | 0.044497914 | 0.090703906 | -1.037821746 | 0.023745 | 0.362391 |
| BC030867 | cDNA sequence BC030867 | 0.202919186 | 0.411820074 | -1.039779728 | 0.047676 | 0.472936 |
| Xkrx | X-linked Kx blood group related, X-linked | 0.165263651 | 0.334403448 | -1.039892707 | 0.032699 | 0.409368 |
| Ppp1r36 | protein phosphatase 1, regulatory subunit 36 | 0.326737116 | 0.668733154 | -1.04131369 | 0.00894 | 0.236504 |
| Atp4a | ATPase, H+/K+ exchanging, gastric, alpha poly | 0.13380619 | 0.273955264 | -1.041336469 | 0.037893 | 0.432601 |
| Sncg | synuclein, gamma | 2.511162838 | 5.14527276 | -1.045036873 | 0.000107 | 0.018291 |
| Slc16a8 | solute carrier family 16 (monocarboxylic acid | 0.754485231 | 1.537788911 | -1.045520472 | 0.022974 | 0.357433 |
| Nrsn2 | neurensin 2 | 15.56696021 | 31.90509398 | -1.050020472 | 8.90E-07 | 0.000581 |
| Ace | angiotensin I converting enzyme (peptidyl-di | 3.309185593 | 6.767696355 | -1.051496539 | 0.04541 | 0.462192 |
| Dnah11 | dynein, axonemal, heavy chain 11 | 0.070833322 | 0.144715121 | -1.051979304 | 0.004793 | 0.1735 |
| Lrrc74b | leucine rich repeat containing 74B | 0.215140779 | 0.443174155 | -1.055329016 | 0.019539 | 0.331487 |
| Slc10a4 | solute carrier family 10 (sodium/bile acid cot | 0.252817483 | 0.523392011 | -1.060277387 | 0.041231 | 0.448909 |
| Stoml3 | stomatin (Epb7.2)-like 3 | 0.357734363 | 0.742784141 | -1.062256539 | 0.027331 | 0.379342 |
| Traf1 | TNF receptor-associated factor 1 | 0.105621153 | 0.217909806 | -1.062366647 | 0.001444 | 0.091226 |
| Kdm4d | lysine (K)-specific demethylase 4D | 0.149526317 | 0.311203287 | -1.06284793 | 0.03863 | 0.435662 |
| Cbln4 | cerebellin 4 precursor protein | 1.929133169 | 3.987824829 | -1.06368353 | 2.84E-07 | 0.000228 |
| 1700018L0 | RIKEN cDNA 1700018L02 gene | 0.217435501 | 0.449827975 | -1.064428664 | 0.022595 | 0.35495 |
| AW551984 | expressed sequence AW551984 | 0.754996362 | 1.565931396 | -1.068099367 | 7.76E-10 | 1.95E-06 |

| | | | | | | |
|---|---|---|---|---|---|---|
| Lgr5 | leucine rich repeat containing G protein cou | 0.080531457 | 0.16731207 | -1.070261625 | 0.011094 | 0.26378 |
| Tgtp1 | T cell specific GTPase 1 | 0.156285731 | 0.32678738 | -1.070679683 | 0.048157 | 0.474657 |
| Pifo | primary cilia formation | 0.751167285 | 1.566551521 | -1.070740462 | 0.026037 | 0.373081 |
| Gm37691 | predicted gene, 37691 | 0.1723715 | 0.36365271 | -1.078032585 | 0.047366 | 0.47273 |
| Tmie | transmembrane inner ear | 0.855495271 | 1.790238124 | -1.079873838 | 2.58E-05 | 0.007829 |
| Spag8 | sperm associated antigen 8 | 0.626773568 | 1.317093137 | -1.081095616 | 0.012479 | 0.277167 |
| Tmc4 | transmembrane channel-like gene family 4 | 0.217740503 | 0.459113013 | -1.08131783 | 0.004015 | 0.16123 |
| Dnah7a | dynein, axonemal, heavy chain 7A | 0.076141203 | 0.159248596 | -1.081762917 | 0.002064 | 0.115461 |
| Oca2 | oculocutaneous albinism II | 0.245758866 | 0.513538384 | -1.08343605 | 0.028055 | 0.382242 |
| Hs3st3b1 | heparan sulfate (glucosamine) 3-O-sulfotrans | 0.22415 | 0.470573963 | -1.083776577 | 0.04812 | 0.474657 |
| Tmem255a | transmembrane protein 255A | 2.383722997 | 5.020017295 | -1.089572679 | 3.30E-13 | 1.46E-09 |
| Gm17455 | predicted gene, 17455 | 0.523781542 | 1.118165275 | -1.097874426 | 0.01945 | 0.330607 |
| Dnah12 | dynein, axonemal, heavy chain 12 | 0.071854842 | 0.152839657 | -1.101018931 | 0.017813 | 0.319413 |
| Dcn | decorin | 3.865499521 | 8.229982838 | -1.104133476 | 2.10E-07 | 0.000185 |
| Mycbpap | MYCBP associated protein | 0.346255096 | 0.741127421 | -1.10419626 | 0.005588 | 0.189435 |
| Gm3739 | predicted gene 3739 | 0.264683276 | 0.566948911 | -1.104770301 | 0.016768 | 0.315592 |
| Iqca | IQ motif containing with AAA domain | 0.236811472 | 0.505019476 | -1.105762572 | 0.002336 | 0.120423 |
| Serpinb1b | serine (or cysteine) peptidase inhibitor, clade | 0.730205168 | 1.552465 | -1.107001967 | 0.016892 | 0.315592 |
| Tekt1 | tektin 1 | 1.48386624 | 3.158451152 | -1.108569098 | 0.000548 | 0.050334 |
| Gm29675 | predicted gene, 29675 | 0.136522526 | 0.293451456 | -1.115083976 | 0.0033 | 0.142987 |
| Pon3 | paraoxonase 3 | 0.571007363 | 1.225906883 | -1.121518835 | 0.00441 | 0.16609 |
| Cd59b | CD59b antigen | 0.319234932 | 0.690140429 | -1.121689072 | 0.041327 | 0.449122 |
| Mlf1 | myeloid leukemia factor 1 | 0.843374668 | 1.818368706 | -1.122217557 | 0.003312 | 0.142987 |
| Wdr63 | WD repeat domain 63 | 0.265570511 | 0.572231173 | -1.124129802 | 0.007875 | 0.22354 |
| Lrrc23 | leucine rich repeat containing 23 | 1.392308562 | 3.01368819 | -1.125296047 | 0.000805 | 0.064539 |
| Ubxn10 | UBX domain protein 10 | 0.409885037 | 0.887845546 | -1.126982598 | 0.002083 | 0.115461 |
| Adh1 | alcohol dehydrogenase 1 (class I) | 0.287897888 | 0.624691631 | -1.129660819 | 0.003118 | 0.139645 |
| Selenov | selenoprotein V | 0.21450652 | 0.468164931 | -1.134064144 | 0.04317 | 0.451337 |
| Ak7 | adenylate kinase 7 | 0.780494906 | 1.702339824 | -1.134609663 | 0.000118 | 0.019405 |
| 1700016K1 | RIKEN cDNA 1700016K19 gene | 1.103156539 | 2.40708022 | -1.13491227 | 0.000986 | 0.072698 |
| Rbm47 | RNA binding motif protein 47 | 0.173195218 | 0.374365165 | -1.136524618 | 0.024385 | 0.363714 |
| Gm13629 | predicted gene 13629 | 0.783185944 | 1.70367556 | -1.139944147 | 0.024019 | 0.363714 |
| Otof | otoferlin | 0.330993267 | 0.720023253 | -1.141586076 | 4.14E-05 | 0.010727 |
| Scube3 | signal peptide, CUB domain, EGF-like 3 | 0.252698582 | 0.55203007 | -1.143056074 | 0.002087 | 0.115461 |
| Gm29595 | predicted gene 29595 | 0.724929298 | 1.593870161 | -1.144560519 | 0.028021 | 0.382242 |
| Cfap77 | cilia and flagella associated protein 77 | 0.153839886 | 0.336514216 | -1.146025624 | 0.016393 | 0.31404 |
| Gpr88 | G-protein coupled receptor 88 | 1.634755651 | 3.581813412 | -1.151508146 | 0.032675 | 0.409368 |
| Bbox1 | butyrobetaine (gamma), 2-oxoglutarate diox | 0.409468849 | 0.904088911 | -1.152914441 | 0.000539 | 0.050016 |
| 2010001K2 | RIKEN cDNA 2010001K21 gene | 0.192116815 | 0.42671916 | -1.160454949 | 0.006588 | 0.205888 |

| | | | | | | |
|---|---|---|---|---|---|---|
| Cfap61 | cilia and flagella associated protein 61 | 0.123807227 | 0.274661728 | -1.160993511 | 0.017083 | 0.315784 |
| Mapk15 | mitogen-activated protein kinase 15 | 0.447974746 | 0.994766067 | -1.161784297 | 0.001685 | 0.10205 |
| Ccdc33 | coiled-coil domain containing 33 | 0.0786654 | 0.174805273 | -1.169919855 | 0.019796 | 0.333567 |
| Tmem212 | transmembrane protein 212 | 2.595795719 | 5.804247906 | -1.170855122 | 0.005707 | 0.191845 |
| Vat1l | vesicle amine transport protein 1 like | 4.223597745 | 9.464578348 | -1.182114938 | 0.001755 | 0.103969 |
| Capn6 | calpain 6 | 0.137540809 | 0.309423172 | -1.18551914 | 0.002915 | 0.13602 |
| Zfp185 | zinc finger protein 185 | 0.41852224 | 0.944690269 | -1.186517421 | 0.006429 | 0.203601 |
| Myo1h | myosin 1H | 0.055351784 | 0.125044054 | -1.188749648 | 0.041588 | 0.449122 |
| Mybpc2 | myosin binding protein C, fast-type | 0.112008209 | 0.253693275 | -1.190566758 | 0.013704 | 0.288529 |
| Mfsd7a | major facilitator superfamily domain contair | 0.184668988 | 0.416644484 | -1.192101619 | 0.011772 | 0.270195 |
| Ptpn20 | protein tyrosine phosphatase, non-receptor t | 0.093370541 | 0.212343769 | -1.193237418 | 0.042292 | 0.449801 |
| Maats1 | MYCBP-associated, testis expressed 1 | 0.235771709 | 0.534815444 | -1.198544388 | 8.22E-05 | 0.01642 |
| Gm47936 | predicted gene, 47936 | 0.46350364 | 1.062593032 | -1.208499092 | 0.041938 | 0.449674 |
| Spag17 | sperm associated antigen 17 | 0.044317874 | 0.101357836 | -1.211128639 | 0.023875 | 0.363225 |
| Krt15 | keratin 15 | 0.528502471 | 1.223301442 | -1.217280531 | 0.003402 | 0.145193 |
| Baiap2l1 | BAI1-associated protein 2-like 1 | 0.320211065 | 0.734362214 | -1.217356806 | 0.006271 | 0.201336 |
| Exoc3l4 | exocyst complex component 3-like 4 | 0.065726058 | 0.152677716 | -1.224204236 | 0.033921 | 0.411285 |
| Apoc1 | apolipoprotein C-I | 0.922614878 | 2.135039274 | -1.224723216 | 0.020763 | 0.340459 |
| Nkx2-1 | NK2 homeobox 1 | 0.147955601 | 0.342013213 | -1.228016843 | 0.020874 | 0.341521 |
| Tmem202 | transmembrane protein 202 | 0.32640836 | 0.756590002 | -1.229771132 | 0.003006 | 0.137611 |
| Ect2l | epithelial cell transforming sequence 2 oncog | 0.069730625 | 0.163699724 | -1.232591526 | 0.042567 | 0.449801 |
| Cfap52 | cilia and flagella associated protein 52 | 0.677961562 | 1.585568951 | -1.236236031 | 0.000308 | 0.036069 |
| Six4 | sine oculis-related homeobox 4 | 0.037183234 | 0.086762228 | -1.238710408 | 0.014969 | 0.300531 |
| Myb | myeloblastosis oncogene | 0.090595123 | 0.212986988 | -1.243800843 | 0.000764 | 0.062908 |
| Gm15478 | predicted gene 15478 | 0.158810235 | 0.372937982 | -1.24880343 | 0.021461 | 0.345785 |
| Gm42460 | predicted gene 42460 | 0.111571173 | 0.262867116 | -1.253412087 | 0.038299 | 0.434618 |
| 2410004P( | RIKEN cDNA 2410004P03 gene | 0.802696686 | 1.901926456 | -1.254563485 | 0.00034 | 0.038442 |
| Arhgef16 | Rho guanine nucleotide exchange factor (GEF | 0.079662421 | 0.188336914 | -1.262458496 | 0.037498 | 0.430888 |
| Fsd2 | fibronectin type III and SPRY domain contain | 0.094158407 | 0.22518829 | -1.271171353 | 0.026365 | 0.373882 |
| A530072M | RIKEN cDNA gene A530072M11 | 0.191463347 | 0.463197234 | -1.277346263 | 0.015237 | 0.303835 |
| Ccdc170 | coiled-coil domain containing 170 | 0.164287654 | 0.395843337 | -1.27916663 | 0.003413 | 0.145311 |
| Cfap57 | cilia and flagella associated protein 57 | 0.155242702 | 0.373698633 | -1.280242368 | 0.002589 | 0.126769 |
| Pld5 | phospholipase D family, member 5 | 0.234878548 | 0.564556399 | -1.286943179 | 0.001737 | 0.103969 |
| Slc35d3 | solute carrier family 35, member D3 | 0.203559435 | 0.495012512 | -1.293389154 | 0.00406 | 0.161238 |
| Gsta3 | glutathione S-transferase, alpha 3 | 0.101556758 | 0.248921971 | -1.294236203 | 0.016853 | 0.315592 |
| B130024G: | RIKEN cDNA B130024G19 gene | 0.036555349 | 0.08813721 | -1.294417978 | 0.01967 | 0.332748 |
| Dnah6 | dynein, axonemal, heavy chain 6 | 0.160022717 | 0.389537021 | -1.295223651 | 5.63E-05 | 0.012729 |
| Cfap206 | cilia and flagella associated protein 206 | 0.429177074 | 1.045765395 | -1.295940731 | 0.002251 | 0.119646 |
| Armc3 | armadillo repeat containing 3 | 0.314415506 | 0.76729301 | -1.297535896 | 0.000433 | 0.043554 |

| | | | | | | |
|---|---|---|---|---|---|---|
| Gm46376 | predicted gene, 46376 | 0.319837847 | 0.783266095 | -1.298866252 | 0.048883 | 0.475925 |
| Gm47202 | predicted gene, 47202 | 0.139982169 | 0.341330408 | -1.30302751 | 0.045084 | 0.461229 |
| Rsph1 | radial spoke head 1 homolog (Chlamydomon | 3.089129872 | 7.57133639 | -1.306654091 | 4.02E-05 | 0.010568 |
| Ccdc180 | coiled-coil domain containing 180 | 0.129166596 | 0.317088495 | -1.30735469 | 0.003687 | 0.152551 |
| Dnali1 | dynein, axonemal, light intermediate polype | 0.66304925 | 1.636436432 | -1.30995769 | 0.001596 | 0.097347 |
| Gm867 | predicted gene 867 | 0.40891547 | 1.014796914 | -1.318300737 | 0.007693 | 0.221946 |
| Samd11 | sterile alpha motif domain containing 11 | 0.112755112 | 0.278201781 | -1.318764895 | 0.017631 | 0.318417 |
| Rsph4a | radial spoke head 4 homolog A (Chlamydomo | 0.708822666 | 1.760870241 | -1.323875288 | 9.45E-08 | 0.00011 |
| Resp18 | regulated endocrine-specific protein 18 | 15.66719388 | 38.87053824 | -1.326192579 | 1.61E-13 | 9.46E-10 |
| Wnt9b | wingless-type MMTV integration site family, r | 0.1892266 | 0.470519011 | -1.331660926 | 0.03863 | 0.435662 |
| Adrb3 | adrenergic receptor, beta 3 | 0.084819713 | 0.211132371 | -1.332632072 | 0.033073 | 0.411041 |
| Lrrc43 | leucine rich repeat containing 43 | 0.14885895 | 0.376125317 | -1.336256477 | 0.046607 | 0.469916 |
| Ccdc153 | coiled-coil domain containing 153 | 2.181546371 | 5.468778211 | -1.33712874 | 0.003803 | 0.155187 |
| Cbln1 | cerebellin 1 precursor protein | 1.545848759 | 3.860637569 | -1.341183016 | 1.51E-05 | 0.005312 |
| Glp1r | glucagon-like peptide 1 receptor | 0.210483322 | 0.528934315 | -1.342685714 | 0.031084 | 0.400526 |
| Lbp | lipopolysaccharide binding protein | 1.443248121 | 3.629094672 | -1.348720454 | 0.0331 | 0.411041 |
| Drc7 | dynein regulatory complex subunit 7 | 1.070536926 | 2.696818227 | -1.350312296 | 0.048875 | 0.475925 |
| Fsip1 | fibrous sheath-interacting protein 1 | 0.111694376 | 0.285167836 | -1.351111372 | 0.033645 | 0.411285 |
| Lin28b | lin-28 homolog B (C. elegans) | 0.075203399 | 0.191019254 | -1.352669399 | 0.002706 | 0.129245 |
| Cfap221 | cilia and flagella associated protein 221 | 0.082470224 | 0.209076332 | -1.361616464 | 0.022052 | 0.351135 |
| Sulf1 | sulfatase 1 | 0.931210122 | 2.371214851 | -1.367170017 | 0.049823 | 0.479645 |
| 1700007K1 | RIKEN cDNA 1700007K13 gene | 1.530316924 | 3.921730287 | -1.372786987 | 1.99E-05 | 0.006406 |
| Lrrc34 | leucine rich repeat containing 34 | 0.1720791 | 0.443386401 | -1.374063403 | 0.034177 | 0.412986 |
| Serpina3g | serine (or cysteine) peptidase inhibitor, clade | 0.185792815 | 0.475102497 | -1.375066538 | 0.003488 | 0.146404 |
| Pla2g5 | phospholipase A2, group V | 0.42313416 | 1.089513606 | -1.382313546 | 0.005264 | 0.182498 |
| Gm26532 | predicted gene, 26532 | 0.325853882 | 0.839754362 | -1.384199482 | 0.029906 | 0.3931 |
| Ttc25 | tetratricopeptide repeat domain 25 | 0.108456778 | 0.284083391 | -1.400737425 | 0.006177 | 0.199768 |
| 6820408C1 | RIKEN cDNA 6820408C15 gene | 0.677397359 | 1.794808657 | -1.416743137 | 5.47E-07 | 0.000402 |
| Kl | klotho | 2.013069335 | 5.307932926 | -1.417321422 | 0.047153 | 0.47273 |
| Arhgap6 | Rho GTPase activating protein 6 | 0.124687213 | 0.330039046 | -1.417390789 | 1.24E-07 | 0.000125 |
| Cfap73 | cilia and flagella associated protein 73 | 0.226947234 | 0.604379493 | -1.421212952 | 0.033493 | 0.411285 |
| Ripk3 | receptor-interacting serine-threonine kinase | 0.078094342 | 0.208075305 | -1.424138428 | 0.047499 | 0.472936 |
| Epn3 | epsin 3 | 0.822274586 | 2.183312484 | -1.426763348 | 0.018135 | 0.322491 |
| Uox | urate oxidase | 0.079349306 | 0.213156992 | -1.428212972 | 0.006662 | 0.206058 |
| Fam216b | family with sequence similarity 216, membe | 0.625996636 | 1.67322034 | -1.42958621 | 9.79E-05 | 0.017426 |
| Ttc29 | tetratricopeptide repeat domain 29 | 0.104784277 | 0.281669382 | -1.430911809 | 0.004916 | 0.176109 |
| 4933406B1 | RIKEN cDNA 4933406B17 gene | 0.075473111 | 0.204500088 | -1.445311905 | 0.010594 | 0.258274 |
| Dnajb13 | DnaJ heat shock protein family (Hsp40) mem | 0.178719595 | 0.48544867 | -1.45388477 | 0.014669 | 0.297056 |
| Tekt2 | tektin 2 | 0.256457239 | 0.703112095 | -1.459783925 | 0.002217 | 0.119646 |

| | | | | | | |
|---|---|---|---|---|---|---|
| Tnni3k | TNNI3 interacting kinase | 0.050153696 | 0.137168991 | -1.466481504 | 0.042067 | 0.449674 |
| Cbln3 | cerebellin 3 precursor protein | 0.15394505 | 0.419341514 | -1.466757748 | 0.025042 | 0.36754 |
| Gm29683 | predicted gene, 29683 | 0.079461788 | 0.219041661 | -1.469148364 | 0.013961 | 0.289976 |
| Fam196b | family with sequence similarity 196, member | 0.158706551 | 0.43404011 | -1.469361196 | 0.002259 | 0.119646 |
| 4930520O( | RIKEN cDNA 4930520O04 gene | 0.091561447 | 0.25131323 | -1.472844304 | 0.006322 | 0.202147 |
| Smim5 | small integral membrane protein 5 | 0.432413137 | 1.202722299 | -1.487898522 | 0.000289 | 0.034695 |
| Sostdc1 | sclerostin domain containing 1 | 2.950805584 | 8.172230293 | -1.489516298 | 0.040963 | 0.447817 |
| Spata18 | spermatogenesis associated 18 | 0.25294739 | 0.706901014 | -1.490075111 | 0.004289 | 0.165686 |
| AU023762 | expressed sequence AU023762 | 0.132385929 | 0.369842795 | -1.491215799 | 0.001572 | 0.097312 |
| 1700001C( | RIKEN cDNA 1700001C02 gene | 0.355444185 | 1.002705912 | -1.50043611 | 0.013858 | 0.289777 |
| Jhy | junctional cadherin complex regulator | 0.103212543 | 0.287394012 | -1.500483359 | 0.01505 | 0.30147 |
| Sntn | sentan, cilia apical structure protein | 0.22696556 | 0.642249117 | -1.503703176 | 0.016861 | 0.315592 |
| Cfap58 | cilia and flagella associated protein 58 | 0.11996254 | 0.338272161 | -1.504653759 | 0.002728 | 0.12997 |
| Vegfd | vascular endothelial growth factor D | 0.38597788 | 1.086362401 | -1.504816964 | 3.02E-05 | 0.008574 |
| H2-Q2 | histocompatibility 2, Q region locus 2 | 0.21958677 | 0.615420641 | -1.505486499 | 0.012529 | 0.277167 |
| Myl2 | myosin, light polypeptide 2, regulatory, card | 0.113185079 | 0.320538067 | -1.505647938 | 0.024685 | 0.365666 |
| Gm16201 | predicted gene 16201 | 0.122958306 | 0.347636792 | -1.506784522 | 0.012627 | 0.278917 |
| F2rl3 | coagulation factor II (thrombin) receptor-like | 0.103749469 | 0.294080826 | -1.506785466 | 0.029264 | 0.389312 |
| Ebf2 | early B cell factor 2 | 0.061071942 | 0.171028308 | -1.510068403 | 0.033205 | 0.411061 |
| Daw1 | dynein assembly factor with WDR repeat don | 0.172431268 | 0.490769861 | -1.512840694 | 0.006645 | 0.206058 |
| Gm44907 | predicted gene 44907 | 0.108170802 | 0.310057763 | -1.515564558 | 0.044935 | 0.461002 |
| Grhl3 | grainyhead like transcription factor 3 | 0.075674656 | 0.213610188 | -1.516643849 | 0.016356 | 0.313715 |
| Mrap2 | melanocortin 2 receptor accessory protein 2 | 0.687523026 | 1.953613669 | -1.520785334 | 1.65E-06 | 0.000917 |
| Gm41414 | predicted gene, 41414 | 0.340210473 | 0.9653966 | -1.523654757 | 0.011756 | 0.270195 |
| Ttc21a | tetratricopeptide repeat domain 21A | 0.259098089 | 0.74338622 | -1.532577386 | 7.44E-07 | 0.000517 |
| Gm48865 | predicted gene, 48865 | 0.064222545 | 0.182320138 | -1.533620024 | 0.036031 | 0.424092 |
| Mdh1b | malate dehydrogenase 1B, NAD (soluble) | 0.267666333 | 0.773345086 | -1.542956927 | 0.002468 | 0.123592 |
| Krt8 | keratin 8 | 0.390123655 | 1.123961927 | -1.546856718 | 0.008088 | 0.225748 |
| Gm47414 | predicted gene, 47414 | 0.069547323 | 0.201874715 | -1.547419512 | 0.032276 | 0.406666 |
| Gabrq | gamma-aminobutyric acid (GABA) A receptor | 0.089855215 | 0.260262535 | -1.549717924 | 0.000164 | 0.024184 |
| Lmx1a | LIM homeobox transcription factor 1 alpha | 0.124019994 | 0.358170504 | -1.552464759 | 0.005702 | 0.191845 |
| Vgll3 | vestigial like family member 3 | 0.305899932 | 0.888660376 | -1.552565449 | 0.0001 | 0.017496 |
| Ecel1 | endothelin converting enzyme-like 1 | 0.50526744 | 1.466970163 | -1.555814379 | 1.27E-07 | 0.000125 |
| Gm26610 | predicted gene, 26610 | 0.173523389 | 0.509790719 | -1.563291337 | 0.024574 | 0.365666 |
| Lhx1os | LIM homeobox 1, opposite strand | 0.506006277 | 1.465241366 | -1.566146358 | 0.032039 | 0.405418 |
| Six3os1 | SIX homeobox 3, opposite strand 1 | 0.046613449 | 0.136140283 | -1.56697773 | 0.00966 | 0.244306 |
| Slc39a4 | solute carrier family 39 (zinc transporter), me | 0.210356698 | 0.623742143 | -1.582101442 | 0.002954 | 0.136672 |
| C230072F1 | RIKEN cDNA C230072F16 gene | 0.238334608 | 0.710652619 | -1.587713647 | 0.002451 | 0.123592 |
| Lhx5 | LIM homeobox protein 5 | 0.192028203 | 0.567827973 | -1.589374516 | 0.010017 | 0.250767 |

| | | | | | | |
|---|---|---|---|---|---|---|
| Acp7 | acid phosphatase 7, tartrate resistant | 0.11479457 | 0.344320057 | -1.596518013 | 0.011581 | 0.268242 |
| Htr2c | 5-hydroxytryptamine (serotonin) receptor 2( | 2.000762002 | 6.002158845 | -1.602245239 | 0.000566 | 0.05165 |
| Scn5a | sodium channel, voltage-gated, type V, alpha | 0.101868488 | 0.306743663 | -1.604779695 | 3.45E-05 | 0.009634 |
| Tssk1 | testis-specific serine kinase 1 | 0.035887117 | 0.109146299 | -1.606008658 | 0.044111 | 0.456222 |
| Gm26808 | predicted gene, 26808 | 0.363476624 | 1.102217313 | -1.614188117 | 0.047413 | 0.47273 |
| Krt18 | keratin 18 | 0.617388533 | 1.898619291 | -1.633431708 | 0.002839 | 0.133079 |
| Spag16 | sperm associated antigen 16 | 0.163199866 | 0.501502158 | -1.636758096 | 0.000217 | 0.028228 |
| Nek5 | NIMA (never in mitosis gene a)-related expres | 0.205959989 | 0.634165979 | -1.639715087 | 0.001202 | 0.08149 |
| Ngb | neuroglobin | 0.997365535 | 3.081109535 | -1.642543765 | 6.03E-11 | 1.77E-07 |
| Got1l1 | glutamic-oxaloacetic transaminase 1-like 1 | 0.21688258 | 0.678114398 | -1.644567184 | 0.016565 | 0.31404 |
| Gm7854 | predicted gene 7854 | 0.092768935 | 0.290417811 | -1.647914624 | 0.041727 | 0.449122 |
| Large2 | LARGE xylosyl- and glucuronyltransferase 2 | 0.04819832 | 0.150011325 | -1.650522552 | 0.017189 | 0.316008 |
| Amdhd1 | amidohydrolase domain containing 1 | 0.067419787 | 0.211258022 | -1.657155819 | 0.024269 | 0.363714 |
| D930020B: | RIKEN cDNA D930020B18 gene | 0.045064413 | 0.14204028 | -1.661611863 | 0.017586 | 0.318417 |
| Sfrp5 | secreted frizzled-related sequence protein 5 | 0.50876957 | 1.596231802 | -1.666323428 | 0.034706 | 0.414579 |
| Rab38 | RAB38, member RAS oncogene family | 0.236443843 | 0.743271603 | -1.666481091 | 0.006353 | 0.202147 |
| Gm29154 | predicted gene 29154 | 0.020719335 | 0.064461787 | -1.66671233 | 0.018812 | 0.327337 |
| 1700012B( | RIKEN cDNA 1700012B09 gene | 0.47063971 | 1.494778317 | -1.676400219 | 0.009574 | 0.244306 |
| 4930526F1 | RIKEN cDNA 4930526F13 gene | 0.094691491 | 0.30216817 | -1.678085711 | 0.027914 | 0.382242 |
| Crygn | crystallin, gamma N | 0.586472689 | 1.878493669 | -1.681674608 | 0.001087 | 0.076943 |
| Gm16418 | predicted pseudogene 16418 | 0.714845409 | 2.24100745 | -1.685202358 | 0.041603 | 0.449122 |
| Oprk1 | opioid receptor, kappa 1 | 0.047622347 | 0.151354036 | -1.689920253 | 0.00013 | 0.020762 |
| C1qtnf3 | C1q and tumor necrosis factor related protei | 0.135675477 | 0.432286645 | -1.691319549 | 0.005968 | 0.19607 |
| Gm44597 | predicted gene 44597 | 0.036567428 | 0.117800456 | -1.692966622 | 0.026427 | 0.374467 |
| Prr29 | proline rich 29 | 0.078868491 | 0.25097235 | -1.694213205 | 0.042379 | 0.449801 |
| Dynlrb2 | dynein light chain roadblock-type 2 | 1.736427279 | 5.576875947 | -1.695375155 | 1.18E-05 | 0.00452 |
| Gm39244 | predicted gene, 39244 | 0.137939532 | 0.443464492 | -1.698930605 | 0.017216 | 0.316008 |
| Eppk1 | epiplakin 1 | 0.010686762 | 0.034926686 | -1.708334095 | 0.048728 | 0.475516 |
| Pax5 | paired box 5 | 0.022194541 | 0.071851309 | -1.710387842 | 0.010588 | 0.258274 |
| Klhl1 | kelch-like 1 | 0.09499403 | 0.308371514 | -1.717232146 | 0.000326 | 0.037361 |
| Cfap70 | cilia and flagella associated protein 70 | 0.167553595 | 0.546868509 | -1.720711216 | 8.78E-06 | 0.003684 |
| Col8a2 | collagen, type VIII, alpha 2 | 0.905889815 | 2.961319471 | -1.726329914 | 0.020044 | 0.334904 |
| Gm18180 | predicted gene, 18180 | 0.16976931 | 0.558792738 | -1.72713373 | 0.013533 | 0.287755 |
| 1700024G: | RIKEN cDNA 1700024G13 gene | 0.31464392 | 1.052066785 | -1.746884008 | 0.009447 | 0.243219 |
| Samd3 | sterile alpha motif domain containing 3 | 0.082335635 | 0.274090131 | -1.751576347 | 0.015306 | 0.304034 |
| Gm34045 | predicted gene, 34045 | 0.152366015 | 0.509038969 | -1.752008777 | 0.024652 | 0.365666 |
| Cfap161 | cilia and flagella associated protein 161 | 0.245792187 | 0.831759528 | -1.765141245 | 1.66E-06 | 0.000917 |
| Acox2 | acyl-Coenzyme A oxidase 2, branched chain | 0.076510926 | 0.256844776 | -1.765383415 | 0.008584 | 0.23271 |
| Gabra6 | gamma-aminobutyric acid (GABA) A receptor | 0.111251291 | 0.376590041 | -1.782656166 | 0.047667 | 0.472936 |

| | | | | | | |
|---|---|---|---|---|---|---|
| Rec8 | REC8 meiotic recombination protein | 0.482428602 | 1.642907852 | -1.786901582 | 2.36E-08 | 3.78E-05 |
| Rd3 | retinal degeneration 3 | 0.130021222 | 0.446943359 | -1.79080463 | 0.002621 | 0.127172 |
| Slco1a5 | solute carrier organic anion transporter fami | 0.095971713 | 0.33009586 | -1.797143729 | 0.009621 | 0.244306 |
| Krt19 | keratin 19 | 0.070961511 | 0.248838388 | -1.801255669 | 0.047542 | 0.472936 |
| Gm31518 | predicted gene, 31518 | 0.037122426 | 0.128486419 | -1.801541984 | 0.032185 | 0.406386 |
| Acss3 | acyl-CoA synthetase short-chain family meml | 0.049675648 | 0.171830554 | -1.808174951 | 0.006867 | 0.209416 |
| 1700080G1 | RIKEN cDNA 1700080G11 gene | 0.277966821 | 0.971233169 | -1.826485816 | 0.024884 | 0.367061 |
| Crb3 | crumbs family member 3 | 0.249960568 | 0.873085806 | -1.826592203 | 0.003129 | 0.139645 |
| Tacr3 | tachykinin receptor 3 | 0.252820547 | 0.8840834 | -1.829026049 | 2.52E-07 | 0.000211 |
| Calml4 | calmodulin-like 4 | 0.584846182 | 2.061253347 | -1.832378885 | 0.014185 | 0.290753 |
| Gm32817 | predicted gene, 32817 | 0.057700803 | 0.204040472 | -1.8326638 | 0.009164 | 0.238131 |
| Magel2 | melanoma antigen, family L, 2 | 0.187122961 | 0.665411567 | -1.845741952 | 2.93E-06 | 0.001396 |
| A230072E1 | RIKEN cDNA A230072E10 gene | 0.026751101 | 0.094450166 | -1.855035365 | 0.00978 | 0.24593 |
| 9330158H( | RIKEN cDNA 9330158H04 gene | 0.014367413 | 0.052571899 | -1.862690404 | 0.019381 | 0.330205 |
| Gm26911 | predicted gene, 26911 | 0.037815355 | 0.135575519 | -1.871865491 | 0.046315 | 1 |
| Gm38414 | predicted gene, 38414 | 0.055187046 | 0.203166016 | -1.878342386 | 0.017219 | 0.316008 |
| Wdr72 | WD repeat domain 72 | 0.037832789 | 0.137299822 | -1.879298679 | 0.02119 | 0.34331 |
| Slc4a1 | solute carrier family 4 (anion exchanger), mer | 0.036115911 | 0.135716661 | -1.902861098 | 0.018757 | 0.327186 |
| B630019A1 | RIKEN cDNA B630019A10 gene | 0.015746884 | 0.059458832 | -1.913898008 | 0.01669 | 0.31499 |
| 4833427G( | RIKEN cDNA 4833427G06 gene | 0.228663789 | 0.854934526 | -1.915958997 | 0.012214 | 0.274963 |
| Mir212 | microRNA 212 | 1.287570969 | 4.855898863 | -1.920092026 | 0.040482 | 0.444433 |
| Gm20684 | predicted gene 20684 | 0.35010943 | 1.319822747 | -1.920761058 | 0.023224 | 0.360229 |
| Prlr | prolactin receptor | 0.247771703 | 0.92626195 | -1.922374254 | 0.044309 | 0.456743 |
| Gm48898 | predicted gene, 48898 | 0.03880406 | 0.146136397 | -1.9234455 | 0.02796 | 0.382242 |
| Gm26684 | predicted gene, 26684 | 0.033190121 | 0.125180143 | -1.924399261 | 0.003474 | 0.146404 |
| Irx5 | Iroquois homeobox 5 | 0.080935845 | 0.305343609 | -1.928533905 | 0.001758 | 0.103969 |
| Mab21l1 | mab-21-like 1 (C. elegans) | 0.178667328 | 0.68132777 | -1.942503252 | 0.004743 | 0.172749 |
| Zfp474 | zinc finger protein 474 | 0.136306281 | 0.522313387 | -1.944866154 | 0.000736 | 0.061471 |
| Cpn1 | carboxypeptidase N, polypeptide 1 | 0.05956233 | 0.230174536 | -1.952501374 | 0.008893 | 0.236504 |
| Gm29771 | predicted gene, 29771 | 0.136720841 | 0.529585034 | -1.96466705 | 0.001275 | 0.085801 |
| Crocc2 | ciliary rootlet coiled-coil, rootletin family m | 0.097218096 | 0.378554186 | -1.966458739 | 7.63E-07 | 0.000517 |
| Gabre | gamma-aminobutyric acid (GABA) A receptor | 0.052733086 | 0.207506117 | -1.981495167 | 0.004562 | 0.169288 |
| Irx3 | Iroquois related homeobox 3 | 0.144317977 | 0.568785427 | -1.987413712 | 0.00133 | 0.087213 |
| Smkr-ps | smal lysine rich protein 1, pseudogene | 0.164992438 | 0.653772639 | -1.996771166 | 0.013005 | 0.282313 |
| Clic6 | chloride intracellular channel 6 | 1.968033984 | 7.766028528 | -1.999413659 | 0.01876 | 0.327186 |
| Dydc2 | DPY30 domain containing 2 | 0.197881632 | 0.790589076 | -2.007430524 | 0.031276 | 0.40182 |
| 2900040C( | RIKEN cDNA 2900040C04 gene | 2.47370322 | 9.868694123 | -2.014944811 | 0.048649 | 0.475516 |
| Slc6a5 | solute carrier family 6 (neurotransmitter trar | 0.014947969 | 0.058932455 | -2.016634467 | 0.048457 | 0.475326 |
| Otx2 | orthodenticle homeobox 2 | 0.541761667 | 2.19810216 | -2.039094308 | 0.002385 | 0.12122 |

| | | | | | | |
|---|---|---|---|---|---|---|
| Angptl7 | angiopoietin-like 7 | 0.054368409 | 0.224440602 | -2.040688505 | 0.031807 | 0.404338 |
| Lrat | lecithin-retinol acyltransferase (phosphatidy | 0.016412795 | 0.069604658 | -2.068002248 | 0.029527 | 1 |
| Baiap3 | BAI1-associated protein 3 | 0.846120779 | 3.54700288 | -2.081500441 | 1.20E-06 | 0.000755 |
| Gm42427 | predicted gene 42427 | 0.014218233 | 0.060515897 | -2.102938622 | 0.027633 | 1 |
| Gm15956 | predicted gene 15956 | 0.146032617 | 0.634029824 | -2.110142111 | 0.040048 | 1 |
| Cyp26a1 | cytochrome P450, family 26, subfamily a, po | 0.093122373 | 0.400394238 | -2.112305397 | 0.00277 | 0.131592 |
| Corin | corin | 0.027499891 | 0.118485109 | -2.116879946 | 0.003745 | 0.154231 |
| Ttr | transthyretin | 417.0855196 | 1792.780956 | -2.121513468 | 0.048945 | 0.476133 |
| Mogat1 | monoacylglycerol O-acyltransferase 1 | 0.038239834 | 0.166571827 | -2.136056459 | 0.043157 | 1 |
| Barhl2 | BarH like homeobox 2 | 0.083625206 | 0.366190065 | -2.142070888 | 0.006663 | 0.206058 |
| Bdkrb1 | bradykinin receptor, beta 1 | 0.064114978 | 0.281062466 | -2.163583866 | 0.023922 | 0.363515 |
| Gm26685 | predicted gene, 26685 | 0.029475783 | 0.132926832 | -2.172112195 | 0.040114 | 1 |
| 4930502E1 | RIKEN cDNA 4930502E18 gene | 0.052764356 | 0.237623168 | -2.176896069 | 0.030629 | 1 |
| Lhb | luteinizing hormone beta | 0.285258151 | 1.304557227 | -2.2108058 | 0.00469 | 0.171875 |
| Gm32224 | predicted gene, 32224 | 0.091547258 | 0.41701786 | -2.214026386 | 0.018967 | 0.327826 |
| Gm48536 | predicted gene, 48536 | 0.021952245 | 0.10278514 | -2.22150877 | 0.036422 | 1 |
| Tfap2a | transcription factor AP-2, alpha | 0.018551899 | 0.085527385 | -2.232706096 | 0.032262 | 0.406666 |
| Aqp1 | aquaporin 1 | 1.246393035 | 5.851481632 | -2.249063894 | 0.041972 | 0.449674 |
| Gm28729 | predicted gene 28729 | 0.128836322 | 0.608643905 | -2.25542541 | 0.000235 | 0.029635 |
| Olfr1033 | olfactory receptor 1033 | 0.013761166 | 0.066199461 | -2.265679034 | 0.033339 | 1 |
| Cxcl5 | chemokine (C-X-C motif) ligand 5 | 0.136465442 | 0.656029647 | -2.285796766 | 0.003611 | 0.150828 |
| Gm16287 | predicted gene 16287 | 0.026085496 | 0.125849981 | -2.293175274 | 0.02852 | 1 |
| Dlk1 | delta like non-canonical Notch ligand 1 | 0.343071156 | 1.691713122 | -2.319236599 | 2.00E-18 | 1.77E-14 |
| Scd4 | stearoyl-coenzyme A desaturase 4 | 0.024681044 | 0.119666152 | -2.326205037 | 0.012918 | 0.281812 |
| Gm17180 | predicted gene 17180 | 0.168167412 | 0.859720089 | -2.345629785 | 0.01443 | 1 |
| Gm17747 | predicted gene, 17747 | 0.07532893 | 0.376580355 | -2.361804629 | 0.048948 | 1 |
| Tns4 | tensin 4 | 0.020049119 | 0.102846747 | -2.364740014 | 0.007025 | 0.212654 |
| Gm45470 | predicted gene 45470 | 0.011937541 | 0.060807664 | -2.373076855 | 0.007279 | 0.217108 |
| Mfrp | membrane frizzled-related protein | 0.480327262 | 2.453608408 | -2.373318449 | 0.038373 | 0.434618 |
| Cox8b | cytochrome c oxidase subunit 8B | 0.629782893 | 3.230553754 | -2.385724477 | 0.000998 | 0.07329 |
| S100a9 | S100 calcium binding protein A9 (calgranulin | 0.142240815 | 0.773766752 | -2.441272041 | 0.034538 | 0.414161 |
| Dlx4os | distal-less homeobox 4, opposite strand | 0.035803883 | 0.194465642 | -2.457524784 | 0.041807 | 1 |
| Prr32 | proline rich 32 | 0.828341536 | 4.547491426 | -2.472938704 | 0.009479 | 0.243219 |
| Gm13807 | predicted gene 13807 | 0.045198942 | 0.259452267 | -2.516036501 | 0.024423 | 1 |
| 4933439K1 | RIKEN cDNA 4933439K11 gene | 0.014888182 | 0.086276478 | -2.520333114 | 0.034749 | 1 |
| Gm11465 | predicted gene 11465 | 0.105633671 | 0.59402467 | -2.533598395 | 0.048641 | 1 |
| Slc4a5 | solute carrier family 4, sodium bicarbonate c | 0.269802657 | 1.546492936 | -2.534094731 | 0.019968 | 0.334904 |
| Isl1 | ISL1 transcription factor, LIM/homeodomain | 0.023477223 | 0.132339245 | -2.538213587 | 0.017867 | 0.319851 |
| Gm10639 | predicted gene 10639 | 0.04088681 | 0.243608917 | -2.561079757 | 0.026075 | 1 |

| | | | | | | |
|---|---|---|---|---|---|---|
| Irs4 | insulin receptor substrate 4 | 0.034180655 | 0.206071102 | -2.624352509 | 5.93E-05 | 0.012806 |
| Insrr | insulin receptor-related receptor | 0.00786795 | 0.048265732 | -2.637355529 | 0.017075 | 1 |
| Vpreb1 | pre-B lymphocyte gene 1 | 0.048904361 | 0.310796301 | -2.669533715 | 0.031942 | 1 |
| Dio3 | deiodinase, iodothyronine type III | 0.385650166 | 2.476037036 | -2.698202795 | 8.98E-09 | 1.98E-05 |
| Cckar | cholecystokinin A receptor | 0.012210067 | 0.078846921 | -2.699442781 | 0.031375 | 1 |
| Gm26783 | predicted gene, 26783 | 0.012323197 | 0.082889398 | -2.740912274 | 0.043939 | 1 |
| Gm44109 | predicted gene, 44109 | 0.02298138 | 0.151781958 | -2.746328781 | 0.021548 | 1 |
| B230312C( | RIKEN cDNA B230312C02 gene | 0.053381158 | 0.352339455 | -2.748624498 | 0.00117 | 0.080244 |
| Sh3rf2 | SH3 domain containing ring finger 2 | 0.014168275 | 0.094399357 | -2.760186514 | 0.008288 | 0.228265 |
| Gm15587 | predicted gene 15587 | 0.012237961 | 0.082874798 | -2.784347861 | 0.018659 | 1 |
| Dppa5a | developmental pluripotency associated 5A | 0.047321873 | 0.33298737 | -2.811021805 | 0.046503 | 1 |
| Gm49484 | predicted gene, 49484 | 0.01814505 | 0.124157629 | -2.837883771 | 0.014356 | 1 |
| Sost | sclerostin | 0.030329576 | 0.214173206 | -2.876585861 | 0.021279 | 0.344035 |
| Hs3st6 | heparan sulfate (glucosamine) 3-O-sulfotrans | 0.035980791 | 0.266821178 | -2.876897291 | 0.038754 | 1 |
| Gm13814 | predicted gene 13814 | 0.010182142 | 0.071851496 | -2.887859339 | 0.032545 | 1 |
| Gm37968 | predicted gene, 37968 | 0.009150965 | 0.069068647 | -2.912678767 | 0.02855 | 1 |
| Hba-a2 | hemoglobin alpha, adult chain 2 | 0.399714693 | 3.02326201 | -2.924541534 | 0.000106 | 0.018291 |
| Gdnf | glial cell line derived neurotrophic factor | 0.008257286 | 0.061536994 | -2.929245735 | 0.040235 | 1 |
| 9430034N: | RIKEN cDNA 9430034N14 gene | 0.007725334 | 0.060554414 | -2.965205265 | 0.039471 | 1 |
| Gm12070 | predicted gene 12070 | 0.030276717 | 0.234637357 | -2.967629474 | 0.031494 | 1 |
| Nppa | natriuretic peptide type A | 0.033863861 | 0.277818396 | -3.015037793 | 0.023999 | 1 |
| Myh13 | myosin, heavy polypeptide 13, skeletal musc | 0.004739845 | 0.038803289 | -3.023716328 | 0.03177 | 1 |
| Gm32468 | predicted gene, 32468 | 0.081669813 | 0.66180976 | -3.023799431 | 0.00372 | 0.153573 |
| Cpa6 | carboxypeptidase A6 | 0.004427693 | 0.035719779 | -3.035170402 | 0.022236 | 1 |
| Hba-a1 | hemoglobin alpha, adult chain 1 | 3.795048405 | 31.94688693 | -3.081621997 | 0.00181 | 0.106678 |
| Hbb-bt | hemoglobin, beta adult t chain | 1.302551606 | 10.98911414 | -3.082305698 | 0.002608 | 0.127172 |
| Rd3l | retinal degeneration 3-like | 0.022400707 | 0.192991053 | -3.108193368 | 0.026036 | 1 |
| Mir541 | microRNA 541 | 0.325469328 | 2.864126046 | -3.131360723 | 0.022951 | 1 |
| Hbb-bs | hemoglobin, beta adult s chain | 7.403889926 | 66.21179305 | -3.167276055 | 0.001063 | 0.076488 |
| Sln | sarcolipin | 0.146066422 | 1.355792554 | -3.222280104 | 1.59E-08 | 3.12E-05 |
| Slc34a2 | solute carrier family 34 (sodium phosphate), | 0.00312492 | 0.030043085 | -3.244952769 | 0.044964 | 1 |
| BC021767 | cDNA sequence BC021767 | 0.008178397 | 0.085162662 | -3.258597785 | 0.041381 | 1 |
| Rdh1 | retinol dehydrogenase 1 (all trans) | 0.003733345 | 0.038916853 | -3.26113767 | 0.035436 | 1 |
| Rsph6a | radial spoke head 6 homolog A (Chlamydomc | 0.012154355 | 0.118538781 | -3.314283227 | 0.011411 | 1 |
| Gm44321 | predicted gene, 44321 | 0.005120909 | 0.055836366 | -3.326421801 | 0.033665 | 1 |
| Gm47692 | predicted gene, 47692 | 0.013522622 | 0.147149862 | -3.328202961 | 0.037927 | 1 |
| Tmem184a | transmembrane protein 184a | 0.005541101 | 0.055560847 | -3.328213915 | 0.038034 | 1 |
| Lrp8os2 | low density lipoprotein receptor-related pro | 0.023344004 | 0.23743262 | -3.367840236 | 0.000216 | 0.028228 |
| Tnfrsf26 | tumor necrosis factor receptor superfamily, r | 0.004543255 | 0.051464329 | -3.37106074 | 0.031892 | 1 |

| | | | | | | |
|---|---|---|---|---|---|---|
| Gm34721 | predicted gene, 34721 | 0.009318591 | 0.095052912 | -3.386570327 | 0.012196 | 1 |
| 4933431C1 | RIKEN cDNA 4933431C10 gene | 0.011589293 | 0.132367383 | -3.399371164 | 0.036336 | 1 |
| Otx2os1 | orthodenticle homeobox 2 opposite strand 1 | 0.022547655 | 0.23842439 | -3.419972842 | 0.004092 | 0.161621 |
| Gm853 | predicted gene 853 | 0.012636296 | 0.141394397 | -3.487056908 | 0.036484 | 1 |
| Sox6os | SRY (sex determining region Y)-box 6, opposit | 0.034809988 | 0.432399049 | -3.496099628 | 0.038831 | 1 |
| Gm49373 | predicted gene, 49373 | 0.00751681 | 0.095037874 | -3.537280174 | 0.026701 | 1 |
| Uncx | UNC homeobox | 0.02331026 | 0.275571695 | -3.558035161 | 0.000503 | 0.048481 |
| Akr1c19 | aldo-keto reductase family 1, member C19 | 0 | 0.016412355 | -3.567635969 | 0.049818 | 1 |
| Pde6a | phosphodiesterase 6A, cGMP-specific, rod, al | 0.003233841 | 0.0436846 | -3.616315684 | 0.020172 | 1 |
| Chil5 | chitinase-like 5 | 0.009688665 | 0.1301356 | -3.625228506 | 0.016466 | 1 |
| Gm38973 | predicted gene, 38973 | 0 | 0.116308442 | -3.629567897 | 0.04535 | 1 |
| Gm47372 | predicted gene, 47372 | 0.004139967 | 0.055781122 | -3.633943849 | 0.024974 | 1 |
| Gm2670 | predicted gene 2670 | 0.005232541 | 0.072405975 | -3.666856043 | 0.022914 | 1 |
| Gm42572 | predicted gene 42572 | 0.004343412 | 0.060467647 | -3.682843401 | 0.012511 | 1 |
| Tctex1d1 | Tctex1 domain containing 1 | 0.005005202 | 0.070925072 | -3.704398431 | 0.015953 | 1 |
| Prss30 | protease, serine 30 | 0 | 0.055164734 | -3.735342738 | 0.033412 | 1 |
| Tacstd2 | tumor-associated calcium signal transducer 2 | 0.00845651 | 0.125196212 | -3.765906006 | 0.012995 | 1 |
| 4933437G1 | RIKEN cDNA 4933437G19 gene | 0 | 0.095492351 | -3.775079708 | 0.048047 | 1 |
| Gm23441 | predicted gene, 23441 | 0 | 0.88866207 | -3.775664362 | 0.048689 | 1 |
| Gm23297 | predicted gene, 23297 | 0 | 0.879439229 | -3.778662072 | 0.047319 | 1 |
| Gm14414 | predicted gene 14414 | 0.051030349 | 0.725375751 | -3.814273464 | 0.005659 | 1 |
| Gm26753 | predicted gene, 26753 | 0 | 0.04226741 | -3.816229297 | 0.041982 | 1 |
| Gm32635 | predicted gene, 32635 | 0 | 0.099018664 | -3.819134266 | 0.045289 | 1 |
| AC154218. | novel transcript | 0 | 0.074566902 | -3.827692518 | 0.046479 | 1 |
| Sstr5 | somatostatin receptor 5 | 0.010699008 | 0.154181715 | -3.842281661 | 0.00222 | 1 |
| Gm49330 | predicted gene, 49330 | 0.116444799 | 1.817219297 | -3.851695194 | 0.008299 | 1 |
| Lbhd2 | LBH domain containing 2 | 0.28858683 | 4.124828385 | -3.85193264 | 0.000174 | 0.024665 |
| Gm28119 | predicted gene 28119 | 0.049235049 | 0.771134093 | -3.856247463 | 0.020318 | 1 |
| Myl3 | myosin, light polypeptide 3 | 0 | 0.058694428 | -3.865286476 | 0.022954 | 1 |
| Clca1 | chloride channel accessory 1 | 0 | 0.031602663 | -3.866184595 | 0.03895 | 1 |
| Gm35024 | predicted gene, 35024 | 0 | 0.124589512 | -3.877134126 | 0.038278 | 1 |
| Gm37573 | predicted gene, 37573 | 0 | 0.132157658 | -3.895431627 | 0.042016 | 1 |
| Gm47595 | predicted gene, 47595 | 0 | 0.315567855 | -3.920396942 | 0.022955 | 1 |
| 6430500D0 | RIKEN cDNA 6430500D05 gene | 0 | 0.107701509 | -3.935665311 | 0.046905 | 1 |
| Oxt | oxytocin | 0.054451378 | 0.863816705 | -3.973131655 | 0.006784 | 0.208472 |
| Gm14323 | predicted gene 14323 | 0 | 0.207245796 | -3.986350568 | 0.031805 | 1 |
| Gast | gastrin | 0 | 0.282494717 | -3.999120011 | 0.031226 | 1 |
| Soat2 | sterol O-acyltransferase 2 | 0 | 0.059640601 | -4.017139488 | 0.016898 | 1 |
| Gm47403 | predicted gene, 47403 | 0 | 0.049054786 | -4.039187783 | 0.030759 | 1 |

| | | | | | | |
|---|---|---|---|---|---|---|
| 4930572O: | RIKEN cDNA 4930572O13 gene | 0 | 0.173089306 | -4.042163845 | 0.035665 | 1 |
| Gm15884 | predicted gene 15884 | 0 | 0.210129545 | -4.044116549 | 0.017176 | 1 |
| Sh2d4a | SH2 domain containing 4A | 0.005388191 | 0.098019734 | -4.070515542 | 0.008271 | 1 |
| Zfp819 | zinc finger protein 819 | 0 | 0.056137 | -4.077637425 | 0.042349 | 1 |
| Defb22 | defensin beta 22 | 0 | 0.22245972 | -4.081904474 | 0.025556 | 1 |
| Gm15943 | predicted gene 15943 | 0 | 0.036138224 | -4.088014266 | 0.023817 | 1 |
| Zfp966 | zinc finger protein 966 | 0 | 0.047364082 | -4.104476246 | 0.041741 | 1 |
| Gm9908 | predicted gene 9908 | 0 | 0.31565738 | -4.108054722 | 0.047426 | 1 |
| Gm42868 | predicted gene 42868 | 0 | 0.052202693 | -4.110766458 | 0.013791 | 1 |
| Tsga13 | testis specific gene A13 | 0 | 0.085462435 | -4.121952529 | 0.027177 | 1 |
| Gcgr | glucagon receptor | 0 | 0.054070315 | -4.122164734 | 0.013541 | 1 |
| Gm4799 | predicted gene 4799 | 0 | 0.217228623 | -4.125843329 | 0.011866 | 1 |
| Gm9522 | predicted gene 9522 | 0 | 0.155342458 | -4.129914165 | 0.038665 | 1 |
| Gm43210 | predicted gene 43210 | 0 | 0.023290959 | -4.135659035 | 0.014114 | 1 |
| Trim10 | tripartite motif-containing 10 | 0 | 0.065640495 | -4.172454368 | 0.023356 | 1 |
| Slc18a1 | solute carrier family 18 (vesicular monoamin | 0.002716542 | 0.05358617 | -4.186145587 | 0.0056 | 1 |
| Mrgprh | MAS-related GPR, member H | 0 | 0.079936647 | -4.256442136 | 0.01479 | 1 |
| D730003K: | RIKEN cDNA D730003K21 gene | 0 | 0.185450278 | -4.269090102 | 0.015499 | 1 |
| Bpifc | BPI fold containing family C | 0 | 0.057382925 | -4.330530914 | 0.032288 | 1 |
| Dpt | dermatopontin | 0.008510467 | 0.193362934 | -4.382495821 | 0.00231 | 1 |
| AC126280. | novel transcript | 0 | 0.058000219 | -4.387686921 | 0.004971 | 1 |
| 2610318M | RIKEN cDNA 2610318M16 gene | 0 | 0.301775169 | -4.424678007 | 0.014394 | 1 |
| Gm49427 | predicted gene, 49427 | 0 | 0.312327844 | -4.438897533 | 0.009232 | 1 |
| Rbp7 | retinol binding protein 7, cellular | 0 | 0.248008712 | -4.450386521 | 0.005855 | 1 |
| Gm23706 | predicted gene, 23706 | 0 | 2.30464339 | -4.531189645 | 0.017492 | 1 |
| Glyat | glycine-N-acyltransferase | 0 | 0.039762506 | -4.544517228 | 0.019624 | 1 |
| 4930429P: | RIKEN cDNA 4930429P21 gene | 0 | 0.084823957 | -4.56757211 | 0.002927 | 1 |
| Cyp4f41-ps | cytochrome P450, family 4, subfamily f, poly | 0 | 0.191353869 | -4.655401408 | 0.002113 | 1 |
| Gm26725 | predicted gene, 26725 | 0 | 0.207256141 | -4.826670064 | 0.006265 | 1 |
| C1ql4 | complement component 1, q subcomponent | 0.009918721 | 0.327454172 | -4.926737959 | 0.00325 | 0.142987 |
| 4933429O: | RIKEN cDNA 4933429O19 gene | 0.004891333 | 0.211818752 | -5.320091163 | 0.000326 | 0.037361 |
| Calcr | calcitonin receptor | 0 | 0.083754848 | -5.321484615 | 0.00134 | 1 |
| Prdm12 | PR domain containing 12 | 0.00633963 | 0.262354586 | -5.349460753 | 0.029205 | 0.389312 |
| Frmpd1os | FERM and PDZ domain containing 1, opposit | 0.035229385 | 1.618788579 | -5.404039371 | 0.000224 | 0.028799 |
| Irx2 | Iroquois homeobox 2 | 0.013395577 | 0.587732941 | -5.45799689 | 0.00019 | 0.025896 |
| Xist | inactive X specific transcripts | 0.004893841 | 14.76302222 | -11.56198294 | 5.03E-13 | 1.77E-09 |

| MM DEGs relative to SS | | MS DEGs relative to SS | | SM DEGs relative to SS | |
|---|---|---|---|---|---|
| Upregulated | Downregulated | Upregulated | Downregulated | Upregulated | Downregulated |
| Tbr1 | Hap1 | Tsc22d3 | Slc16a1 | Wdfy1 | Crocc2 |
| Mex3a | Dlk1 | Ppp1r3g | Cirbp | Gm44799 | Ak7 |
| Stxbp6 | Resp18 | Gm19439 | Gm14414 | Gm47623 | Dnah6 |
| Cadm2 | Tmem255a | Mertk | Hmgcs2 | Gm14776 | Ccdc153 |
| Kcnk1 | Xist | Edn1 | Aplnr | Gm42970 | C1qtnf3 |
| Maml2 | Ngb | Slc2a1 | Aldh1a2 | Egr1 | Col1a2 |
| Sema5a | AW551984 | Zbtb16 | Bbc3 | Mid1 | Mxra8 |
| Prox1 | Dio3 | Hif3a | Amd-ps3 | Trib1 | Ttc21a |
| Pkia | Sln | Atp10a | Mst1r | Ppp1r3g | Myoc |
| Nfia | Zcchc12 | Lrrc8c | Sncg | Mertk | Dnah11 |
| Btbd3 | Rec8 | Cdkn1a | BC051019 | Gm26736 | Lrrc23 |
| Fam163b | Gprasp2 | Trp53inp1 | Fam117a | G530011O06Rik | Col1a1 |
| Ccbe1 | Hrh3 | Kctd16 | Ccdc107 | Npas4 | Vstm2l |
| Gm17322 | Nr2f2 | Gm14776 | Psmg4 | Sall3 | Amd-ps3 |
| Lrrtm4 | Rsph4a | Gm44799 | Rarres2 | Gm12816 | Zic2 |
| Npas4 | Slc32a1 | Per2 | Abhd16a | Fosb | Fam117a |
| Iyd | Arhgap6 | Gm8995 | Lmo1 | Gm7887 | Cfap54 |
| Jph1 | Ecel1 | Sgms1 | Mfap5 | Ikzf1 | Pld5 |
| Skil | Col23a1 | Inpp5d | Sox12 | Tbr1 | A2m |
| Epha4 | Dcn | Aff1 | Efemp1 | Hsd11b1 | Nme9 |
| Mfsd4a | Tacr3 | 2900022M07Rik | Abcb6 | Gm20692 | Ankrd55 |
| Bhlhe22 | Cbln4 | Pik3r1 | Slc16a11 | Satb2 | Resp18 |
| Dcx | Tmem130 | Adipor2 | Nanos2 | Gm19445 | Gabre |
| Sema3c | 6820408C15Rik | Satb1 | Gabre | Csmd1 | Armc4 |
| Kcnj6 | Ttc21a | Npas4 | Zswim5 | Bend5 | Lum |
| Dtna | Crocc2 | Smim3 | Tmem11 | Mpeg1 | A530072M11Rik |
| Mkx | Nrsn2 | Rin2 | Cygb | Camkk2 | Col9a1 |
| Orai2 | Baiap3 | Gm47623 | Mymk | Irak2 | Zfp474 |
| Arhgap20 | Vstm2l | Igf1r | Slc26a11 | Dusp6 | Tekt1 |
| Hsd11b1 | Mrap2 | Mat2a | Gjb2 | Gm6058 | 2410004P03Rik |
| Adcy1 | Cfap161 | Tekt4 | Ajuba | Ccbe1 | Fam166b |
| Plxna4 | Gap43 | Trib1 | Ifitm10 | Gm12250 | AW551984 |
| Mef2c | Zfp941 | Pabpc5 | Slc6a20a | Inka2 | Frmpd2 |
| Ano3 | Peg10 | Rnf144b | Cfap161 | Gm43322 | Cp |
| Lyst | Magel2 | Klf9 | Gm15706 | Hexb | Mycbpap |
| Egr1 | mt-Th | Gjb6 | Crip1 | Fcgr1 | Gm16201 |
| Grm2 | Hs6st2 | Ikzf1 | Shisal2a | Lpcat2 | Efcab12 |
| C1ql3 | Gpx3 | Hip1 | Lrrc23 | Tmem181b-ps | Cdhr3 |
| Cyp26b1 | Cfap70 | Dgki | Spsb4 | Gm42853 | Armc3 |
| Sphkap | Nap1l5 | Gm42772 | Mcrip2 | AC165079.1 | Enpp1 |
| Thbs4 | Sp9 | Adra1a | Gm48536 | Eva1a | Serping1 |
| Gm42772 | Dynlrb2 | Cttnbp2nl | Crygn | Isg15 | Gm19461 |
| Mcm6 | Radil | Tbc1d4 | 2810459M11Rik | Tmem181c-ps | Slc5a8 |
| Slc39a6 | Rftn1 | Htra1 | Tspan4 | Gm44228 | Sncg |
| Hnrnpa3 | Cbln1 | Kirrel2 | C4b | Arc | Tmem267 |
| Epha7 | Col6a1 | Mast4 | Atf1 | 4931413K12Rik | Pnma3 |
| Csmd1 | 1700007K13Rik | Foxo3 | Bbox1 | Dtl | Hap1 |
| Trhde | Tmie | Fosb | Mgst1 | Gramd2 | Gas2l2 |
| Zcchc14 | Timp2 | Csmd1 | Col9a2 | Gm42488 | Cercam |
| Dclk3 | Wnt4 | Rtp1 | Sf3b5 | Dock8 | Six3 |
| Kbtbd11 | Vegfd | Mast2 | Myoc | Gm44230 | 4833423E24Rik |
| Plekha2 | Scn5a | Npas2 | Crnde | Gm13835 | Gm29771 |
| Zbtb18 | Oxtr | Nav3 | Khk | Hapln1 | Dnah10 |
| Mkl2 | Rsph1 | Fzd2 | Tmem198b | Fbxw7 | Rsph4a |
| Il20rb | Otof | Hspa5 | mt-Ts2 | Gm11549 | Musk |
| Sh3kbp1 | Dync2li1 | Gm26801 | Krt15 | Gm15975 | Col3a1 |
| Dgki | Tmem200c | Pten | 1700001C02Rik | Tmem119 | Crocc |
| Prickle2 | Gaa | Irak2 | C1qtnf3 | St6gal2 | Lrrc36 |
| Sox11 | Tcerg1l | Eva1a | Aox3 | Rasal3 | Nkx6-1 |
| Fstl4 | Tmem179 | Homer1 | Ergic3 | Cd47 | Jhy |
| Prkce | Dnah6 | Myrip | Zan | A830082K12Rik | Cckar |
| Zbtb20 | Necab2 | Spire1 | Tmie | Rnf17 | Gm17455 |
| Epha6 | Gabra3 | Gm42853 | 1700024G13Rik | Gm15521 | Aplnr |
| Frmd4b | Irs4 | Gm15721 | Glrx5 | Gm44633 | Irx3 |
| Nexmif | Wdr6 | Fmo2 | Scn7a | Pvrig | Gm4593 |
| Ccdc71l | Maats1 | Clic5 | Mdfi | Cyr61 | Ccdc170 |
| Dgkh | Cpne2 | Fas | Ppie | Spry2 | Cd24a |
| Il1rap | Myh7 | Fam171b | Cplx4 | Ap3m1 | Lhx5 |
| Zfp81 | Cfap54 | Med13l | Rdh10 | Gm26801 | Mir541 |

| | | | | | |
|---|---|---|---|---|---|
| Gm44799 | Cpne7 | Usp31 | Eif6 | Setbp1 | Bmp6 |
| Pcdhgb1 | Tmem91 | Lrrc7 | Ndn | Vcl | Uncx |
| Ago1 | Efcab1 | Rpe65 | Dusp9 | Osgin2 | Cfap161 |
| Hpdl | Adgra1 | Galnt15 | Rabac1 | Map4k5 | Zfp467 |
| Rfx3 | Foxj1 | Nostrin | Nbl1 | Gm20172 | 1700012B09Rik |
| Megf10 | Fam216b | Kcnj2 | Efna4 | BC049352 | Ptpn13 |
| Fat4 | Vgll3 | Id2 | Il13ra2 | Gm2366 | Aox3 |
| Rasa1 | Hba-a2 | Ctgf | Wnt7a | P2ry13 | Fam216b |
| Atad2b | Sncg | Arhgap26 | Pipox | Etv5 | Gm10610 |
| Nol4 | Cthrc1 | Ly6c1 | Gm29771 | Arsb | Tm4sf1 |
| Sertad4 | Ak7 | Hivep3 | Cpsf4 | 2900022M07Rik | Rasa4 |
| Gm14776 | Oprk1 | Mprip | Rab4b | Gm9828 | Gm44751 |
| Tex13c2 | Hdac11 | G630022F23Rik | mt-Th | AI464131 | Loxl2 |
| Dhx33 | Chst8 | Gm44228 | Lhx5 | Camk2n1 | Ccdc162 |
| Rbfox1 | Pnkd | Gm26736 | Hint2 | Gm17039 | Iqca |
| Cdkn1b | Atg9b | Dlgap2 | Flnc | Mkx | Crygn |
| Setbp1 | Rnf227 | A830009L08Rik | Slc19a1 | Gm22933 | Gm48865 |
| Slc16a7 | Gabrq | Chrdl1 | Radil | Ppp2r5c | Mrap2 |
| Frrs1l | Cfap45 | Sgk1 | Tmem106c | Cdc27 | Adm |
| Mid1 | Npffr1 | Itpr1 | Ppcs | Gm13868 | Foxj1 |
| Gm8738 | Lbhd2 | Itsn2 | Iqca | Gm36963 | Flnc |
| Slc35f3 | Rpp25 | Sdf2l1 | Gm13889 | Hpgds | Fsd2 |
| Pappa2 | Irx2 | Myh3 | Ggact | Htra1 | Hydin |
| Spata13 | Gpr101 | Tcap | Smdt1 | Tmem178 | Peg10 |
| Dagla | Lrp8os2 | Osbpl3 | A730017C20Rik | Irf8 | Cfh |
| Inka2 | Tspan33 | Pml | Klhdc8b | Selplg | 2310034G01Rik |
| Gm42970 | Spag16 | Bmp3 | Tdrp | Creg2 | Nid1 |
| Pitpnm2 | Frmpd1os | Tlr13 | Hes1 | Foxp1 | Pkdrej |
| Ptprz1 | Cdh13 | Itgav | Ostf1 | Ros1 | Trh |
| Atrx | Gm28729 | Ovol2 | Gm17455 | Dkkl1 | Plppr3 |
| Kctd4 | Zwint | Rab11fip4 | Rec8 | 3100002H09Rik | Fbxw10 |
| Gm44228 | Bace2 | AC165271.1 | Plekhj1 | P2ry6 | Klhl1 |
| Neurod1 | Smim5 | Shank2 | Ppp1r32 | Xlr4a | Traf1 |
| Grin2a | Epb41l4a | Zfp365 | Gm10687 | Irgm2 | Cma1 |
| Mycl | Cfap52 | Dio2 | Dync2li1 | Fam107a | Gm19935 |
| E2f3 | Prelp | Mkx | B630019A10Rik | Sult1a1 | Myadml2 |
| Ptx3 | Klhl1 | Acot11 | Frs3 | Lgals9 | Nkx2-1 |
| Slc8a2 | 4933429O19Rik | Pcsk1 | Traf1 | Nav3 | Irs4 |
| Smim3 | Ankrd24 | Acsl3 | Otoa | Stx1a | Tctex1d4 |
| Bcl11b | 2410004P03Rik | Arhgap20 | Inhbb | 1810028F09Rik | Cfap57 |
| Klk8 | Rtl8c | Fgfr3 | Zcchc12 | Gm37529 | Cd55 |
| Ccr6 | Gas6 | Gm6058 | Neurl2 | Map3k5 | Col5a3 |
| Rasal2 | 6330403K07Rik | Arrdc2 | Kdelr2 | Gm44518 | Wls |
| Il16 | Spats2l | AC098880.2 | Mir23b | Vopp1 | Tbx18 |
| Plxna2 | Nnat | Usp54 | Rnase1 | Gm47155 | Thbs2 |
| Ptpro | Samd14 | Havcr2 | Amdhd2 | Atp6v0c-ps2 | Got1l1 |
| Mef2a | Armc3 | Lonrf3 | Frmpd2 | C530025M09Rik | Dnali1 |
| Cdh8 | Impact | Net1 | Tmem9 | Nr4a1 | Ces1d |
| Klf13 | Rsph9 | Ipcef1 | Copz2 | Gm42772 | Pih1h3b |
| Mpeg1 | Dmac2 | Cyr61 | Ctxn2 | Cdc45 | Gm39244 |
| Rlf | Uncx | Arap2 | Gm47692 | Pde8b | Ccdc146 |
| Dgkz | Zdbf2 | Gm5415 | Morn1 | Pclaf | Gm5127 |
| Sypl2 | Crocc | Dclre1a | Hic1 | Tlr13 | Rgs22 |
| Cacna1h | Npr3 | Mapk4 | Resp18 | Gm5640 | Tmem106c |
| Kif18a | Klc4 | Dclk1 | Abhd14b | Tyrobp | Chil5 |
| Tgfbr1 | Oprl1 | Tmod2 | Lrrc32 | Trps1 | Spag8 |
| Atf6 | Bbox1 | Slmap | Prdx5 | Mettl11b | Six3os1 |
| Atp2b1 | Tekt1 | L3mbtl3 | Lrrc29 | Asap1 | Epb41l4a |
| Igfbpl1 | Htr2c | Stxbp5l | Trip6 | Gnb1 | Sln |
| Thumpd2 | Rasa4 | Pde8b | Glis1 | Gm5869 | Clec3b |
| Exoc6 | Ldb2 | Kcnf1 | Mdk | Trim43a | Rsph9 |
| Ptprj | Mme | Nol4 | Srp14 | Nde1 | 4930556M19Rik |
| Tgfa | Elof1 | Chat | Nop10 | Arpp19 | Il13ra2 |
| Smchd1 | Rit2 | Cables1 | Nle1 | Tfrc | Sdr39u1 |
| Lrrc8b | Kcnk9 | Magi2 | Gm14964 | Apol11b | mt-Ts2 |
| Prkx | Efcab12 | Samd12 | Katnal2 | 1600012H06Rik | Dynlrb2 |
| Enc1 | Btbd11 | Dopey2 | Npas3 | Tle4 | Eno4 |
| Cblb | Grp | Car8 | Pigyl | Tex13c2 | Cdkn1c |
| Npnt | 1700037H04Rik | Gm11549 | Emc10 | Ddx58 | Mdfi |
| Klf9 | Zfp474 | Cmtm3 | Fndc8 | Vsir | Ifitm1 |
| 3300002I08Rik | Myb | Fam126b | Serping1 | Homer1 | Slc22a4 |

| | | | | | |
|---|---|---|---|---|---|
| Rtl3 | Mmab | F3 | Ptgdr | Csf1r | Frem3 |
| Tmem170b | Lrrc23 | Klhl34 | Gm48632 | Fos | Gm19619 |
| Bag4 | Ubxn11 | Cdh19 | Rpia | Dtna | Gm48632 |
| Zeb2 | Gm14204 | Tacc1 | Ddt | AC165271.1 | Slc6a4 |
| 4921524J17Rik | Unc5d | Smarca2 | Vasp | Mcur1 | Pou4f1 |
| Ncan | Ly6h | Gm16485 | Coa3 | Iyd | Slc39a4 |
| Tmem181c-ps | Slc5a5 | Tshz3 | B230312C02Rik | Gm36251 | Coch |
| Add2 | Ache | Adcy1 | Gm44109 | Isg20 | Zcchc12 |
| Dab1 | Tac1 | Abcb1a | Sox1 | Rpl3-ps1 | Serpinb1b |
| Tcf4 | Cd55 | Fstl4 | Anxa5 | Gm7237 | Trac |
| Pak7 | Stk32b | Nfkbia | Emc9 | Entpd1 | Bmp2 |
| Trib1 | Lrsam1 | Fst | Gm48408 | Cd5 | Hsd11b2 |
| Itsn2 | 1700016K19Rik | Ncoa2 | Fam216b | Gm26670 | Tmem130 |
| Chd1 | Cox8b | Fut9 | Mrpl57 | Slc1a3 | 1700024G13Rik |
| Ddx19a | Htr7 | Sipa1l1 | Rsph9 | Rc3h2 | Krt18 |
| Tmem144 | Hbb-bs | Chsy3 | Mrps28 | Gm7476 | Tgfbr3 |
| Tiam2 | Tox2 | Klhl38 | Pcgf2 | Zfp738 | Ccdc187 |
| Tnfrsf19 | Crygn | Slc2a13 | Selenom | Csrnp1 | Tmem212 |
| Slc7a14 | Myoc | C230034O21Rik | Cpxm1 | Tmem144 | Lrrc71 |
| Ptbp3 | Emb | Gm44873 | Gm28373 | Thbs4 | Baiap3 |
| Fam19a2 | B230312C02Rik | Arc | B230319C09Rik | Gldc | Tmem176a |
| Pclaf | Nek5 | Zfp101 | Efna3 | Egr2 | Ttll6 |
| Auts2 | Slc7a3 | Gm15398 | Ankrd55 | Ppm1k | Daw1 |
| Rgs7bp | Gm29771 | Dlg2 | Cryzl2 | Cd84 | Acox2 |
| Zfp882 | Cyb561 | Sema3a | Cd320 | Capn11 | Col18a1 |
| Trps1 | Kcnh2 | Tiparp | Gabrr1 | Gm20632 | Mlycd |
| Ifi203 | Gjd2 | Arhgap31 | Polr2l | Rasgef1c | Efhb |
| Plcl1 | Irx3 | Etl4 | Rps6ka1 | Nuggc | Tpm2 |
| Cemip | Rarres2 | Sptbn2 | Tssc4 | Gm34237 | 1110017D15Rik |
| Nsl1 | Calcr | Klf15 | Samd1 | Ifi44 | Dnah12 |
| Fmr1 | Pcbd1 | Ulk2 | mt-Tg | Gbp3 | Gm17720 |
| 2810025M15Rik | Fhl1 | Plxna2 | Vstm2l | Gm30382 | Slc36a2 |
| Islr2 | Plagl1 | Farp2 | Aspg | Iqcn | Cd163 |
| Bmpr2 | Myo16 | Ncor1 | Ier5l | Gm13688 | Lhb |
| Gm42600 | Nipal2 | Camk4 | Mab21l1 | Pak7 | Gm22596 |
| Sipa1l3 | Dnaic2 | Ccdc141 | 2010001K21Rik | Prkd1 | Krt15 |
| Cnot6 | Traf1 | Ubxn2b | Pcbd1 | Gm35552 | Klc4 |
| Nrxn1 | Gm19935 | Mxi1 | Ppdpf | Havcr2 | Gm10076 |
| Lhfpl2 | AU023762 | Abcc9 | Pkdcc | Trub2 | Gm15478 |
| Kdm7a | Eif2s3x | Fnbp1l | Serpinb9 | Nr2e1 | Sdc1 |
| Abcc9 | Dnali1 | Per1 | Ascc1 | Gm9761 | 4930523C07Rik |
| Fnip2 | Mapk15 | 9330182L06Rik | Gm15853 | Gm43355 | Trp73 |
| Akt3 | Arl2 | Gm42970 | Dnah12 | 4930488L21Rik | Bgn |
| Trim2 | Atp5h | Gfod1 | Spata24 | Hck | Ccdc180 |
| Tgs1 | Pld5 | Ocm | Psma7 | Gm28723 | Slc6a5 |
| Spry2 | Drc3 | Gm42848 | Riiad1 | Zfp729a | C3 |
| Nfib | Vat1l | Ankhd1 | 2410022M11Rik | 8-Sep | Ccdc160 |
| Syncrip | Irx5 | Xkr4 | 4933440N22Rik | Pemt | Lncenc1 |
| Ccdc85a | Hba-a1 | Pde4c | Cyp2j8 | Snord104 | Tnc |
| Sema5b | Capsl | Rims1 | Pax7 | Exoc6 | Postn |
| Olfml2b | Rab3c | Cdc42bpa | Gjb3 | Wdr1 | Krt19 |
| Ncald | Cbx7 | Plekhf1 | Cyb561d2 | Ndufaf4 | Serpinf1 |
| Uba1y | Zfp423 | Setd7 | Slc40a1 | Myrip | Cox8b |
| Kbtbd7 | Edn3 | Tfrc | Rpa2 | Cx3cr1 | Sh3bgrl2 |
| Rasgrf2 | Efna5 | BC006965 | Tbcc | Mid1-ps1 | Gprasp2 |
| Nedd4l | Camk2d | Frmpd1 | Dpm3 | Atf2 | Ppp1r36 |
| Lct | Ankrd34b | Col19a1 | Pwwp2b | Gnao1 | Pitx2 |
| G530011O06Rik | Grb10 | Abcg2 | Anxa2 | Klhl41 | Isl1 |
| Als2 | Dnah7a | Cited4 | Gm5373 | Dnajb7 | Pcolce |
| Abi2 | Ubxn10 | Btbd8 | Srpk3 | Gm43813 | Wdr93 |
| Stim2 | Scube3 | Fzd4 | Eif3i | Gm3279 | Pvalb |
| Dock8 | Sox1ot | Hivep2 | Morn2 | Xlr4b | Csad |
| Egr3 | Cyp4f41-ps | Lrrc8b | Gm29154 | Incenp | Lrrc74b |
| Gpr155 | Rxrg | Gm45222 | Fgl2 | Gm45222 | Ccdc40 |
| Syt10 | Dlx6 | Ssh2 | Eppk1 | Dennd1b | 9530085L11Rik |
| Slc1a2 | Ccdc146 | Nrxn1 | Sdhc | Xk | Crispld2 |
| Epc2 | Tekt2 | 5330417C22Rik | Gm25492 | Dio2 | Gm48536 |
| Pde8b | Sstr5 | Setbp1 | Naxe | Rab43 | Gm23706 |
| Wdr5 | Nenf | Uhrf1bp1l | Necab2 | Ube2cbp | Ctbp2 |
| Tiam1 | Cfap206 | Vcl | Tmem130 | Nufip2 | Capn6 |
| Kalrn | Gga1 | Jcad | Gm20684 | AC165278.1 | Gm37968 |

| | | | | | |
|---|---|---|---|---|---|
| Skida1 | Fam196b | Map3k5 | Gata3 | Gm48614 | Fbln1 |
| Klf3 | Nkd2 | Dtl | Ppp4c | Kcnj2 | Cxcl16 |
| Ttc28 | Nog | 5930438M14Rik | Selenbp1 | Sgms1 | Slc25a5-ps |
| AC165271.1 | Dpt | Klf13 | 1810044D09Rik | Cyp4f14 | Gm43272 |
| Napepld | Ifit3b | Arhgap32 | Id3 | Edrf1 | Igsf1 |
| Ddx21 | Iqca | Asap1 | Ptov1 | Creld2 | Col4a3 |
| Vcan | Gm11992 | Kcnh7 | 2210406H18Rik | Usp18 | Adcy8 |
| Gm37529 | Otx2 | Zfp445 | Serpinf1 | Trim30a | Scn5a |
| Lrrc58 | Ppcdc | 2810021J22Rik | Dera | Glrp1 | Calca |
| Ubash3b | C230072F16Rik | Ctnnd2 | Pnma3 | Gm4202 | Rbp2 |
| Creg2 | Rasgrp2 | Ankrd33b | Cpa6 | Csnk2a1 | Aifm3 |
| Epha5 | Mdh1b | Serpinb8 | Efnb3 | Lamp5 | Efnb1 |
| Ncapg | Ptpn5 | Ppargc1a | Rbm3 | Aplf | Tgfb3 |
| Btbd9 | Scn9a | Lrrc58 | Fam90a1b | Ica1l | Mr1 |
| Nr3c2 | Cfap57 | Satb2 | Colca2 | Rims1 | AC158985.1 |
| Susd5 | Hbb-bt | Asap2 | Zswim3 | Cmip | Col5a2 |
| Spsb1 | Abhd14b | Serinc3 | Tal1 | Gm7292 | Calb2 |
| Fbxw11 | Rd3 | Gm43355 | Lrrc43 | Kcnh7 | Gm14703 |
| Inhba | Camk1 | Gm43322 | Gast | Gm48042 | Gm26902 |
| Eva1a | Lin28b | Gm19410 | Slc35b2 | Gm37320 | Spag17 |
| Card6 | Cfap58 | Grik3 | Paip2 | Pla1a | Gm18001 |
| Plppr5 | Cyp26a1 | Frmpd4 | Tceal1 | P2ry12 | Bbox1 |
| Sorl1 | Tmem106c | Tanc2 | Smco4 | 9230112E08Rik | Gpr149 |
| Camk2b | Trmt9b | Hecw2 | Gm14323 | Pik3cg | Sema3b |
| Fat3 | Dpp10 | Wee1 | Syt3 | Cst6 | Csrp2 |
| Nhlh2 | Pygm | Rin3 | Tmem121 | Gm37124 | Gm16083 |
| Pdgfra | Krt18 | Sptbn5 | 9530052E02Rik | Smim3 | Foxd1 |
| Shisa6 | Capn6 | Exph5 | Cisd1 | Fcer1g | Pax8 |
| Nck2 | 4930429P21Rik | P2ry14 | Tarm1 | Opcml | Sh2d3c |
| Foxo1 | Iqcg | Socs7 | P3h4 | Ascl1 | Gm37422 |
| Dusp6 | Slc39a4 | Ptprt | Gm12407 | Rnf6 | Prr29 |
| Ankrd45 | Postn | Pmaip1 | 4833423E24Rik | Gm13861 | Scube3 |
| Ncoa6 | Tmem202 | Trim44 | Crocc2 | St8sia5 | Radil |
| Ski | Lhfpl5 | Rap1gap2 | Foxo6os | B530045E10Rik | Rec8 |
| Lin7c | Kctd9 | Oprd1 | Gm26644 | B230303O12Rik | Calcr |
| Cttnbp2nl | Podxl2 | Hecw1 | 1110017D15Rik | Trhde | Gm49427 |
| Prr14l | Plekhb2 | Rhou | Rab34 | AC154486.3 | Irx2 |
| Bdnf | Adh1 | Rbm41 | Ephb4 | Socs7 | Tlr2 |
| Chrna5 | Crb3 | Thbs4 | Zdhhc12 | Zfp668 | Srpk3 |
| Acan | Scml4 | Rai14 | Ifitm2 | Sh2b3 | Alkal2 |
| Gabrb3 | Klhdc8b | Ice1 | Iscu | Gm45901 | Morn3 |
| Pcp2 | C1ql4 | Myo5a | Stoml3 | Sgtb | Cped1 |
| Lrrc10b | Eno4 | Cdh12 | Tsen34 | R3hdm1 | Col12a1 |
| Galnt17 | Gad2 | Lrrk2 | Coro1b | Pcdha3 | Anxa2 |
| Ythdf3 | Frmpd2 | Lrrtm3 | Pdcd2l | Dbil5 | Pcbd1 |
| Nav1 | Gm29675 | Arid5b | 4930577N17Rik | Siah1a | Gm10175 |
| Tanc1 | Mlf1 | Unc79 | Sys1 | Rab6b | Gpr55 |
| Spry1 | Unc119 | Kcnh5 | Slc5a8 | Gm44151 | B230118H07Rik |
| Pdyn | Eml2 | Gm9954 | Lrrc71 | Plaur | Llgl2 |
| Pou3f3 | Cplx1 | Acer2 | Bri3 | Hist1h2bj | Mtss1 |
| Lrrc55 | Krt15 | Espnl | Cda | Gm5415 | Zswim3 |
| Fam171b | Ccdc170 | Fam107a | Cops6 | Dkk3 | C1qtnf6 |
| Ppp4r2 | Tctn2 | Dusp1 | Psme1 | Gm6548 | Dmkn |
| Inf2 | Gm26684 | Ccng1 | Odf3b | Mef2a | Unc5cl |
| Scn3b | Crhbp | Helz2 | Oxld1 | Arid4a | Erich3 |
| Ralgapa2 | Pcgf2 | Neto2 | Myl3 | Pgm2l1 | Gm11992 |
| Gm26736 | Serpina3g | Slc39a10 | Cep164 | Gm47505 | Jph2 |
| Alg10b | B630019K06Rik | Klhl24 | Coq3 | Rad1 | Rnaseh2a |
| Btg2 | Cxcl5 | Agbl4 | Prr14 | Scmh1 | Dnah5 |
| Map3k3 | Dgkk | Gabrb2 | Gm23369 | Dmrta2 | Nkx6-2 |
| Dyrk1a | Tfr2 | Zfp317 | CT010429.1 | Gm42848 | Gm16365 |
| Abhd13 | Ccdc180 | Cacnb4 | Gm26558 | 4930447M23Rik | Adamts5 |
| Cdc40 | Gm32468 | Spsb1 | Gm30025 | Rsad2 | Erich2 |
| Nrp1 | Corin | Zbtb40 | Rela | Btg2 | Sstr5 |
| Epha3 | Kcnt1 | Lnx2 | Trappc2 | Gm7901 | Cd59b |
| Lingo3 | Nol3 | Hlf | Gm6767 | Gm31518 | Ccdc33 |
| Disp3 | Ccdc153 | R3hdm2 | Mad2l2 | Gm4045 | Rit2 |
| Zfp148 | Col25a1 | Gm10334 | Xirp1 | Gm5913 | Tubgcp3 |
| Fam111a | Nmnat1 | Cry1 | Zcchc3 | Gm37824 | Smim11 |
| Foxo3 | Etnppl | Pla1a | Grtp1 | Olfr692 | Ccl11 |
| Epn2 | Tmc4 | Rasal3 | Clec3b | Rnf217 | Lrp2 |

| | | | | | |
|---|---|---|---|---|---|
| Rasgrp1 | Ldhb | Ccnt2 | Gm11985 | Vip | 1700001C02Rik |
| Ppp1cb | Slc35d3 | Pcare | Casc1 | Zfp365 | Slc22a6 |
| Rsf1 | Rpl41 | Hs6st3 | Fgfrl1 | Gm18822 | Syne4 |
| Tle1 | Otx2os1 | Epas1 | Daw1 | Smc4 | CT573017.2 |
| Kcnj2 | Zfp688 | Tmed8 | Ascl2 | Stat4 | Tgfbi |
| Sstr2 | Gm973 | Maff | Col2a1 | Zfp957 | Nap1l5 |
| Cpeb4 | Commd9 | Sgtb | 4933434E20Rik | Msln | Btc |
| Dmrta1 | Abat | Kcnh1 | Gm33609 | Gm43048 | Chrna3 |
| Bmi1 | Spata18 | Zfp760 | Inka1 | Gm20712 | Glipr2 |
| Zfp251 | Cds1 | Ralgapb | Polr2d | 4933406J09Rik | Wdr63 |
| Slc2a13 | Krt77 | Elavl4 | Ptgr1 | Hacd2 | 1-Sep |
| Dip2c | Slc26a11 | Rmnd5a | Mrpl2 | Gm11408 | Retn |
| Syndig1 | Pon3 | Adgrl4 | Cnn1 | Gm16116 | Nle1 |
| Aldh1l2 | Cfap126 | Pnlip | 1700086O06Rik | Gm14411 | Gm37095 |
| Rfx7 | Scg5 | Vps13a | AC126280.1 | Ccr5 | Ist1 |
| Ednra | Gabre | Tmem132b | Gstz1 | Olfr31 | Cpq |
| Syt7 | Sparc | Pcdha5 | Bscl2 | 4930558J18Rik | C230072F16Rik |
| Kcns2 | Gm9866 | Tiam2 | Mlycd | Itsn2 | Fam183b |
| Npy1r | Nudt14 | Kcnb1 | Arhgef16 | Gm40466 | Ccdc113 |
| Ephb2 | Lhb | Cntnap5a | Olfml2a | Foxr2 | Cnmd |
| Mndal | Mab21l1 | Gm36823 | Gm7332 | Hnrnpa3 | Klhl13 |
| Dgkd | Dnah11 | 1700020I14Rik | Got1l1 | D830025C05Rik | Gsto1 |
| Mycn | Ccdc81 | Scube1 | Hpcal1 | Cttnbp2nl | Cfap46 |
| Robo2 | Carmil3 | Sf3b1 | Gm26685 | Slc5a12 | Ecm1 |
| Nectin3 | Ttc29 | Kcna1 | Mycbp | Gcnt4 | Susd2 |
| Mdc1 | Susd2 | Gm12868 | Gm9292 | Tgm3 | Gm11642 |
| Ipcef1 | AC126280.1 | Ccny | Gatd1 | Hmgb2 | Cps1 |
| Adamts17 | Mcrip1 | Ccdc50 | Blvrb | Npas2 | Trim63 |
| Dsp | Trpm2 | Gm19353 | 3110045C21Rik | Ptbp3 | Ankub1 |
| Lrp1b | Pla2g5 | Kcnk1 | Mesd | Gm37848 | Catip |
| Igsf3 | Fuca1 | Dock8 | Tdgf1 | AC174678.1 | Dok2 |
| Xpr1 | Abcf3 | Cacng1 | Bloc1s5 | Gm40493 | Gm4208 |
| Rab40b | Ggact | Ldlrad4 | Wnk4 | Serpina3n | Sparc |
| Pcdhga2 | Snx32 | Mllt3 | Bhlhe41 | Orc4 | Gm15473 |
| Garem1 | Gpsm1 | Pdk1 | Islr | Gm37551 | Zfp455 |
| Carmil1 | Mycbpap | Vopp1 | Adsl | Sema3a | Mdh1b |
| Rab10 | Slc18a1 | Unc5d | Prtn3 | Arhgap25 | Spata18 |
| Igf1r | P3h1 | Tbr1 | Klf10 | Gm40123 | Gm42427 |
| Itpr2 | Gm14414 | Ralgps1 | Pgap2 | Dlgap2 | Ecel1 |
| Gm5881 | Lmx1a | Rarb | Gm11646 | Gpd1l | Rtl1 |
| Htra1 | Tmem212 | Gm11762 | Tead2 | Snora17 | Pde6g |
| Ppp3ca | Coprs | Prkcb | Zcchc18 | Rtl3 | Spsb4 |
| Hipk4 | Rbp7 | Xbp1 | Gm19426 | Gm5398 | Vwa3a |
| Fry | C1qtnf3 | Negr1 | Foxo6 | Gm47096 | Zfp423 |
| Myt1l | Megf11 | Igsf11 | Clec14a | Nectin3 | Kcnh2 |
| Dopey2 | Spire2 | Zfp644 | Thnsl2 | Ly6c1 | Smpx |
| Pter | Spata24 | Ttyh3 | Sox13 | Tiparp | Stac |
| Arhgef33 | Ttc25 | Arhgap35 | Krtcap2 | Dab1 | Pabpc1l |
| Msl2 | Cdkn1c | 1700047F07Rik | Gm42427 | 9630010A21Rik | Dlec1 |
| Cacna2d1 | Gm26725 | R3hdm1 | Vtn | Acsl5 | Pax5 |
| AC171111.1 | Baiap2l1 | Top1 | Xrra1 | Ano3 | Gm4241 |
| Cecr2 | Tmem192 | Vill | Gm16201 | Platr23 | Wnt5a |
| Ly75 | 4930520O04Rik | Mbd5 | Ammecr1l | Gm16897 | Rnf227 |
| Srl | Rxra | Gm47155 | Scamp3 | Ttc9c | Slc38a6 |
| Neurod2 | Rab38 | Rapgef3 | Gm26808 | Amh | Mks1 |
| Lmo7 | Zfp185 | Fam83d | 2610524H06Rik | Ppp2r1b | Gm14125 |
| Fsd1l | Ccdc65 | Gm37490 | Ly6g6f | CT025659.3 | Lpar3 |
| Ermp1 | Sst | Cst6 | Ccdc33 | Esco1 | Shisal2a |
| 2010300C02Rik | Fam131c | Ano3 | Gm38414 | Epn2 | Ncstn |
| Arpp19 | Togaram2 | Trim9 | BC017158 | 0610039K10Rik | Itih2 |
| Rnf19a | 2010001K21Rik | Phf24 | Tspan32 | Nrxn1 | Ctxn2 |
| Asxl3 | Drd2 | Lrrtm2 | Gm12033 | Fignl1 | BC067074 |
| Cyp7b1 | Daw1 | Gm38394 | Cryz | Zeb2 | Egflam |
| Wipf3 | Uox | Myt1l | Ccdc78 | Trerf1 | Acta2 |
| Gatad2b | Barhl2 | Dpp8 | Spire2 | Cplx3 | C230014O12Rik |
| AC151284.2 | Oxt | Pak7 | Fhl1 | 2810408A11Rik | Vim |
| Tnr | Aldh2 | Thrb | 9-Mar | Eif3s6-ps2 | Slc6a12 |
| Fgd4 | Mrps6 | Egr1 | Prss35 | Anxa3 | Gm35850 |
| Rbpj | Acss3 | Ppp1r12b | Tbxa2r | Amd-ps4 | Mtfr1 |
| Gpc1 | Gm16702 | Mmp16 | Aprt | Lyn | Ubxn6 |
| Cdk19 | Gpank1 | Tet2 | Palm3 | Plxnd1 | Prap1 |

| | | | | | |
|---|---|---|---|---|---|
| Smurf2 | Tns4 | Dzip1l | Frat2 | Igsf11 | Foxo6os |
| Cd33 | Cacna2d2 | Gm44941 | Tmem129 | Lix1 | Efcab10 |
| AI115009 | Kif9 | Pknox2 | Lrp2 | Ppm1h | Ltbr |
| Ablim3 | Mdfic | Brinp3 | Trmt112 | Sms-ps | Dusp15 |
| Pcdhb18 | Gm45470 | Atrnl1 | Arl2 | Frg2f1 | Tgtp1 |
| Draxin | Dusp26 | Mki67 | Foxc1 | Kcnh3 | Slc26a11 |
| Zfpm1 | Ucma | Azin1 | Cxcr4 | Fam19a2 | Nid2 |
| Lrrc4 | Gad1 | Vps33a | Trpm5 | | Uox |
| 2700081O15Rik | Bmp6 | Ppm1k | Pnpo | | Rpl9-ps8 |
| Lmnb1 | Nppc | Magi3 | Ndnf | | 4833417C18Rik |
| Unc13a | Cfap44 | Anks1b | Prmt2 | | 5930422O12Rik |
| Kmt2c | Sncb | Mef2c | Ap2s1 | | T2 |
| Cxadr | Fras1 | Paqr8 | Mpdu1 | | Iqcg |
| Agfg1 | Gm867 | AI464131 | Blcap | | Nupr1l |
| Zmym1 | Ddt | Gm26910 | Coprs | | Nts |
| Mfap3 | BC067074 | Atxn1 | Rcn3 | | Prph2 |
| AI593442 | Lrguk | Magi1 | Morn5 | | Gm43690 |
| Golm1 | 5930420M18Rik | Ncf1 | Relb | | Tspan4 |
| Iqgap2 | Gm2694 | Gm47415 | Nme6 | | Gm9772 |
| A330008L17Rik | Sh3bgrl2 | Gpt2 | Gck | | Tex15 |
| 9430081H08Rik | Cxxc4 | Egr2 | Fam183b | | Il22 |
| Tnks2 | Wdr63 | B230334C09Rik | Ifitm3 | | Rslcan18 |
| Ccdc88a | Lnx1 | AC110166.2 | Nfu1 | | Glra1 |
| Rras2 | Anapc13 | Arih1 | Gstm6 | | Sun3 |
| Sfpq | Fgf18 | Sh3rf3 | Uchl3 | | Mlkl |
| CT010433.2 | Krt8 | Amd1 | 4933431K14Rik | | Myof |
| Sall3 | Fgf11 | Zfp106 | Hps6 | | BC035947 |
| Esyt2 | Ifi27 | Rab11fip3 | Trappc2l | | Rasgrp2 |
| N4bp2l2 | Dlec1 | Zhx3 | Anapc11 | | Cep112it |
| Rps6ka3 | Sh2d4a | Blnk | Lypla2 | | Angpt1 |
| Ltbp2 | Sh3rf2 | Washc2 | Gm1821 | | Foxl1 |
| Psen1 | Gm49330 | Arhgef9 | Ttll8 | | Nrsn2 |
| Bmp2k | Oaz1 | Gm13601 | Gm33543 | | Nccrp1 |
| Kcnt2 | Ptpru | Gm9899 | Dut | | Cfap206 |
| Rock1 | Cryzl2 | Ifih1 | Nupr1l | | Steap2 |
| Cdh9 | Iscu | 4933431E20Rik | Pafah1b3 | | Scn7a |
| Tmem132b | Abhd16a | Gm44230 | Ebf4 | | Gm24187 |
| Acvr2a | Acox2 | Sowahb | Cdh24 | | Ankrd37 |
| Arl15 | Vwa5b1 | Rb1cc1 | Vat1 | | Slc41a3 |
| Gm44241 | B130034C11Rik | Gm5869 | Tap2 | | Eppk1 |
| Camkk2 | Zcchc18 | Unc13b | Txndc15 | | Gm13568 |
| Cacna1e | Nkx6-2 | AC165278.1 | Efemp2 | | Rd3 |
| Qser1 | Mea1 | Gm47232 | Lhfpl5 | | Cars2 |
| Nckap1 | Cpn1 | Resf1 | Slc27a2 | | AF529169 |
| Acap2 | Polr2m | St6gal2 | Cldn19 | | Fbln5 |
| Nrn1 | Ppp1r36 | Mettl11b | Gm48865 | | Nipal4 |
| Gnaq | Tmem176a | Gm2824 | Nenf | | Ucma |
| Samd12 | Dmpk | Pitpnm2 | Nap1l5 | | Gpat2 |
| Nwd2 | Dmkn | Jakmip2 | Manbal | | Nhlrc4 |
| Ghsr | Wdr66 | Ppp1r16b | Plin2 | | Ache |
| Dtl | Aifm3 | Ccdc88a | Gm26109 | | Spag16 |
| Camk1d | Gm32817 | AC118542.3 | Lyplal1 | | Rcc1 |
| Ikzf1 | Gm49427 | Fkbp5 | Gli1 | | Htra2 |
| Zfp729a | Cdh22 | Pgr | Mtch1 | | Col6a3 |
| Mpped2 | 1700024G13Rik | Dock2 | Gm9484 | | Tbc1d2b |
| Ppp2r5c | Prr32 | C730002L08Rik | Smim17 | | Fgl2 |
| Vcpip1 | 1700012B09Rik | Igfn1 | Gm9885 | | Tfap2b |
| Slitrk3 | Nxph1 | Nsd2 | B130024G19Rik | | Kcne1l |
| Irak2 | Slco1a5 | Cebpg | Wrb | | Clca1 |
| Mib1 | Sdr39u1 | Palm2 | Ndufs7 | | Gpx3 |
| Vps26b | Six3os1 | Ube2cbp | Retn | | Ccdc81 |
| Ssh2 | A230072E10Rik | Ankrd45 | Fxr2 | | Cldn3 |
| Zfpm2 | Chga | Faxc | Asgr1 | | Rarres2 |
| Zfp72 | Exosc5 | Abcc4 | Nrip2 | | Cnn1 |
| Paqr9 | Lhx5 | Flt1 | Sgcg | | Osr1 |
| Gm37824 | Dhrs11 | Dock3 | Sdc1 | | Gm9484 |
| Capn3 | BC064078 | Htr5a | Inafm1 | | Ubxn10 |
| Dmrta2 | Ctbp2 | Dkkl1 | Wfdc17 | | Cfap126 |
| Scn3a | Zfr2 | Grm3 | 3000002C10Rik | | Nckap5los |
| Ackr2 | Prune2 | Abcb7 | Prr3 | | Kcnj15 |
| Tet3 | Ikbip | Rabep1 | Nsg1 | | Gm11738 |

| | | | | |
|---|---|---|---|---|
| Mctp1 | Slc6a7 | A830018L16Rik | Gm805 | Tspan33 |
| Slco5a1 | Ccdc187 | Myh2 | Col5a3 | Abhd14b |
| Tbc1d20 | Pax5 | Ppp1r9a | Rac3 | Ank1 |
| P2ry13 | 4933406B17Rik | Csf1r | H19 | Tuft1 |
| Gm14662 | Ddrgk1 | Phka2 | Nipsnap3b | Ring1 |
| Mex3b | Plekha7 | Kcnq3 | Slc22a4 | Pi4k2b |
| Map4k5 | Vstm2b | Gm16234 | Tmem179 | Apeh |
| 3-Sep | Dleu7 | Tex13c2 | Atraid | Pth1r |
| Slmap | Lgr5 | Dgat2l6 | Card19 | B230319C09Rik |
| Emx2 | Aox1 | Tbc1d30 | Ffar2 | Ccdc154 |
| Spred2 | Crhr2 | Fam111a | Kctd15 | Igf1 |
| Map3k5 | Lrp2 | Rab6b | Cep295nl | Atp11c |
| Slc7a2 | Vstm5 | Ipo9 | Unc5cl | Parp3 |
| Nrep | Gabrg1 | Trhr2 | Zfp568 | Siglecg |
| Prkd1 | 1700023F06Rik | Abcd2 | Aspscr1 | Gm37477 |
| Bend4 | Cend1 | Fam168b | 4933400C23Rik | Thbs1 |
| Homer3 | Rsph6a | 4930488L21Rik | Tcf3 | Tmem205 |
| Ripk2 | Acp7 | Mkl2 | Dnali1 | Mfsd7a |
| Fosb | Mirg | Smg7 | B630019K06Rik | Tarm1 |
| Ss18l1 | Gm41414 | 11-Sep | Pex10 | Slc35d3 |
| Tle4 | Mfsd7a | BC018473 | Fam210b | Efcab1 |
| Qtrt2 | Gm4799 | Daam2 | Col1a1 | Gpc3 |
| Clstn2 | Rab3b | Sbno1 | Dnph1 | Ccdc9b |
| Ctdspl2 | mt-Ts2 | Gm40493 | Soat2 | 6430511E19Rik |
| Mn1 | Maged2 | Rps6ka3 | Cutal | Gm36638 |
| Erc2 | Ccdc28a | Scai | Cd1d1 | Marveld3 |
| Med13l | Gsta4 | Rfx7 | Lncppara | Rbp3 |
| Cnksr2 | Gm14597 | Plxnd1 | Polr2f | Ccdc96 |
| Ryr2 | Hdc | Klf7 | Rpusd3 | Barhl1 |
| Tmem114 | Casp1 | Synj2 | Tmem147 | Pcgf1 |
| Srsf10 | Aifm2 | Hipk3 | 6820408C15Rik | Rsph1 |
| Top1 | Gm34721 | Gm37069 | Dnajc17 | Cbln2 |
| Nrde2 | 4833427G06Rik | Nlk | Ctu1 | Gm44696 |
| Gm26801 | BC029722 | Rasgrf2 | Kif6 | Irf4 |
| Cdc42ep4 | Cdc42ep3 | Astn2 | 2700046G09Rik | Eci3 |
| Gm7237 | Spag8 | Dapk2 | Higd2a | Rnf149 |
| Diras2 | Gm42572 | Gm16183 | Hddc2 | Wdr6 |
| Pcdha5 | H2-Q2 | Cacna1i | Rftn1 | Gm38155 |
| Lamc1 | Gm16201 | Gm765 | Stmn3 | BC024139 |
| Hivep2 | AI854703 | Ghrh | Dok7 | Dcdc2a |
| Ncor1 | Zfp385a | Med1 | B3gnt9 | Cmah |
| Usp12 | Ephx1 | Gm11827 | Crispld2 | Gm11629 |
| Trib2 | Gpr149 | Ank | Sgce | Zfp966 |
| Ddx46 | Scd4 | Sox5 | S1pr2 | Rbfa |
| Sort1 | Oxld1 | Gna13 | Ngb | Fgf18 |
| Zfp101 | Tacstd2 | Nrros | 9430038I01Rik | Itih3 |
| Rrm2 | Smkr-ps | Ighm | Gm24993 | C2cd4d |
| Ntf3 | Hs3st2 | Klf6 | Tex14 | Cartpt |
| Plcb1 | Hsf4 | Gm12854 | Trappc1 | Ppp1r32 |
| Iqsec2 | Catip | Fry | Gm13919 | Dbp |
| Grin2c | Glp2r | Clvs1 | Slbp | Srebf1 |
| Rapgefl1 | 6430571L13Rik | Manf | Lrrc36 | Ppp1r3e |
| Arhgap4 | Wdr78 | Kdm3a | Aif1l | Krt87 |
| Zfp26 | Armc4 | Map3k6 | Oit3 | Tceal9 |
| Ppm1h | 4933407L21Rik | D130017N08Rik | Kdelr3 | Cdk5rap2 |
| Egfem1 | Frs3 | Nyap2 | Gm35024 | Arap1 |
| Lnpep | Gm18180 | Bicral | Fam181a | Rps6ka5 |
| Dclk1 | Gcgr | Slf2 | Rps16-ps2 | Mycbp |
| Scara3 | Gm10076 | Rbm28 | Tcf15 | Wdr33 |
| Tanc2 | Scn7a | Gm6277 | Pou4f2 | Btla |
| Slc4a7 | Dnah1 | Ighg2c | Fam89a | Sgce |
| Pfkfb3 | Mybpc2 | Phactr1 | Romo1 | St8sia6 |
| Ier5 | Smarcd3 | Il7r | Gm28982 | Mt2 |
| Abr | Tmem132e | Pdzd2 | Gm14066 | Unc45b |
| Kcna4 | Gm42868 | Pdlim1 | Gm10167 | Alpk3 |
| Zfp738 | Snrnp25 | Kcna5 | Hscb | Mab21l2 |
| Gm37452 | 1700001C02Rik | Trim33 | Frat1 | Oaz1 |
| Dgat2 | Bscl2 | Rbl2 | Fam43b | AC171205.1 |
| Desi2 | Gm29683 | Grm8 | Fam69a | Insrr |
| Wapl | Ergic3 | Zfyve9 | Pdlim2 | Eif2s3x |
| St6gal2 | Ift22 | Cd34 | Rwdd2a | Rln3 |

| | | | | |
|---|---|---|---|---|
| Washc4 | Mgst3 | Spred1 | Npc2 | Rdh5 |
| Pcdh20 | Sbsn | Clock | 9530052C20Rik | Eya2 |
| Kcnh7 | Coa4 | Abca7 | Mrps7 | Cfap70 |
| Rab11fip2 | Gm43210 | Mpl | Gm26876 | Tm6sf2 |
| Mylk3 | Dnajc27 | Atxn3 | Zeb2os | Lpin1 |
| Zbtb1 | Calml4 | D430041D05Rik | Gm48819 | Gm33756 |
| Rc3h2 | Gm49484 | Fgd6 | Gnpda1 | Gprin2 |
| Foxn2 | Fbxl12 | Megf10 | 1810010K12Rik | Rdh10 |
| Cggbp1 | 2610318M16Rik | Bcr | Phf1 | Trpm2 |
| Stxbp5 | Gm17180 | Purb | Fsd2 | Magel2 |
| Lrp4 | Spata6 | Rxfp1 | Ifnar2 | Aspscr1 |
| Gm43322 | Nbdy | Gm35040 | Araf | Ebf3 |
| Fhl5 | Ccdc113 | Usp2 | Mmp9 | Sox14 |
| Prox1os | Ppp1r1b | Gimap3 | Psmb3 | Ogn |
| Abcb7 | Dnajb13 | Ankrd17 | Tbx18 | Gm10754 |
| Nrip1 | Myof | Gm37529 | Gm9522 | 4933437G19Rik |
| Gm42853 | Grin3a | Arhgef19 | Gstp2 | Spata6 |
| Ccng2 | Fbxw9 | Wasf2 | Tcea3 | 2310009B15Rik |
| Gng2 | Mrgprh | Ascc3 | Gjd2 | Gjd2 |
| Btbd8 | Tbcb | Hexb | Mindy1 | Cd9 |
| Gm37124 | Six4 | C130083M11Rik | Efcab12 | Cplx4 |
| Arl5b | Jhy | Rybp | Ifi27 | Sntg2 |
| Calb1 | Cdhr3 | Grid2 | Med20 | Col9a2 |
| Pcsk2 | Pnma3 | Gm28928 | Zic5 | Mettl13 |
| B3gnt5 | A530072M11Rik | Synj2bp | Acads | Mlf1 |
| Myo9a | Samd3 | Tmem63c | Fuca1 | 1700007K13Rik |
| Zbtb41 | Rnf207 | Rnf169 | Uba52-ps | Irx5 |
| Mertk | D830030K20Rik | Zfp236 | Scml4 | Lrsam1 |
| Camkk1 | D730003K21Rik | Phtf2 | Gm13315 | Zfp868 |
| Ackr3 | Enkur | Tlr7 | Nme9 | Tsg101-ps |
| Chn1 | Vwc2 | Osbpl8 | Barhl2 | B630019K06Rik |
| Gm37285 | Crtac1 | Zmynd8 | Taf10 | Gm42572 |
| Kif26b | Csrp2 | Kcng3 | Hes6 | Gm49322 |
| Zfp266 | 4933431K14Rik | Prodh | Mfsd7a | Otof |
| Arhgap15 | Gm14539 | Yae1d1 | Gm37583 | Tdrd7 |
| Pik3c2a | Trim62 | Iqcn | Gm10481 | Tekt5 |
| C1ql2 | Cfap69 | Gm13716 | Sh3bgr | 3425401B19Rik |
| Rnf2 | Mns1 | Glul | Gm45694 | Bag3 |
| Fgf5 | Tctex1d1 | Gm13306 | Lmna | Mab21l1 |
| Gm3364 | Gm15417 | Haus3 | A930024N18Rik | mt-Te |
| Tpx2 | Iqub | Gm35853 | Gm4926 | Vps11 |
| 2700049A03Rik | Cfap43 | Pex5l | Akr1b3 | Fgg |
| Pwwp2a | Grik1 | Srpk2 | Myb | Htra3 |
| Cebpd | Grhl3 | Pclaf | Fam162a | Ccr2 |
| Cebpa | Cfap77 | Gm5171 | Sparc | Ajuba |
| Panx1 | Zdhhc1 | Mapkbp1 | Gsdma | Sec14l4 |
| Mef2d | Tjp3 | Pcdhb16 | Pcgf1 | Agt |
| Gm44633 | Chil5 | Gpatch8 | Gm33869 | Tmem192 |
| Med13 | Cdh7 | 1110019D14Rik | Efcab1 | Flna |
| Ddx6 | Zfp618 | Lrp8 | Unc119 | Plek2 |
| Itpr1 | Got1l1 | Lrp4 | Ly6h | Xpnpep2 |
| Tdg | Meg3 | Enpp4 | Mrpl54 | Gpat3 |
| Eya1 | B630019A10Rik | 4930447M23Rik | Egflam | Gm30054 |
| Abcd2 | Gm3739 | Dtna | Uox | Gm805 |
| Arnt | Xylb | Ankle2 | Ribc1 | Cfap52 |
| Kat6b | Zcwpw1 | Htr2a | Ccdc114 | Bace2 |
| Nbea | Gsta3 | Man2a1 | 6530409C15Rik | Gm36325 |
| Kcnd2 | Sntn | Gm45750 | Hes5 | 4930517J16Rik |
| 2510009E07Rik | Pbx3 | Kdm7a | Lrrc25 | Gm11611 |
| Gm19963 | Serpinb1b | Trerf1 | Nkx6-1 | Arhgef16 |
| Robo1 | Soat2 | Tmem181c-ps | Fabp7 | Gm26614 |
| Pgbd5 | Zmat3 | Garnl3 | Uncx | Tsnaxip1 |
| Ap3m1 | Rab10os | Cramp1l | Ndufv1 | Tal1 |
| Carnmt1 | Insrr | Tmc1 | Slc6a13 | Scn9a |
| Uhrf1 | Hspbp1 | Cxcl12 | Tesmin | Gm23369 |
| Arhgap12 | Cfap61 | Gm28836 | Hist1h1c | Isyna1 |
| Abhd17c | Gm15884 | Rasgef1c | Cd63 | Adam4 |
| Atf7ip | Large2 | Fam81a | Gm38037 | Katnal2 |
| Klhl2 | Ccdc151 | Pkp1 | Gfap | Col4a6 |
| Tspan5 | Gm39244 | Car10 | Amer1 | Xpc |
| Fbxo7 | Gm38414 | Calr | S100a11 | Dpy19l2 |

| | | | | |
|---|---|---|---|---|
| 1700020I14Rik | Mcrip2 | Usf3 | Col1a2 | Ngfr |
| Sema6d | Mir124-2hg | Trim37 | Gm37906 | Tead2 |
| Skint3 | Hint1 | Emcn | Gm15478 | Gm44597 |
| Dpysl3 | Tenm4 | Ccdc129 | Gm29865 | Cbr2 |
| Lrrn3 | Snx8 | Zbtb38 | Tfap2b | Zfp618 |
| Limd2 | Esrra | Sel1l3 | Tubb2a-ps2 | Fsip1 |
| Sipa1l2 | Gm23706 | Mfsd2a | S100a10 | Fam222a |
| Hpgds | Ap1s2 | Aldh1l2 | Zic2 | Islr |
| Gm10604 | D930020B18Rik | Spocd1 | Cdc42ep4 | 1300017J02Rik |
| Zfp867 | Ttc12 | Gpha2 | Trim27 | Gm11427 |
| Arih1 | Esrrg | Vip | Gm11629 | Tmigd3 |
| Gpr68 | Samd11 | Ncoa1 | Ddah2 | Gm42913 |
| Chst11 | Podn | Flrt2 | Tmem199 | Cryzl2 |
| Siah1a | Gipc1 | Pou3f2 | Uckl1 | Prss53 |
| Ptgs2 | Col5a3 | Gm14612 | Mrpl34 | Ndnf |
| Stat3 | Dnah12 | Gm17275 | Fibp | Wdr78 |
| Slitrk5 | Isl1 | Abhd15 | Ccdc180 | Pcgf2 |
| Lrrc57 | Fam161b | Nedd4l | Smim12 | Ppp1r14a |
| Mgll | Plch2 | Pik3c2a | Gm973 | Lmf2 |
| Tmem178 | Zfp768 | Iqcm | Gm45716 | Barhl2 |
| B3galt1 | Prdx4 | Zbtb34 | Cpne2 | Ccdc184 |
| Ankhd1 | Epn3 | Kalrn | Gm14486 | Tmem255a |
| Clcn4 | Vwc2l | Rgs7bp | A430105J06Rik | Sap30bpos |
| Prdm8 | Mmp9 | Tmem52 | Ccpg1os | Lhfpl5 |
| Zmym2 | Mrgpre | Pi4ka | Nlrx1 | Atp8b2 |
| Pgm2l1 | Kif6 | Tmem119 | Cps1 | Hhat |
| Olfm1 | Gm15587 | Pcdha3 | Col13a1 | Gm4756 |
| Gm35037 | Slc4a1 | Slc24a2 | Enpp1 | Tmem8 |
| Zfp518b | Clic6 | Atrx | H2afj | Oprk1 |
| P2ry6 | Atp5d | Tktl1 | Mri1 | Slc16a12 |
| Atp8a1 | Dlx5 | Gm47583 | Gprasp2 | 1810010K12Rik |
| Gpr63 | Gm29154 | Ubr2 | Gm49314 | Tatdn3 |
| Fbxo34 | Pgrmc1 | Txndc2 | Cnpy2 | Ypel3 |
| Scfd2 | Spg7 | Npc1l1 | Hdac11 | Kif27 |
| Sh3bp5 | Fam181a | Gm13026 | Ptch2 | Zfp503 |
| Stc2 | Gm32224 | Mlh3 | Tmem176a | Col6a2 |
| Gm5083 | Rhov | Clip1 | Gstt2 | Gbx1 |
| Acot3 | Camk2n2 | Atp2b4 | Gm8495 | Cthrc1 |
| Slc26a10 | Cbarp | Akap6 | Gm28095 | Cfap77 |
| Sh3bp2 | Ucp2 | Mkln1 | Ptges | Wisp2 |
| Mpl | Ptgr2 | Mtag2 | Npffr1 | C920021L13Rik |
| Prex1 | 2010320M18Rik | Lpgat1 | Lmo2 | Dse |
| Prkca | Smim12 | Strbp | Gm43429 | Enkd1 |
| Tsr1 | 9330158H04Rik | Gm21814 | Rbp3 | Igf2bp1 |
| Gm42864 | Bc1 | Slc19a3 | Ist1 | BC034090 |
| Ppp1cc | Gm17455 | Plekhm3 | 1810059H22Rik | Gk5 |
| Ap1s3 | Krt2 | Dennd4a | Ino80b | Sost |
| Abi1 | Lrrc74b | Rps27a-ps1 | Cd24a | Ano6 |
| Gm15624 | Kif27 | Gpr155 | Gm45453 | Irx6 |
| Nfya | Dlx2 | 9130227L01Rik | Ccdc146 | Cfap73 |
| Rcor1 | Glyat | Entpd1 | Pomgnt2 | Kctd15 |
| Slc30a6 | B130024G19Rik | Gm28455 | Nkx2-2 | Gm5345 |
| Gm38340 | Trmt112 | Pgm2l1 | Naa20 | Cep164 |
| Nebl | Ccdc33 | Fsd1l | Zfp777 | Plekhb2 |
| Tmem74 | Slc4a5 | Gm13861 | Wdr83os | S100a8 |
| Cpeb1 | Mtfp1 | Top3b | Gm23441 | Exosc3 |
| Arfgef3 | Col8a2 | Tlk1 | Gm44386 | Clrn1 |
| Smad4 | Gprin2 | Gm36251 | Gm9903 | Efcab11 |
| Gng10 | Pde6a | Gm47102 | Atp5s | Adgb |
| Tmem204 | Cdr2 | Tmcc1 | Mapk7 | Prelp |
| Fam84a | Lsm6 | Dlg1 | Timm17b | Slc30a7 |
| Ppp3cb | 1700001L19Rik | Mtmr2 | Smim4 | Gm7045 |
| Foxk1 | Nhs | Ntm | Gm36634 | Nsun7 |
| Fbxo11 | Gm28119 | Zfp148 | Gm8250 | |
| Pxylp1 | Rom1 | Nat8f3 | Pfn1 | |
| Gm45643 | Flnc | Knstrn | Washc3 | |
| Dcp1a | Cdh4 | Tpbg | Col3a1 | |
| Zfx | Mrpl58 | Fam49a | Gm13112 | |
| 2900022M07Rik | Efnb2 | Vcam1 | Snapc2 | |
| Ppm1l | Agt | Gm12371 | Tbx4 | |
| Amer2 | Ndufv3 | Gm13477 | Gm12324 | |

| | | | |
|---|---|---|---|
| Pum1 | Thap3 | Gm19445 | Pyroxd2 |
| Ppp3r1 | Apoc1 | Gm26742 | Mrps26 |
| Hunk | Col9a1 | Tk1 | Socs1 |
| Scarb1 | Nkx2-1 | Dusp14 | Slc16a13 |
| Wnt2 | Nrbp2 | Gm9796 | Selenoh |
| Fbrsl1 | Acsf3 | Hivep1 | Vim |
| Usp38 | Galk1 | Adamtsl5 | 9430034N14Rik |
| Slf2 | Wdr72 | Arhgap10 | Myl9 |
| Sp4 | Uimc1 | Lad1 | Atp5g3 |
| Hcfc2 | Sost | Fzd3 | Gm2897 |
| Zfp831 | Gm15478 | Fam160a2 | Wfdc3 |
| B4galt6 | Dlx1 | Efcab5 | Itm2c |
| Spin1 | Gm44109 | Prkcz | Pex11b |
| Dlg3 | Esyt3 | Sult1a1 | Bdh1 |
| Ppp6r3 | Dgat1 | Adam10 | Alg5 |
| Ogfrl1 | Cib2 | Vav1 | St8sia2 |
| Kdm5b | Hfe | 6430584L05Rik | Cdk4 |
| Chrm1 | Cfap221 | Tgm3 | Tgif1 |
| Hexb | 1810044D09Rik | Necab1 | Alox12 |
| Mosmo | Psmg1 | Btaf1 | Tm4sf5 |
| AC154486.3 | Cpa6 | Gm17322 | Foxd1 |
| Socs7 | Zswim3 | Gm38352 | Ccdc154 |
| Prdm2 | Klf5 | Il1rapl2 | Ccdc121 |
| 3110001I22Rik | Npbwr1 | Gm26833 | Psmd9 |
| Ino80d | Inafm1 | Gcnt4 | Slc8b1 |
| Kcnh1 | Morn2 | Bmpr2 | Tnfaip8 |
| Gm4202 | 1700018L02Rik | Zfyve26 | Tmed3 |
| Gm42492 | Clpp | Gm26795 | Trmt9b |
| Fkbp5 | Fam149a | Ripk2 | Lgals1 |
| Lrrc8c | Gm2670 | CT010433.2 | Gm17089 |
| Slc35a5 | Mir541 | Ncam2 | Gxylt2 |
| Enox2 | Myl3 | Creb1 | Lsm4 |
| Qrich1 | Gm47595 | D3Ertd254e | Faap20 |
| Celf2 | Slc22a17 | Irgq | C1ql4 |
| Mycbp2 | Blcap | Dusp3 | Crtap |
| Grm3 | Slc16a8 | Rnf125 | Ebf2 |
| Gpr158 | Gm20684 | Crebbp | Syne4 |
| Lysmd3 | Ndufa9 | Ppp2r2c | Pnkd |
| Rimbp3 | Dzank1 | Cntn3 | Etaa1os |
| Fam49a | Phf1 | Lrrc55 | Zap70 |
| Rell1 | Trim10 | Mosmo | Fbxl12 |
| Synpr | Mroh1 | Erfe | Vmn2r-ps24 |
| Asphd2 | Mroh5 | Bcl2l1 | Gm26911 |
| Klhl38 | Fkbp2 | Hdac4 | Rimkla |
| Prdm10 | Rtl1 | Fer | Ppp1r8 |
| Tlr3 | Odf3b | Ppp2r5c | |
| Clspn | Crb1 | Mtmr6 | |
| Zfp960 | Gm15943 | Prickle2 | |
| Gm45698 | Meis2 | Gm37788 | |
| Pcdhb4 | Spag17 | Mindy2 | |
| Zfp212 | Il33 | Pcdh10 | |
| Fbxl18 | Bdkrb1 | Gm18134 | |
| Gpsm2 | Acbd4 | Ank2 | |
| Otub2 | Nppa | Kcnt2 | |
| Ascl1 | Gm13629 | Arl4d | |
| Lonrf3 | Cryz | Gm29514 | |
| Rmnd5a | Hes1 | Zmat3 | |
| Chgb | Ppp1r3b | Marf1 | |
| B230219D22Rik | Amdhd1 | Tmem56 | |
| Sirt1 | 1700037C18Rik | Ddit4l | |
| Gm43355 | Sod1 | Usp15 | |
| Ube2cbp | Gas2l2 | Hsp90b1 | |
| Slc24a2 | Pyroxd2 | Ap3m1 | |
| Ulk2 | Katnal2 | Osbpl1a | |
| Cilp2 | Itga10 | Trbc2 | |
| Topbp1 | Rbm47 | Zfp260 | |
| Uhrf2 | Tceal9 | Trp53bp2 | |
| Tspan18 | Tspan9 | Itga9 | |
| Mmp16 | H2-M3 | Lmtk2 | |
| Gabra5 | Gm13807 | Cdk19 | |
| Gm43099 | Gm26610 | Gm26530 | |

| | | |
|---|---|---|
| Megf9 | 3-Mar | Gm21112 |
| Fam117b | Gm34045 | Cep290 |
| Gm37735 | Myl2 | Kcnj6 |
| Hcfc1 | Ap3s2 | Paqr5 |
| Tcf24 | Tbc1d9 | Vmn2r118 |
| Trim30a | Cfap65 | Lamc2 |
| Mgat4a | 1700080G11Rik | Rabl6 |
| Trpc5 | Mrpl14 | Erbb4 |
| Emid1 | Gm47372 | Cacna1b |
| Mta2 | Ctsf | Susd5 |
| Gpr85 | Cbln3 | Hpse |
| Mcl1 | Tal1 | Extl1 |
| Gm11839 | Ascc1 | AU041133 |
| 2610021A01Rik | Parm1 | Adam33 |
| Nudt10 | Rbfa | Nin |
| Gm9111 | Defb22 | Usp45 |
| Prickle1 | 4930539E08Rik | Cdc37l1 |
| Nptx1 | Nme6 | Gprin3 |
| 4933425D22Rik | Sox1 | I730030J21Rik |
| Arf4 | Meis1 | AI593442 |
| Mast4 | Myl6b | Cyld |
| Col4a1 | Tnfaip8 | Mtus2 |
| Clcn3 | Rd3l | Slc1a2 |
| Cysltr2 | Pifo | Nfkb2 |
| Fam84b | Gm10639 | Gli3 |
| Fam19a1 | Stmn3 | Trim43a |
| Nhlh1 | Cntnap3 | Gm8822 |
| Gm42636 | Elob | Cacng2 |
| Ybx1 | Lypd6b | Dclk3 |
| Zyg11b | Akip1 | Pum2 |
| Atp2c1 | Aebp1 | Gm13803 |
| Zfp324 | Fsd2 | Grin2c |
| Naa25 | Gm44597 | Plcb1 |
| Tril | Mgst1 | Mxd4 |
| 9330159F19Rik | Gm49373 | Nr4a2 |
| Rbm12 | Arhgap36 | Agtpbp1 |
| Gm19445 | mt-Tg | Papola |
| Plxnd1 | BC034090 | Rims4 |
| Ncoa1 | Grk3 | Gm37711 |
| Gm9761 | Renbp | Uggt1 |
| Dcun1d1 | Pnck | Gabra1 |
| Tbpl1 | Tsga13 | Ppfia3 |
| Gm46210 | Mak | Fam120a |
| Kcnq3 | Stoml3 | Atp6ap1l |
| Socs4 | Nov | Mtcp1 |
| Abcb10 | Timm17b | Pip5k1c |
| Jmjd1c | Spint2 | Ecpas |
| Igsf11 | Gm42427 | Gm12411 |
| Hecw1 | Gm43759 | Bsn |
| Vps37b | Gabrg3 | AC125351.1 |
| Pdlim1 | Chchd6 | Prdm10 |
| Kidins220 | Gm19531 | Zbtb37 |
| Sh3gl1 | Gm9794 | Gm43088 |
| Rnf38 | 4930526F13Rik | Fam53b |
| Bcl6 | Gm48898 | Zfp871 |
| Dhx29 | Gm29595 | Gcc2 |
| Rock2 | Oca2 | Cntn4 |
| Ralgps1 | Trim66 | Ppm1l |
| Med1 | Gale | Gm43813 |
| Fam3c | Khk | Dhx29 |
| Smpd3 | Thnsl2 | Zfp143 |
| Tapt1 | Pnpo | Gm18959 |
| Chm | Metrnl | Atf6 |
| Fam160b1 | Gm26902 | 4930515G01Rik |
| Slc30a3 | Pink1 | Map9 |
| Sgtb | Gm16287 | 2210408F21Rik |
| Gm42798 | Gm37968 | Ppp4r3b |
| Camsap2 | Mdh1 | Gm10118 |
| Dpysl5 | 1500009C09Rik | Chm |
| Zfp704 | Pde9a | Calm1 |
| Kdm2b | Dnaaf3 | Lmbrd2 |

| | | |
|---|---|---|
| Dnajb5 | Ubxn6 | Fbxw7 |
| C130071C03Rik | Ccdc114 | Pdzrn3 |
| Trim9 | Fam96b | Rasal2 |
| Isg20l2 | Ndufv1 | Tll2 |
| Il10ra | Calca | Ctnna2 |
| Snora57 | Tppp3 | Gm26280 |
| Celf1 | Slc5a6 | B3galt2 |
| Tcea1 | Prdm12 | Olfr464 |
| Pkig | Narfl | Zfp709 |
| Ighm | F2rl3 | Numb |
| Igsf6 | Tspyl2 | Ash1l |
| Ankle2 | Lrat | Incenp |
| Gm15894 | Cradd | Rsf1 |
| Ckap2l | Tram1l1 | Raph1 |
| Rbfox3 | Fam161a | Gm20163 |
| Nmt1 | Klc3 | Rph3a |
| Jmy | Gm26532 | Wnt2 |
| Ss18 | Gria4 | B230209E15Rik |
| Homer1 | S100a10 | Egr3 |
| Zfp281 | Atp2b4 | Bcor |
| Sema7a | Sdhc | Ptk2 |
| Tlk1 | Brd3os | Cdkl5 |
| Fam222b | Bbs2 | Cntnap5b |
| Gm37106 | Gm17018 | Pip4k2b |
| Arsb | Mrps24 | Tedc1 |
| Magi2 | 4930502E18Rik | Smim13 |
| Cog5 | Gm47403 | Gfra2 |
| Neurod6 | Rspo3 | Gm30340 |
| Crk | Kcnj9 | Slc35f1 |
| Sf3b1 | Neil2 | Nckap1l |
| Gm16551 | Glp1r | Sema7a |
| Mapk1ip1l | Gast | Gm37860 |
| Camk2n1 | Dcdc2a | Gm31518 |
| D16Ertd472e | Tmem240 | Cobl |
| Gm36823 | Dydc2 | Tpx2 |
| Pde4dip | Cckar | Efna5 |
| E030026E10Rik | Cntnap4 | Cop1 |
| Pou2f2 | Naxe | Igfbp1 |
| Nfix | Col6a2 | Zfr |
| Vps54 | Gm12070 | Hipk1 |
| Insr | Htr3a | Fam208a |
| Krr1 | Fndc9 | Brd4 |
| Rbm24 | Myh13 | Syne1 |
| Zkscan8 | Gm14323 | Rngtt |
| Pml | Angptl7 | Ivns1abp |
| Tnks | Ckmt1 | 4930506C21Rik |
| Frmpd4 | Zfp575 | Diras2 |
| Api5 | Ifitm10 | Nwd2 |
| Cep55 | Tnfrsf26 | AC116527.2 |
| Kdm4c | Vpreb1 | Gm30382 |
| Chrdl1 | Lhx1os | Gm45884 |
| Wdhd1 | Gm31518 | Man1a2 |
| Cd46 | Ypel3 | Cadm2 |
| 1810019N24Rik | Mlycd | Ttbk1 |
| Wdr26 | Tfap2a | Runx1 |
| Dlgap1 | Gm47414 | Ern1 |
| Onecut2 | Bpifc | Gm48552 |
| Acvr1 | Gm13814 | Kcnh3 |
| C630031E19Rik | Gpr55 | Gm48717 |
| Prc1 | Gpr88 | Heatr5b |
| Gm12532 | Xkrx | Zfp518a |
| Grm7 | Dohh | Fam13b |
| Gm48342 | 9-Mar | Camsap2 |
| Stxbp5l | Atp5b | Pcdhgb8 |
| Gal | Adrb3 | Pdzph1 |
| Gm47623 | Lbp | Gm46409 |
| Csnk2a1 | 9030407P20Rik | Gm47917 |
| Marcks | Aspscr1 | Kpna1 |
| Elk1 | Ebf2 | Ube3b |
| Dsel | Smyd1 | Snx16 |
| Tsc22d2 | Car10 | Zc3h12c |

| | | |
|---|---|---|
| Frmd4a | Xpc | Zfp276 |
| Ppp1r12a | Olfr1033 | Myo9a |
| Gabra4 | C77080 | Gm12689 |
| Mllt3 | Prss30 | Flvcr2 |
| Brinp1 | Gng4 | Gm553 |
| Kcnj11 | Cfap73 | Ckap2l |
| Dbh | Fbxo44 | Cspg4 |
| Cttnbp2 | Fsip1 | Golga4 |
| Zik1 | Tacr1 | AC154200.1 |
| Tmem196 | Gm44321 | Fn1 |
| Map9 | Osbpl7 | Adgrl2 |
| Gm20716 | Gm17383 | Usp34 |
| Strn3 | Gpc3 | Kcnk6 |
| Tmtc3 | Krt1 | Ankmy1 |
| Rragd | Pcsk4 | Dok5 |
| Rasa3 | Lyrm9 | Slc18a3 |
| Mbtps2 | Mst1r | Npy1r |
| Arntl | Exoc3l4 | Lancl3 |
| Gm14033 | Lrrc34 | Ttc7b |
| Ust | Oaz2 | Herc6 |
| Usp1 | Ccdc121 | Nr2c2 |
| Fut8 | Dpm1 | Slc16a7 |
| Pik3r1 | Cdkl4 | Gm43048 |
| Extl2 | Cby1 | Gm32122 |
| Glce | Ppp1r14a | Ptar1 |
| Lats2 | S100a9 | Slc30a4 |
| Serpina3n | Sfrp5 | Add2 |
| Zfp84 | Tsku | Rock1 |
| Cspg4 | Bex1 | Gm33016 |
| Slc7a5 | 4933439K11Rik | P2ry6 |
| Btn1a1 | Myo5c | Gm43389 |
| Tmcc1 | Tmtc4 | Etv5 |
| Cd163l1 | Rxfp3 | Rnf157 |
| Arhgap21 | Rdh1 | Foxp1 |
| Pcdhgb6 | Tecr | Gm12941 |
| Gm48538 | 4930572O13Rik | Fbxo41 |
| Usp22 | Gm4419 | Zfp507 |
| Ptpn12 | Gm33869 | Pank2 |
| | Mob2 | Zyg11b |
| | Gm48865 | Rgs12 |
| | Fam210b | Gm20535 |
| | Anxa2 | Creld2 |
| | Efnb3 | Gda |
| | 4933431C10Rik | Per3 |
| | Pvalb | Adap2 |
| | Gm48536 | Rpgrip1l |
| | Gm853 | Cntnap3 |
| | Nat8f1 | Tut7 |
| | Cab39l | Gpr26 |
| | Col5a1 | Pcmtd1 |
| | Cpq | Anln |
| | Wls | Ino80d |
| | Tspan4 | Gm3279 |
| | Sf3b5 | Zfp704 |
| | Crebl2 | Tmem151b |
| | Syne4 | Chn1os3 |
| | A730063M14Rik | Arpp19 |
| | AF529169 | Nipbl |
| | Mycbp | Osbpl6 |
| | Zc2hc1c | AC078895.2 |
| | Arhgef16 | Olfr111 |
| | Ano2 | Adam22 |
| | Lzts2 | Sik2 |
| | Dnah10 | Gm47439 |
| | Diras1 | Helz |
| | Atp4a | Gm38386 |
| | Gm47692 | BC024978 |
| | Tmem184a | Zfp397 |
| | Ilvbl | Cacna1a |
| | Gm35024 | Usp28 |
| | Gm42460 | Atp13a5 |

| | |
|---|---|
| Rdh5 | Akt3 |
| Mfrp | |
| Slc22a4 | |
| Cmss1 | |
| Wnt9b | |
| Kdm4d | |
| Gm9522 | |
| Chid1 | |
| Hs3st6 | |
| Sox6os | |
| Cisd1 | |
| Clca1 | |
| Ubb | |
| 4932438H23Rik | |
| Mpnd | |
| Rbms3 | |
| Tmem161a | |
| 9430034N14Rik | |
| Pcsk1 | |
| Mrpl40 | |
| Gm4285 | |
| Map1lc3a | |
| Mief2 | |
| Clba1 | |
| Gm15956 | |
| Gm26685 | |
| Gdnf | |
| Sub1 | |
| Acaa2 | |
| Ier3 | |
| Mir212 | |
| E430024P14Rik | |
| Gpx1 | |
| Sostdc1 | |
| Prdm16 | |
| Ccdc162 | |
| Tex9 | |
| Slc10a4 | |
| Cd59b | |
| Zbtb11os1 | |
| BC021767 | |
| Slc27a2 | |
| 4732491K20Rik | |
| Myo1h | |
| Gm16418 | |
| Aven | |
| Gm7854 | |
| Zfp966 | |
| Smim17 | |
| Dlx4os | |
| Copz2 | |
| Gm47936 | |
| Aqp1 | |
| Gm26753 | |
| Hspb8 | |
| Gm37573 | |
| Tnni3k | |
| C8g | |
| Abca8a | |
| Rwdd2a | |
| Ptpn20 | |
| Zfp819 | |
| Prr29 | |
| Spsb4 | |
| Ndst3 | |
| Tomm7 | |
| Ect2l | |
| Ccdc160 | |
| Cyb5rl | |
| Kdelr3 | |
| Car14 | |

Itih3
Gm9905
Mrpl13
Ap1s1
Gpx4
Bfsp1
Mogat1
Selenov
Plppr3
Rab26
Gm19744
Zbtb8b
Impa2
Mpp7
Gm26783
Tssk1
3110040N11Rik
Met
Prlr
Tmem134
Chdh
Ndufb11
Gm44907
Slc34a2
Mum1l1
Tsc22d4
Cacna1g
Gm47202
Tmco3
Sytl4
Gm32635
Bst2
Rhbdl3
Gm38973
Ace
Gpat3
Aldh3a2
Kcne1l
Gm26911
Akr1c18
Isyna1
AC154218.2
5730414N17Rik
Dppa5a
Lrrc43
Limk2
Phf2os1
6430500D05Rik
Clrn1
Kl
Wdr93
Gm23297
Gm37691
Pcp4l1
Gm26808
Tgfb3
Gm9908
Ripk3
Casc1
Krt19
Gabra6
BC030867
Dzip1
2010204K13Rik
Fn3k
Spef2
Adssl1
4933437G19Rik
Hs3st3b1
Tgtp1
Mthfsd

B9d1
Hexa
Slc6a5
Rcan2
Trmt11
Gm11465
2900040C04Rik
Gm23441
Enpp1
Eppk1
Miga2
Polm
Drc7
Gm46376
Ttr
Gm17747
Sord
Spr
Lum
Hdhd2
Sdhaf3
Nme9
Rnf25
Gm14325
Cadm1
Gm1673
Sult5a1
Gm37090
Akr1c19
Sulf1
A530058N18Rik
